# Direct regulation of a tRNA modification enzyme by ppGpp links stress signaling to translational control and biofilm development

**DOI:** 10.1126/sciadv.aek6976

**Published:** 2026-11-04

**Authors:** Aude E. Trinquier, Franck Slama, Junlang Liu, Lucille Pham, Lauren Stolt, Jue D. Wang

**Affiliations:** ^1^ Department of Bacteriology, University of Wisconsin-Madison, Madison, WI 53706, USA.; ^2^ Independent researcher, Madison, WI, USA.; ^3^ Department of Chemistry and Chemical Biology, Harvard University, Cambridge, MA 02138, USA.

## Abstract

Transfer RNA (tRNA) modifications influence decoding efficiency and translational fidelity, yet how they are integrated into bacterial signaling networks remains poorly understood. Here, we identify a direct connection between stress signaling and codon-specific translational regulation. We show that the alarmone guanosine tetraphosphate (ppGpp) directly inhibits MnmE (TrmE), a conserved tRNA-modification GTPase required for methylaminomethyl (mnm) modification of wobble uridine. High-resolution ribosome profiling in *Bacillus subtilis* revealed that loss of MnmE selectively increases ribosome pausing at AAG codons and reprograms translation of a subset of genes, including small open reading frames. Among these is a previously unrecognized AAG-containing leader peptide that controls expression of the biofilm repressor AbrB. Consistent with this mechanism, *mnmE* mutants exhibit constitutive extracellular matrix production and bypass the biofilm defect of a *relA* mutant. Together, these findings establish a pathway linking ppGpp signaling, tRNA modification, codon-specific translation, and multicellular development.

## INTRODUCTION

The epitranscriptome, the ensemble of posttranscriptional RNA modifications, has emerged as a universally conserved regulatory layer influencing translation from bacteria to humans (*1*–*4*). Transfer RNAs (tRNAs) are among the most heavily modified RNA species, with ∼10% of their nucleotides enzymatically altered (*5*). These chemical modifications modulate tRNA stability, folding dynamics, and codon-anticodon pairing, thereby tuning translation rate and accuracy (*6*–*9*). Perturbations in the tRNA modification landscape can lead to atypical translation events such as frameshifting, stop-codon readthrough, and ribosome pausing (*10*–*12*). Despite substantial progress, the mechanisms regulating tRNA-modifying enzymes and their coordination with cellular physiology remain poorly understood.

A key contributor to the tRNA epitranscriptome is MnmE (also known as TrmE), a conserved guanosine triphosphatase (GTPase) that, together with its partner MnmG (also called GidA), catalyzes methylaminomethyl (mnm) modifications at the wobble base (U_34_) of select tRNAs (*13*) to enhance codon-anticodon pairing (*14*, *15*). MnmE and MnmG are widely conserved across bacteria and eukaryotes, with mitochondrial homologs (MSS1/MTO1 in yeast, GTPBP3/MTO1 in humans), and are even retained in minimal genomes (*5*, *16*, *17*). MnmE and MnmG are required for bacterial pathogenicity (*18*), and defects in their human homologs cause mitochondrial diseases leading to infantile cardiomyopathy (*5*, *16*), highlighting their physiological relevance.

Despite the critical role of wobble-uridine modification in physiology, there has been limited evidence for its regulation. MnmE and MnmG form oligomeric complex: MnmE binds guanosine 5′-triphosphate (GTP) and 5,10-methylene-tetrahydrofolate, the one-carbon donor for the modification; MnmG binds flavin adenine dinucleotide (FAD) and reduced nicotinamide adenine dinucleotide (NADH), which participate in the redox chemistry required for the U_34_ modification (*13*, *19*). GTP hydrolysis by MnmE drives conformational changes of the MnmEG complex that orchestrate the (cm)nm^5^(s^2^)U_34_ tRNA modification (*20*, *21*), and its GTPase cycle is modulated by negative feedback from guanosine diphosphate (GDP) and inorganic phosphate to prevent excessive GTP consumption (*20*). In *Escherichia coli*, different growth conditions, as well as oxidative and acid stress exposure, were shown to affect MnmEG-mediated tRNA modification levels (*19*, *22*). However, the mechanism by which MnmE tRNA modification activity is regulated by environmental signals remains unknown.

A conserved signal of bacterial stress responses is the nucleotide (p)ppGpp (guanosine tetra- and pentaphosphate), which accumulates rapidly during starvation and other stresses to globally reprogram cellular physiology (*23*, *24*). (p)ppGpp down-regulates DNA replication, transcription, translation, and purine metabolism, thereby suppressing growth and promoting survival (*25*–*27*). In addition, (p)ppGpp reprograms gene expression to facilitate stress adaptation and developmental processes, including biofilm formation—a widespread lifestyle in which bacteria attach to surfaces through an extracellular matrix and form resilient multicellular communities (*28*). However, the mechanisms by which (p)ppGpp promotes these adaptive behaviors remain poorly understood.

In this study, we uncover a mechanism by which this stress signal directly regulates tRNA modification. We show that (p)ppGpp inhibits the tRNA-modifying GTPase MnmE, thereby linking stress sensing to wobble-uridine modification. Using genetic, transcriptomic, and ribosome-profiling approaches, we define the translational consequences of MnmE inactivation. Loss of MnmE increases ribosome pausing at lysine AAG codons, alters translation of stress and adaptation-related genes, and reveals the translation of numerous small open reading frames (ORFs), many of which were previously unannotated. Among these, we identify an *abrB* leader peptide (LP) that modulates translation of this master biofilm repressor. Genetic epistasis analyses further support that (p)ppGpp promotes biofilm formation through this MnmE-dependent pathway, suggesting a stress-responsive regulatory cascade that links tRNA modification, translational reprogramming, and multicellular behavior.

## RESULTS

### ppGpp directly binds to and inhibits the GTPase activity of MnmE

The GTPase MnmE (formerly TrmE) is a highly conserved tRNA modification enzyme responsible for wobble uridine (U_34_) modification of specific tRNAs (*13*, *29*, *30*). MnmE emerged as a strong ppGpp-binding candidate in our DRaCALA (Differential Radial Capillary Action of Ligand Assay)–based screens using the *Bacillus anthracis* overexpression library and ^32^P-labeled ppGpp, a result also observed in *E. coli* (*31*, *32*). We purified MnmE and its interacting partner, MnmG, from the closely related species *Bacillus subtilis*. Using DRaCALA, we confirmed that purified *B. subtilis* MnmE (Fig. 1A and fig. S1A), but not MnmG (fig. S1B), binds (p)ppGpp, with ppGpp showing stronger affinity (∼9 μM) than pppGpp. Unlike *E. coli* MnmE, which hydrolyzes pppGpp to ppGpp with a low efficiency (*32*), *B. subtilis* MnmE does not hydrolyze pppGpp (fig. S1C).

**Fig. 1.**
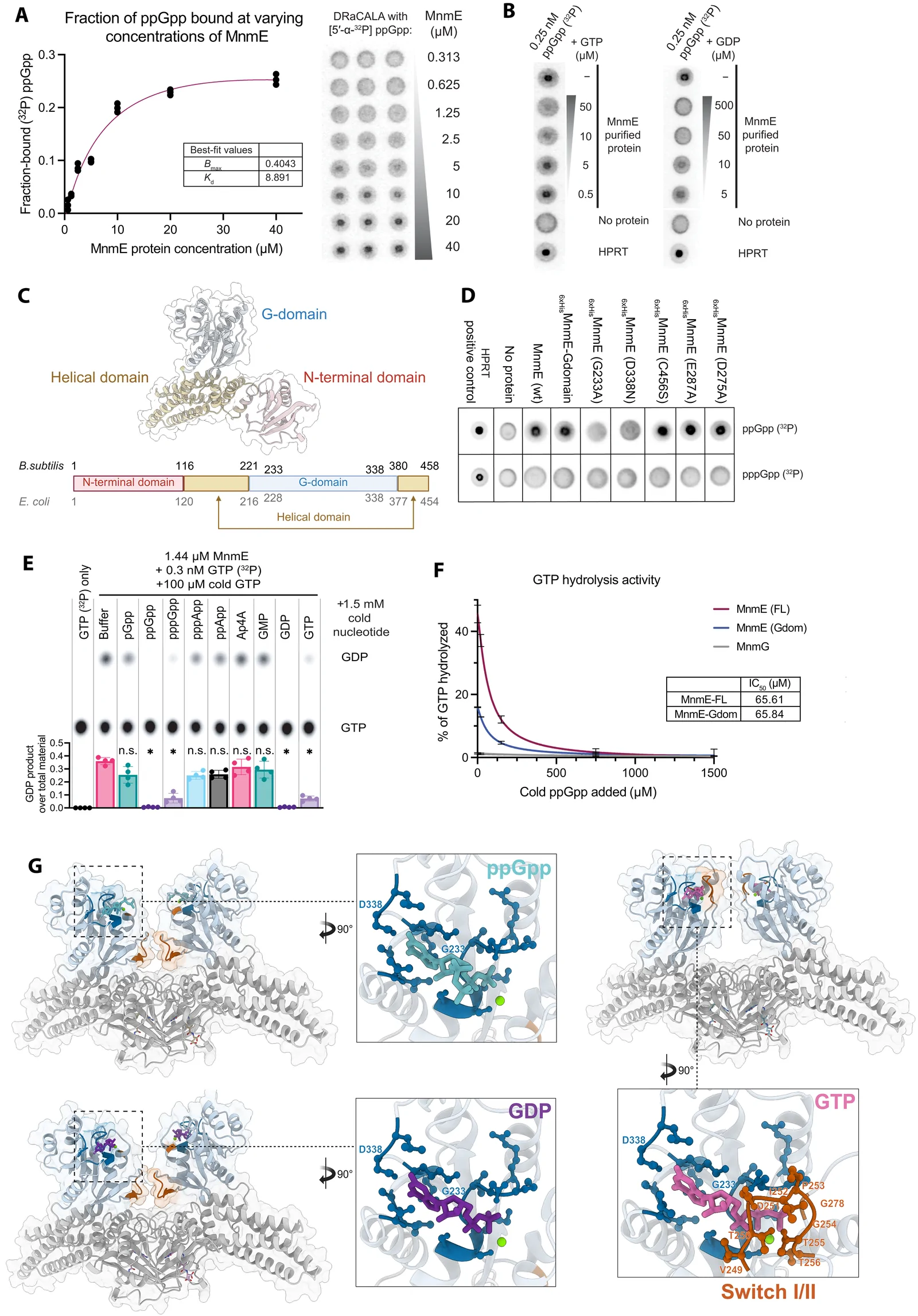
ppGpp binds and inhibits the MnmE GTPase. (**A**) DRaCALA analysis of 0.25 nM [5′-α-^32^P]–ppGpp binding to increasing concentration of purified *B. subtilis* MnmE. Fraction bound (*n* = 3) is shown with nonlinear regression fits and binding parameters [dissociation constant (*K*_d_); in micromolar). (**B**) DRaCALA of 0.25 nM [5′-α-^32^P]–ppGpp binding to 10 μM MnmE in increasing concentrations of unlabeled GTP or GDP. (**C**) Top: AlphaFold3 structure of MnmE monomer. Bottom: MnmE domain architecture with corresponding residues in *B. subtilis* and *E. coli.* (**D**) DRaCALA of 0.25 nM [5′-α-^32^P]–ppGpp and pppGpp binding to MnmE or its truncation or point mutation variants (10 μM). Hypoxanthine-guanine phosphoribosyltransferase (HPRT) protein is shown as a positive (p)ppGpp-binding control. (**E**) In vitro MnmE GTP hydrolysis in the presence of the indicated nucleotide inhibitors. Reactions contained 100 μM GTP supplemented with [α-^32^P]–GTP and 1.9 μM MnmE. Top: Representative thin-layer chromatography (TLC). Bottom: GDP production (mean ± SD; *n* = 4). Statistical significance was determined by repeated-measures one-way analysis of variance (ANOVA) with Dunnett’s correction (*P < 0.01; n.s., not significant). (**F**) GTP hydrolysis by MnmE full-length (FL) and G-domain (Gdom) proteins. MnmG served as a non-GTPase control. Reactions contained 100 μM GTP, [α-^32^P]–GTP, 1.9 μM enzyme, and indicated concentrations of ppGpp, followed by TLC analysis. Data are mean ± SD (*n* = 3); curves were fit by nonlinear regression to determine median inhibitory concentration (IC_50_) values. (**G**) AlphaFold3 predictions of the MnmE dimer bound to ppGpp, GDP, or GTP. The ppGpp-bound and GDP-bound models adopt similar conformations, with switch I and II regions (orange) positioned away from the ligand-binding site. The GTP-bound model undergoes substantial conformational reorganization, with switch I and II regions (orange; see close-up) folding over the ligand-binding site and bringing the G domains (light blue) into close proximity. Conserved residues required for GTP hydrolysis are in blue. Magnesium ions (green spheres) and formyl-tetrahydrofolate are also shown.

MnmE is a GTPase composed of three domains: N-terminal, helical, and the G-domain responsible for GTP binding (Fig. 1C). Competition assays demonstrated that both GDP and GTP compete for ppGpp binding to MnmE (Fig. 1B), and the truncated G-domain of MnmE is sufficient to retain ppGpp binding (Fig. 1, C and D), implicating the GTP-binding site in ppGpp interaction. We introduced point mutations in conserved residues critical for GTP binding and hydrolysis (*33*). Mutations known to abolish GTP binding in *E. coli* (G233A and D338N) also abolished ppGpp binding (Fig. 1, C and D), supporting the idea that ppGpp occupies the GTP-binding pocket.

The tRNA modification activity of MnmE is powered by GTP hydrolysis (*20*). Therefore, we examined the impact of (p)ppGpp on its GTPase activity using in vitro assays with ^32^P-labeled GTP. Both pppGpp and ppGpp inhibit MnmE GTPase activity, while other alarmones in *B. subtilis*, Ap_4_A and (p)ppApp (*34*), had no significant effect (Fig. 1E). We quantified GTP hydrolysis by full-length MnmE and a truncation variant containing only the G-domain, with varying concentrations of ppGpp. Both forms exhibited similar inhibition profiles, with median inhibitory concentration (IC_50_) values near 66 μM (Fig. 1F). These results indicate that ppGpp directly interferes with the GTPase activity of MnmE at physiologically relevant concentrations, as cellular ppGpp ranges from ∼10 μM basal level to ∼1 mM upon starvation (*35*). As a negative control, MnmG showed no GTPase activity.

To further characterize binding of ppGpp and its impact on MnmE conformation, we performed structural modeling with AlphaFold3, as the protein showed pervasive aggregation in vitro, precluding experimental structural characterization (fig. S1D). AlphaFold3 predicts that ppGpp binds tightly within the canonical GTP-binding pocket, and the ppGpp-bound MnmE adopts a conformation overlapping with the GDP-bound but different from the GTP-bound conformation (Fig. 1G, fig. S2, A to C, and table S4). In the GTP-bound state, switch I and switch II loops adopt a closed conformation, pulled inward by the γ-phosphate of GTP to form a tight, well-ordered interface between the two G-domains of the MnmE dimer. In contrast, ppGpp stabilizes a conformation resembling the GDP-bound state, in which switch I and switch II relax away from the nucleotide, loosening the G-domain interface. This open state has reduced affinity for tRNA and represents the off state of MnmE (Fig. 1G) (*20*, *33*, *36*), indicating that ppGpp inhibits MnmE-dependent tRNA modification by blocking its GTP utilization.

### Loss of MnmE-dependent tRNA modification promotes biofilm formation in *B. subtilis*

The physiological consequences of various tRNA modification defects range from no growth impairments to complete loss of viability (*7*). To evaluate the physiological impact of MnmE-dependent tRNA modification, we deleted *mnmE* or *mnmG* genes in *B. subtilis* NCIB3610. The resulting mutants have no apparent growth defect but displayed rugous colony morphology (fig. S3A), a phenotype frequently linked to biofilm, in which cells grow as matrix-embedded communities rather than as free-living individuals (*37*, *38*). Therefore, we tested biofilm formation both in liquid and on solid medium. Using pellicle assays to measure biofilm formation at the air-liquid interface of static culture (*39*, *40*), we found that ∆*mnmE* and ∆*mnmG* formed thick, highly wrinkled biofilm compared to wild-type (WT) cells (Fig. 2A). Both mutants produced pellicles not only in the biofilm-promoting MSgg medium but also in a control medium in which glycerol and glutamate were replaced with glucose and ammonia (MSGA), a condition that does not support biofilm formation in WT cells (Fig. 2A). Similar trends were observed in mesh-supported pellicle biofilms (fig. S3B) (*40*). In addition, on solid medium, ∆*mnmE*, ∆*mnmG*, and ∆*mnmE*∆*mnmG* mutants all formed biofilms with morphologies distinct from WT; mutant colonies were extensively wrinkled and lacked the smooth inner ring characteristic of WT colony biofilms (Fig. 2B) (*41*).

**Fig. 2.**
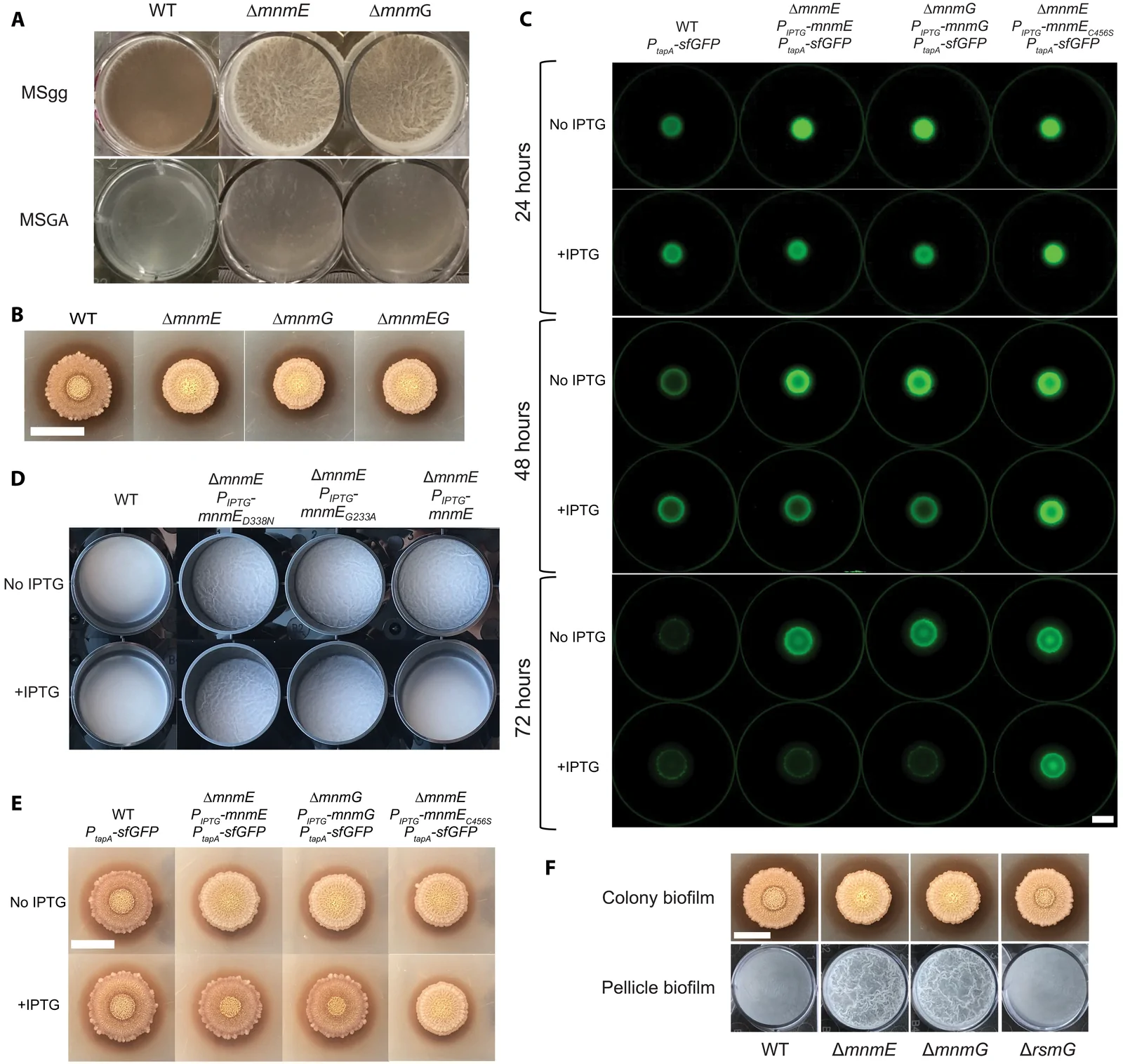
Loss of MnmE-dependent tRNA modification promotes biofilm development. (**A**) Pellicle biofilms grown in static cultures of biofilm-promoting medium (MSgg) or not–biofilm-promoting medium (MSGA) for 27 hours at 37°C. MSGA medium contains glucose and ammonia as carbon and nitrogen sources, respectively, instead of the glycerol and glutamate of MSgg. (**B**) Colony biofilms on MSgg agar plates grown at 30°C for 72 hours. Scale bar, 1 cm. (**C**) Colony biofilms of strains containing the *P_tapA_-sfGFP* reporter grown on MSgg agar plates [containing 0 or 1 mM isopropyl-β-d-thiogalactopyranoside (IPTG)] at 30°C and scanned for green fluorescent protein (GFP) fluorescence after 24, 48, or 72 hours. Expression of tRNA-binding–defective MnmE_C456S_ does not complement Δ*mnmE* biofilm phenotype. Scale bar, 1 cm. (**D**) Pellicle biofilms grown in static cultures of defined S7 + 20AA medium, with or without IPTG addition (0 or 1 mM IPTG) at 37°C for 16 hours. Expression of GTP-catalytically dead mutants of MnmE (MnmE_D338N_ or MnmE_G233A_) does not complement Δ*mnmE* biofilm phenotype. (**E**) Macroscopic image of colony biofilms grown on MSgg agar at 30°C for 72 hours. These colonies are the ones imaged for GFP fluorescence in (C). Scale bar, 1 cm. (**F**) Biofilm phenotypes of the Δ*rsmG* mutant compared to WT, Δ*mnmE*, and Δ*mnmG*. Top row: Macroscopic image of colony biofilms grown at 30°C for 72 hours on MSgg agar plates. Bottom row: Pellicle biofilms grown in S7 + 20AA static cultures for 19 hours at 37°C. Scale bar, 1 cm.

To quantitatively measure biofilm formation, we fused the well-established biofilm promoter *P_tapA_* to *sfGFP* to report activation of the *tapA-sipW-tasA* operon (encoding key components of the biofilm extracellular matrix) and thereby biofilm development in *B. subtilis* (*38*). Fluorescence imaging throughout 3 days of biofilm development on solid MSgg agar revealed that ∆*mnmE* and ∆*mnmG* mutants consistently exhibited ∼2-fold higher fluorescence than WT cells (*P* < 0.05) (Fig. 2C and fig. S3C). By day 3, *P_tapA_* induction was restricted to the colony edge in WT but sustained in the interior of both mutants (Fig. 2C), consistent with a hyperbiofilm phenotype. Confocal microscopic imaging shows that WT contains both wrinkled and nonwrinkled zones, while ∆*mnmE* appears uniformly wrinkled with elevated *P_tapA_-sfGFP* signal throughout, indicative of constitutive biofilm formation (fig. S3D).

The hyperbiofilm phenotype in ∆*mnmE* or ∆*mnmG* mutants—*P_tapA_-sfGFP* fluorescence levels and colony and pellicle morphology—is rescued by ectopically expressing WT *mnmE* or *mnmG*, respectively (Fig. 2, C to E, and fig. S3C). As expected, the tRNA-binding–defective variant MnmE_C456S_ (*33*) or catalytic mutants with impaired GTP hydrolysis activity (MnmE_G233A_ and MnmE_D338N_) failed to rescue the biofilm phenotype (Fig. 2, C to E).

*mnmE* and *mnmG* are coencoded with *rsmG* [encoding the G535 16*S* ribosomal RNA (rRNA) methylase] and *noc* (encoding the nucleoid occlusion factor) (fig. S3E), but the *rsmG* deletion mutant displays a WT biofilm phenotype (Fig. 2F). Together with the complementation, these data demonstrate that hyperbiofilm formation results from loss of MnmEG tRNA modification activity rather than secondary effects of operon disruption.

### Hyperbiofilm phenotype is specific to the loss of (cm)nm^5^(s^2^)U tRNA modification

*B. subtilis* is among the few bacteria with a well-characterized tRNA modification landscape (*42*). This allowed us to survey mutants lacking enzymes that modify the wobble base (position 34) or adjacent nucleotides known to influence codon decoding. We found that only Δ*mnmE* and Δ*mnmG* displayed hyperbiofilm phenotypes with increased pellicle wrinkling, while the other tRNA modification mutants resembled the WT pellicle biofilm (Fig. 3A). On plates, most mutants (*trmR*, *cspR*, *queA*, and *tgt*) resembled WT colony biofilm, while *miaA, queF*, and *truA* mutants showed minor deviations [e.g., absence of a smooth inner ring (*43*)]. None of the mutants exhibited increased biofilm seen in *mnmE* or *mnmG* mutants.

**Fig. 3.**
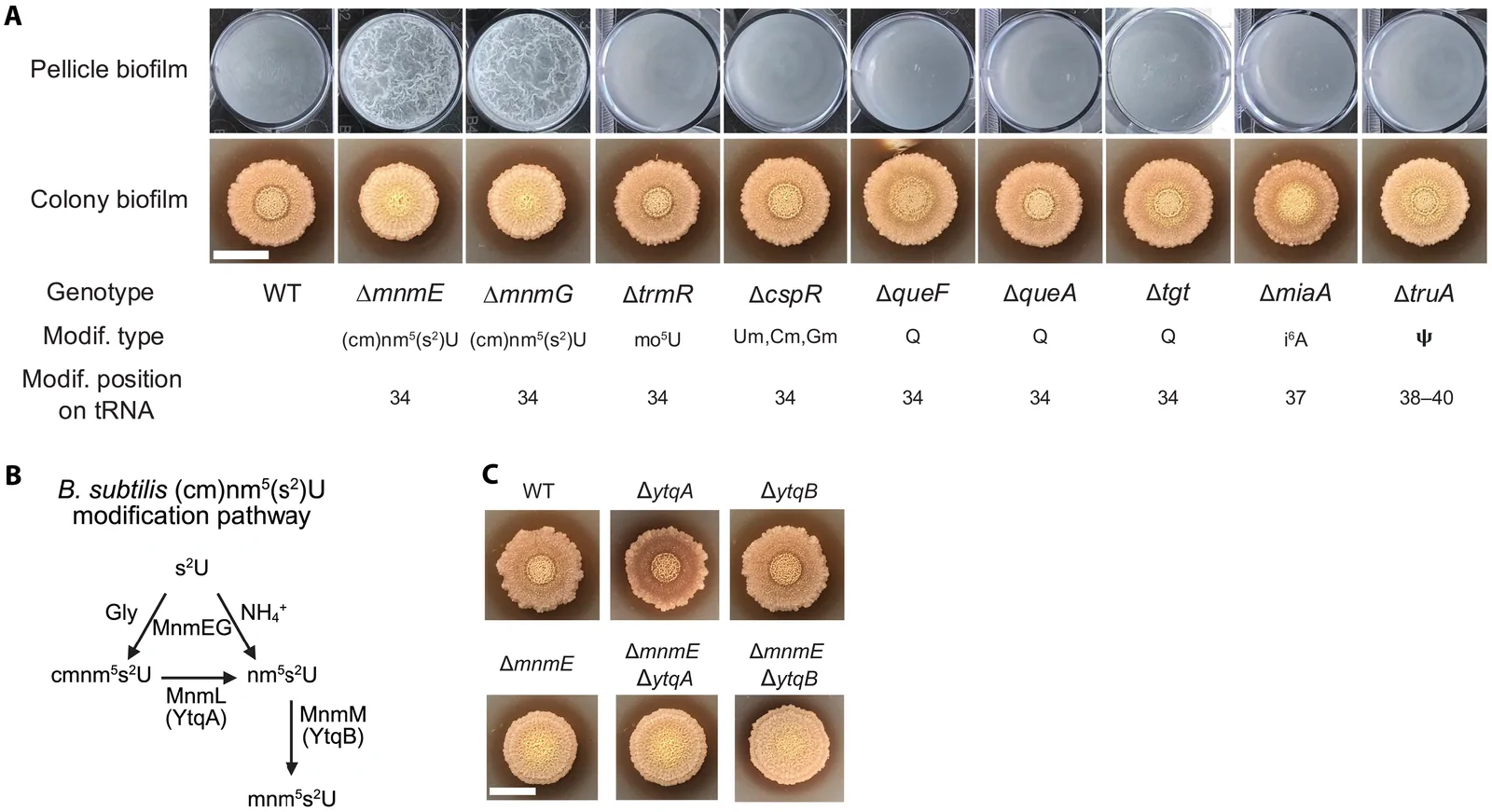
The hyperbiofilm phenotype is specific to loss of MnmEG. (**A**) Survey of biofilm phenotypes for tRNA modification mutants (anticodon loop region): Modifications and their position(s) are listed below the mutant genotype. Top row: Pellicle biofilms grown in S7 + 20AA static cultures for 19 hours at 37°C. Bottom row: Colony biofilms grown at 30°C for 72 hours on MSgg agar plates. Scale bar, 1 cm. WT, Δ*mnmE*, and Δ*mnmG* data are from the same experiment shown in Fig. 2F. Modif., modification. (**B**) Schematic of the (cm)nm^5^(s^2^)U tRNA modification pathway in *B. subtilis*, adapted from *Jaroch et al.* (*69*). (**C**) Epistatic study of *ytqA* (*mnmL*) and *ytqB* (*mnmM*) genes relative to *mnmE* in the context of colony biofilm formation. Macroscopic image of colony biofilms grown at 30°C for 72 hours on MSgg agar plates. Scale bar, 1 cm.

The wobble uridine tRNA modification pathway proceeds from a common precursor through MnmEG-dependent formation of (cm)nm^5^s^2^U, followed by further branch-point modifications by MnmL [yielding nm^5^(s^2^)U] or MnmM [mnm^5^(s^2^)U] (Fig. 3B). Deletion of either *mnmL* (*ytqA*) or *mnmM* (*ytqB*) resulted in WT biofilm morphology (Fig. 3C). Furthermore, combining each deletion with Δ*mnmE* retained the hyperbiofilm phenotype of ∆*mnmE* (Fig. 3C). These genetic relationships indicate that the hyperbiofilm phenotype arises specifically from loss of the (cm)nm^5^ modification itself, rather than from changes in the relative abundance of downstream wobble-uridine derivatives (cmnm^5^, nm^5^, or mnm^5^).

### The transcriptomics of the *mnmE* mutant reveal increased biofilm matrix gene expression and translational stress

To study how loss of (cm)nm^5^(s^2^) modification affects global gene expression, we compared the transcriptomes of ∆*mnmE* and WT strains by RNA sequencing (RNA-seq) under several conditions: exponentially growing, shaking cultures in LB; and static cultures in MSgg (biofilm-promoting) or MSGA (non–biofilm-promoting) medium at 8 and 24 hours postinoculation (Fig. 4, A and B) following established protocols for pellicle biofilm development (*40*). Across all conditions, *mnmE* emerged as the most strongly diminished gene in the ∆*mnmE* strain, confirming the deletion and indicating that *mnmE* is normally expressed under these growth regimes (Fig. 4, C to G). Notably, we observed consistent induction of the multidrug ATP-binding cassette (ABC) transporter–encoding genes *bmrC* and *bmrD* (*44*) in the mutant, an operon subjected to translation-dependent regulation and specifically activated under translation stress (Fig. 4C and fig. S4, B and C) (*45*, *46*). Beyond *mnmE*, *bmrC*, and *bmrD*, no other genes are significantly altered in LB cultures [|log_2_ fold change| (|log_2_FC|) > 0.58 and false discovery rate (FDR) < 0.05], indicating that MnmE has a minimal impact on global gene regulation under nutrient-rich growth conditions (table S5).

**Fig. 4.**
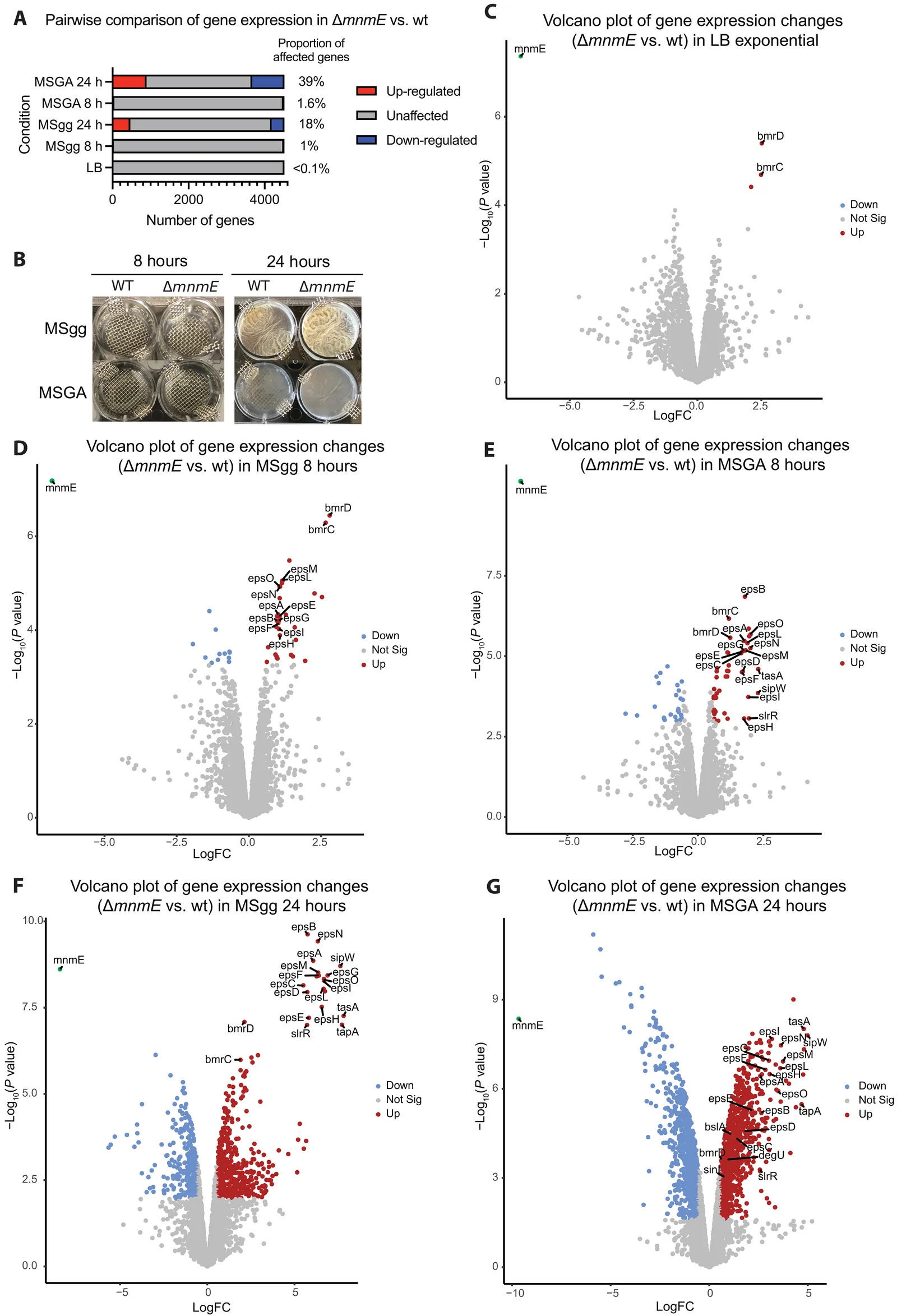
Transcriptomic analysis of the *mnmE* mutant reveals enhanced biofilm gene expression and signatures of translational stress. (**A**) Overview of differential gene expression between Δ*mnmE* and WT in the stand-alone RNA-seq study across growth conditions. h, hours. (**B**) Representative pictures of samples collected for transcriptomic studies: static cultures grown in MSgg or MSGA medium for 8 or 24 hours in the presence of metal mesh (*40*). Same experiment as fig. S3B. (**C** to **F**) Volcano plots of differential gene expression (Δ*mnmE* versus WT). Genes differentially expressed in Δ*mnmE* (by |log_2_FC| > 0.58 and FDR < 0.05) are colored in red for up-regulated and in blue for down-regulated genes. Biofilm genes and *bmrCD* are labeled. *mnmE* gene is indicated by a green dot. (C) is in LB exponential phase; (D) static cultures in MSgg medium after 8 hours; (E) static cultures in MSGA medium after 8 hours; (F) static cultures in MSgg medium after 24 hours; (**G**) static cultures in MSGA medium after 24 hours.

In static MSgg or MSGA cultures, WT cells remain planktonic 8 hours after inoculation (*40*), and differential expression analysis revealed that 1 to 1.6% of genes differ between ∆*mnmE* and WT cells (Fig. 4, A and B and D and E). Notably, biofilm matrix genes were among the most up-regulated in ∆*mnmE* in both media (Fig. 4, D and E; fig. S4, D and E; and table S5), including the *epsA-O* operon, encoding proteins required for synthesis of exopolysaccharide, a key component of the biofilm extracellular matrix, and the *tapA-sipW-tasA* operon, encoding the amyloid fiber protein TasA and the signal peptidase SipW, suggesting that ∆*mnmE* cells prematurely induce the extracellular matrix genes even in the absence of the full set of environmental cues that trigger biofilm formation. The ∆*mnmE* strain did not grow faster than WT and instead exhibited slightly reduced growth (fig. S4A), indicating that biofilm-gene induction in ∆*mnmE* is not due to early entrance into stationary phase.

At 24 hours, WT cells formed pellicle biofilms in MSgg but not in MSGA, while ∆*mnmE* formed pellicles in both media (Fig. 4B). Transcriptomic differences between ∆*mnmE* and WT cells were more pronounced, with 18% of genes differentially expressed in MSgg and 39% in MSGA, where WT and ∆*mnmE* adopt distinct lifestyles (Fig. 4, A and B). Notably, most matrix genes were strongly up-regulated in ∆*mnmE* (Fig. 4, F and G; fig. S4, F and G; and table S5). Collectively, these transcriptomic analyses reveal that lack of (cm)nm^5^(s^2^)U tRNA modification leads to signatures of translation stress and sustained activation of biofilm matrix genes.

### 5′-end assignment enables nucleotide resolution in *B. subtilis* ribosome profiling

To directly assess the impact of MnmE on translation, we performed ribosome profiling (Ribo-seq), a technique that provides a genome-wide snapshot of ribosome occupancy on mRNAs (*47*, *48*). Δ*mnmE* and WT cells were grown in MSgg, and samples were collected for parallel Ribo-seq and RNA-seq analyses. Because MnmE modifies the wobble uridine of specific tRNAs, its deletion is expected to impair decoding of certain codons. However, bacterial Ribo-seq is limited by the use of micrococcal nuclease (MNase), whose sequence bias generates ribosome footprints of heterogeneous lengths, reducing positional resolution compared with eukaryotic Ribo-seq, where ribonuclease (RNase) I produces more uniform ∼28–nucleotide (nt) fragments (*49*). In *E. coli*, assigning ribosome density to the 3′-end reads improves positional accuracy relative to center assignment and is now standard (*50*). By contrast, 3′-end strategy fails in *B. subtilis* Ribo-seq, where initiation and elongation signals remain blurred even with center-based or related alignments.

We systematically revisited end-assignment methods using metagene analysis of ribosome footprints aligned to start or stop codons (Fig. 5A and fig. S5A). 5′-end assignment produced sharp, discrete peaks consistently located 18 nt upstream of the start codon regardless of footprint length, whereas 3′-end peaks shifted linearly with fragment size (Fig. 5A). This pattern is the exact opposite of *E. coli*, and our pipeline reproduced the expected 3′-end precision on published *E. coli* datasets (fig. S5B) (*50*). We confirmed that 5′-end assignment provides the most accurate positional information in *B. subtilis* Ribo-seq by extending our analysis to a published dataset generated in LB medium (fig. S5C) (*51*). Analysis revealed consistent 5′-end assignment accuracy with a constant 18-nt distance between the 5′-end and start codon in *B. subtilis*, whereas distance derived from 3′-end assignment shifted proportionally with footprint size (fig. S5C). These results establish that 5′-end assignment is the most reliable approach for achieving nucleotide resolution in *B. subtilis* Ribo-seq experiments. On the basis of the ribosome initiation signal, the P-site is located 18 nt from the 5′ end of the read. We therefore applied the 5′-end offsets of 21, 18, and 15 nt to assign the A-, P-, and E-site positions, respectively (Fig. 5B).

**Fig. 5.**
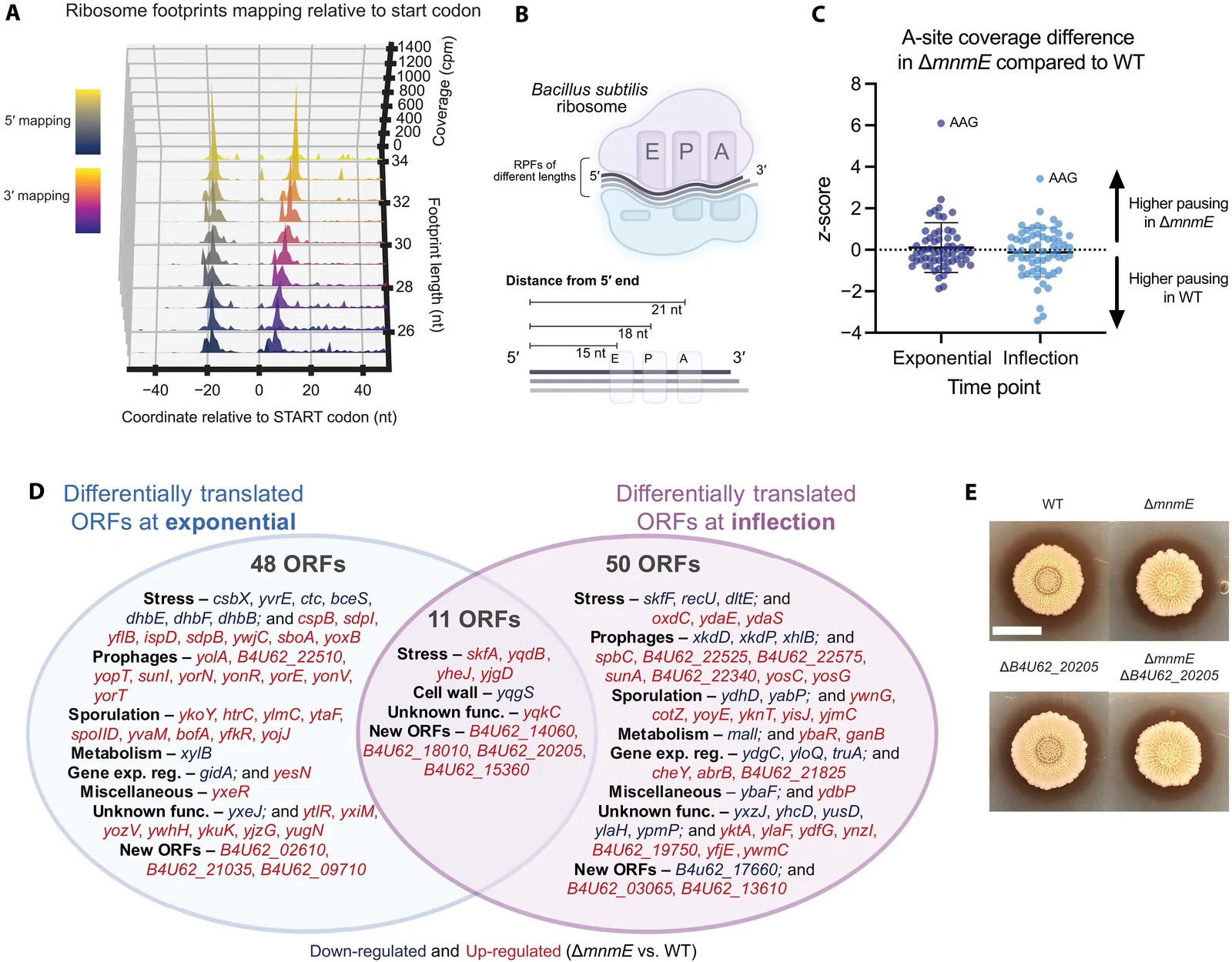
Optimized Ribo-seq reveals lysine AAG codon–specific ribosome pausing and differential ORF translation in Δ*mnmE*. (**A**) Average ribosome occupancy aligned at the start codon depending on footprint length. Reads from our WT t1-1 dataset were binned on the basis of lengths and assigned positions using the 5′ end of the reads (cold tone scale) or the 3′ end of the reads (warm tone scale). 5′-end assignment shows a discrete coverage peak 18 nt upstream of start codon regardless of footprint length, whereas 3′-end assignment peak position varies linearly with footprint length. Similar results were obtained with the other datasets of our study. (**B**) Schematic of the above-determined 5′ offsets to infer A-, P-, and E-site positions for the *B. subtilis* ribosome. (**C**) Genome-wide A-site codon coverage of elongating ribosomes: Plotted is *z*-score of coverage difference for each elongating codon in Δ*mnmE* compared to WT during exponential phase and at inflection point. Null *z*-score indicates no A-site coverage difference between Δ*mnmE* and WT; outlier codons are labeled. (**D**) Venn diagram of differentially regulated ORFs during exponential phase and at inflection point: Gene categories are indicated with corresponding ORFs listed in blue if down-regulated or red if up-regulated (in Δ*mnmE* versus WT). *yheJ* is *bmrB* synonym. (**E**) Epistatic study between the predicted small ORF *B4U62_20205* and *mnmE* in the context of colony biofilm formation. Macroscopic image of colony biofilms grown at 30°C for 72 hours on MSgg agar plates. Scale bar, 1 cm.

### Ribo-seq reveals increased pausing at lysine AAG codons in Δ*mnmE* and 109 differentially translated ORFs

Using the 5′-end assignment method described above, we performed high-resolution ribosome-pausing analysis in WT and Δ*mnnE* mutant. A-site codon was assigned for all Ribo-seq reads, ribosome occupancy was normalized to RNA-seq coverage, and codon-specific densities were averaged across all ORFs to quantify pausing at the 61 sense codons (fig. S6, A and B). Our analysis confirmed previous observations of ribosome pausing at serine and cysteine codons under biofilm conditions (*52*) and showed that this pausing is independent of MnmE (Fig. 5C and fig S6, A and B). To identify codons specifically affected by MnmE, we used *z*-scores to quantify pausing differences between Δ*mnmE* and WT and found that loss of *mnmE* significantly increased pausing at lysine AAG codons (Fig. 5C). Because all lysine codons (AAA and AAG) are decoded by tRNA^Lys(UUU)^, a substrate of MnmE (*13*, *42*), our findings suggest that (cm)nm^5^(s^2^) modification specifically promotes decoding of AAG, but not AAA, by tRNA^Lys(UUU)^.

Next, we identified ORFs whose translations are affected by MnmE, by calculating their translation efficiency (TE) (*53*), defined as the ratio of ribosome-protected fragment (RPF) coverage to transcript abundance. In total, 109 ORFs showed significant changes in TE between WT and mutant strains, either during exponential growth, at the inflection point, or at both stages (Fig. 5D and tables S6 and S7). These ORFs are linked to sporulation, biofilm, stress responses, or prophage activity (Fig. 5D). In contrast, housekeeping genes appeared largely unaffected by the loss of *mnmE*, suggesting that tRNA modification preferentially modulates stress-responsive pathways (*1*).

In addition to known genes, we also identified previously unannotated ORFs directly from ribosome profiling data using machine-learning tools implemented in the HRIBO workflow (table S6) (*54*). The prevalence of these putative ORFs under biofilm-promoting conditions is particularly notable, suggesting that this stress-induced state activates a layer of translation that is largely invisible under standard growth conditions. Consistent with this idea, many ORFs with altered TE in Δ*mnmE* are either previously unannotated or encode proteins of unknown function, underscoring that the translational response involves a distinct and underexplored set of coding elements beyond the canonical genome annotation.

### MnmE regulates AbrB expression via an LP-mediated translation control

We next examined the set of differentially translated ORFs to identify candidates that could plausibly drive the Δ*mnmE* hyperbiofilm phenotype. The LP BmrB (*yheJ*) showed increased TE in Δ*mnmE*, consistent with enhanced downstream transcription of *bmrCD* via antitermination (fig. S6D) (*45*). However, deletion of *bmrCD* had no effect on biofilm formation (fig. S6E), arguing that *bmrCD* is not responsible for the phenotype. Similarly, our newly identified putative ORF B4U62_20205, located between *pbpG* and *ywhD* and overlapping a predicted noncoding RNA (ncRNA) (nrc2768) (*55*), showed the strongest TE increase in Δ*mnmE* (log_2_FC = 1.59 to 3.20; fig. S6C), yet its deletion did not alter biofilm phenotypes (Fig. 5E). Thus, although both the *bmr* operon and B4U62_20205 are strongly MnmE responsive at the translational level, neither appears to contribute to the hyperbiofilm output.

Among differentially translated candidates, the global transition-state regulator AbrB (*56*) stood out as a compelling link between translation control and biofilm development. AbrB is a known repressor of biofilm genes and the *bmrBXCD* operon (*45*, *57*), placing it at an intersection where modest shifts in its expression can be amplified into large phenotypic consequences. AbrB TE is significantly altered at the growth inflection point (log_2_FC = 1.52). Both ribosome footprint values and mRNA levels were reduced in Δ*mnmE*, indicating an overall reduction in *abrB* output that would lead to higher biofilm formation (table S6).

We closely examined our Ribo-seq data to assess the effect of Δ*mnmE* on *abrB* translation. Ribo-seq over *abrB* ORF showed no evidence of elongation defects or increased pausing (Fig. 6A). This is consistent with the strong bias of lysine codon bias in AbrB: All 11 lysines are encoded by AAA and none by AAG, predicting minimal sensitivity to loss of MnmE-dependent modification (fig. S6F). 5′-end assignment shows that *abrB* translation initiates from the second annotated ATG, consistent with prior N-terminal sequencing (*58*). Against this backdrop, we identified a reproducible Δ*mnmE-*specific ribosome-footprint peak in the *abrB* 5′ untranslated region (5′UTR; Fig. 6A). This peak maps to an upstream ATG predicted to initiate a short out-of-frame LP. The seven–amino-acid LP (MTKKNII) contains tandem lysine codons (AAG-AAA), including a MnmE-sensitive AAG, thereby placing the Δ*mnmE* translation signature upstream of the *abrB* coding sequence. Given continuous transcription from the *abrB* P2 promoter, these data suggest that LP translation may represent an additional regulatory layer controlling downstream *abrB* expression.

**Fig. 6.**
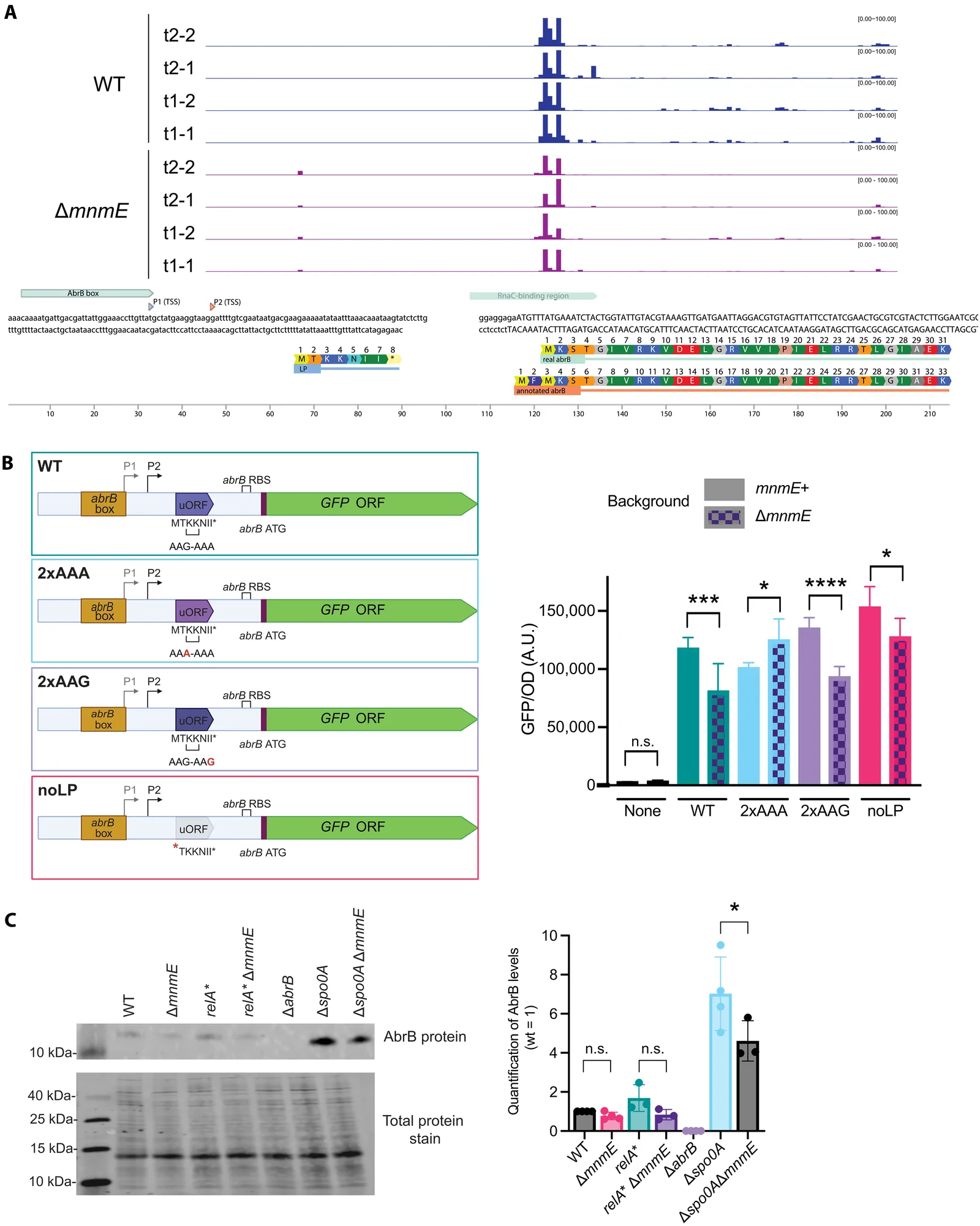
MnmE enhances AbrB expression through LP-mediated control of translation. (**A**) P-site ribosome coverage for each sample aligned to NCIB3610 genome: Integrative Genomics Viewer (IGV) view of the 5′UTR region of *abrB*. DNA sequence is annotated with P1 and P2 transcription start sites (TSS), AbrB DNA binding region (AbrB box), RnaC binding region, the putative LP ORF, and *abrB* ORF (annotated version and actual ORF). t1 are exponential phase, and t2 are inflection point samples. (**B**) Left: Schematic of the *P_abrB_-GFP* reporter constructs. Right: GFP fluorescence signal normalized per optical density (OD) measured at the inflection point of growth in S7 + 20AA medium for strains with different version of the *P_abrB_-GFP* reporter or no reporter control in WT (plain) or Δ*mnmE* (checkered) background. Normalized signal values were analyzed by two-way ANOVA with reporter and background as factors, followed by Šídák’s multiple-comparison test to assess background effects for each reporter. Asterisks indicate adjusted *P* value (*P < 0.05, ***P < 0.001, and ****P < 0.0001). Error bars indicate SD across replicates. A.U., arbitrary units. (**C**) Western blot analysis of AbrB protein levels. AbrB protein levels are normalized for loading using total protein stain. The bar graph shows relative AbrB protein levels (WT = 1), and error bars indicate SD across replicates. Statistical data analysis was done using paired *t* test; asterisk indicates *P* value (*P < 0.05).

To test the effect of LP and MnmE on *abrB* expression, we constructed green fluorescent protein (GFP: *GFPmut2^A206K^*) reporters driven by the native *abrB* promoter and containing the entire 5′UTR (Fig. 6B). Reporter expression was reduced by ∼31% in Δ*mnmE* relative to WT cells (Fig. 6B), consistent with the decreased *abrB* translational output observed in Ribo-seq (Fig. 6A). Substituting the LP start codon (ATG) with a stop codon (TAG) increased GFP expression, indicating that the LP negatively regulates *abrB* expression. Recoding of the AAG-AAA codons further revealed sequence dependence; the tandem-AAG variant (2xAAG) preserved Δ*mnmE* sensitivity (31% decrease), whereas the tandem-AAA variant (2xAAA) reversed the effect, resulting in a 20% higher GFP expression in Δ*mnmE*, suggesting that absence of AAG codons eliminates the Δ*mnmE*-dependent repression.

Last, we assessed AbrB protein levels by Western blot using native AbrB antibodies. AbrB levels were modestly lower in Δ*mnmE* than in WT cells, although the difference did not reach statistical significance, because Spo0A-dependent repression limits *abrB* expression and results in weak signals. To improve detection, we eliminated Spo0A-mediated transcriptional repression. Under these conditions, AbrB levels increased substantially, enabling a clearer comparison, and the Δ*mnmE* Δ*spo0A* strain exhibited significantly lower AbrB protein levels than the Δ*spo0A* single mutant (Fig. 6C). Thus, AbrB is regulated at both the transcriptional and translational levels, with Spo0A repressing *abrB* transcription and MnmE influencing AbrB production through the upstream LP.

### Genetic epistasis defines a (p)ppGpp-MnmE-AbrB pathway regulating biofilm formation

AbrB is a well-established repressor of biofilm gene expression (*57*–*59*), and thus, reduced AbrB output in Δ*mnmE* is consistent with the hyperbiofilm phenotype of this strain. Nevertheless, whether MnmE acts downstream of (p)ppGpp in biofilm regulation and whether AbrB is the principal target through which MnmE controls biofilm formation remain unresolved. To address these questions, we performed epistasis analyses using key regulators of the *B. subtilis* biofilm pathway. In this pathway, nutrient and quorum-sensing inputs converge on the master regulator Spo0A, which promotes biofilm formation by antagonizing the activities of the two principal repressors, AbrB and SinR (Fig. 7A) (*37*, *57*). Consistent with prior studies, deletion of either *abrB* or *sinR* resulted in hyperbiofilm formation, whereas deletion of *spo0A*, *sinI*, *remA*, or *remB* abolished biofilm development (Fig. 7B). Neither Δ*mnmE* nor Δ*mnmG* restored biofilm formation in these biofilm-deficient backgrounds, indicating that MnmEG function cannot bypass core biofilm activation pathways controlled by Spo0A, SinI, and RemAB (Fig. 7B). Suppressor analyses further refined pathway order. Loss-of-biofilm suppressors isolated from Δ*mnmE* or Δ*mnmG* mapped to *remAB* or to *eps* matrix biosynthesis genes (Fig. 7C), placing MnmEG upstream of matrix production and consistent with a role in biofilm regulation rather than matrix assembly itself. Together, these results indicate that MnmEG functions upstream of matrix production and requires the canonical biofilm regulatory machinery. However, the epistasis analyses did not identify a specific node within the established biofilm regulatory network through which MnmEG exerts its effects.

**Fig. 7.**
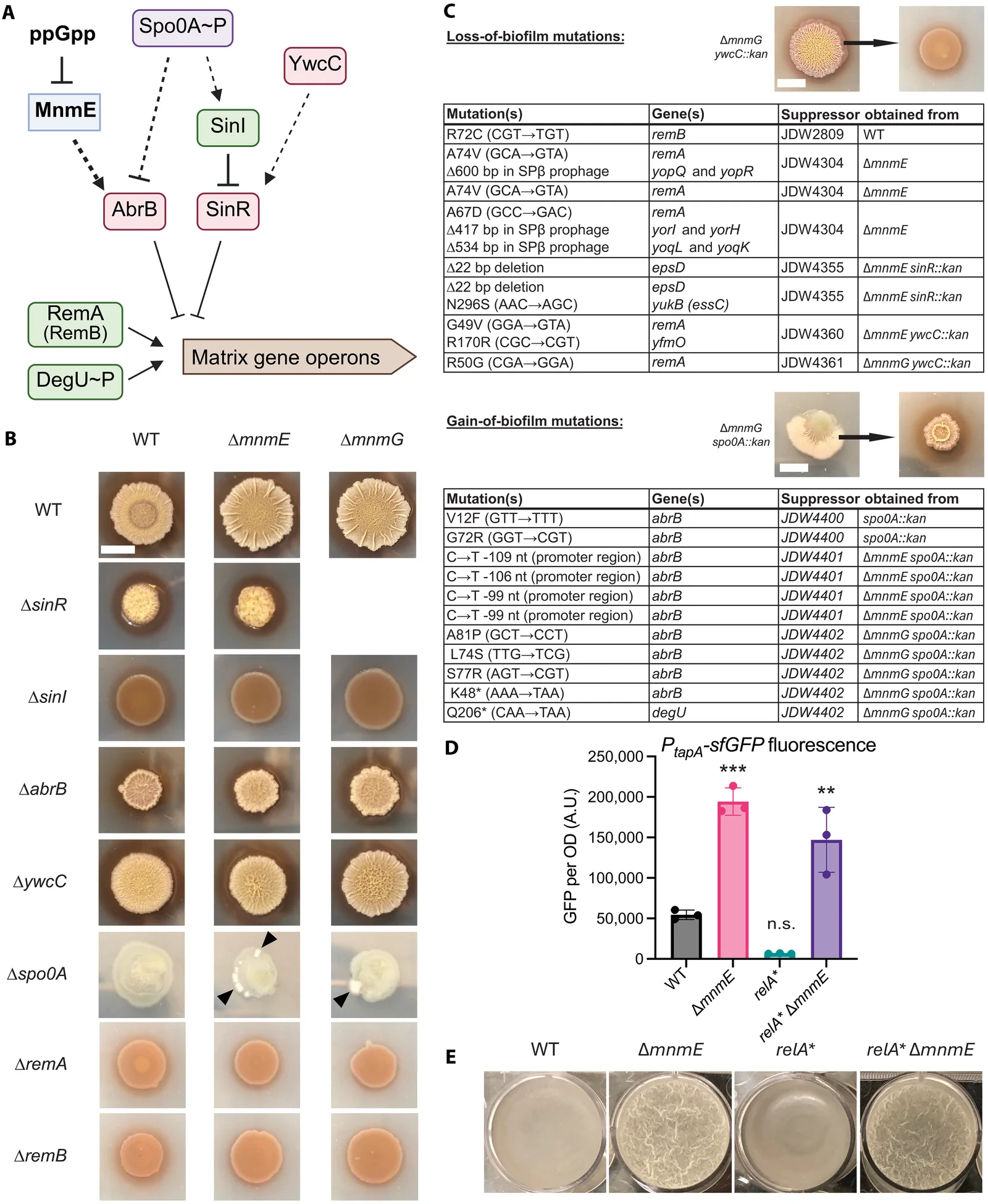
A (p)ppGpp-MnmE-AbrB regulatory axis controlling biofilm formation. (**A**) Simplified schematic of the regulatory pathways controlling expression of the biofilm matrix gene operons and consequences of the ppGpp-mediated regulation of MnmE activity. Schematic focuses on the direct transcription regulators (rounded boxes) of the three biofilm matrix gene operons and is adapted from Cairns *et al.* (*38*)*.* The direct inhibition of MnmE activity by ppGpp decreases levels of the biofilm repressor AbrB because MnmE activity normally acts positively on AbrB translation (dotted arrow) through its LP. (**B**) Checkerboard analysis of biofilm-pathway mutants combined with Δ*mnmE* or Δ*mnmG* during colony biofilm formation. Macroscopic images of colony biofilms grown at 30°C for 72 hours on MSgg agar plates. Black arrows in the figure point the appearance of gain-of-biofilm suppressor within the course of the colony biofilm assay. Scale bar, 1 cm. (**C**) List of loss-of-biofilm and gain-of-biofilm mutations isolated from spontaneous suppressors using colony biofilm assays. One macroscopic image showing appearance of the suppressor of biofilm mutant and confirmation of the phenotype of the isolated suppressor is shown as an example for each type. Scale bar, 1 cm. (**D**) Bar graph of *P_tapA_-sfGFP* fluorescence to OD ratio at 24-hour time point for strains grown in S7 + 20AA medium (full data curve in fig. S7A). Error bars indicate SD across triplicates. Statistical significance relative to the WT control strain was assessed using one-way ANOVA with Dunnett correction for multiple comparison; adjusted *P* values (**P for <0.01, ***P for <0.001, and n.s., not significant). (**E**) Pellicle biofilms grown in static S7 + 20AA cultures for 20 hours and 30 min at 37°C. The ppGpp-defective relA* strain grows but does not form any pellicle.

In parallel, we isolated spontaneous suppressors that restored biofilm formation in ∆*spo0A* strains, both in the presence and absence of *mnmE/G*. Notably, all but one of these suppressors mapped to *abrB* (Fig. 7C), with mutations either in the *abrB* promoter or causing missense changes or truncations in the AbrB protein, consistent with loss-of-function alleles that reduce AbrB expression or activity (*58*). These findings confirm AbrB as the major barrier to biofilm formation in the absence of Spo0A. Our Western blot showed that loss of MnmEG reduces AbrB expression independently of Spo0A. Accordingly, rough biofilm-like sectors emerged rapidly in Δ*spo0A*Δ*mnmE* and Δ*spo0A*Δ*mnmG* colonies, whereas suppressors appeared only after prolonged incubation in the Δ*spo0A* single mutant (Fig. 7, B and C, arrows). We propose that loss of MnmEG partially lowers AbrB output, weakening repression of biofilm genes and sensitizing cells to secondary *abrB* loss-of-function mutations. This model explains the accelerated emergence of biofilm-forming suppressors in the double mutants and supports AbrB as a major downstream target of MnmEG-dependent biofilm regulation.

We next asked whether (p)ppGpp regulates biofilm formation through this MnmE-AbrB axis. To test this, we used a relA* mutant carrying a well-characterized D264G substitution that abolishes RelA-dependent (p)ppGpp synthesis (*60*). The relA* mutant, although able to grow in amino acid–supplemented medium, failed to form biofilms (Fig. 7, D and E, and fig. S7, A to C), confirming that (p)ppGpp is required for biofilm development. Deletion of *mnmE* restored biofilm formation in the relA* background (Fig. 7, D and E). Consistent with this phenotypic rescue, expression of a *tapA* promoter–driven sfGFP reporter was abolished in relA* but restored in relA* Δ*mnmE*, indicating that loss of *mnmE* compensates for the absence of (p)ppGpp signaling during biofilm formation.

Together with our translational analyses of *abrB*, these genetic data support a model in which stress-induced (p)ppGpp signaling promotes biofilm formation by counteracting MnmE-dependent tRNA modification (Fig. 7A). Reduced MnmE activity lowers *abrB* expression, relieving AbrB-mediated repression of biofilm genes. As a result, loss of *mnmE* phenocopies a key output of (p)ppGpp signaling and restores biofilm formation in a *relA* mutant.

## DISCUSSION

Bacteria adapt to fluctuating environments by transitioning between lifestyles such as biofilm formation, sporulation, and motility (*38*, *61*, *62*). Although the regulatory networks governing these transitions have been extensively studied at the transcriptional level, the role of translational control remains less understood. Here, we uncover a tRNA modification–based regulatory mechanism linking stress signaling to biofilm development. We show that the (cm)nm^5^(s^2^)U tRNA–modifying GTPase MnmE is inhibited by the alarmone ppGpp. Loss of MnmE causes a hyperbiofilm phenotype and restores biofilm formation in a ppGpp-compromised background, indicating that ppGpp promotes biofilm development, at least in part, by inhibiting MnmE-dependent tRNA modification. High-resolution ribosome profiling revealed increased pausing at AAG codons in Δ*mnmE* cells and altered translation of stress-response genes and previously unannotated small ORFs. Notably, one such ORF encodes an LP within the *abrB* 5′UTR that modulates translation of this key biofilm repressor. Through this mechanism, MnmE promotes AbrB expression and planktonic growth, whereas ppGpp-mediated inhibition of MnmEG lowers AbrB levels and enables biofilm formation. Together, our findings establish regulated tRNA modification as a translational control mechanism linking stress signaling to bacterial adaptive behavior.

### Direct inhibition of a tRNA-modifying enzyme by ppGpp links stress signaling to translational regulation

The tRNA epitranscriptome, the modification profile of cellular tRNAs, is increasingly recognized as dynamic and responsive to environmental cues (*4*, *22*, *63*). However, whether these changes arise through dedicated regulatory pathways or indirectly from environmental perturbations has remained unclear. Variation in tRNA modifications has been mostly attributed to altered expression of modification enzymes or changes in the availability of their specific precursors and cofactors (*64*). For example, deprivation of folate-derived one-carbon donors reduces τm^5^U tRNA formation by MnmEG orthologs in mitochondria (*65*). Our finding suggests an additional mechanism by which stress signaling can influence tRNA-dependent translation. We showed that the alarmone ppGpp directly inhibits the tRNA-modifying GTPase MnmE. With an IC_50_ of ∼60 μM, MnmE is sensitive to physiologically relevant ppGpp concentrations, which rise from ∼10 μM during exponential growth to 1 mM during starvation in *B. subtilis* (*34*). The interaction between MnmE and ppGpp is conserved in *B. subtilis*, *B. anthracis*, and *E. coli* (*32*, *66*), suggesting that regulation of MnmE by ppGpp may be widespread among bacteria. In *E. coli*, oxidative and acid stress alter MnmEG-mediated tRNA modification levels (*22*). It will be important to determine whether these effects are mediated, at least in part, by ppGpp accumulation (*23*).

Our findings identify MnmE as a direct target through which ppGpp can influence the tRNA modification landscape and translational output. As a GTPase, MnmE is well positioned to function as a sensor of cellular guanine nucleotide status, with ppGpp regulating its activity both directly, by competing with GTP for binding, and indirectly, by inhibiting guanylate kinase, a central enzyme in GTP biosynthesis (*32*). Although GDP and ppGpp exhibit similar inhibitory activities toward MnmE in vitro (Fig. 1E), their physiological roles are likely distinct. Previous metabolomic studies quantified nucleotide pools during amino acid starvation, a condition that induces (p)ppGpp accumulation (*31*, *67*). Under nutrient-rich conditions, GDP is present at ∼0.2 mM, whereas after 30 min of amino acid starvation, GDP decreases to ∼38 μM. This decrease is consistent with ppGpp-mediated inhibition of guanylate kinase, which lowers both GDP and GTP pools; however, GTP remains more abundant than GDP under both nutrient-rich and starvation conditions. Thus, despite its inhibitory activity in vitro, GDP is unlikely to strongly suppress MnmE in vivo because GTP remains the predominant competitor for nucleotide binding. In contrast, starvation drives ppGpp accumulation to levels that rival or exceed GTP, favoring ppGpp occupancy of MnmE and inhibition of its activity. Together, these observations suggest that MnmE functions as a metabolic sensor that links guanine nucleotide homeostasis to tRNA modification and translational output, allowing nutrient stress to be translated into selective changes in gene expression. Whether physiological fluctuations in nucleotide pools quantitatively alter MnmE activity and tRNA modification levels remains an important question for future study.

Notably, this model differs from the prevailing view that tRNA modifications primarily function to support stress adaptation (*3*). Instead, our results suggest that regulated inhibition of specific tRNA modification can itself contribute to adaptive responses. In the case of biofilm formation, reduced MnmE activity promotes a translational program that facilitates the transition from planktonic growth to a multicellular lifestyle.

### The broadly conserved wobble-uridine modification enzyme MnmEG suppresses AAG codon pausing

To define the translational consequences of (cm)nm^5^(s^2^)U hypomodification in vivo, we established a 5′-end–based Ribo-seq read-assignment method optimized for *B. subtilis*, enabling single-nucleotide resolution despite low coverage under biofilm growth conditions and the intrinsic blurring of bacterial ribosome footprints. This revealed a pronounced increase in ribosome pausing at lysine AAG codons upon loss of MnmE (Fig. 5C). Because both AAA and AAG lysine codons are decoded by tRNA^Lys-UUU^ in *B. subtilis*, these data indicate that wobble-uridine (cm)nm^5^(s^2^) modification strongly enhances pairing with G-ending codons (*68*). Similar lysine- and leucine-codon–specific ribosome pausing has been observed upon loss of MnmE orthologs activity in human mitochondria, highlighting the conserved role of U_34_ modification in decoding G-ending codons (*65*).

While our analyses identify tRNA^Lys-UUU^ as a primary functional substrate of MnmE under conditions tested, we do not exclude impact of additional MnmE substrates (*69*). Other tRNAs modified by MnmE may have weaker or more context-dependent effects that fall below detection thresholds; their impact might also vary across organisms with distinct codon usage and translational architectures (*7*). Alternative outcomes of U_34_ hypomodification have been reported in other bacteria, such as ribosome drop-off without overt codon-specific pausing in *Pseudomonas aeruginosa mnmG* mutants (*2*). Despite these differences, a common theme across bacterial systems is that U_34_ tRNA modification preferentially influences genes involved in adaptive processes (biofilm, motility, virulence, and stress responses) rather than constitutive housekeeping functions. This selective sensitivity positions MnmEG-mediated tRNA modification as a modulatory layer that fine-tunes translational programs to support physiological adaptation (*1*, *2*, *18*).

### MnmE-dependent tRNA modification regulates upstream open reading frames (uORFs) and AbrB expression

Upstream open reading frames (uORFs) in 5′UTRs are widespread bacterial regulatory elements that modulate downstream gene expression through translational coupling, attenuation, and ribosome stalling (*70*). Our findings suggest that tRNA modification represents a previously unappreciated layer of posttranscriptional control acting through uORFs. We found evidence that translation of multiple putative uORFs is affected by MnmE-mediated U_34_ modification. In the *bmrBCD* operon, the LP *bmrB* controls the multidrug transporter *bmrCD* (*44*) through translation-dependent attenuation. Δ*mnmE* causes increased ribosome coverage of *bmrB* and strongly induced *bmrCD* expression (*45*), which remained up-regulated even during exponential growth in LB medium (Fig. 4 and fig. S4). Because *bmrCD* induction is a hallmark of translational stress triggered by ribosome-targeting antibiotics or accumulation of immature tRNAs (*45*, *46*, *71*), these findings suggest that loss of MnmE creates a chronic translational stress state even under otherwise optimal growth conditions.

We also uncovered a putative uORF encoding an LP in the *abrB* 5′UTR whose translation is sensitive to (cm)nm^5^(s^2^)U-dependent decoding of AAG codons. Loss of U_34_ modification increased ribosome coverage in the LP and reduced AbrB protein levels. AbrB is a conserved transition-state regulator that controls competence, sporulation, and biofilm formation in *Bacillus* species (*58*), and its expression is already subject to multiple layers of regulation, including autoregulation, Spo0A-dependent transcriptional control, and RnaC-mediated inhibition of translation initiation (*58*, *72*). Our findings suggest an additional regulatory layer in which ppGpp-inhibited tRNA modification alters LP translation to modulate AbrB expression during stress.

We propose that MnmE-dependent modification regulates uORF translation, thereby modulating downstream gene expression with broad physiological consequences. In the case of *abrB*, this mechanism enables posttranscriptional stress adaptation that influences biofilm formation and likely extends to other members of the AbrB regulon, including the ribosome hibernation factor, consistent with recent reports of (p)ppGpp-dependent AbrB reduction (*73*). This mode of regulation parallels examples in other systems where transcription regulators respond to tRNA wobble-base modification status. In *Mycobacterium bovis BCG*, codon-biased translation of the hypoxic master regulator DosR depends on elevated wobble-base modification during hypoxia (*74*). By contrast, our findings suggest an inverse regulatory logic: Inhibition of wobble-base modification alters AbrB translation via its uORF, promoting adaptive responses.

### A regulatory pathway of biofilm and multicellularity via inhibition of tRNA wobble-uridine modification

Biofilm represents the predominant lifestyle of most bacteria, offering resilience in harsh environment and immunity in clinical settings. *B. subtilis* NCIB3610 is an archetype for Gram-positive biofilm, and decades of research have uncovered the complex regulatory cascade controlling the costly synthesis of its biofilm components (*38*, *40*, *59*). However, the molecular mechanisms that transmit environmental cues into regulation of the biofilm pathway are poorly understood, especially how a community of isogenic cells can adopt different phenotypes within the biofilm (*37*, *75*, *76*).

Our work uncovers a mechanistic role for the alarmone ppGpp in tuning tRNA modification levels to influence developmental decision. Although several tRNA modifications have been implicated in biofilm development (*77*, *78*), only loss of the (cm)nm^5^(s^2^)U modification produced a hyperbiofilm phenotype, as observed in ∆*mnmE* strains. Notably, ∆*mnmE* colonies sustain biofilm-gene expression at the colony center even after 3 days of growth (Fig. 2C), resulting in more homogenous biofilms that lack the characteristic ring of death in WT biofilms (Figs. 2 and 3) (*79*, *80*). In addition, *mnmE* deletion restores biofilm formation in a *relA* mutant. Together, these findings suggest that ppGpp-regulated U_34_ modification contributes to the spatiotemporal control of biofilm development, placing the ppGpp-MnmE-AbrB regulatory network at the core of environmental sensing and community organization in *B. subtilis*.

tRNA modifications occur at multiple positions across the tRNA molecule and that many tRNA modification enzymes are housekeeping factors rather than regulated signaling nodes (*6*, *7*). One example is TrmD, an essential enzyme that catalyzes m^1^G37 formation and is essential for translational fidelity, as this modification prevents frameshifting during decoding (*81*). TrmD enzyme is blocked by nonphysiological 3′,5′-cyclic adenosine monophosphate (cAMP) in *Staphylococcus aureus*, whereas TrmD orthologs from organisms that naturally produce this nucleotide have evolved to discriminate against this inhibition (*82*). In contrast, MnmE is nonessential and controlled by ppGpp, allowing stress signals to reprogram translation during adverse conditions. Thus, while TrmD primarily safeguards baseline tRNA integrity and decoding accuracy, MnmE links nucleotide signaling to adaptive translational remodeling.

The tRNA wobble position (U_34_) may represent an ancient regulatory hotspot, as modification at this site can directly alter decoding preferences and reshape translation in response to environmental or developmental cues. Consistent with this idea, hypomodification of tRNA^Lys-UUU^ at U_34_ has been linked to oligodendrocyte differentiation in mammals (*83*). Together, our findings provide a molecular basis for developmental differentiation in *Bacillus* and highlight a broader role for the tRNA epitranscriptome in coordinating complex, multicellular behaviors across domains of life.

## MATERIALS AND METHODS

### Bacterial strains

Bacterial strains used in this work are listed in table S1. All *B. subtilis* mutant strains are derivatives of NCIB3610 strain *comI*^Q12L^ and retain the mega plasmid pBS32 (*84*). LB [yeast extract (5 g/liter), NaCl (10 g/liter), and tryptone (10 g/liter)] and LB agar [LB + agar (15 g/liter)] were used for cloning and maintenance of strains. For selection in *B. subtilis*, media were supplemented with the following antibiotics when required: spectinomycin (100 μg/ml), chloramphenicol (5 μg/ml), and kanamycin (10 μg/ml). Carbenicillin (100 μg/ml) was used for selection in *E. coli*. Plasmids used in this study are listed in table S2, while primers and synthetic gene fragments used for cloning are listed in table S3.

*B. subtilis* deletion mutants were constructed by homologous recombination with polymerase chain reaction (PCR) products from genomic DNA (gDNA) of the *B. subtilis* knockout collection (obtained from BGSC) (*85*) and verified by sequencing, unless otherwise indicated. The lox-site–flanked kanamycin resistance cassette was removed using pDR244-cre, followed by screening for restored kanamycin sensitivity.

The Δ*B4U62_20205* marker-less deletion was constructed by CRISPR-Cas9 editing method (*86*): The CRISPR editing plasmid pJW893 was assembled using the Golden Gate method (New England Biolabs) using pPB41 backbone, a guide RNA template, and a repair template (Twist Bioscience). The pPB41 backbone was amplified by PCR (with oJW2775/oJW2821) as previously described (*31*). The Δ*B4U62_20205* repair template is a synthetic gene fragment (Twist Bioscience) containing 500–base pair (bp) regions upstream and downstream of predicted *B4U62_20205* ORF. For the small predicted ORF *B4U62_20205* coordinates, we used the largest prediction HRIBO (*54*) made from our Ribo-seq data (see table S6), i.e., between coordinates 3,851,875 to 3,852,143 on the NCIB3610 chromosome (CP020102.1). The guide RNA template was made from annealing oJW4999 with oJW5000. CRISPR editing plasmids were used to edit *B. subtilis* genomes following previously published method (*31*, *86*). The synthetase-inactivated relA*(*D264G*) mutants were also obtained using this published CRISPR editing method (*31*, *86*). All strain-editing and plasmid-making details can be found in table S1 and fig. S2.

The fluorescent reporter for the induction of biofilm genes (*P_tapA_-sfGFP*), integrated on the chromosome at the *ycgO* locus, was obtained from O. Venturelli’s laboratory. The reporter for *abrB* expression *P_abrB_*(*wt*)*-GFPmut2^A206K^* and its 5′UTR point mutation variants are cloned in a plasmid derived from pDR110 (D. Rudner) for chromosomal integration at the *amyE* locus. Because AbrB is known to bind its own promoter and regulate its own gene expression (*58*), all strains carrying the *P_abrB_* fluorescent reporters have the *abrB* ORF deleted (*abrB::kan*) so the MnmE effect on *abrB* expression is studied independently of the AbrB-mediated transcriptional feedback.

For planktonic growth assays, *B. subtilis* strains were grown in LB or S7-defined medium (*34*) with 20 amino acids [50 mM Mops, (NH_4_)_2_SO_4_ (1.32 g/liter), (NH_4_)_2_SO_4_ (0.68 g/liter), 0.1% glutamate, 1% glucose, 2 mM MgCl_2_, 0.7 mM CaCl_2_, 50 μM MnCl_2_, 1 μM ZnCl_2_, thiamine-HCl (1 μg/ml), 5 μM FeCl_3_, alanine (50 μg/ml), arginine (50 μg/ml), asparagine (50 μg/ml), glutamine (50 μg/ml), histidine (50 μg/ml), lysine (50 μg/ml), proline (50 μg/ml), serine (50 μg/ml), threonine (50 μg/ml), glycine (50 μg/ml), isoleucine (50 μg/ml), leucine (50 μg/ml), methionine (50 μg/ml), valine (50 μg/ml), phenylalanine (50 μg/ml), aspartic acid (500 μg/ml), glutamic acid (500 μg/ml), tryptophan (20 μg/ml), tyrosine (20 μg/ml), and cysteine (40 μg/ml)]. Cells were harvested from young, overnight LB agar plates (<12 hours), backdiluted into fresh medium at optical density at 600 nm (OD_600_) ∼ 0.005, and grown at 37°C with vigorous shaking at 250 rpm.

### Protein purification

For biochemical assays, *B. subtilis mnmE* (*trmE*) and *mnmG* (*gidA*) coding sequences were cloned into the pLIC-trPC-HA vector (pJW269) using the ligation independent cloning (LIC) protocol (*87*, *88*). For the truncation protein MnmE–G-domain, a portion of *mnmE* sequence encoding G221 to Y380 was used for cloning, with added start and stop codons. The pLIC-trPC-HA plasmid encodes a 6× histidine (6xHis) tag and a tobacco etch virus (TEV) protease recognition site upstream of the ORF. For MnmE variants, amino acid substitutions were performed with a megaprimer site-directed mutagenesis protocol (*89*).

Recombinant plasmids were transformed into *E. coli* BL21 and grown at 30°C with shaking at 225 rpm in LB-M9 [49 mM Na_2_HPO_4_, 15 mM KH_2_PO_4_, 8.6 mM NaCl, 19 mM NH_4_Cl, 11 mM glucose, 3.7 mM sodium succinate, tryptone (10 g/liter), and yeast extract (5 g/liter)] supplemented with carbenicillin (100 μg/ml; RPI). A seed culture (grown to mid-log phase) was diluted 1:50 to inoculate the batch culture. Protein synthesis was induced at OD_600_ ≈ 0.8 with 1 mM isopropyl-β-d-thiogalactopyranoside (IPTG) (RPI) for 4 hours. Cells were pelleted and stored at −80°C until purification.

For the full-length MnmE: Cells were resuspended in lysis buffer [50 mM tris-HCl, 500 mM NaCl, 5% glycerol, and 10 mM imidazole (pH 8)] and lysed using a French press. The lysate was centrifuged to obtain the supernatant, which was then filtered through 0.45-μm filters (Nalgene). The sample was loaded on a HisTrap FF column (Cytiva) connected to an ÄKTA Pure FPLC (GE Healthcare), washed with 10 column volumes of lysis buffer supplemented with 3% elution buffer [50 mM tris-HCl, 500 mM NaCl, 5% glycerol, and 500 mM imidazole (pH 8)], and then eluted with a gradient of increasing elution buffer; and recombinant protein fractions were cleaved by TEV protease (equivalent of 1 mg of TEV for 1 liter of cells) in dialysis buffer I [50 mM tris (pH 8.0), 150 mM NaCl and 5% glycerol, and 1 mM dithiothreitol (DTT) and 0.5 mM EDTA]. Protein was further dialyzed into dialysis buffer II [50 mM tris (pH 8.0), 150 mM NaCl, and 5% glycerol] before reverse purification using HisTrap FF column (Cytiva) equilibrated with dialysis buffer II. Flow-through fractions containing the cleaved protein were concentrated five times using an Amicon Ultra-15 10 kDa MWCO centrifugal filter device (Millipore) before loading on a preparative size exclusion column HiPrep Sephacryl S-200 HR 26/60 (Cytiva) equilibrated in 50 mM tris (pH 7.5), 100 mM NaCl, 5 mM MgCl_2_, and 5 mM DTT buffer. Fractions containing the protein were concentrated using an Amicon Ultra-15 10 kDa MWCO centrifugal filter device (Millipore), aliquoted and flash frozen in liquid nitrogen before storage at −80°C. Protein purity was determined using SDS–polyacrylamide gel electrophoresis, and protein concentrations were measured using the Bradford assay (Bio-Rad).

For MnmG, we attempted a similar purification protocol as MnmE; however, the TEV tag removal was unsuccessful, so 6xHis-MnmG was purified using HisTrap FF column (Cytiva), dialyzed in dialysis buffer II, and concentrated before storage. Hence, MnmG used in our assays retains the 6xHis tag.

MnmE point mutants and truncation variants were obtained from smaller-scale purification using the Ni-NTA Spin Columns (QIAGEN). Briefly, samples were lysed in lysis buffer supplemented with lysozyme and benzonase endonuclease and loaded on equilibrated spin columns. Columns were washed twice in wash buffer [50 mM tris-HCl, 500 mM NaCl, 5% glycerol, and 50 mM imidazole (pH 8)]. Proteins were eluted in elution buffer and dialyzed into dialysis buffer II using Mini Slide-A-Lyzer devices (Thermo Fisher Scientific).

### Nucleotide preparation and DRaCALA assays

The radiolabeled nucleotides [5′-α-^32^P]–pppGpp, [5′-α-^32^P]–ppGpp, and [5′-α-^32^P]–pGpp were synthesized from [5′-α-^32^P]–GTP and purified as previously described (*31*, *90*). A similar approach using nonradiolabeled GTP was used to make nonradiolabeled (pp)pGpp. Differential radial capillary action ligand assay (DRaCALA) for measurement of the (p)ppGpp-binding fraction was previously outlined (*31*). We use purified hypoxanthine-guanine phosphoribosyltransferase (HPRT) protein as a positive control for (pp)pGpp binding in our assays (*91*). To estimate the apparent dissociation constants (*K*_d_), increasing concentrations of purified MnmE (from 0.313 to 40 μM) were incubated with a constant amount of radiolabeled nucleotide (typically 0.25 nM) in binding buffer [10 mM tris (pH 7.5), 100 mM NaCl, and 5 mM MgCl_2_) for 10 min at room temperature. This DRaCALA titration method was shown previously to give comparable *K*_d_ values to those determined by isothermal titration calorimetry (ITC) (*91*) and was preferred since MnmE was found to rapidly precipitate during ITC. Binding curves were generated by plotting the fraction bound against protein concentration for three replicates, and apparent dissociation constants (*K*_d_) were obtained by nonlinear regression fitting using the one-site total binding model in the Prism software (GraphPad).

### MnmE [5′-α-^32^P]–GTP and [5′-α-^32^P]–pppGpp hydrolysis assays

For GTP hydrolysis assays: A 100 μM nonradiolabeled GTP (Chem-Impex) was combined with 0.3 nM [5′-α-^32^P]–GTP (Revvity) in 1× buffer B [40 mM tris-HCl (pH 7.5), 100 mM NaCl, 10 mM MgCl_2_, and 100 mM KCl] (*32*) and incubated for 30 min at 37°C in the presence of 1.9 μM purified proteins (or corresponding volume of protein storage buffer as negative control) and nonradiolabeled competitors at concentrations indicated in the figures. Reactions were stopped by adding formic acid to a final concentration of 0.33 M; the cleavage products were resolved by thin-layer chromatography (TLC) on PEI-cellulose plates (EMD Millipore), with 0.85 M KH_2_PO_4_ (pH 3.4) buffer; and plates were exposed to a phosphor screen, which was scanned on a Typhoon FLA 9000 scanner (GE Healthcare). Nucleotides were quantified on Fiji [National Institutes of Health (NIH)]. For the calculation of IC_50_, the median inhibitory concentration of GTPase activity by ppGpp, the inhibition curves were fitted with a nonlinear regression using the Prism software (GraphPad).

For pppGpp hydrolysis assays: A 0.3 nM [5′-α-^32^P]–pppGpp was incubated in 1× buffer B for 30 min at 37°C in presence of increasing concentrations of purified protein (as listed in the figure). Reactions were stopped and resolved similarly to the GTP hydrolysis assay, except that 1.5 M KH_2_PO_4_ (pH 3.4) buffer was used for TLC.

### Growth curves with fluorescence measurements

For growth measurement, fresh colonies of *B. subtilis* strains obtained from overnight LB agar plates were resuspended, diluted to OD_600_ ≈ 0.005 in indicated growth medium, and grown in black 96-well plates with clear bottom (Corning). OD_600_ and GFP fluorescence were measured every 30 min at 37°C under vigorous shaking in the Synergy 2 microplate reader (BioTek). Plots of GFP per OD_600_ ratio over time were obtained with at least three replicates and plotted on the Prism software (GraphPad). For plots of inflection point data: Growth curves (at least three biological replicates) were smoothed using a Savitzky-Golay filter, and instantaneous growth rate (*d*OD/*dt*) was computed. The inflection point was defined as the time corresponding to the maximum growth rate as established previously (*92*), and GFP fluorescence values were linearly interpolated at the OD inflection time for the corresponding replicate. OD and GFP fluorescence at inflection were recorded, and summary statistics were calculated across replicates and plotted on the Prism software (GraphPad).

### Colony biofilm and pellicle biofilm assays

Air-liquid interface biofilms (pellicles) were grown in the outer wells of a 12-well nontreated polystyrene plate (Corning) at 37°C in 4 ml of culture medium under nonshaking conditions. We used S7 + 20AA–rich medium (*34*), MSgg minimal medium (*39*) [5 mM potassium phosphate (pH 7), 100 mM Mops (pH 7), 2 mM MgCl_2_, 700 μM CaCl_2_, 50 μM MnCl_2_, 50 μM FeCl_3_, 1 μM ZnCl_2_, 2 μM thiamine, 0.5% glycerol, and 0.5% glutamate], or a medium we define as MSGA, similar to MSgg but with 0.5% glucose and 0.5% ammonia (instead of 0.5% glycerol and 0.5% glutamate). To facilitate collection, a custom-made stainless mesh was added to the culture wells as previously described (*40*).

For colony biofilm assays, MSgg medium was modified (with 125 μM FeCl_3_) to ensure comparable iron bioavailability to liquid culture (*93*), supplemented with 1.5% (w/v) agar (VWR), and poured into 60-mm petri dishes (Thermo Fisher Scientific). Plates were incubated at 30°C for up to 3 days in a Tupperware box to maintain moisture.

Biofilm colony or pellicle morphologies were documented using photographs taken under natural light with a smartphone camera (iPhone 8). Fluorescence from the *P_tapA_*-*sfGFP* reporter was recorded using a Typhoon scanner (GE Healthcare) to quantify enhanced GFP signal across colonies; total colony fluorescence was measured using Fiji (NIH) and plotted on the Prism software (GraphPad).

For the microcolony biofilm assay, biofilm was grown in a custom-made microfluidic biofilm system with MSgg medium flow and imaged by confocal microscopy as published previously (*94*). Fluorescence projection and volume rendering were done using the Volume Viewer plugin on Fiji (NIH) on the z-stack acquired at the 24-hour time point.

### Genetic screens for biofilm mutants and suppressors

We isolated loss-of-biofilm spontaneous suppressors from the edges of WT, Δ*mnmE*, or double mutants *sinR::kan* Δ*mnmE*, *ywcC::kan* Δ*mnmE*, and *ywcC::kan* Δ*mnmG* biofilm colonies after prolonged plate incubation (up to 7 days) at 30°C. Suppressors were isolated to single colonies by restreaking smooth patches from the biofilm colony edges onto LB plates. Loss-of-biofilm phenotype of the suppressors was confirmed by repeating the colony biofilm assay on MSgg-agar plates.

The gain-of-biofilm spontaneous suppressors were isolated from the otherwise biofilm-deficient *spo0A::kan* or *spo0A::kan* Δ*mnmE* and *spo0A::kan* Δ*mnmG* double mutants on MSgg-agar plates. Suppressors appeared within 3 days of incubation at 30°C. We picked any rough patches growing on the otherwise runny/mucoid colonies, restreaking them on LB plates. Single colonies were isolated and regrown in MSgg-agar colony biofilm assays to confirm their rugous phenotype.

gDNA of the suppressors and parental strain was extracted and sent for whole-genome sequencing either using Illumina 200 Mbp at SeqCenter (Pittsburgh) or equivalent service at the Cold Spring Harbor Laboratory (NY). Last, the mutation(s) in suppressors were identified using breseq (*95*).

### RNA-seq and Ribo-seq

#### Standalone RNA-seq

For the LB exponential-growth samples: LB cultures (in flasks) were inoculated at OD_600_ ≈ 0.0003 (from a same-day LB preculture) and grown at 37°C with shaking (200 rpm) until reaching OD_600_ ≈ 0.3; a 10-ml sample was then harvested by fast filtration through a 0.22-μm nylon filter. Cells were scraped from the filter using a spatula and immediately flash frozen in liquid nitrogen before storage at −80°C.

For the pellicle biofilm samples, nonshaking cultures in 12-well plates were cultured at 37°C, and samples were harvested similarly to a previous study (*40*). Briefly, outer wells of the plate containing 4 ml of MSgg or MSGA and a metal mesh were inoculated at OD_600_ ≈ 0.002 from a late-exponential preculture. Planktonic samples were harvested after 8 hours of growth by filtration and subsequent flash freezing (*40*). Later time point (24 hours) cultures consisted of pellicle samples were harvested using the metal mesh as previously described (*40*). Because MSGA medium does not support biofilm formation in WT, this 24 hour-time point sample was still planktonic and was collected similarly to the earlier time point. Biological triplicates were collected for each condition.

Total RNA was extracted using phenol-chloroform following a published method (*96*). RNA-seq was performed by SeqCenter (Pittsburgh), as follows. Samples were treated with RNase-free deoxyribonuclease (DNase; Invitrogen). Library preparation was performed using the Stranded Total RNA Prep Ligation with Ribo-Zero Plus kit (Illumina) and 10-bp unique dual indices. Sequencing was done on a NovaSeq 6000 or NovaSeq 6000 (Illumina), producing paired-end 151-bp reads. Sequencing demultiplexing, quality control, and adapter trimming were performed with bcl-convert (v4.1.5). Analysis was done on Galaxy (usegalaxy.org) using a custom workflow (see public link in the Data, code and materials availability section) comprising quality control, trimming, mapping, quantification, and visualization steps. Briefly, quality control was performed using FastQC, low-quality bases were trimmed using Cutadapt, reads were aligned to the *B. subtilis* NCIB3610 genome (NZ_CP020102.1 and NZ_CP020103.1) using Bowtie2, and unmapped reads were removed and BAM files were coordinate sorted using SAMtools. Gene-level read counts were obtained with featureCounts, ncRNA entries were removed, coverage tracks for visualization were generated using bamCoverage, and differential expression analysis was carried out in EdgeR on triplicate featureCounts tables for each strain-pair comparison (JDW2809 versus JDW4304) across conditions (exponential, MSgg: 8 hours, MSGA: 8 hours, MSgg: 24 hours, MSGA: 24 hours). Significantly differentially expressed genes were defined by |log_2_FC| > 0.58 and FDR < 0.05. Volcano plots were generated within Galaxy using these thresholds to visualize expression changes. In the volcano plots, we labeled *mnmE* and all the genes passing thresholds and involved in biofilm according to SubtiWiki (*97*). Genome browser snapshots of RNA-seq coverage were generated in IGV (Integrative Genomics Viewer, Broad Institute) from the aligned bigWig files. Hierarchical clustering of differentially expressed genes was performed on *z*-scored normalized expression values using Euclidean distance and complete linkage. Gene clusters were defined by cutting the resulting dendrogram. Functional annotation of each gene cluster was performed by Gene Ontology overrepresentation analysis using the PANTHER Classification System (v19.0). Enrichment was assessed against the annotated *B. subtilis* gene background, and statistical significance was determined using Fisher’s exact test with FDR correction.

#### Ribo-seq and matched RNA-seq

Prewarmed MSgg medium (in a 1-liter flask) was inoculated at OD_600_ ≈ 0.01 from fresh colonies on overnight plates. Cultures were grown at 37°C with shaking (200 rpm), and OD_600_ was measured regularly. We harvested t1 samples around OD_600_ ≈ 0.4 to 0.5 (exponential phase) and t2 samples during the inflection point of growth (occurring around OD_600_ ≈ 1.6 in these growth conditions). Sample harvesting for ribosome profiling was conducted as previously recommended (*47*, *48*), where 50 ml of cultures were flash frozen directly in liquid nitrogen and stored at −80°C until lysis. Biological duplicates were collected for each condition.

Cell lysates were prepared from frozen cultures following the procedure previously published (*47*), an aliquot was saved for RNA-seq library preparation (see below), and the remainder was used for Ribo-seq (polysome profiling). For Ribo-seq library preparation: Lysates were digested with MNase for 1 hour at room temperature (*47*, *48*). Digestions were quenched with EGTA to a final concentration of 6 mM, and monosomes were purified by size exclusion chromatography on MicroSpin S-400 HR columns (GE Healthcare) as described earlier (*98*). Size selection of footprints with length of 25 to 40 nt was performed by electrophoresis on 15% acrylamide tris-borate EDTA–urea gels (Invitrogen). Illumina-ready Ribo-seq libraries were prepared using a SMARTer smRNA-seq kit (Takara Bio). Library concentrations were measured by Qubit fluorometer (Thermo Fisher Scientific), and their quality was assessed on a 2100 bioanalyzer (Agilent). Ribo-seq libraries were sequenced on a NovaSeq 6000 sequencer (Illumina), single read, 1 × 50 cycles.

For the matched RNA-seq: Total RNA was purified from an aliquot of cell lysate using the RNA Clean & Concentrator −5 kit (Zymo Research). rRNA was depleted from total RNA using Ribo-Zero plus rRNA depletion kit (Illumina). mRNA fragmentation was conducted for 20 min at 94°C to generate RNA fragments with similar sizes to those of the ribosome footprints. SMARTer smRNA-seq kit (Takara Bio) was used to generate Illumina-ready RNA-seq libraries. Library concentrations were measured by Qubit fluorometer (Thermo Fisher Scientific), and their quality was assessed on a 2100 bioanalyzer (Agilent). RNA-seq libraries were sequenced on a NovaSeq 6000 sequencer (Illumina), single read, 1 × 50 cycles.

After sequencing and demultiplexing, all sequences added during library preparation were removed from the reads. Trimmed Ribo-seq and RNA-seq reads were analyzed using the HRIBO workflow (*54*), and *B. subtilis* NCIB3610 genome (NZ_CP020102.1 and NZ_CP020103.1) was used as a reference. Contrast of interest was Δ*mnmE* versus WT at t1 (exponential phase) or t2 (inflection point). Output table (.xlsx) of the HRIBO run can be found in table S6, while all HRIBO output files are deposited in our Mendeley data repository (see the Data, code, and materials availability section). Genome browser snapshots of RNA-seq and Ribo-seq coverage were generated in IGV (Broad Institute) from the aligned bigWig files. For visualization of the P-site ribosome position, 5′-assigned read positions were shifted by 18 nucleotides as determined in our metagene analysis (see below). Many genes from the *B. subtilis* NCIB3610 genome were missing functional annotation; therefore, functional annotation in table S7 was manually complemented with the “functional categories” from SubtiWiki (*97*).

### Metagene analysis from Ribo-seq data

HRIBO (*54*)–embedded metagene analysis was run with the following parameters: positionsOutsideORF: 50; positionsInORF: 100; filteringMethods: [“overlap”, “length”, “rpkm”]; neighboringGenesDistance: 100; rpkmThreshold: 10.0; lengthCutoff: 50; mappingMethods: [“fiveprime”, “threeprime”]; readLengths: “25–34”. Previously published Ribo-seq datasets from *E. coli* [Gene Expression Omnibus (GEO), accession number GSE64488, sample GSM1572273] (*50*) or *B. subtilis* (GEO, accession number GSE95211, sample GSM2500125) (*51*) were processed in the same way for comparison. Metagene analysis results of 5′-mapped and 3′-mapped reads aligned to the stop or start codon were plotted on three-dimensional (3D) plots using custom Python scripts (relying on NumPy, Pandas, and Matplotlib). We did metagene analysis of reads aligned to the start codon (initiating ribosomes) and looked at influence of read length on the distance to P-site, an analytical method previously published for *E. coli* datasets (*50*)*.* However, we determined that for *B. subtilis* datasets, 5′-end assignment method of ribosome footprints is the most accurate for gaining nucleotide resolution because the distance is not varying with read length. Thus, with the 3D-plot analysis, we saw that the 5′ end is constantly located 18 nt upstream of the P-site regardless of read length, and we determined the following 5′-end position offsets that we use later on: 21 nucleotides for the A-site, 18 nucleotides for the P-site, and 15 nucleotides for the E-site of the ribosome.

### Genome-wide codon pausing analysis from Ribo-seq data

Custom Python scripts were developed to compute (for each condition) the A-site genome-wide (average) coverage of each sense codon normalized to its respective mRNA level to account for differences in transcriptome. Briefly: 5′-assigned, length-filtered RPF coverage was retrieved as strand-specific, per-nucleotide bigWig files (forward and reverse fiveprime tracks, min normalized) from the HRIBO output (*54*). In parallel, from the HRIBO overview tables and total read count summaries, we computed reads per nucleotide (RPN) for each CDS (RPKM × total mapped reads/10^9^) and restricted the analysis to nonoverlapping protein-coding genes with annotated amino acid sequences and sufficient ribosome occupancy (RPN ≥ 0.33 in all samples). For this filtered gene set, in-frame codon coordinates were derived from the GFF and converted to a BED file, excluding the first two and last two codons of each ORF to minimize initiation/termination effects. Fiveprime bigWig files were converted to wiggle/bedGraph; shifted to align A-, P-, and E-site positions based on metagene calibration; forward and reverse strands were merged and cleaned; and sorted coverage tracks were reconverted to bigWig for efficient querying. Codon-level A-site coverage was then extracted by intersecting the shifted bigWig files with codon intervals in the BED file, yielding mean coverage per codon triplet (61 sense codons, stop codons excluded) across the genome for each sample. Matching RNA-seq–derived codon coverage distributions were obtained analogously. For each condition and replicate, codon-wise A-site ribosome (RIBO) coverage was normalized to the total genome-wide coverage and plotted against normalized RNA coverage following a published method (*52*). For each codon and condition, we calculated RIBO/RNA coverage ratios, propagated SDs across biological replicates, and derived *z*-scores to rank codons by the magnitude of change in ribosome occupancy adjusted for uncertainty (without formal hypothesis testing given; *n* = 2). All custom Python scripts used for codon coordinate generation, bigWig processing, codon coverage, and *z*-score calculations have been packaged in a Bash script (CodonPausingKit) that is available at our GitHub public repository (see Data, code, and materials availability section).

### AlphaFold3 costructure predictions

AlphaFold3 (DeepMind; version 3.0.0) was used for the predictions of (i) *B. subtilis* MnmE dimer bound to two GTP, two magnesium ions, and two formyl-tetrahydrofolate molecules; (ii) *B. subtilis* MnmE dimer bound to two ppGpp, two magnesium ions, and two formyl-tetrahydrofolate molecules; and (iii) *B. subtilis* MnmE dimer bound to two GDP, two magnesium ions, and two formyl-tetrahydrofolate molecules. MnmE is known to function as a dimer, and formyl-tetrahydrofolate serves as a carbon donor for the (cm)nm^5^ tRNA modification, while the magnesium ion is required for GTP binding (*99*). The full-length amino acid sequence of *B. subtilis* MnmE (UniProt accession: P25811) was provided in FASTA format within the AlphaFold3 JSON input. Ligands were explicitly specified using Chemical Component Dictionary identifiers (“GTP” for GTP; “GDP” for GDP; “G4P” for ppGpp; “MG” for magnesium ion; “FON” for formyl-tetrahydrofolate) and were modeled directly during inference.

The default AlphaFold3 data pipeline was used without modification, and no externally precomputed MSAs or templates were supplied. Structure prediction was performed using default inference parameters in protein-ligand complex mode. Inference was conducted using a single seed, with the default number of recycles (*10*). No positional restraints or post hoc docking procedures were applied. Data pipeline computations were executed on Intel “Sapphire Rapids” CPUs, and model inference was performed on NVIDIA A100 GPUs (A100 3g.20gb configuration) on the Harvard FAS Research Computing (FASRC) cluster.

Models were ranked using AlphaFold3 internal confidence metrics, including mean predicted local distance difference test (pLDDT) and predicted aligned error (PAE). The top-ranked model for each condition was selected for downstream analysis. Per-residue pLDDT values were mapped onto structural coordinates, and corresponding PAE matrices were extracted from the output files. The top-ranked models colored by pLDDT and their PAE plots are provided in fig. S2 (A to C). The AlphaFold3 input JSON files and outputs obtained for all predictions have been deposited in our public Mendeley data repository to facilitate reproducibility (see the Data, code and materials availability section).

### Western blot

Cultures were grown to mid-exponential phase in LB medium; cells were harvested, lysed in lysis buffer [10 mM tris (pH 7.5), 100 mM NaCl, 5 mM MgCl_2_, lysozyme (0.25 mg/ml), 1 μM phenylmethylsulfonyl fluoride, and DNase I (10 μg/ml)] by freeze-thaw cycles, and centrifuged to pellet cell debris. Crude whole-cell lysates were diluted to normalize their concentration, and 5 μg of each sample was mixed with equal volume of 2× Tricine Sample Buffer (Bio-Rad) with 2% fresh β-mercaptoethanol. Samples were denatured for 5 min at 95°C, ran on a Mini-Protean Tris-Tricine Precast gel 16.5% (Bio-Rad) for 1 hour 40 min at 100 V, and transferred onto 0.2-μm nitrocellulose membrane (Bio-Rad) in Towbin buffer [25 mM tris, 192 mM glycine, and 20% (v/v) methanol (pH 8.3)]. Nitrocellulose membranes were air dried and stained using Revert 700 Total Protein Stain (LICORbio) according to the manufacturer instructions. Membrane was imaged in the 700-nm channel using the Odyssey CLx Imager (LICORbio) before blocking in Intercept Blocking Buffer (LICORbio) at room temperature for 1 hour. Membrane was then hybridized with AbrB antibodies (gift from E. Denham and J. Dworkin). Primary antibodies were visualized with fluorescent IRDye 800CW goat anti-rabbit IgG secondary antibodies (LICORbio) following the manufacturer instructions at 800 nm on the Odyssey CLx Imager (LICORbio). AbrB signal was quantified and normalized against total protein strain signal on the Image Studio software (LICORbio), and results obtained across biological triplicates were plotted on the Prism software (GraphPad).

### Mass photometry

Oligomerization of MnmE (with or without ppGpp) was studied using mass photometry on a OneMP mass photometer (Refeyn). Phosphate-buffered saline (PBS; containing 137 mM NaCl, 2.7 mM KCl, 10 mM Na_2_HPO_4_, and 1.8 mM KH_2_PO_4_) was used in all measurements. Bovine serum albumin (MilliporeSigma) in 1× PBS was used for instrument calibration. For the MnmE measurements, 15 μl of 1× PBS was preloaded into a silicone well (Grace Bio-Labs), and then 1 μl of a 750 nM MnmE protein solution was mixed in before acquisition (47 nM MnmE final). ppGpp was subsequently added at 180 μM final concentration, and acquisition was done immediately or after 10 min of incubation. Analysis of the data was done on DiscoverMP software (Refeyn).
