# Supplementary material for: Direct regulation of a tRNA modification enzyme by ppGpp links stress signaling to translational control and biofilm development
Source: Sci Adv. 2026 Nov 4;12(45):eaek6976. doi: 10.1126/sciadv.aek6976 (PMC13733827; doi:10.1126/sciadv.aek6976)
Supplement: Supplementary file 1 [file sciadv.aek6976_sm.pdf]

Science Advances
AAAS

Supplementary Materials for

Direct regulation of a tRNA modification enzyme by ppGpp links stress signaling to translational control and biofilm development

Aude E. Trinquier *et al.*

Corresponding author: Aude E. Trinquier, trinquier@wisc.edu; Jue D. Wang, wang@bact.wisc.edu

Sci. Adv. **12**, eaek6976 (2026)
DOI: 10.1126/sciadv.aek6976

The PDF file includes:

Figs. S1 to S7
Tables S1 to S5
Legends for tables S6 and S7

Other Supplementary Material for this manuscript includes the following:

Tables S6 and S7

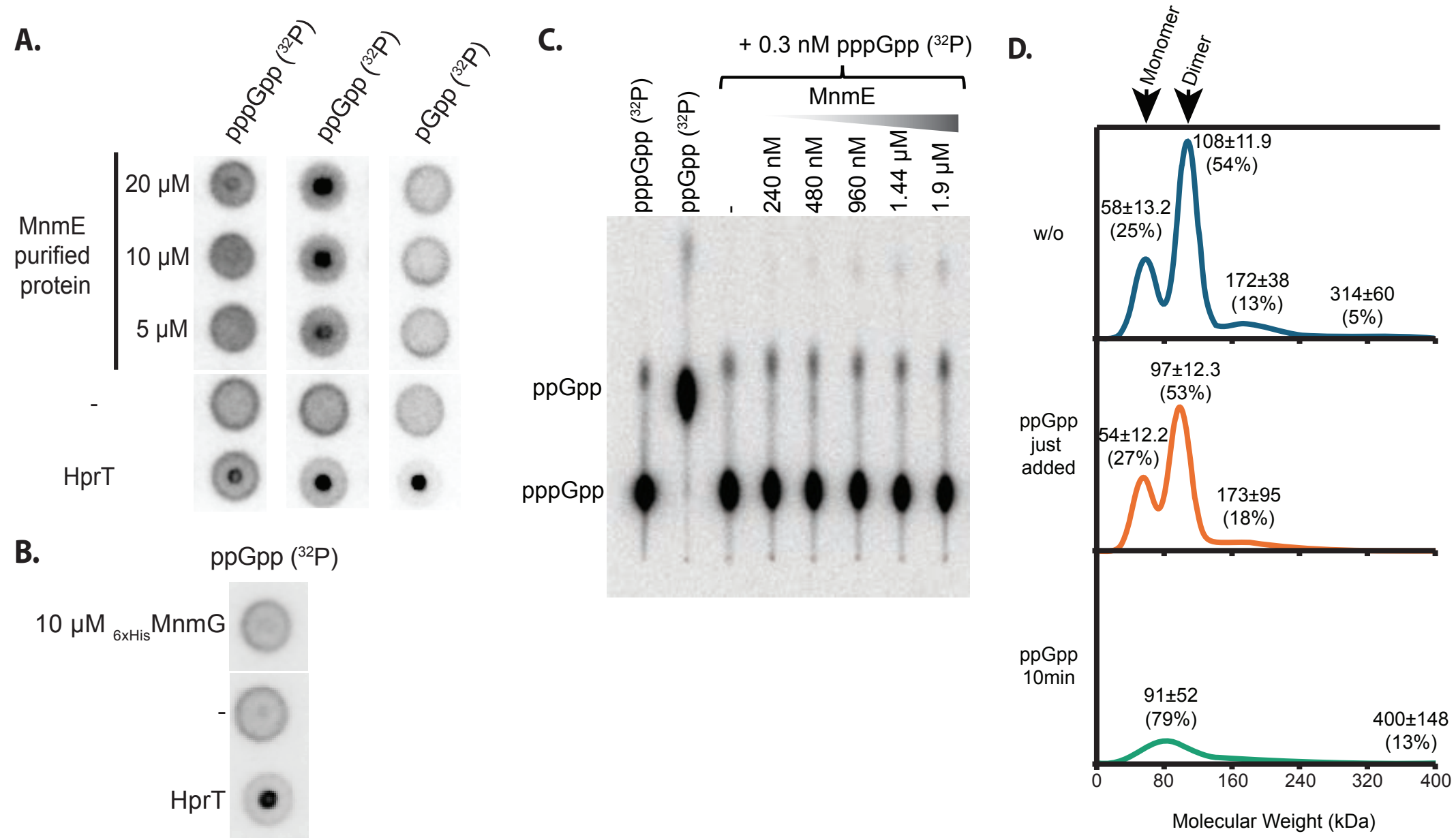

Figure S1. Additional analyses supporting Figure 1.

(A) DRaCALA assay of [5´-α-32P]-pppGpp, ppGpp and pGpp binding to different amounts of purified B. subtilis MnmE protein. HprT purified protein is shown as a positive (pp)pGpp-binding control.

(B) DRaCALA assay of [5´-α-32P]-ppGpp showing absence of binding to purified *B. subtilis* 6xHisMnmG protein. HprT purified protein is shown as a positive (pp)pGpp-binding control

(C) [5´-α-32P]-pppGpp hydrolysis assay in presence of increasing concentrations of purified MnmE protein shows no pppGpp hydrolysis activity.

(D) Representative distribution of MnmE monomeric, dimeric, and higher oligomeric species in the absence of ppGpp (blue), with 180 µM ppGpp just added (orange), or after 10 min incubation (green) in a mass photometry experiment.

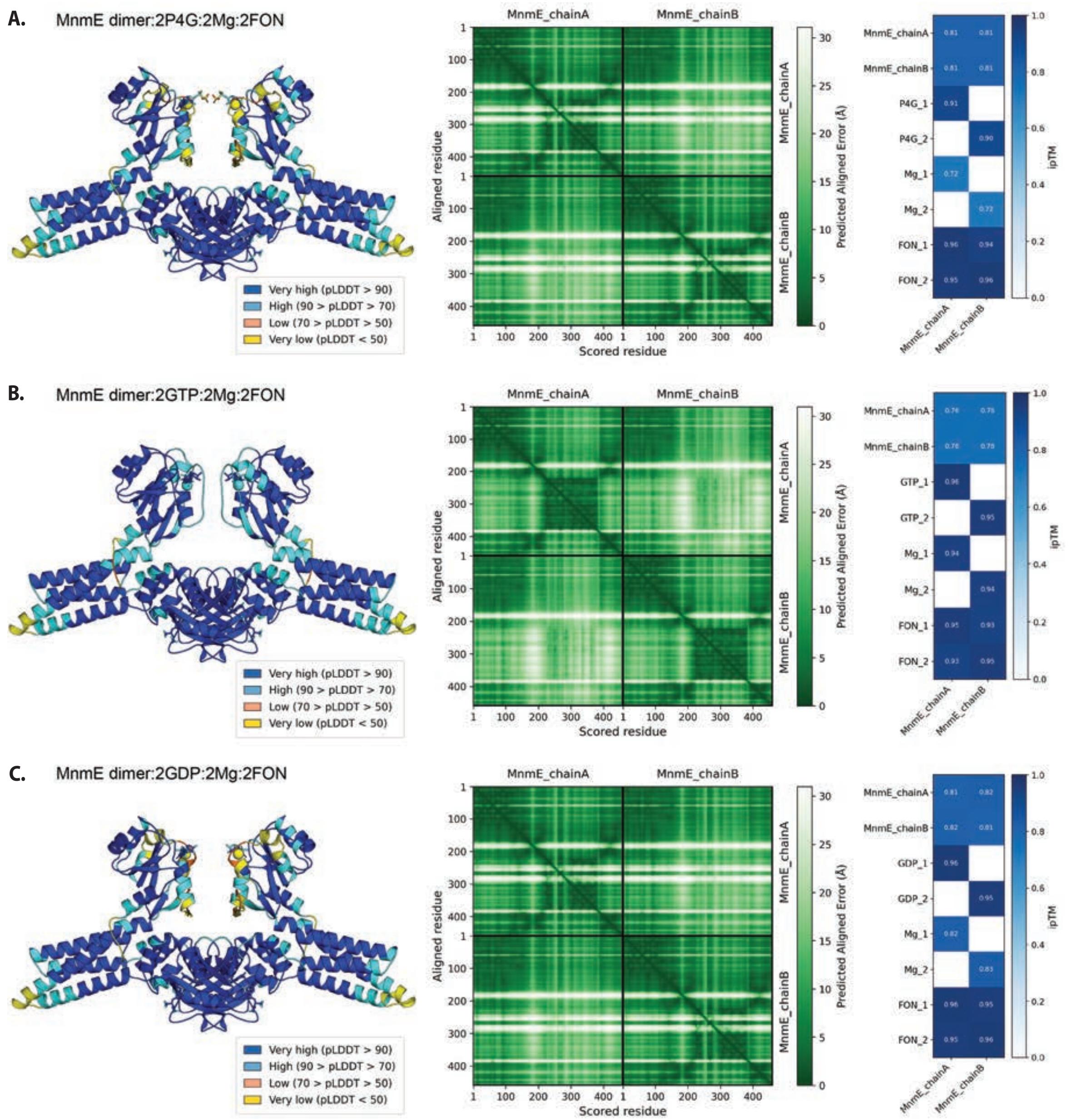

Figure S2. Additional analyses supporting Figure 1.

Three-panel confidence summary for AlphaFold3 co-structure predictions. Left: Local structural confidence with colored by per-residue pLDDT (predicted Local Distance Difference Test). Center: Relative domain positioning confidence as a protein-only PAE (Predicted Aligned Error) plot. Right: Interface confidence as iPTM (interface Predicted TM-score) score matrix. (A) corresponds to MnmE-ppGpp prediction, (B) is MnmE-GTP prediction, and (C) is MnmE-GDP prediction. Since ppGpp interacts with MnmE in essentially the same way as GDP, the heat maps are almost identical (there are slight differences).

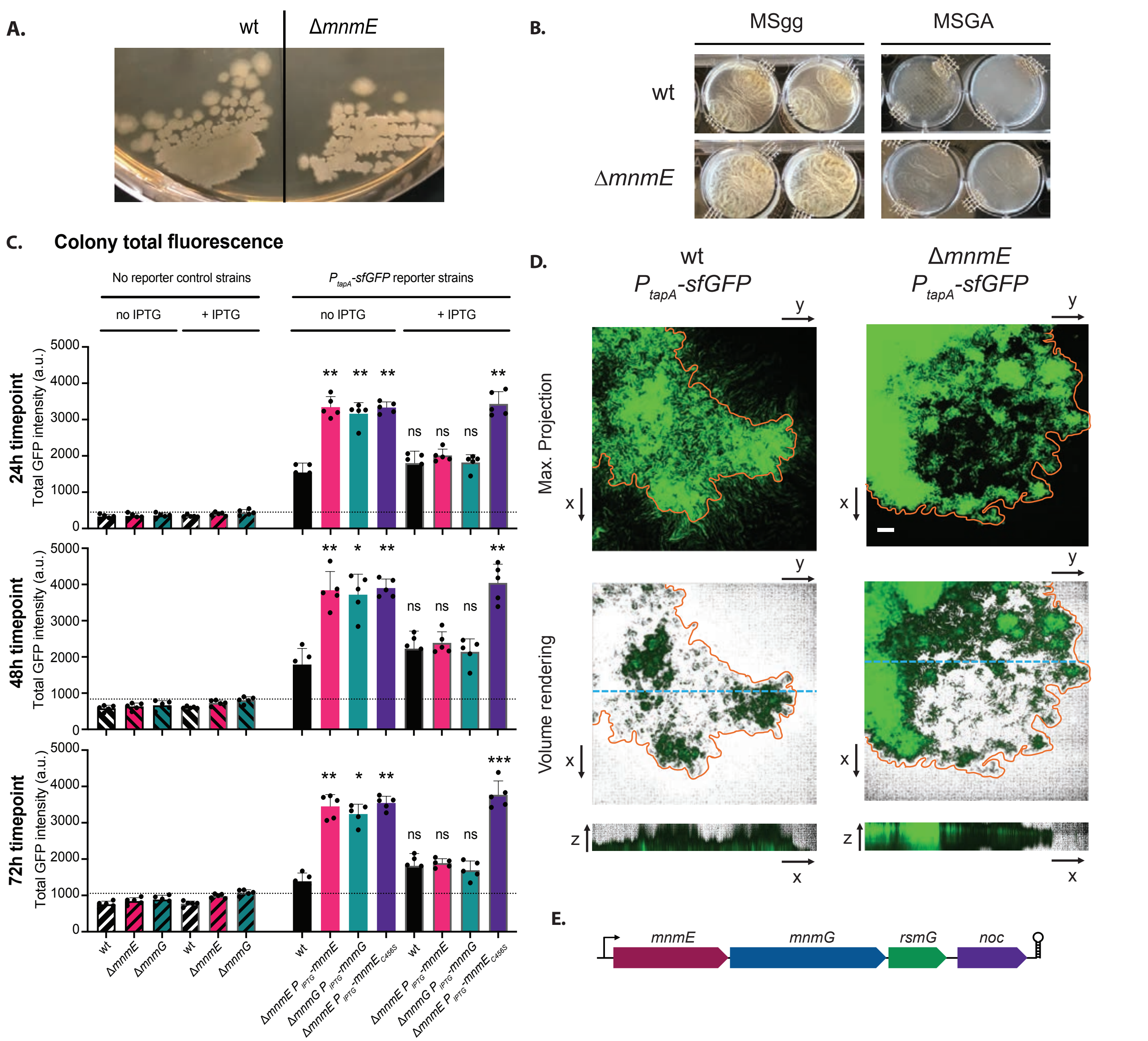

Figure S3. Additional analyses supporting Figure 2.

(A) Colony morphology of WT and Δ*mnmE* strains streaked on LB agar plate and grown overnight at 37°C.

(B) Pellicle biofilms grown in static cultures of biofilm promoting medium (MSgg) or not-biofilm-promoting medium (MSGA) for 24h at 37°C in presence of metal mesh (like previously described in(40)).

(C) Quantification of Fig. 2C *PtapA-sfGFP* reporter assays: colony fluorescence was quantified from the fluorescence scan for each strain grown in presence or absence of IPTG. To account for cell autofluorescence, corresponding strains without the *PtapA-sfGFP* reporter were also assayed and are shown as controls. The dashed line represents resulting colony autofluorescence threshold for the timepoint. Error bars represent standard deviation for five replicates. Statistical significance was assessed using a Kruskal-Wallis test with Dunn's multiple-comparisons correction, comparing each condition to wild type (no IPTG); adjusted p values (*** for <0.001, ** for <0.01, * for <0.05, and ns for non-significant).

(D) Fluorescence rendering of *PtapA-sfGFP* micro-colony biofilm grown in a microfluidic device at 24 hours in MSgg medium. Upper x-y panel represents the maximum fluorescence over the z-stack projected in 2D. Middle panel reflects the volume of the colony over the z-stack rendered over the x-y. We used the volume rendering to delimit the 3D wrinkled zone (orange line) and overlaid that zone on the upper panel. This reveals that wild type biofilm colony has two distinct zones, thick wrinkles towards the center of the colony and an un-wrinkled edge where cell bundles are in the same focal plan. On the contrary, Δ*mnmE* colony appears thick and wrinkled all the way towards colony edge and part of the colony is out-of-focus because it is much thicker than the wild type. The lower panel shows an x-y slice of the volume rendering done over the blue dashed line. Scale bar, 50 µm.

(E) Map of the *B. subtilis mnmE* and *mnmG* operon. Black arrow represents the promoter of the transcriptional unit, and the hairpin represents terminator.

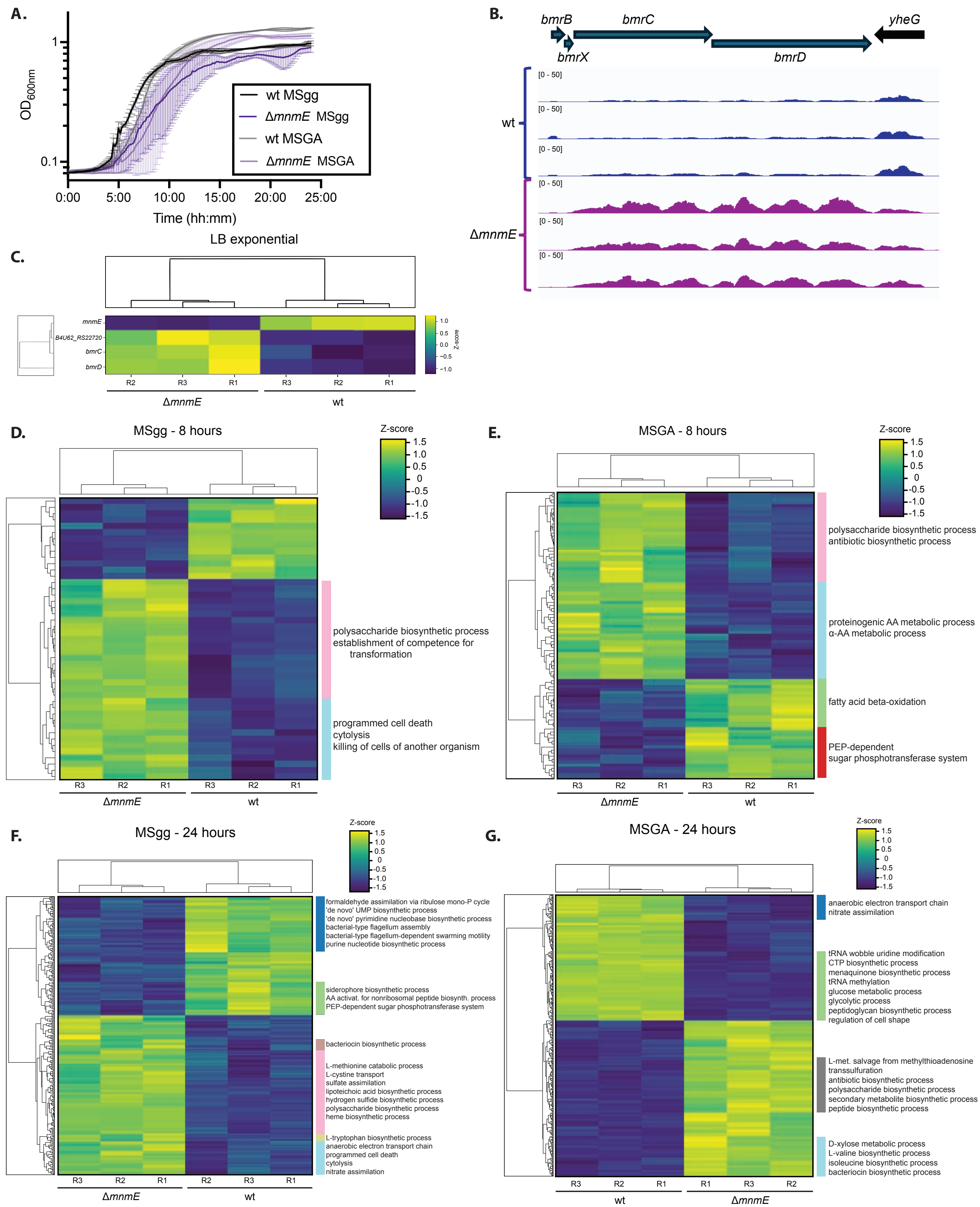

Figure S4. Additional analyses supporting Figure 4.
(A) Growth curves (OD600nm) of WT and Δ*mnmE* grown in the plate reader with shaking in MSgg or MSGA medium. Error bars represent standard deviation across triplicates, and as expected, biofilm formation leads to higher variability in OD600nm readings. (B) IGV view of the LB exponential RNA-seq reads aligned to *bmrBXCD* operon for both WT (blue) and Δ*mnmE* (purple) triplicates. (C-G) Hierarchical clustering heatmaps of differential gene expression (Δ*mnmE* vs. WT). Enrichment of functional categories was determined using gene ontology analysis. (C) is in LB exponential phase; (D) static cultures in MSgg medium after 8 hours; (E) static cultures in MSGA medium after 8 hours; (F) static cultures in MSgg medium after 24 hours; (G) static cultures in MSGA medium after 24 hours.

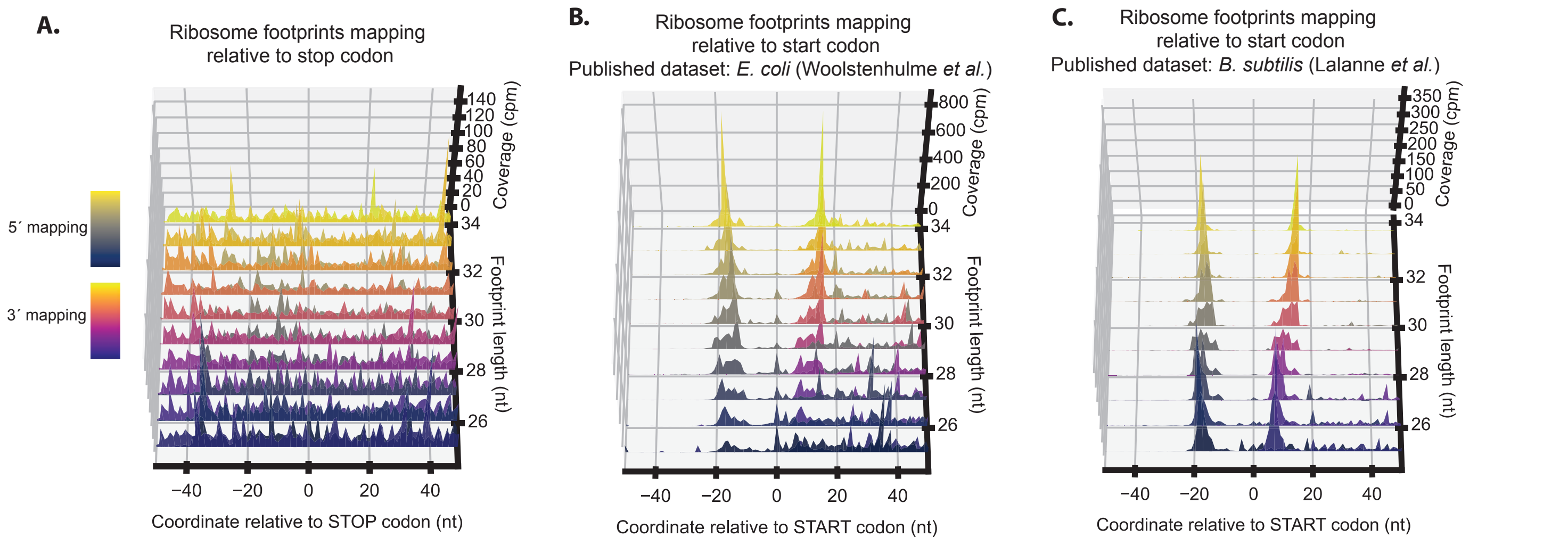

Figure S5. Additional analyses supporting Figure 5.

(A) Average ribosome occupancy aligned at the stop codon depending on footprint length. Reads from our wt t1-1 dataset were binned based on lengths and assigned positions using the 5´-end of the reads (cold tone scale) or the 3´-end of the reads (warm tone scale). Coverage at stop codon is low and does not show discrete peaks. Similar results were obtained with the other datasets of our study.

(B) Same analysis as in Fig. 5A was done on a published LB wild-type *E. o li* Ribo-seq dataset from the Burskirk lab (Gene Expression Omnibus, accession number GSE64488, sample GSM1572273)(50).

(C) Same analysis as in Fig. 5A was done on a published LB wild-type *B. subtilis* Ribo-seq dataset from the Li lab (Gene Expression Omnibus, accession number GEO: GSE95211, sample GSM2500125) (51).

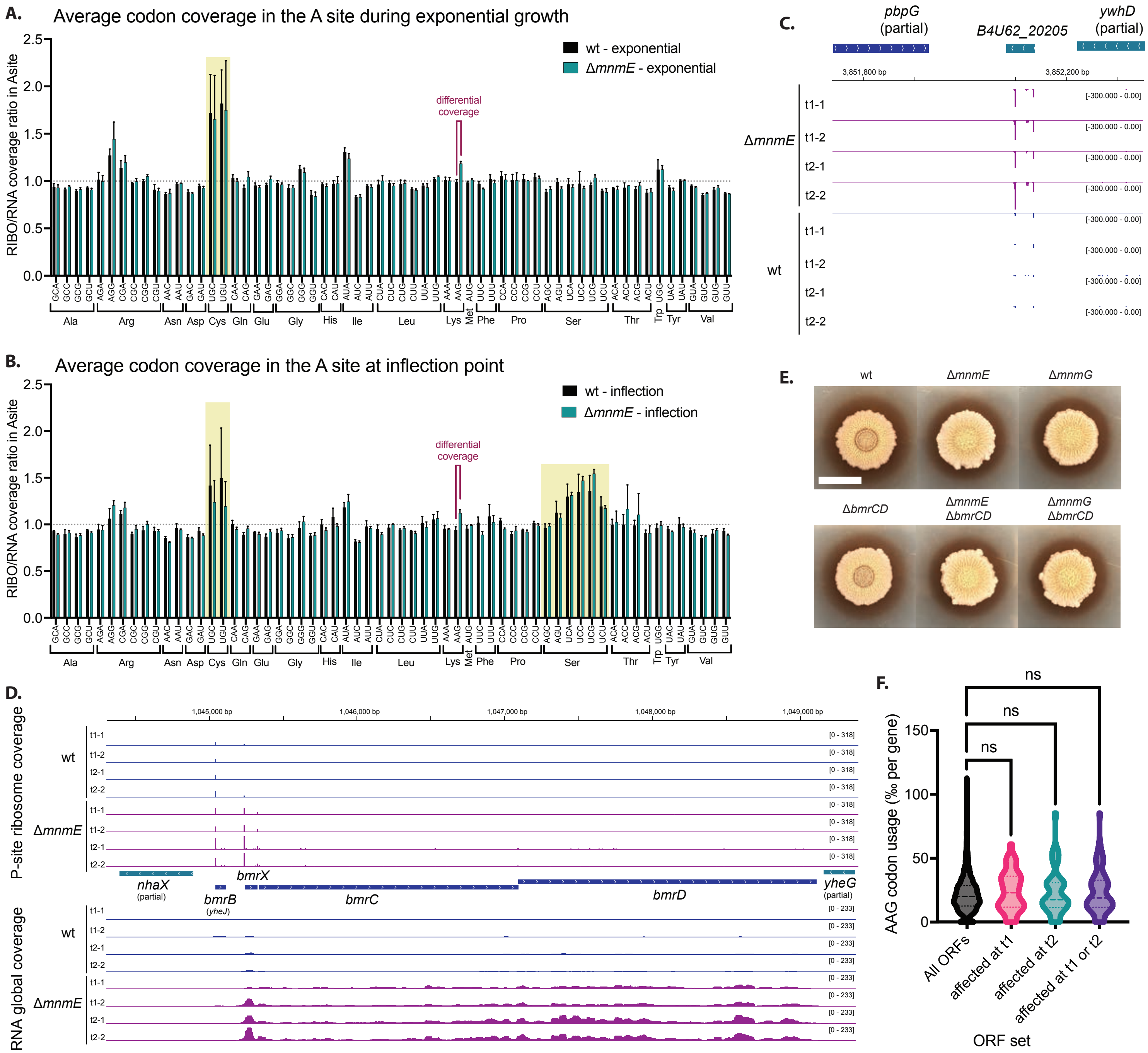

Figure S6. Additional analyses supporting Figure 5.

(A) Average A-site normalized codon coverage for elongating ribosomes in exponential phase Ribo-seq experiment. Dashed line shows a theoretical coverage ratio of 1 across coding codons, and yellow box highlights elevated Cys codon coverage in biofilm promoting conditions(52). Differential coverage at Lys-AAG between Δ*mnmE* and WT is shown in purple.

(B) Average A-site normalized codon coverage for elongating ribosomes in inflection point Ribo-seq experiment. Dashed line shows a theoretical coverage ratio of 1 across coding codons and yellow box highlights elevated Cys and Ser codon coverage in biofilm promoting media(52). Differential coverage at Lys-AAG between Δ*mnmE* and WT is shown in purple.

(C) IGV view of the P-site ribosome coverage aligned to the *pbpG-ywhD* intergenic region for both wt (blue) and Δ*mnmE* (purple) samples. t1 is exponential phase, and t2 is inflection point.

(D) IGV view of the P-site ribosome coverage (upper panel) and RNA-seq reads (lower panel) aligned to the *bmrBXCD* operon region for both wt (blue) and Δ*mnmE* (purple) samples. t1 is exponential phase, and t2 is inflection point.

(E) Epistatic study of *bmrCD* genes relative to *mnmE* and *mnmG* in the context of colony biofilm formation. Macroscopic image of colony biofilms grown at 30°C for 72 hours on MSgg agar plates. Scale bar, 1cm.

(F) Distribution of Lysine-AAG codon usage across all ORFs or selected gene sets. Violin plots show the distribution of per-gene AAG codon frequency (‰ per gene) for all ORFs (black) and differentially translated genes at t1 (exponential phase, pink), t2 (inflection point, green), or any timepoint (purple) between Δ*mnmE* and WT. Horizontal lines indicate median and quartiles. Statistical comparisons between subsets and all ORFs were performed using Kruskal-Wallis test with multiple comparison correction (ns, non-significant).

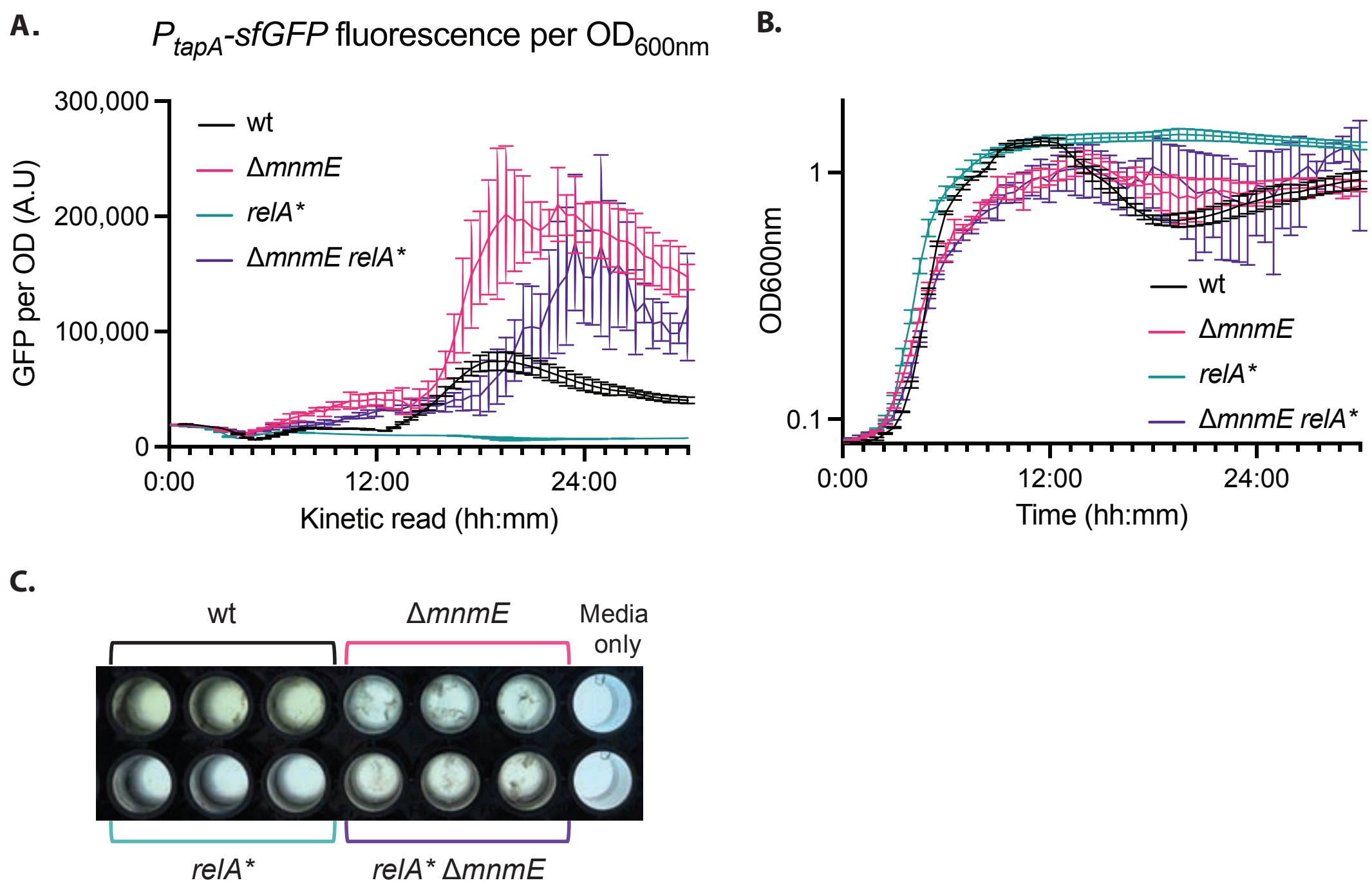

Figure S7. Additional analyses supporting Figure 7.
(A) *PtapA-sfGFP* fluorescence to OD ratio acquired over time in S7+20AA medium in the plate reader. Error bars indicate standard deviation across triplicates.
(B) Growth curves (OD600nm) associated with Fig.S7A.
(C) Picture of the 96-well plate taken at the end of the assay for Fig. 7D showing presence of visible biofilm matrix fragments in all strains except *relA**.

Table S1. Strain list

Strain number	Genotype	Construction details	Source
JDW2809	*B. subtilis* NCIB 3610 *comI* (Q12L)	published wt	PMID: 23836866
JDW3928	*B. subtilis* NCIB 3610 *ycgO::PtapA-sfGFP(Cm)*	gift	Ophelia S. Venturelli lab
JDW4247	*B. subtilis* NCIB 3610 comI(Q12L) *relA*(D264G)*	Made by CRISPR/Cas9 editing: multimerized pJW840 was used to edit *relA* ORF towards synthetase dead *relA** D264G in JDW2809.	This study
JDW4253	*E. coli* BL21 *lacIq* pJW756 (*pLIC- trpC_HA*-Bs_*mnmE*)	pJW756 plasmid transformed in E. coli BL21.	This study
JDW4284	*E. coli* BL21 *lacIq* pJW763 (*pLIC- trpC_HA*-Bs_*mnmG*)	pJW763 plasmid transformed in E. coli BL21.	This study
JDW4293	*E. coli* BL21 *lacIq* pJW764 (*pLIC- trpC_HA*-Bs_*mnmE(D275A)*)	pJW764 plasmid transformed in E. coli BL21.	This study
JDW4294	*E. coli* BL21 *lacIq* pJW765 (*pLIC- trpC_HA*-Bs_*mnmE(E287A)*)	pJW765 plasmid transformed in E. coli BL21.	This study
JDW4295	*E. coli* BL21 *lacIq* pJW766 (*pLIC- trpC_HA*-Bs_*mnmE(C456S)*)	pJW766 plasmid transformed in E. coli BL21.	This study
JDW4296	*E. coli* BL21 *lacIq* pJW767 (*pLIC- trpC_HA*-Bs_*mnmE(D338N)*)	pJW767 plasmid transformed in E. coli BL21.	This study
JDW4298	*E. coli* BL21 *lacIq* pJW772 (*pLIC- trpC_HA*-Bs_*mnmE(G233A)*)	pJW772 plasmid transformed in E. coli BL21.	This study
JDW4302	*E. coli* BL21 *lacIq* pJW773 (*pLIC- trpC_HA*-Bs_*mnmE-Gdomain*)	pJW773 plasmid transformed in E. coli BL21.	This study
JDW4304	*B. subtilis* NCIB 3610 *comI* (Q12L) Δ*mnmE*	Made by homologous recombination. PCR product of *mnmE::kan* (with 500bp flanking regions) was amplified from BKK41020 (BGSC) using oJW4383+oJW4384 and transformed in JDW2809. In a second step, cassette was looped-out using pDR244 to get a marker-less Δ*mnmE* strain.	This study
JDW4305	*B. subtilis* NCIB 3610 *comI* (Q12L) Δ*mnmG*	Made by homologous recombination. PCR product of *mnmG::kan* (with 500bp flanking regions) was amplified from BKK41010 (BGSC) using oJW4385+oJW4386 and transformed in JDW2809. In a second step, cassette was looped-out using pDR244 to get a marker-less Δ*mnmG* strain.	This study
JDW4310	*B. subtilis* NCIB 3610 *comI* (Q12L) Δ*mnmE* *amyE::Pspank-mnmE(D338N)*	Made by homologous recombination: linearized plasmid pJW777 was integrated at *amyE* locus of JDW4304.	This study
JDW4311	*B. subtilis* NCIB 3610 *comI* (Q12L) Δ*mnmE* *amyE::Pspank-mnmE(G233A)*	Made by homologous recombination: linearized plasmid pJW778 was integrated at *amyE* locus of JDW4304.	This study
JDW4312	*B. subtilis* NCIB 3610 *comI* (Q12L) Δ*mnmE* *amyE::Pspank-mnmE*	Made by homologous recombination: linearized plasmid pJW776 was integrated at *amyE* locus of JDW4304.	This study
JDW4351	*B. subtilis* NCIB 3610 *comI* (Q12L) *ycgO::PtapA-sfGFP(Cm)*	Made by homologous recombination. The biofilm reporter and flanking regions was amplified by PCR using oJW4515+oJW4516 (on genomic DNA from JDW3928) and transformed into JDW2809.	This study
JDW4352	*B. subtilis* NCIB 3610 *comI* (Q12L) Δ*mnmE* *amyE::Pspank-mnmE(wt) ycgO::PtapA-sfGFP(Cm)*	Made by homologous recombination. First pJW776 was integrated at *amyE* locus of JDW4304. Then, the biofilm reporter and flanking regions was amplified by PCR using oJW4515+oJW4516 (on genomic DNA from JDW3928) and transformed into complementation strain.	This study
JDW4353	*B. subtilis* NCIB 3610 *comI* (Q12L) Δ*mnmG* *amyE::Pspank-mnmG(wt) ycgO::PtapA-sfGFP(Cm)*	Made by homologous recombination. First pJW778 was integrated at *amyE* locus in JDW4305. The biofilm reporter and flanking regions was amplified by PCR using oJW4515+oJW4516 (on genomic DNA from JDW3928) and transformed into complementation strain.	This study

Strain number	Genotype	Construction details	Source
JDW4354	*B. subtilis* NCIB 3610 comI(Q12L) *sinR::kan*	Made by homologous recombination. PCR product of *sinR::kan* (with 500bp flanking regions) was amplified from BKK24610 (BGSC) using oJW4521+oJW4522 and transformed in JDW2809.	This study
JDW4355	*B. subtilis* NCIB 3610 comI(Q12L) Δ*mnmE* *sinR::kan*	Made by homologous recombination. PCR product of *sinR::kan* (with 500bp flanking regions) was amplified from BKK24610 (BGSC) using oJW4521+oJW4522 and transformed in JDW4304.	This study
JDW4356	*B. subtilis* NCIB 3610 comI(Q12L) *sinI::kan*	Made by homologous recombination. PCR product of *sinI::kan* (with 500bp flanking regions) was amplified from BKK24600 (BGSC) using oJW4523+oJW4524 and transformed in JDW2809.	This study
JDW4357	*B. subtilis* NCIB 3610 comI(Q12L) Δ*mnmE* *sinI::kan*	Made by homologous recombination. PCR product of *sinI::kan* (with 500bp flanking regions) was amplified from BKK24600 (BGSC) using oJW4523+oJW4524 and transformed in JDW4304.	This study
JDW4358	*B. subtilis* NCIB 3610 comI(Q12L) Δ*mnmG* *sinI::kan*	Made by homologous recombination. PCR product of *sinI::kan* (with 500bp flanking regions) was amplified from BKK24600 (BGSC) using oJW4523+oJW4524 and transformed in JDW4305.	This study
JDW4359	*B. subtilis* NCIB 3610 comI(Q12L) *ywcC::kan*	Made by homologous recombination. PCR product of *ywcC::kan* (with 500bp flanking regions) was amplified from BKK38220 (BGSC) using oJW4525+oJW4526 and transformed in JDW2809.	This study
JDW4360	*B. subtilis* NCIB 3610 comI(Q12L) Δ*mnmE* *ywcC::kan*	Made by homologous recombination. PCR product of *ywcC::kan* (with 500bp flanking regions) was amplified from BKK38220 (BGSC) using oJW4525+oJW4526 and transformed in JDW4304.	This study
JDW4361	*B. subtilis* NCIB 3610 comI(Q12L) Δ*mnmG* *ywcC::kan*	Made by homologous recombination. PCR product of *ywcC::kan* (with 500bp flanking regions) was amplified from BKK38220 (BGSC) using oJW4525+oJW4526 and transformed in JDW4305.	This study
JDW4362	*B. subtilis* NCIB 3610 comI(Q12L) *abrB::kan*	Made by homologous recombination. PCR product of *abrB::kan* (with 500bp flanking regions) was amplified from BKK00370 (BGSC) using oJW4527+oJW4528 and transformed in JDW2809.	This study
JDW4363	*B. subtilis* NCIB 3610 comI(Q12L) Δ*mnmE* *abrB::kan*	Made by homologous recombination. PCR product of *abrB::kan* (with 500bp flanking regions) was amplified from BKK00370 (BGSC) using oJW4527+oJW4528 and transformed in JDW4304.	This study
JDW4364	*B. subtilis* NCIB 3610 comI(Q12L) Δ*mnmG* *abrB::kan*	Made by homologous recombination. PCR product of *abrB::kan* (with 500bp flanking regions) was amplified from BKK00370 (BGSC) using oJW4527+oJW4528 and transformed in JDW4305.	This study
JDW4394	*B. subtilis* NCIB 3610 *comI* (Q12L) *cspR::kan*	Made by homologous recombination. PCR product of *cspR::kan* (with 500bp flanking regions) was amplified from BKK08930 (BGSC) using oJW4571+oJW4572 and transformed in JDW2809.	This study
JDW4395	*B. subtilis* NCIB 3610 *comI* (Q12L) *rsmG::kan*	Made by homologous recombination. PCR product of *rsmG::kan* (with 500bp flanking regions) was amplified from BKK41000 (BGSC) using oJW4583+oJW4584 and transformed in JDW2809.	This study
JDW4396	*B. subtilis* NCIB 3610 *comI* (Q12L) *miaA::kan*	Made by homologous recombination. PCR product of *miaA::kan* (with 500bp flanking regions) was amplified from BKK17330 (BGSC) using oJW4575+oJW4576 and transformed in JDW2809.	This study
JDW4397	*B. subtilis* NCIB 3610 *comI* (Q12L) *trmR::kan*	Made by homologous recombination. PCR product of *trmR::kan* (with 500bp flanking regions) was amplified from BKK27360 (BGSC) using oJW4567+oJW4568 and transformed in JDW2809.	This study

Strain number	Genotype	Construction details	Source
JDW4398	*B. subtilis* NCIB 3610 *comI* (Q12L) *truA::kan*	Made by homologous recombination. PCR product of *truA::kan* (with 500bp flanking regions) was amplified from BKK01480 (BGSC) using oJW4581+oJW4582 and transformed in JDW2809.	This study
JDW4399	*B. subtilis* NCIB 3610 *comI* (Q12L) *queA::kan*	Made by homologous recombination. PCR product of *queA::kan* (with 500bp flanking regions) was amplified from BKK27720 (BGSC) using oJW4569+oJW4570 and transformed in JDW2809.	This study
JDW4400	*B. subtilis* NCIB 3610 comI(Q12L) *spo0A::kan*	Made by homologous recombination. PCR product of *spo0A::kan* (with 500bp flanking regions) was amplified from BKK24220 (BGSC) using oJW4585+oJW4586 and transformed in JDW2809.	This study
JDW4401	*B. subtilis* NCIB 3610 comI(Q12L) Δ*mnmE* *spo0A::kan*	Made by homologous recombination. PCR product of *spo0A::kan* (with 500bp flanking regions) was amplified from BKK24220 (BGSC) using oJW4585+oJW4586 and transformed in JDW4304.	This study
JDW4402	*B. subtilis* NCIB 3610 comI(Q12L) Δ*mnmG* *spo0A::kan*	Made by homologous recombination. PCR product of *spo0A::kan* (with 500bp flanking regions) was amplified from BKK24220 (BGSC) using oJW4585+oJW4586 and transformed in JDW4305.	This study
JDW4428	*B. subtilis* NCIB 3610 *comI* (Q12L) Δ*mnmE* Δ*mnmG*	Made by homologous recombination. PCR product 1 containing 500nt upstream of *mnmE* deletion and the BKK kan cassette was amplified from BKK41020 genomic DNA using oJW4384+oJW4608. PCR product 2 containing 500nt downstream of *mnmG* deletion and the BKK kan cassette was amplified from BKK41010 genomic DNA using oJW4607+oJW4386. Overlap PCR with oJW4384+oJW4386 on fragment 1+2 was used to amplify the final PCR product that was later transformed in JDW4305. In a second step, cassette was looped-out using pDR244 to get a marker-less Δ*mnmEG* strain.	This study
JDW4446	*B. subtilis* NCIB 3610 comI(Q12L) Δ*mnmE* *relA*(D264G)*	Made by CRISPR/Cas9 editing: multimerized pJW840 was used to edit *relA* ORF towards synthetase dead *relA** D264G in JDW4304.	This study
JDW4463	*B. subtilis* NCIB 3610 comI(Q12L) *remA::kan*	Made by homologous recombination. PCR product of *remA::kan* (with 500bp flanking regions) was amplified from BKK15670 (BGSC) using oJW4598+oJW2379 and transformed in JDW2809.	This study
JDW4464	*B. subtilis* NCIB 3610 comI(Q12L) Δ*mnmE* *remA::kan*	Made by homologous recombination. PCR product of *remA::kan* (with 500bp flanking regions) was amplified from BKK15670 (BGSC) using oJW4598+oJW2379 and transformed in JDW4304.	This study
JDW4465	*B. subtilis* NCIB 3610 comI(Q12L) Δ*mnmG* *remA::kan*	Made by homologous recombination. PCR product of *remA::kan* (with 500bp flanking regions) was amplified from BKK15670 (BGSC) using oJW4598+oJW2379 and transformed in JDW4305.	This study
JDW4466	*B. subtilis* NCIB 3610 comI(Q12L) *remB::kan*	Made by homologous recombination. PCR product of *remB::kan* (with 500bp flanking regions) was amplified from BKK00050 (BGSC) using oJW4599+oJW4600 and transformed in JDW2809.	This study
JDW4467	*B. subtilis* NCIB 3610 comI(Q12L) Δ*mnmE* *remB::kan*	Made by homologous recombination. PCR product of *remB::kan* (with 500bp flanking regions) was amplified from BKK00050 (BGSC) using oJW4599+oJW4600 and transformed in JDW4304.	This study
JDW4468	*B. subtilis* NCIB 3610 comI(Q12L) Δ*mnmG* *remB::kan*	Made by homologous recombination. PCR product of *remB::kan* (with 500bp flanking regions) was amplified from BKK00050 (BGSC) using oJW4599+oJW4600 and transformed in JDW4305.	This study

Strain number	Genotype	Construction details	Source
JDW4599	B. subtilis NCIB 3610 comI(Q12L) Δ*bmrCD*	Made by homologous recombination: PCR product for *bmrCD::kan* (with 500bp flanking regions) was obtained by 2-fragment overlapping PCR with oJW4839+oJW4840. The upper fragment containing 500bp homology region upstream of *bmrC* and the BKK antibiotic cassette was amplified from BKK09710 (BGSC) using oJW4839+oJW4608. The lower fragment containing the 500bp homology region downstream of *bmrD* was amplified from BKK09720 (BGSC) using oJW4840+oJW4607. The overlap PCR product was transformed into JDW2809 and in a second step, cassette was looped-out using pDR244 to get a marker-less Δ*bmrCD* strain.	This study
JDW4600	B. subtilis NCIB 3610 comI(Q12L) Δ*mnmG* Δ*bmrCD*	Made by homologous recombination: PCR product for *bmrCD::kan* (with 500bp flanking regions) was obtained by 2-fragment overlapping PCR with oJW4839+oJW4840. The upper fragment containing 500bp homology region upstream of *bmrC* and the BKK antibiotic cassette was amplified from BKK09710 (BGSC) using oJW4839+oJW4608. The lower fragment containing the 500bp homology region downstream of *bmrD* was amplified from BKK09720 (BGSC) using oJW4840+oJW4607. The overlap PCR product was transformed into JDW4305 and in a second step, cassette was looped-out using pDR244 to get a marker-less Δ*bmrCD* strain.	This study
JDW4607	B. subtilis NCIB 3610 comI(Q12L) Δ*mnmE* Δ*bmrCD*	Made by homologous recombination: PCR product for *bmrCD::kan* (with 500bp flanking regions) was obtained by 2-fragment overlapping PCR with oJW4839+oJW4840. The upper fragment containing 500bp homology region upstream of *bmrC* and the BKK antibiotic cassette was amplified from BKK09710 (BGSC) using oJW4839+oJW4608. The lower fragment containing the 500bp homology region downstream of *bmrD* was amplified from BKK09720 (BGSC) using oJW4840+oJW4607. The overlap PCR product was transformed into JDW4304 and in a second step, cassette was looped-out using pDR244 to get a marker-less Δ*bmrCD* strain.	This study
JDW4825	*B. subtilis* NCIB 3610 *comI* (Q12L) *queF::kan*	Made by homologous recombination. PCR product of *queF::kan* (with 500bp flanking regions) was amplified from BKK13750 (BGSC) using oJW4958+oJW4959 and transformed in JDW2809.	This study
JDW4827	*B. subtilis* NCIB 3610 *comI* (Q12L) *tgt::kan*	Made by homologous recombination. PCR product of *tgt::kan* (with 500bp flanking regions) was amplified from BKK27710 (BGSC) using oJW4956+oJW4957 and transformed in JDW2809.	This study
JDW4949	*B. subtilis* NCIB 3610 *comI* (Q12L) Δ*mnmE* Δ*B4U62_20205*	Made by CRISPR/Cas9 editing: multimerized pJW893 was used to delete region containing predicted ORF *B4U62_20205* in JDW4304.	This study
JDW4950	*B. subtilis* NCIB 3610 *comI* (Q12L) Δ*B4U62_20205*	Made by CRISPR/Cas9 editing: multimerized pJW893 was used to delete region containing predicted ORF *B4U62_20205* in JDW2809.	This study
JDW4972	*B. subtilis* NCIB 3610 *comI* (Q12L) Δ*mnmE* *amyE::Pspank-mnmE(C456S) ycgO::PtapA-sfGFP(Cm)*	Made by homologous recombination. First, pJW783 was integrated at the *amyE* locus in JDW4304. Then, the biofilm reporter and flanking regions was amplified by PCR using oJW4515+oJW4516 (on genomic DNA from JDW3928) and transformed.	This study
JDW4984	*B. subtilis* NCIB 3610 *comI* (Q12L) Δ*mnmE* *ytqA::kan*	Made by homologous recombination. PCR product of *ytqA::kan* (with 500bp flanking regions) was amplified from BKK30480 (BGSC) using oJW5043+oJW5044 and transformed in JDW4304.	This study

Strain number	Genotype	Construction details	Source
JDW4985	*B. subtilis* NCIB 3610 *comI* (Q12L) *ytqB::kan*	Made by homologous recombination. PCR product of *ytqB::kan* (with 500bp flanking regions) was amplified from BKK30490 (BGSC) using oJW5045+oJW5046 and transformed in JDW2809.	This study
JDW5032	*B. subtilis* NCIB 3610 *comI* (Q12L) *ytqA::kan*	Made by homologous recombination. PCR product of *ytqA::kan* (with 500bp flanking regions) was amplified from BKK30480 (BGSC) using oJW5043+oJW5044 and transformed in JDW2809.	This study
JDW5033	*B. subtilis* NCIB 3610 *comI* (Q12L) Δ*mnmE* *ytqB::kan*	Made by homologous recombination. PCR product of *ytqB::kan* (with 500bp flanking regions) was amplified from BKK30490 (BGSC) using oJW5045+oJW5046 and transformed in JDW4304.	This study
JDW5034	*B. subtilis* NCIB 3610 *comI* (Q12L) Δ*mnmE* *ycgO::PtapA-sfGFP(Cm)*	Made by homologous recombination. The biofilm reporter and flanking regions was amplified by PCR using oJW4515+oJW4516 (on genomic DNA from JDW3928) and transformed into JDW4304.	This study
JDW5045	*B. subtilis* NCIB 3610 *comI* (Q12L) *ycgO::PtapA-sfGFP(Cm) relA*(D264G)*	Made by CRISPR/Cas9 editing: multimerized pJW840 was used to edit *relA* ORF towards synthetase dead *relA** D264G in JDW4351.	This study
JDW5046	*B. subtilis* NCIB 3610 *comI* (Q12L) Δ*mnmE* *ycgO::PtapA-sfGFP(Cm) relA*(D264G)*	Made by CRISPR/Cas9 editing: multimerized pJW840 was used to edit *relA* ORF towards synthetase dead *relA** D264G in JDW5034.	This study
JDW5216	B. subtilis NCIB 3610 comI(Q12L) Δ*mnmE* *amyE::PabrB(wt)-GFPmut2(A206K) abrB::kan*	Made by homologous recombination: linearized plasmid pJW902 was integrated at *amyE* locus of JDW4304. Then, *abrB* was deleted by transforming a PCR product of abrB::kan (with 500bp flanking regions) amplified from BKK00370 (BGSC) using oJW4527+oJW4528.	This study
JDW5217	B. subtilis NCIB 3610 comI(Q12L) *amyE::PabrB(wt)-GFPmut2(A206K) abrB::kan*	Made by homologous recombination: linearized plasmid pJW902 was integrated at *amyE* locus of JDW2809. Then, *abrB* was deleted by transforming a PCR product of abrB::kan (with 500bp flanking regions) amplified from BKK00370 (BGSC) using oJW4527+oJW4528.	This study
JDW5218	B. subtilis NCIB 3610 comI(Q12L) Δ*mnmE* *amyE::PabrB(2xAAA)-GFPmut2(A206K) abrB::kan*	Made by homologous recombination: linearized plasmid pJW907 was integrated at *amyE* locus of JDW4304. Then, *abrB* was deleted by transforming a PCR product of abrB::kan (with 500bp flanking regions) amplified from BKK00370 (BGSC) using oJW4527+oJW4528.	This study
JDW5219	B. subtilis NCIB 3610 comI(Q12L) *amyE::PabrB(2xAAA)-GFPmut2(A206K) abrB::kan*	Made by homologous recombination: linearized plasmid pJW907 was integrated at *amyE* locus of JDW2809. Then, *abrB* was deleted by transforming a PCR product of abrB::kan (with 500bp flanking regions) amplified from BKK00370 (BGSC) using oJW4527+oJW4528.	This study
JDW5220	B. subtilis NCIB 3610 comI(Q12L) Δ*mnmE* *amyE::PabrB(2xAAG)-GFPmut2(A206K) abrB::kan*	Made by homologous recombination: linearized plasmid pJW906 was integrated at *amyE* locus of JDW4304. Then, *abrB* was deleted by transforming a PCR product of abrB::kan (with 500bp flanking regions) amplified from BKK00370 (BGSC) using oJW4527+oJW4528.	This study
JDW5221	B. subtilis NCIB 3610 comI(Q12L) *amyE::PabrB(2xAAG)-GFPmut2(A206K) abrB::kan*	Made by homologous recombination: linearized plasmid pJW906 was integrated at *amyE* locus of JDW2809. Then, *abrB* was deleted by transforming a PCR product of abrB::kan (with 500bp flanking regions) amplified from BKK00370 (BGSC) using oJW4527+oJW4528.	This study
JDW5222	B. subtilis NCIB 3610 comI(Q12L) Δ*mnmE* *amyE::PabrB(noLP)-GFPmut2(A206K) abrB::kan*	Made by homologous recombination: linearized plasmid pJW904 was integrated at *amyE* locus of JDW4304. Then, *abrB* was deleted by transforming a PCR product of abrB::kan (with 500bp flanking regions) amplified from BKK00370 (BGSC) using oJW4527+oJW4528.	This study

Strain number	Genotype	Construction details	Source
JDW5223	B. subtilis NCIB 3610 comI(Q12L) *amyE::PabrB(noLP)-GFPmut2(A206K) abrB::kan*	Made by homologous recombination: linearized plasmid pJW904 was integrated at *amyE* locus of JDW2809. Then, *abrB* was deleted by transforming a PCR product of abrB::kan (with 500bp flanking regions) amplified from BKK00370 (BGSC) using oJW4527+oJW4528.	This study

Table S2. Plasmid list

Plasmid number	Description	Construction details	Source
pDR110	*amyE::Pspank* (amp, spec)	Integration vector for *amyE* locus with IPTG-controlled promoter (*Pspank*).	David Rudner
pDR244	*pDR244-cre* +ts origin	Temperature-sensitive plasmid with constitutively expressed Cre recombinase.	PMID: 28189581
pJW269	*pLIC- trpC_HA*	Expression vector	PMID: 12071693
pJW756	*pLIC- trpC_HA-Bs_mnmE* (amp)	Expression vector for full-length *B. subtilis* MnmE made by cloning *mnmE* coding region (amplified using oJW4360 and oJW4361) into pJW269 using ligation independent cloning.	This study
pJW763	*pLIC- trpC_HA-Bs_mnmG* (amp)	Expression vector for full-length *B. subtilis* MnmG made by cloning *mnmG* coding region (amplified using oJW4387 and oJW4388) into pJW269 using ligation independent cloning.	This study
pJW764	*pLIC- trpC_HA-Bs_mnmE(D275A)* (amp)	Expression vector for *B. subtilis* MnmE(D275A) point mutant. Obtained by site-directed mutagenesis (megaprimer method) of pJW756 using oJW4409 and oJW492 to introduce the GAT>GCT mutation.	This study
pJW765	*pLIC- trpC_HA-Bs_mnmE(E287A)* (amp)	Expression vector for *B. subtilis* MnmE(E287A) point mutant. Obtained by site-directed mutagenesis (megaprimer method) of pJW756 using oJW4410 and oJW492 to introduce the GAG>GCG mutation.	This study
pJW766	*pLIC- trpC_HA-Bs_mnmE(C456S)* (amp)	Expression vector for *B. subtilis* MnmE(C456S) point mutant. Obtained by site-directed mutagenesis (megaprimer method) of pJW756 using oJW4411 and oJW2596 to introduce the TGT>TCT mutation.	This study
pJW767	*pLIC- trpC_HA-Bs_mnmE(D338N)* (amp)	Expression vector for *B. subtilis* MnmE(D338N) point mutant. Obtained by site-directed mutagenesis (megaprimer method) of pJW756 using oJW4412 and oJW492 to introduce the GAC>AAC mutation.	This study
pJW772	*pLIC- trpC_HA-Bs_mnmE(G233A)* (amp)	Expression vector for *B. subtilis* MnmE(G233A) point mutant. Obtained by site-directed mutagenesis (megaprimer method) of pJW756 using oJW4408 and oJW492 to introduce the GGA>GCA mutation.	This study
pJW773	*pLIC- trpC_HA-Bs_mnmE-Gdomain* (amp)	Expression vector for *B. subtilis* MnmE G-domain made by cloning the *mnmE* coding region for residues G221 to Y380 with added ATG and STOP codons (amplified using oJW4417 and oJW4415) into pJW269 using ligation independent cloning.	This study
pJW776	*pDR110-Pspank-mnmE* (amp,spec)	Integration vector for amyE locus with IPTG-controlled *mnmE* expression. The *mnmE* ORF and a standard RBS was amplified using oJW4453 and oJW4454, and cloned into pDR110 using restriction enzyme cloning (with SalI and SphI).	This study
pJW777	*pDR110-Pspank-mnmE(D338N)* (amp,spec)	Integration vector for amyE locus with IPTG-controlled *mnmE(D338N)* expression. The *mnmE(D338N)* ORF and a standard RBS was amplified from pJW767 using oJW4453 and oJW4454, and cloned into pDR110 using restriction enzyme cloning (with SalI and SphI).	This study

Plasmid number	Description	Construction details	Source
pJW778	*pDR110-Pspank-mnmE(G233A)* (amp,spec)	Integration vector for amyE locus with IPTG-controlled *mnmE(G233A)* expression. The *mnmE(G233A)* ORF and a standard RBS was amplified from pJW772 using oJW4453 and oJW4454, and cloned into pDR110 using restriction enzyme cloning (with SalI and SphI).	This study
pJW778	*pDR110-Pspank-mnmE(G233A)* (amp,spec)	Integration vector for amyE locus with IPTG-controlled *mnmE(G233A)* expression. The *mnmE(G233A)* ORF and a standard RBS was amplified from pJW772 using oJW4453 and oJW4454, and cloned into pDR110 using restriction enzyme cloning (with SalI and SphI).	This study
pJW783	*pDR110-Pspank-mnmE(C456S)* (amp,spec)	Integration vector for amyE locus with IPTG-controlled *mnmE(C456S)* expression. The *mnmE(C456S)* ORF and a standard RBS was amplified from pJW766 using oJW4453 and oJW4454, and cloned into pDR110 using restriction enzyme cloning (with SalI and SphI).	This study
pJW840	*pPB41-gRNA(relA)-relA*D264G* (amp, spec)	CRISPR editing plasmid for editing *relA* into *relA(D264G)* . Plasmid assembled using the Golden Gate method with: the pPB41 backbone (amplified by PCR with oJW2775 and oJW2821); the guide RNA template (annealed oJW2927 and oJW2928) and the *relA(D264G)* repair template (obtained by fusion PCR of oJW2768+oJW2926 and oJW2769+oJW2925).	This study
pJW893	*pPB41-gRNA(B4U62_20205)-* Δ*B4U62_20205* (amp, spec)	CRISPR editing plasmid for Δ*B4U62_20205* deletion. Plasmid assembled using the Golden Gate method with: the pPB41 backbone (amplified by PCR with oJW2775 and oJW2821); the guide RNA template (annealed oJW4999 and oJW5000) and the Δ*B4U62_20205* repair template (as a synthetic gene fragment).	This study
pJW902	*pDR110-PabrB(wt)-GFPmut2(A206K)* (amp, spec)	Integration vector for amyE locus with *PabrB(wt)-GFPmut2(A206K)* reporter. *PabrB(wt)-GFPmut2(A206K)* was ordered as a synthetic gene fragment, and cloned into pDR110 using Ligase Chain Reaction (LCR) excluding the *Pspank* region.	This study
pJW904	*pDR110-PabrB(noLP)-GFPmut2(A206K)* (amp,spec)	Integration vector for amyE locus with *PabrB(noLP)-GFPmut2(A206K)* reporter. *PabrB(noLP)-GFPmut2(A206K)* reporter containing a mutation of the START codon of the leader peptide ORF(ATG to TAG) was ordered as a synthetic gene fragment, and cloned into pDR110 using Ligase Chain Reaction (LCR) excluding the Pspank region.	This study
pJW906	*pDR110-PabrB(2xAAG)-GFPmut2(A206K)* (amp, spec)	Integration vector for amyE locus with *PabrB(2xAAG)-GFPmut2(A206K)* reporter. Mutation (2xAAG) of the leader peptide ORF was introduced using site-directed mutagenesis of pJW902 with oJW5266.	This study
pJW907	*pDR110-PabrB(2xAAA)-GFPmut2(A206K)* (amp, spec)	Integration vector for amyE locus with *PabrB(2xAAA)-GFPmut2(A206K)* reporter. Mutation (2xAAA) of the leader peptide ORF was introduced using site-directed mutagenesis of pJW902 with oJW5267.	This study

Table S3. Primer and Synthetic DNA list

Primer number	Sequence	Purpose
oJW0492	GCTTTGTTAGCAGCCGGATCAG	Primer on pLIC backbone used for amplifying megaprimer for site-directed mutagenesis.
oJW2596	ATTCCGACAGCATCGCCAGTCACTA	Primer on pLIC backbone used for amplifying megaprimer for site-directed mutagenesis.
oJW2768	CAGGGTCTCACCTTATGGCGAACGAACAAGTATTGACTGC	Forward primer for cloning full-length *relA* with Golden Gate.
oJW2769	GTCGGTCTCACAGCTTAGTTCATGACGCGGCGCACAGAA	Reverse primer for cloning full-length *relA* with Golden Gate.
oJW2775	GACGGTCTCAGCTGGCTGTAGGCATAGGCTTGGTTATG	Forward primer to amplify pBR41 plasmid backbone for Golden Gate cloning.
oJW2821	GTAGGTCTCTAAGGATTTCGCGGGATCGAGATCCTGCATTAATG	Reverse primer to amplify pBR41 plasmid backbone for Golden Gate cloning.
oJW2925	GGTTTGCTTGCTGTCCGTATTCTTGTGAATAGCATAAAGGACTGCTAC	Forward primer for generating relA D264G mutation.
oJW2926	ATTCACAAGAATACGGACAGCAAGCAAACCGTAAATTTCATTGAATTGCTTATTTTGCAG	Reverse primer for generating relA D264G mutation.
oJW2927	AAACAATAAGCAATTCAATGAAATTTACGATTTGTG	Forward strand of the guide RNA for targeting the *relA* ORF with CRISPR.
oJW2928	AAAACACAAATCGTAAATTTCATTGAATTGCTTATT	Reverse strand of the guide RNA for targeting the *relA* ORF with CRISPR.
oJW4360	TACTTCCAATCCAATGCAATGGATACAATTGCGGCAATTTCCAC	Forward primer to amplify full-length *mnmE* for cloning into pLICtrPC-HA vector.
oJW4361	TTATCCACTTCCAATGTTATTATTTTCCTAAACAGAATTGAGAAAAGAG	Reverse primer to amplify full-length *mnmE* for cloning into pLICtrPC-HA vector.
oJW4383	CTTAAATATGTCCCTGTCGTCATGAC	Reverse primer for PCR amplification of mn*mE::kan* (with 500nt flanking regions).
oJW4384	CATCAAGCTTGCTGACCAGGTTTTG	Forward primer for PCR amplification of mn*mE::kan* (with 500nt flanking regions).
oJW4385	GAATCGGTGTTGAACGTTCTCGCCAG	Forward primer for PCR amplification of mn*mG::kan* (with 500nt flanking regions).
oJW4386	CTTCACCAGAGGCAAGCAAAGCTCACTC	Reverse primer for PCR amplification of mn*mG::kan* (with 500nt flanking regions).
oJW4387	TACTTCCAATCCAATGCAATGGGGTATGAAGCAGGCCAATAC	Forward primer to amplify full-length *mnmG* for cloning into *pLIC- trpC_HA*.
oJW4388	TTATCCACTTCCAATGTTATTACTCGGCTATCTTCGCAATGCGTC	Reverse primer to amplify full-length *mnmG* for cloning into *pLIC- trpC_HA* .
oJW4408	CATTATCGGCCGGCCAAACGTAGCAAAATCATCTCTTCTGAAC	For making megaprimer for G233A site-directed mutagenesis of *mnmE* ORF.
oJW4409	CTTCGTCTGGTTGCTACAGCAGGTATTC	For making megaprimer for D275A site-directed mutagenesis of *mnmE* ORF.
oJW4410	GAGGATATTGTTGCGCGAATCGGTGTTG	For making megaprimer for E287A site-directed mutagenesis of *mnmE* ORF.
oJW4411	CTCTTTTCTCAATTCTCTTTAGGAAAATAATAACATTGGAAGTGG	For making megaprimer for C456S site-directed mutagenesis of *mnmE* ORF.
oJW4412	GTGATTTTGAATAAGACTAACCTTGAGCCGAAG	For making megaprimer for D338N site-directed mutagenesis of *mnmE* ORF.
oJW4415	TTATCCACTTCCAATGTTATTAGTAGAACAGTGATTGAATCGCTTCTTC	Reverse primer for amplifying MnmE G-domain into pLIC- trpC_HA.
oJW4417	TACTTCCAATCCAATGCAATGGGTCTTTCTACCGTCATTATC	Forward primer for amplifying MnmE G-domain into pLIC- trpC_HA.
oJW4453	ACATGTCGACAAAGGAGGTGTACATATGGATACAATTGCGGCAATTTCCAC	PCR amplification of mn*mE* ORF with standard RBS for cloning into pDR110.
oJW4454	ACATGCATGCTTATTTTCCTAAACAGAATTGAGAAAAG	PCR amplification of mn*mE* ORF with standard RBS for cloning into pDR110.
oJW4515	ATTGTGACGGGTGTTGTTGTGGCAT	PCR amplification of the *ycgO::PtapA-sfGFP* reporter and flanking regions for homologous recombination.
oJW4516	CCTCCTCCTTTCGATCTTTATTGCGAC	PCR amplification of the *ycgO::PtapA-sfGFP* reporter and flanking regions for homologous recombination.
oJW4567	GTCCATGACTTTGCCCTGATATTC	PCR amplification of *trmR* locus with flanking regions for homologous recombination.
oJW4568	CATAAGCTGCTGACAATGGCTTC	PCR amplification of *trmR* locus with flanking regions for homologous recombination.
oJW4569	GTACAATACCAAACAGCCCTTG	PCR amplification of *queA* locus with flanking regions for homologous recombination.
oJW4570	GAGGTTGAGATTGATAAACCGTC	PCR amplification of *queA* locus with flanking regions for homologous recombination.
oJW4571	CATAGAGGAGCGAATGGCTTTC	PCR amplification of *cspR* locus with flanking regions for homologous recombination.
oJW4572	CTTCTGCAAGGCTGACATTTC	PCR amplification of *cspR* locus with flanking regions for homologous recombination.
oJW4575	GTGAACACATTGGGCCTGTATC	PCR amplification of *miaA* locus with flanking regions for homologous recombination.
oJW4576	GTTCAAGTGGAAACGAGGTG	PCR amplification of *miaA* locus with flanking regions for homologous recombination.
oJW4581	GACACATGAAGGACCGATTATC	PCR amplification of *truA* locus with flanking regions for homologous recombination.
oJW4582	GATGCTCAGAACCACGGTATAC	PCR amplification of *truA* locus with flanking regions for homologous recombination.
oJW4583	GTGCCTCCTGTGTTAAATCATG	PCR amplification of *rsmG* locus with flanking regions for homologous recombination.
oJW4584	GAAATCGGACATCGCATTGGATTG	PCR amplification of *rsmG* locus with flanking regions for homologous recombination.
oJW4607	ATAACTTCGTATAGCATACATTATACGAACGGTAGAGAGAGCACAGATAC	PCR amplification of Kan cassette from BKK strain for overlapping PCR.
oJW4607	ATAACTTCGTATAGCATACATTATACGAACGGTAGAGAGAGCACAGATAC	For overlapping PCR of *bmrCD::kan* with flanking regions for homologous recombination.
oJW4608	GTATCTGTGCTCTCTCTACCGTTCGTATAATGTATGCTATACGAAGTTAT	PCR amplification of Kan cassette from BKK strain for overlapping PCR.
oJW4608	GTATCTGTGCTCTCTCTACCGTTCGTATAATGTATGCTATACGAAGTTAT	For overlapping PCR of *bmrCD::kan* with flanking regions for homologous recombination.
oJW4839	GATATAGCTTGCCTCAGCTGCGGGTC	For overlapping PCR of *bmrCD::kan* with flanking regions for homologous recombination.
oJW4840	GAGAATGCTGAAATCGTGATCAGCTGTCTAGGCAC	For overlapping PCR of *bmrCD::kan* with flanking regions for homologous recombination.
oJW4956	CTACAGCTGGCCTTCACTTC	PCR amplification of *tgt* locus with flanking regions for homologous recombination.
oJW4957	CAAGGAAAGAGGCTGGATG	PCR amplification of *tgt* locus with flanking regions for homologous recombination.
oJW4958	CTGCAGCTGGTGTGACTC	PCR amplification of *queF* locus with flanking regions for homologous recombination.
oJW4959	CTTGAAGCCAGTGGACTTGTGAG	PCR amplification of *queF* locus with flanking regions for homologous recombination.

Primer number	Sequence	Purpose
oJW4999	GACGGTCTCAAAACGACATGGAAAATCCCGAGCCGAAACAAGACTGTTTTAGAGACCGTC	Guide RNA for targeting the *B4U62_20205* locus with CRISPR.
oJW5000	GACGGTCTCTAAAACAGTCTTGTTTCGGCTCGGGATTTTCCATGTCGTTTTGAGACCGTC	Guide RNA for targeting the *B4U62_20205* locus with CRISPR.
oJW5266	GATTTTGTCGAATAATGACGAAAAAAAAATATAATTTAAACAAATAAG	For making megaprimer for 2xAAA site-directed mutagenesis of *PabrB* fluorescent reporter.
oJW5267	GATTTTGTCGAATAATGACGAAGAAGAATATAATTTAAACAAATAAG	For making megaprimer for 2xAAG site-directed mutagenesis of *PabrB* fluorescent reporter.

Identifier	Sequence
ΔB4U62_20205 repair template	CAGGGTCTCACCTTGATCCGACAGACGGACACGGCGGTTTTGGCGTAGGGGCACTCAGCCTTGATAACATT<br>TCTCCCGTATTCGTTGATGTAGAGGAAAAAGAAGCGTTCGTAGACATCGGCGCTATGCATGCCCGCAGCACC<br>GTTGAAAAAGGAATTAAATTTCTCCCGAATAAAGAAGAAGTGCCGAACGGAAAACCATACTGGCTTGTGTGGG<br>TAACGATTGACAGAAAAGAAGAGGGGCCTTATTACGCTGGTGTGACAGCCTGTGAAATGACGGTCGACAGAA<br>GCATCAGAAGAGGCTACAAGTCACTGCCTGAGCATGTGAATCTGATGGATAAATCCATGAAACGGAAAATCAT<br>CGTTGATAAAATGGACGACAGCTCGAAGCGTGTACTAGGCGAATTTCTGAAAAAGCATGATCAGGGCCTATG<br>GGACCGTTCCTCAGATGAGTTAAAAACGAGCCTGCTTCCAGAAAACAATtaaTTTATGAATTTTTTGTGAACTTTT<br>AAAGTATTTGAGATATTTTTCGTAAACATTGAGTAAACTGGTGATACAGAAATAGGACTGGATGGAAGCCGGTC<br>TTTTGTTTGTTTTGCGGGTTCTGCCCCGTAGCACACGTTTGCGGGTTCTGTCCCTTTCACAAAATATGTGTAA<br>TGCTTAGCGGCGCAGCCGGGACCTGATGAGTAGCCTGTTTCCGGATCAATGTACACACCGGTTACGCCTTT<br>TGGCGGCTTGAACGCGGTTTCCGGTTCGCCTTTGAGCGCGTCCTCCATGAAGTCCGCCCAAATGGTTTTAG<br>CATAGCTTTTTTCTTCGACCGAGTCGATGGTGCTGTTTTTGTCATAGCCGGTCCAAACACCGGCGGCCAGCT<br>TCGGATTGAAGCCGATCATCCAGCTGTCTGCGCTGGTGGTTCCTGATTTTCCGGCATACGTTCTTGTCAGG<br>CGGTTGGCTATCGTTCGCCCCGTAACCGACGTATATCCATTTAAATCGATATCAAACATGCCTGTCATCATACT<br>CGCTGTGACAAAAGCGGCTTTTTCATCAAGCACCTGTTTATGCTGATTCGGGTTTTCGTACAGCACGTGGCC<br>GGCTGCGTCCGTTACTCTTGAAATAAAAGTCGGTTCGCTGTGAGACCGAC
PabrB(noLP)-GFPmut2(A206K)	AACTAATTCTTACAATCAATAGTAAACAAAATGATTGACGATTATTGGAAACCTTGTTATGCTATGAAGGTAAGG<br>ATTTTGTCGAATATAGACGAAGAAAAATATAATTTAAACAAATAAGTATCTCTTGGGAGGAGAATGTTTATGGTT<br>TCTAAAGGTGAAGAATTGTTTACAGGTGTTGTTCCAATTTTGGTTGAATTGGATGGTGATGTTAATGGTCATAA<br>ATTTTCTGTTTCTGGTGAAGGTGAAGGTGATGCTACATACGGTAAATTGACATTGAAATTTATTTGTACAACTG<br>GTAAATTGCCAGTTCCTTGGCCAACATTGGTTACAACATTTGCTTATGGTTTGCAATGTTTTGCTCGTTATCCA<br>GATCACATGAAACAACATGATTTCTTTAAATCTGCTATGCCAGAAGGTTATGTTCAAGAACGTACAATCTTTTTC<br>AAGGATGATGGTAATTATAAGACACGTGCTGAGGTTAAGTTTGAAGGTGATACATTGGTTAATCGTATCGAATT<br>GAAGGGTATCGATTTTAAAGAAGATGGTAATATCTTGGGTCATAAATTGGAATATAATTATAATTCTCATAATGT<br>TTATATCATGGCTGATAAACAAAAGAACGGTATTAAAGTTAATTTTAAAATTCGTCATAATATTGAAGATGGTTCT<br>GTTCAATTGGCTGATCATTATCAACAAAATACACCAATTGGTGATGGTCCAGTTTTGTTGCCAGATAATCATTAT<br>TTGTCTACACAATCTAAATTGTCTAAAGATCCAAATGAAAAACGTGATCACATGGTTTTGTTGGAATTTGTTACA<br>GCTGCTGGTATTACACATGGTATGGATGAATTGTATAAATAA
PabrB(wt)-GFPmut2(A206K)	AACTAATTCTTACAATCAATAGTAAACAAAATGATTGACGATTATTGGAAACCTTGTTATGCTATGAAGGTAAGG<br>ATTTTGTCGAATAATGACGAAGAAAAATATAATTTAAACAAATAAGTATCTCTTGGGAGGAGAATGTTTATGGTT<br>TCTAAAGGTGAAGAATTGTTTACAGGTGTTGTTCCAATTTTGGTTGAATTGGATGGTGATGTTAATGGTCATAA<br>ATTTTCTGTTTCTGGTGAAGGTGAAGGTGATGCTACATACGGTAAATTGACATTGAAATTTATTTGTACAACTG<br>GTAAATTGCCAGTTCCTTGGCCAACATTGGTTACAACATTTGCTTATGGTTTGCAATGTTTTGCTCGTTATCCA<br>GATCACATGAAACAACATGATTTCTTTAAATCTGCTATGCCAGAAGGTTATGTTCAAGAACGTACAATCTTTTTC<br>AAGGATGATGGTAATTATAAGACACGTGCTGAGGTTAAGTTTGAAGGTGATACATTGGTTAATCGTATCGAATT<br>GAAGGGTATCGATTTTAAAGAAGATGGTAATATCTTGGGTCATAAATTGGAATATAATTATAATTCTCATAATGT<br>TTATATCATGGCTGATAAACAAAAGAACGGTATTAAAGTTAATTTTAAAATTCGTCATAATATTGAAGATGGTTCT<br>GTTCAATTGGCTGATCATTATCAACAAAATACACCAATTGGTGATGGTCCAGTTTTGTTGCCAGATAATCATTAT<br>TTGTCTACACAATCTAAATTGTCTAAAGATCCAAATGAAAAACGTGATCACATGGTTTTGTTGGAATTTGTTACA<br>GCTGCTGGTATTACACATGGTATGGATGAATTGTATAAATAA

Table S4. Ligand–protein interactions predicted by AlphaFold3. Residues interacting with guanine nucleotide ligands (GTP/GDP/ppGpp), magnesium ions, and formyl-tetrahydrofolate in the predicted *Bacillus subtilis* MnmE dimer structures. Interacting residues were defined as those containing at least one heavy atom within 4.0 Å of any ligand heavy atom. Residue numbering corresponds to the full-length MnmE sequence (UniProt accession: P25811). Residues are denoted by chain identifier followed by residue number (e.g., A235, B235). Interactions are reported separately for chains A and B and for each ligand instance in the dimeric complex. Common Interacting Residues (All AF3 Models) are residues that contact the specified ligand in all three predicted structures, whereas Model-Specific Interacting Residues are unique to the indicated AF3 model and absent in the other two conditions.

Ligand	Common Interacting Residues (All AF3 Models)	Model-Specific Interacting Residues		
		MnmE dimer:2P4G:2Mg:2FON	MnmE dimer:2GTP:2Mg:2FON	MnmE dimer:2GDP:2Mg:2FON
FON_1	A20, A76, A77, A78, A85, A124, B16, B52, B66, B68, B87, B88, B89	A48	N/A	N/A
FON_2	A16, A52, A66, A68, A87, A88, A89, B20, B76, B77, B78, B85, B124	N/A	N/A	N/A
MG_1	A235	A279	A256	A279
MG_2	B235	B279	B256, B275	B279
nucleotide_1	A230, A231, A232, A233, A234, A235, A236, A335, A336, A338, A339, A361, A362, A363	A229	A249, A250, A251, A252, A253, A254, A255, A256, A278	A229
nucleotide_2	B229, B230, B231, B232, B233, B234, B235, B236, B335, B336, B338, B339, B361, B362, B363	N/A	B249, B250, B251, B252, B253, B254, B255, B256, B278	N/A

Table S5. Genes differentially expressed between ΔmnmE and WT in transcriptome analyses

A. **Significantly differentially expressed genes (contrast of interest *ΔmnmE* - WT) in LB exponential phase:**

gene_name	logFC	logCPM	F	PValue	FDR
mnmE	-6.9184094	7.51741595	517.939277	4.31E-08	0.00019002
bmrD	2.51851192	6.38662973	146.762677	3.99E-06	0.00878126
bmrC	2.48802885	6.01246268	91.8592363	2.04E-05	0.02993255
B4U62_RS227	2.10020502	2.91074591	76.2644928	3.85E-05	0.04236853

Table S5. Genes differentially expressed between ΔmnmE and WT in transcriptome analyses

B. Significantly differentially expressed genes (contrast of interest *ΔmnmE* -WT) in MSgg, 8H timepoint:

gene_name	logFC	logCPM	F	PValue	FDR
mnmE	-6.8189127	6.9795558	434.812062	6.58E-08	0.00028975
bmrD	2.80052886	7.94732688	259.738298	3.58E-07	0.00075352
bmrC	2.66183675	7.72256732	235.776227	5.13E-07	0.00075352
cidB	1.40442274	6.03068161	142.433617	3.28E-06	0.00361268
epsL	1.17140655	6.99245197	108.653005	8.74E-06	0.00578461
epsM	1.15910394	7.08764268	108.427768	8.81E-06	0.00578461
B4U62_RS184	1.15090241	8.14946586	105.167997	9.83E-06	0.00578461
epsO	1.06245221	6.8086577	100.199883	1.17E-05	0.00578461
epsN	1.08135835	7.64844298	99.886515	1.18E-05	0.00578461
lrgB	2.27621766	4.44188504	90.972329	1.65E-05	0.00726502
lrgA	2.53578333	3.74735103	86.5426789	1.97E-05	0.00764936
B4U62_RS184	1.06899132	7.81904107	85.1649104	2.08E-05	0.00764936
azoRB	-1.3668323	5.94997695	71.1306475	3.92E-05	0.01313257
epsA	1.02078257	7.45348647	67.7939685	4.64E-05	0.01313257
yrzI	1.28054044	4.49779833	67.5694186	4.69E-05	0.01313257
mtlF	0.97503213	6.53077344	66.2583217	5.02E-05	0.01313257
epsE	1.06496045	6.81401703	66.0735238	5.07E-05	0.01313257
epsG	1.04212051	7.37138506	62.1659179	6.26E-05	0.01461046
epsB	0.9737682	7.57925341	62.0339349	6.30E-05	0.01461046
epsF	1.02154575	7.41520512	60.093101	7.03E-05	0.01548259
rocG	0.94737196	5.39270581	56.9057519	8.47E-05	0.01746626
comGD	1.58939264	3.42228054	56.4186263	8.72E-05	0.01746626
epsI	1.04047207	7.36514477	55.206716	9.39E-05	0.01789107
mgsR	-1.1474723	4.92280454	54.6079275	9.75E-05	0.01789107
epsH	1.0768984	6.97193042	50.24726	0.00012917	0.02275919
comGE	1.63063828	2.37670581	46.9891577	0.00016172	0.02739938
B4U62_RS114	-1.9377358	1.10141716	44.1954981	0.0001983	0.03235159
mtlA	0.67149105	7.62198495	42.1408249	0.00023206	0.03650793
yfkM	-0.6727484	5.94633833	39.4630388	0.00028777	0.04366802
wapA	-0.6653184	12.4953096	38.9638409	0.00029996	0.04366802
yfkJ	-0.8353804	4.69100926	38.3248765	0.00031653	0.04366802
B4U62_RS197	-1.0170021	2.65053542	38.1085131	0.00032241	0.04366802
ybxG	0.9226669	8.52326665	37.8115669	0.00033069	0.04366802
comGF	1.46368749	2.45811278	37.5899602	0.00033705	0.04366802
mtlD	0.95885182	7.61613688	36.7526458	0.00036252	0.04412317
comGB	1.52554912	4.20121304	36.720393	0.00036355	0.04412317
B4U62_RS115	-1.5048455	1.35716557	36.0973718	0.00038417	0.04412317
rocA	0.96935623	5.69501619	36.0604189	0.00038543	0.04412317
bmrU	-0.6905581	5.1058587	35.9101536	0.00039065	0.04412317
vmlR	0.9930021	5.58612493	35.598294	0.00040176	0.04424334
lysCA	-1.0719117	7.08870963	34.8902567	0.00042849	0.04603699
copZ	1.95377328	5.47847756	36.1715837	0.00045158	0.04736215

Table S5. Genes differentially expressed between ΔmnmE and WT in transcriptome analyses

cwlS	-0.6876758	7.72404641	33.8286102	0.00047295	0.04762413
mphK	0.62478363	6.61213582	33.7669872	0.0004757	0.04762413
B4U62_RS013	-1.2886748	3.49377009	33.256183	0.00049934	0.04887957

Table S5. Genes differentially expressed between ΔmnmE and WT in transcriptome analyses

C. Significantly differentially expressed genes (contrast of interest *ΔmnmE* -WT) in MSGA, 8H timepoint:

gene_name	logFC	logCPM	F	PValue	FDR
mnmE	-6.7850495	7.06497125	2286.17419	3.65E-11	1.61E-07
epsB	1.79477935	6.00891248	286.782035	1.41E-07	0.00031105
bmrC	1.18552263	6.95382439	191.820774	6.78E-07	0.00099556
B4U62_RS184	1.94076058	6.13729161	159.179973	1.39E-06	0.00153459
epsO	2.01196114	5.36897029	142.997102	2.10E-06	0.00167951
B4U62_RS184	1.96181935	6.43487114	138.249294	2.39E-06	0.00167951
bmrD	1.23424773	7.28735206	134.354338	2.67E-06	0.00167951
epsA	1.77807446	5.97803139	126.61529	3.35E-06	0.00184243
epsL	1.89237512	5.28828622	121.418247	3.92E-06	0.00192054
epsN	2.00793867	6.03630366	110.473372	5.61E-06	0.00221198
epsM	1.82496757	5.3523708	105.895116	6.58E-06	0.00221198
epsG	1.75977765	5.66404549	105.203114	6.75E-06	0.00221198
epsE	1.70641341	5.14188974	103.85817	7.08E-06	0.00221198
srfAC	1.12812068	10.9994625	101.697896	7.66E-06	0.00221198
srfAB1	1.1509224	10.5359	100.924257	7.89E-06	0.00221198
epsC	1.65139585	6.22708914	100.425997	8.03E-06	0.00221198
isp	1.17790453	6.24252475	79.2611605	1.94E-05	0.00502296
levF	-1.1666225	3.49095334	77.7682225	2.08E-05	0.00508822
pckA	0.73140173	5.98194148	75.0506598	2.37E-05	0.00545141
tasA	2.30709008	6.39438902	81.5561424	2.51E-05	0.00545141
srfAB2	1.11311743	11.7135432	71.2856649	2.86E-05	0.00545141
B4U62_RS230	1.1598131	9.60864235	71.0203007	2.90E-05	0.00545141
cydC	0.71618302	10.3433829	70.7023881	2.95E-05	0.00545141
epsD	1.71401652	5.83767621	70.560068	2.97E-05	0.00545141
epsF	1.68199692	5.64234037	69.5197225	3.14E-05	0.00552477
fadA	-1.4422898	5.28079467	68.4364823	3.32E-05	0.00562505
srfAA	1.06212708	11.7194784	63.7908557	4.28E-05	0.00656204
comS	1.11631628	7.26226652	63.7324832	4.30E-05	0.00656204
fadN	-1.5877027	6.24517308	63.793009	4.32E-05	0.00656204
pelA	0.71146242	6.56970093	62.8418476	4.52E-05	0.00664136
mtrA	-0.619072	7.10846512	57.7368151	6.13E-05	0.00871508
fadE	-1.502505	6.48855872	56.047946	6.87E-05	0.00946031
msmX	-0.7881886	5.01590968	53.7620515	7.91E-05	0.01055644
ilvN	0.61705194	8.44154657	49.6500984	0.00010475	0.01357152
tdk	-0.7336816	5.91386859	48.1093561	0.000117	0.01452159
ydaB	0.81753572	5.3334497	47.9144302	0.00011868	0.01452159
sipW	2.29615775	4.6393417	49.4622903	0.0001397	0.0157784
argC	0.71395482	10.6540206	45.2706414	0.00014465	0.01592983
lcfB	-0.8368531	4.63429273	43.80195	0.00016217	0.01742347
cydD	0.60524	10.4882797	42.5091437	0.00017984	0.01886137
epsI	1.92731638	5.6397094	46.0053235	0.00018572	0.01902561
walL	0.6904857	9.17612507	41.499784	0.00019533	0.0195547

Table S5. Genes differentially expressed between ΔmnmE and WT in transcriptome analyses

artR	0.60183786	9.77028727	41.167425	0.00020079	0.01965485
mhqA	-0.6630433	4.80998711	40.7082743	0.00020865	0.01998063
yczE	-0.5804687	5.99943142	39.6522491	0.00022825	0.02139248
licC	-1.6537477	3.43399113	34.3132815	0.00037165	0.03088897
etfB	-0.7512353	6.47027284	32.6098724	0.00044004	0.0346135
yfmG	0.61582063	7.68871903	32.0590541	0.00046546	0.03470069
yfhI	-0.7638994	5.71277547	31.4698309	0.00049473	0.03470069
ilvA	0.58319672	8.94352992	31.1946685	0.00050918	0.03470069
nprE	0.66291177	6.77753526	31.0428776	0.00051738	0.03470069
yvqJ	0.59794376	6.44012477	30.9965512	0.00051992	0.03470069
albG	0.66695777	8.25646362	30.6832294	0.00053748	0.03515955
frlB	-0.7350879	9.67772736	30.5915714	0.00054276	0.03515955
srfAD	1.01363891	8.62439781	29.7136551	0.00059976	0.03774219
ssuA	-2.7721072	0.46678524	29.4642225	0.00061328	0.0380494
kinB	0.62512033	6.67001344	29.3024187	0.00062432	0.03819644
levE	-1.0444568	3.18731535	28.9124992	0.00065197	0.03934144
frlO	-0.6449358	8.49101371	28.605934	0.00067479	0.04016805
mneP	-0.6413992	5.37036085	28.3318408	0.00069604	0.04051831
ssuC	-2.3311575	0.68259553	28.2936243	0.00069907	0.04051831
bglH	-0.8683951	4.9431196	27.4242435	0.00077265	0.04363511
levG	-1.3040949	3.75394822	26.8791795	0.00082376	0.04535835
slrR	1.94816544	2.71626209	26.5704529	0.00085857	0.04598318
epsH	1.76699476	5.28769983	28.7256315	0.00086579	0.04598318
azoRB	-0.6236246	6.85025206	26.4403604	0.00086801	0.04598318
aprE	1.13310981	8.20882199	27.0563183	0.00087748	0.04598318
albF	0.62305265	9.10402574	26.2576828	0.0008873	0.04598318
sacC	-1.290201	2.72464801	25.9527021	0.00092073	0.0471604
levD	-1.276472	2.35606985	25.6229952	0.00095865	0.04769249
ybfI	-0.6891959	5.48125761	25.5139436	0.00097162	0.04769249
fadR	-0.8169156	7.09789087	25.155083	0.00101588	0.04769249
argJ	0.77317442	10.8235193	25.1182172	0.00102057	0.04769249
hom	0.6748601	10.7931129	25.0858074	0.00102471	0.04769249

Table S5. Genes differentially expressed between ΔmnmE and WT in transcriptome analyses

D. Significantly differentially expressed genes (contrast of interest *ΔmnmE* -WT) in MSgg, 24H timepoint:

gene_name	logFC	logCPM	F	PValue	FDR
epsB	5.75075103	7.53534481	1640.7419	2.37E-10	8.33E-07
epsN	6.33649736	7.47467735	1454.37774	3.78E-10	8.33E-07
epsA	6.07165941	7.46553519	1041.34714	1.38E-09	1.92E-06
sipW	7.62811978	7.47711107	950.835747	1.96E-09	1.92E-06
mnmE	-8.4431676	6.32612958	899.637217	2.43E-09	1.92E-06
epsM	6.35813284	6.81270738	848.528949	3.05E-09	1.92E-06
epsG	6.89606854	7.05537524	808.027818	3.69E-09	1.92E-06
epsF	6.39038632	7.13994278	805.932397	3.72E-09	1.92E-06
B4U62_RS18	6.26592025	7.57250789	795.48527	3.92E-09	1.92E-06
epsO	6.68490579	6.89479679	759.759669	4.68E-09	2.06E-06
epsI	6.70276467	7.08661062	736.92564	5.26E-09	2.11E-06
epsC	5.50492122	8.02950474	680.884971	7.14E-09	2.62E-06
epsL	6.66534074	6.83613758	641.710698	8.98E-09	3.04E-06
B4U62_RS18	6.74422643	7.92939537	614.577064	1.06E-08	3.30E-06
epsD	5.73505094	7.55376721	605.634055	1.12E-08	3.30E-06
epsH	6.56074211	6.65618026	469.044118	3.00E-08	8.27E-06
tasA	7.81503833	9.33479391	470.418706	5.47E-08	1.42E-05
epsE	5.82501244	6.65943484	387.518814	6.25E-08	1.53E-05
bmrD	2.12864647	7.42122961	360.837995	8.21E-08	1.90E-05
tapA	7.72206949	6.8515539	395.66688	9.89E-08	2.11E-05
slrR	5.71748531	4.64464114	342.277636	1.00E-07	2.11E-05
yybF	-2.9618501	10.9109475	202.27123	7.41E-07	0.00014458
cadA	2.90692269	5.42199852	201.282554	7.55E-07	0.00014458
yxeO	2.53127495	4.04683452	193.053334	8.83E-07	0.00016215
bmrC	1.90568369	7.13067338	185.304676	1.03E-06	0.0001816
yxeN	2.59108797	4.47115491	176.959588	1.23E-06	0.0002076
yjnA	1.90784126	5.03778929	154.000453	2.06E-06	0.00033628
trpD	2.23867146	7.00586891	150.981549	2.22E-06	0.00034912
sndB	2.14791495	5.01560833	145.923721	2.52E-06	0.00038271
bsrB	-2.1093553	6.05859521	140.588392	2.89E-06	0.00042492
yxeQ	2.06419695	4.62297708	138.848596	3.03E-06	0.00043069
trpE	2.13417886	8.18332501	137.58024	3.14E-06	0.00043169
kinB	1.46805045	6.87903025	133.319783	3.52E-06	0.00047042
flgB	-1.3945263	7.78964503	124.196881	4.58E-06	0.00059351
yxeK	1.93910322	4.18901816	117.920489	5.55E-06	0.00069807
ureC	1.27271518	7.59630256	115.811735	5.93E-06	0.00072529
gltB	1.47967085	10.928128	113.950218	6.29E-06	0.00074902
flgC	-1.3495458	7.8914146	112.137812	6.67E-06	0.00075851
trpC	2.1497668	7.0193751	111.944872	6.72E-06	0.00075851
yxeM	2.24286467	4.17064706	107.297284	7.85E-06	0.00086399
bsrA	-1.4154042	7.87496971	106.477215	8.07E-06	0.00086693
tcyP	2.07476937	6.17539552	104.675102	8.59E-06	0.00090084

Table S5. Genes differentially expressed between ΔmnmE and WT in transcriptome analyses

ycnD	1.47899752	6.44561636	102.183391	9.38E-06	0.00092777
azoRB	-1.2890653	5.8842841	101.845241	9.49E-06	0.00092777
fliF	-1.3247471	9.48633653	101.838713	9.50E-06	0.00092777
cysK	1.07279035	8.99288311	101.281056	9.69E-06	0.00092777
dltD	1.22498168	9.09467473	96.2775152	1.17E-05	0.00108192
trpF	2.03285249	7.34622146	95.9693629	1.18E-05	0.00108192
abrB	-1.7301306	5.58519264	92.8184535	1.33E-05	0.00117893
rocA	1.9372844	6.3770235	92.6800769	1.34E-05	0.00117893
cysH	1.15030544	8.4829361	89.9422614	1.49E-05	0.0012885
tcyB	1.45891196	5.87731425	88.761957	1.56E-05	0.00132561
ycnE	1.46062377	4.82621739	87.4219216	1.65E-05	0.00136964
hypO	3.04217655	9.26224477	99.8641466	1.68E-05	0.00136964
tcyC	1.61145184	5.06209845	84.7804068	1.85E-05	0.00147921
hxlB	-1.8717948	5.28500381	83.2411187	1.97E-05	0.00151195
B4U62_RS23	-1.2125008	6.54931619	83.0985869	1.99E-05	0.00151195
pyrD	-3.7431564	6.35743652	102.654353	1.99E-05	0.00151195
ydeQ	1.49251971	4.69472165	81.3302237	2.14E-05	0.00160134
fliE	-1.4900783	6.51952515	80.1525537	2.26E-05	0.00165934
ybfG	-1.2661396	9.97061716	77.8052485	2.51E-05	0.00181566
ytcJ	0.96664786	8.14118779	76.9449081	2.62E-05	0.00185027
dltB	1.05113827	9.47521251	76.7013501	2.65E-05	0.00185027
bglH	-1.5619448	6.69117902	73.3876568	3.10E-05	0.0021324
yhcV	2.25002565	4.82117168	73.0152094	3.15E-05	0.002138
cysP	1.22124549	8.34591846	72.6560693	3.21E-05	0.00214291
dltE	1.01836002	8.47891522	72.3339366	3.26E-05	0.00214458
licB	-2.2598534	4.57365952	71.0650155	3.47E-05	0.00222939
hutP	-1.4483923	4.37241211	70.9598644	3.49E-05	0.00222939
tcyA	1.13611444	6.18831697	70.4362807	3.59E-05	0.00225612
ffoR	-1.2018825	7.80455714	70.1491721	3.64E-05	0.00225683
yqzF	-1.4171789	6.40841122	69.4185507	3.78E-05	0.0023097
sufA	-1.1157348	6.15713132	66.8182677	4.32E-05	0.0026077
tlpA	1.18650521	6.65620579	65.7984755	4.56E-05	0.00271603
besA	-1.0578755	9.05934108	65.3550455	4.67E-05	0.00274451
ydjE	-1.0727039	7.94221034	64.9534577	4.78E-05	0.00276789
yxjH	-1.2621919	8.22616668	64.460236	4.91E-05	0.0028063
yodF	0.91543667	8.27004262	64.1178979	5.00E-05	0.00282275
yczG	-1.026713	6.74782923	63.4025622	5.20E-05	0.00286353
cydC	1.40238441	6.29398148	63.3991257	5.20E-05	0.00286353
nprE	1.117686	7.17886425	63.0573408	5.30E-05	0.00288243
bglP	-1.4775706	7.48290678	62.5331635	5.46E-05	0.00291809
trpB	1.76130492	7.41069371	62.4020907	5.50E-05	0.00291809
yecA	-1.9524911	3.41546341	61.8024543	5.69E-05	0.00295072
copA	1.15229923	5.47647053	61.7839758	5.69E-05	0.00295072
mmuP	1.42656564	6.68641231	59.4133031	6.53E-05	0.00334295
spoIISA	-0.9540466	6.33494789	59.2245445	6.60E-05	0.00334295

Table S5. Genes differentially expressed between ΔmnmE and WT in transcriptome analyses

opuCC	1.02178045	7.71117561	58.7403172	6.79E-05	0.00340104
fliI	-0.9679884	8.84593022	57.9590767	7.12E-05	0.00352353
narJ	5.30234351	5.13210927	77.1004862	7.37E-05	0.00356741
yhdX	-1.8182536	6.69195963	57.3860761	7.37E-05	0.00356741
glcT	-1.208221	6.13827855	56.5935215	7.73E-05	0.00366698
pyrF	-4.023673	5.91283493	75.411322	7.74E-05	0.00366698
gudB	-0.8891491	9.93607032	56.3159146	7.87E-05	0.00368716
ydeK	-1.1642973	4.46309487	55.7196412	8.16E-05	0.00378568
ppsE	-1.1584958	9.31682422	54.8382363	8.63E-05	0.00395913
cidA	2.41131982	3.77246532	54.607327	8.76E-05	0.00397599
yhjG	-1.1475379	11.281358	53.8360436	9.20E-05	0.00398474
ribT	-1.2934923	8.95137657	53.7767641	9.23E-05	0.00398474
guaD	-1.9874986	3.85720496	53.5348962	9.38E-05	0.00398474
mcpA	1.00964236	7.88421844	53.4208433	9.45E-05	0.00398474
snaB	2.02188938	3.0083901	53.3306991	9.50E-05	0.00398474
B4U62_RS21	-1.2462451	5.57219373	53.2785727	9.53E-05	0.00398474
yqgC	0.9978898	6.43506043	53.2496212	9.55E-05	0.00398474
ydzA	-1.2313078	6.80591672	53.1716825	9.60E-05	0.00398474
tagC	-1.1404386	6.21536216	53.1420537	9.62E-05	0.00398474
bmr3	1.17778104	5.93433522	53.045057	9.68E-05	0.00398474
opuCB	0.90317433	7.07028017	52.6738766	9.92E-05	0.00404463
ohaC	-0.8529415	7.78305975	52.1357442	0.00010274	0.00415185
samT	-1.5060296	8.91388778	51.9679667	0.00010388	0.00415358
mtlR	-1.2989435	6.42763418	51.8548344	0.00010467	0.00415358
fliG	-1.3139598	7.96414018	50.8187261	0.00011218	0.00437456
hxlA	-1.7606413	5.13981859	50.8140604	0.00011222	0.00437456
liaM	1.28462883	8.7966926	50.657275	0.00011342	0.00438243
proB	-1.0365439	5.82707238	50.5142489	0.00011452	0.00438667
csn	0.88528496	10.7161783	49.9832307	0.00011875	0.00446545
yhfQ	-0.9895573	8.89218482	49.970455	0.00011885	0.00446545
yybM	0.97393305	8.10094646	49.8765843	0.00011962	0.00446545
opuCD	0.92836553	7.31709513	49.426073	0.00012339	0.00454449
yybH	-1.4815277	4.74975289	49.378625	0.0001238	0.00454449
hemB	0.81123577	9.66760272	48.8692682	0.00012827	0.00466959
sirC	0.8794698	7.31759287	48.4818914	0.0001318	0.00475837
yqzM	-1.5756592	4.5077199	48.3677417	0.00013287	0.00475837
B4U62_RS10	-0.8999055	7.17285854	48.0139357	0.00013624	0.00480469
yfmI	1.06455883	6.77895746	47.9169397	0.00013718	0.00480469
pyrE	-4.0128147	5.78827333	62.7124848	0.00013761	0.00480469
cydD	1.27035312	6.74805273	47.7800437	0.00013852	0.00480469
spoVE	0.82479636	7.4670226	47.299079	0.00014338	0.00493426
uvrB	0.82243672	8.92479919	47.0614262	0.00014586	0.00498064
proA	-0.8140289	7.22647818	46.5434772	0.00015145	0.00513177
glcP	-4.6082983	8.59312537	74.2412188	0.00015371	0.00516865
dhbE	-0.881248	10.9311139	46.0736257	0.00015676	0.0052311

Table S5. Genes differentially expressed between ΔmnmE and WT in transcriptome analyses

yhbJ	1.14467835	4.26087145	45.3705763	0.00016514	0.00544222
sxzY	-1.0197551	8.52450061	45.337113	0.00016555	0.00544222
rsfS	0.85966329	5.84448276	45.0957806	0.00016857	0.00550035
ntdC	-5.2992177	10.1950842	70.9572299	0.00017305	0.00560498
yvpB	-1.4602696	5.60226162	44.1505816	0.00018107	0.00579696
artQ	1.11443825	7.55385616	44.111996	0.00018161	0.00579696
ylbP	-1.1023698	5.78612142	43.8661215	0.00018506	0.00586474
dltC	1.15949449	7.25384071	43.431873	0.00019137	0.00602126
skfH	0.97084526	7.61547181	43.1222884	0.00019603	0.00608308
yuiC	-0.7763327	6.15444684	43.0350122	0.00019737	0.00608308
cotN	1.78303569	5.25034003	43.028076	0.00019748	0.00608308
kapB	1.17719983	4.97185691	42.8506256	0.00020024	0.00611727
cyeB	1.66902264	7.33050248	44.1030696	0.00020136	0.00611727
pyc	0.75436171	10.3534112	42.5514542	0.000205	0.00618523
yvnB	0.94759523	9.43742009	42.4563228	0.00020655	0.00618946
narG	5.20750014	6.79229781	66.2299232	0.00021178	0.00630331
mphK	0.70251829	8.05698584	41.7977708	0.00021766	0.00643486
lutA	-0.9682715	7.01554713	41.5553732	0.00022194	0.00648725
sirB	1.05080213	8.07746491	41.4832073	0.00022323	0.00648725
phrH	-0.8206383	6.71685647	41.3761161	0.00022517	0.00648725
moaC	-0.7702321	6.40340112	41.3677246	0.00022532	0.00648725
narH	5.66769725	6.10937237	64.2190978	0.00023025	0.0065475
yfkAB	-0.7756655	6.45644467	41.0175933	0.00023181	0.0065475
conE	1.15216926	6.22818535	40.93685	0.00023334	0.0065475
ywpJ	0.90957583	6.90496238	40.9359422	0.00023336	0.0065475
yhcA	1.12762917	5.31911814	40.467331	0.0002425	0.00676076
spoVT	0.81910603	6.64038063	39.9467686	0.00025318	0.00696957
pyrAB	-3.9394006	7.7536467	58.3498637	0.00025379	0.00696957
sat	1.35733359	7.98575279	39.8431318	0.00025538	0.00696957
liaK	1.34933617	8.5545074	39.6263344	0.00026005	0.00696957
yhjH	-1.0496509	9.83887881	39.6062864	0.00026049	0.00696957
dhbC	-0.9900955	10.8110614	39.5162717	0.00026246	0.00696957
licA	-1.9441384	4.07265679	39.5083818	0.00026264	0.00696957
B4U62_RS23(	1.1769582	8.51333619	39.5081128	0.00026264	0.00696957
rocB	1.39798584	6.04944917	39.2677253	0.00026802	0.00706963
gltT	0.72794934	9.10262504	39.1817184	0.00026998	0.00707885
pelC	-1.301682	4.09695341	39.0790197	0.00027234	0.00709848
yhzE2	2.70338352	2.51038905	38.7547225	0.00027996	0.00725431
yjzC	-2.6150986	1.94869177	38.2634566	0.00029203	0.00752281
ntdB	-5.5351857	9.58685648	60.2943584	0.00029615	0.0075557
purH	-2.137395	8.08673121	44.5634003	0.00029748	0.0075557
nadB	-0.9599853	9.99952436	38.0123308	0.00029845	0.0075557
glpF	-1.0577171	7.25462484	37.7596905	0.0003051	0.0076797
appC	0.84587231	8.68388988	37.5713061	0.00031017	0.00776305
murD	0.76608551	7.8767587	37.4128997	0.00031452	0.00782742

Table S5. Genes differentially expressed between ΔmnmE and WT in transcriptome analyses

conG	1.18837221	5.86922674	37.3267409	0.00031692	0.00784277
pyrK	-4.1752055	5.87853632	50.9944296	0.00034134	0.00840001
ppaX	0.65866003	7.44771405	36.1468146	0.00035218	0.00852592
skfC	1.19478235	9.05190491	36.1465575	0.00035219	0.00852592
lysP	-1.1311481	5.99102967	36.1443144	0.00035226	0.00852592
B4U62_RS22(	1.58024313	5.84085499	36.0200181	0.00035626	0.00857555
amhX	-1.1337697	7.03750595	35.898075	0.00036024	0.00859957
ntdA	-5.6604582	10.1255556	55.9390005	0.00036116	0.00859957
yotL	-1.2889258	4.38088768	35.7844166	0.000364	0.00862042
nicK	1.33108537	6.5709262	35.6479019	0.00036857	0.00864971
ktrD	-0.8678223	6.27616997	35.6306044	0.00036916	0.00864971
dhbB	-0.8004856	10.322006	35.1924669	0.00038438	0.00889593
rlmA	-0.7662675	6.15850156	35.1900785	0.00038447	0.00889593
narI	5.53384413	5.46783169	53.0053447	0.00038573	0.00889593
yeeI	-0.7793875	7.31587455	35.0662476	0.00038891	0.00892273
gntR	-1.1787616	3.67041892	34.9909153	0.00039165	0.00893896
xlyA	-0.8482086	9.46998647	34.8027269	0.00039859	0.00893926
yjdB	1.63843479	11.1985059	35.7660209	0.00039864	0.00893926
yqeM	0.72291795	6.7753924	34.7938955	0.00039892	0.00893926
yfjA	-0.856908	4.99346531	34.7661234	0.00039996	0.00893926
B4U62_RS07	2.53027408	0.7116942	34.7169138	0.00040181	0.00893926
B4U62_RS10	1.56765957	3.71318992	34.6175774	0.00040558	0.00897732
feuA	-0.7411334	10.9481389	34.5329684	0.00040882	0.00897732
yvbW	1.18470167	5.38383868	34.4691078	0.00041129	0.00897732
appB	0.76045967	8.68176547	34.4391443	0.00041245	0.00897732
opuCA	0.71536555	8.28383656	34.4068256	0.00041371	0.00897732
aroE	0.70134656	6.58607519	34.3085366	0.00041758	0.00901676
dltA	0.77961041	9.62908973	34.248208	0.00041997	0.00902423
ybcC	4.09784991	7.57059304	50.7624268	0.00043279	0.00924884
acr3	1.2316523	3.96721959	33.8882253	0.00043462	0.00924884
gltD	1.22597012	8.96555244	33.7387257	0.0004409	0.00933724
treR	-0.7422361	5.78529848	33.3835825	0.00045626	0.00957736
motP	-0.9399169	4.28660048	33.3763526	0.00045658	0.00957736
spoIIIE	0.62477159	8.0271434	33.0247424	0.00047248	0.00981752
upp	-0.9026548	6.33335685	32.9495246	0.00047597	0.00981752
pnbA	1.00545663	8.35279065	32.9394134	0.00047644	0.00981752
asnO	2.94727179	3.0726129	33.8019726	0.00047788	0.00981752
B4U62_RS06(	1.65230975	4.0411369	32.881066	0.00047918	0.00981752
conQ	1.08593395	7.23245937	32.7297712	0.00048636	0.00989722
yvbU	1.07338477	4.45363517	32.7046938	0.00048756	0.00989722
murF	-0.7317649	7.90095287	32.5878909	0.00049321	0.00996606
ureB	1.3988436	4.4878272	32.5159719	0.00049674	0.0099914
B4U62_RS04	-0.6921127	7.08319519	32.2670354	0.00050917	0.01001388
yjdJ	0.85479776	5.34325812	32.250323	0.00051002	0.01001388
artR	0.89525838	7.76381836	32.2347082	0.00051082	0.01001388

Table S5. Genes differentially expressed between ΔmnmE and WT in transcriptome analyses

purD	-1.9057041	7.45298589	36.7485905	0.00051403	0.01001388
xkdA	1.37956314	3.78647559	32.117902	0.00051681	0.01001388
ppsC	-0.800659	10.6876273	32.0675363	0.00051943	0.01001388
dhbA	-0.9595921	10.0453682	32.0193758	0.00052194	0.01001388
uvrA	0.73873413	9.85268749	31.9982561	0.00052305	0.01001388
liaE	1.43064985	8.99025762	33.186246	0.00052324	0.01001388
aprE	2.64831713	8.30159265	41.0862622	0.00052387	0.01001388
yvbT	0.82252762	6.64888664	31.961698	0.00052497	0.01001388
ytzE	-1.0460066	7.61777101	31.9586163	0.00052513	0.01001388
nfo	0.71814553	7.09875896	31.8048882	0.00053332	0.01012621
ydhC	-0.8332522	4.84257581	31.7260526	0.00053758	0.01016199
pdp	0.70155003	8.02231614	31.6850047	0.00053982	0.01016199
cydB	0.98866425	5.76303019	31.5706319	0.00054611	0.0102367
lrgB	4.64247515	7.48891329	47.5571165	0.0005527	0.01031623
fliH	-1.1063676	7.63797779	31.357898	0.00055806	0.01037245
levR	-0.7368525	6.71336981	31.3137047	0.00056059	0.01037559
pbpG	1.69054915	2.3059917	31.1674368	0.00056904	0.01048792
skfB	1.42653958	8.22771753	32.4217013	0.00057266	0.01048792
kbl	-1.1395889	4.39419613	31.0863766	0.0005738	0.01048792
B4U62_RS23	3.23805253	3.06389557	32.9759198	0.00058271	0.01060673
ssbB	-0.8872666	5.37956581	30.8061155	0.00059063	0.01070673
nrdR	-0.9145626	6.11881398	30.7470328	0.00059426	0.01072834
licH	-2.286693	6.68432335	37.0273973	0.00059835	0.01073293
ridA	0.74814641	7.89650419	30.6642899	0.00059939	0.01073293
pepV	-0.7427402	7.51255955	30.6140161	0.00060253	0.01074552
B4U62_RS23	-1.0211058	6.3642739	30.374511	0.00061779	0.01097321
licC	-2.3424876	6.59982277	36.7963772	0.00062035	0.01097444
nadC	-0.8303841	9.25297052	30.2763328	0.00062418	0.01099811
calJ	0.72558822	7.66649471	30.2028815	0.00062902	0.01103921
hemY	0.80376356	10.5561983	30.0466484	0.00063947	0.01117808
rapH	-0.6602173	9.10813614	29.8610954	0.00065217	0.01129663
fmnP	-1.0798493	7.36703167	29.861013	0.00065218	0.01129663
rbsR	-0.8127526	5.32982764	29.8047078	0.00065609	0.01129663
xerD	-0.626193	7.22449212	29.7971737	0.00065662	0.01129663
B4U62_RS17	-1.5478878	8.77081288	32.2325043	0.00065908	0.01129663
htrC	0.62984481	6.61077692	29.5933634	0.00067105	0.01140739
yfmG	1.34617454	10.8996995	29.563813	0.00067318	0.01140739
gutR	-0.7559299	6.64605473	29.5620444	0.00067331	0.01140739
hemL	0.85126174	10.5085913	29.4511897	0.00068136	0.01149961
ycgA	-0.8731163	6.27160969	29.3294947	0.00069034	0.01157062
pbpE	-0.7142109	8.24652157	29.3069627	0.00069202	0.01157062
B4U62_RS18	0.94578573	4.19778866	29.2878815	0.00069345	0.01157062
brxA	-0.6974451	6.14422355	29.1898381	0.00070084	0.01164974
yxjG	-0.7187883	9.70584596	29.103869	0.00070739	0.01171455
ywqB	0.75024627	6.15420335	28.9879278	0.00071636	0.01181314

Table S5. Genes differentially expressed between ΔmnmE and WT in transcriptome analyses

B4U62_RS04(	-0.8397566	6.46052294	28.9578476	0.00071871	0.01181314
coaE	0.80946134	6.70917308	28.9047489	0.00072288	0.01182651
ybbJ	-0.9364407	4.62221832	28.8792919	0.00072489	0.01182651
sigW	-0.8366907	9.15276389	28.8179893	0.00072977	0.01186205
ppsD	-0.8546881	11.0477799	28.7832588	0.00073254	0.01186343
yoyD	0.91974185	5.8564066	28.6915589	0.00073994	0.01190553
tagV	0.6450374	7.67433379	28.684095	0.00074055	0.01190553
coiA	-0.9729724	4.5185686	28.5129766	0.00075462	0.0120877
resE	0.71214453	10.0694664	28.4465953	0.00076017	0.01213249
ytrD	0.83481329	5.1565454	28.410988	0.00076317	0.01213638
skfG	0.88538992	7.0532357	28.3367775	0.00076947	0.0121925
splA	-1.6313434	3.10346571	28.2498439	0.00077693	0.01226659
yhzE1	2.60374918	2.56208882	28.1449098	0.00078606	0.01232817
ndhF	2.68791631	7.719174	36.3798473	0.00078643	0.01232817
hemD	0.7618851	8.3115927	28.0244669	0.0007967	0.01238133
srfAB2	1.12127487	10.5369367	28.0071493	0.00079824	0.01238133
rpsNB	-1.044808	6.14955395	28.0070664	0.00079825	0.01238133
glgP	1.0614374	5.04390715	27.9373261	0.00080451	0.01243465
maa	-2.0318111	4.7739557	29.7421734	0.00080734	0.01243468
tdh	-0.7460454	4.99477775	27.8134501	0.00081579	0.01252103
ytrC	0.77979677	5.79474393	27.6980316	0.00082647	0.01263995
glpK	-0.8457944	8.15506603	27.6680467	0.00082927	0.01263995
yitI	-0.6944733	5.94933318	27.6212634	0.00083367	0.01266318
gerPC	2.07423946	4.03640629	28.193651	0.00084519	0.01277052
yqeK	0.69864795	6.08404919	27.4862062	0.00084654	0.01277052
cypB	0.90002247	8.03724199	27.4431288	0.00085069	0.01278942
hepA	0.88590773	7.13410947	27.3430516	0.00086045	0.01289206
yddM	0.78681535	5.21114744	27.303557	0.00086434	0.01290642
cysC	1.37530724	7.42174343	28.1418075	0.00087723	0.01305465
liaL	1.14670106	9.24249436	27.1298314	0.0008817	0.0130771
rsmJ	-1.0502404	5.46082512	27.0201424	0.00089289	0.01319865
B4U62_RS01:	0.64187247	8.4939018	26.9514289	0.0009	0.01325915
sunS	1.10460785	10.5458029	26.8324519	0.00091246	0.013398
ribD	-1.8776458	9.19109676	31.1759813	0.00092369	0.01351776
xhlB	-0.7473513	7.31980835	26.5663557	0.00094114	0.01372756
yitB	3.53147834	2.01311361	27.1595036	0.000946	0.01375283
lutP	-1.0890437	8.18329366	26.4217014	0.00095721	0.01387005
lctP	1.17344135	3.99162413	26.3540879	0.00096483	0.01393473
secDF	0.81832691	10.5560264	26.3178906	0.00096895	0.01394843
acsA	-0.7607257	7.12272653	26.2784804	0.00097345	0.01396764
yncC	-1.2754858	3.37176507	26.2450523	0.0009773	0.01397724
spoIIIAF	2.51827268	2.34206048	26.1471101	0.00098866	0.01399924
ade	0.6552993	7.22275831	26.1209718	0.00099173	0.01399924
gerPF	2.25933535	2.74407009	26.1165488	0.00099225	0.01399924
B4U62_RS09:	-0.9876497	5.5138648	26.1008659	0.00099409	0.01399924

Table S5. Genes differentially expressed between ΔmnmE and WT in transcriptome analyses

yfmQ	-1.0049821	8.37708162	26.0954637	0.00099473	0.01399924
purN	-2.1850593	6.69283097	31.4766645	0.00100194	0.01404186
xylB	-1.3034601	8.41968819	27.1360128	0.00100682	0.01404186
yjcM	0.91128354	8.1720562	25.9793287	0.00100853	0.01404186
tagF	0.64587204	8.80825235	25.9368955	0.00101363	0.01404186
skfF	0.9827501	8.78306586	25.8947314	0.00101873	0.01404186
ywfL	-0.6244688	6.43466897	25.8911843	0.00101916	0.01404186
B4U62_RS22	2.05657365	1.21488302	25.883743	0.00102007	0.01404186
spoVIF	3.12960688	4.73979629	31.2872761	0.00103162	0.01414844
nupC	0.77553908	8.05668195	25.7682503	0.00103423	0.01414844
lysCA	-1.6690112	8.83735223	29.2111162	0.00103995	0.01417789
B4U62_RS05	1.79064598	1.22579941	25.6991841	0.00104282	0.01417789
cwlO	0.65669212	10.1015859	25.6687854	0.00104663	0.01418589
putM	2.87813159	2.39815214	26.0832245	0.00105184	0.0142128
yozE	-1.0041119	3.99707928	25.4085642	0.00107997	0.01454819
purE	-1.0104078	7.87455655	25.3376933	0.00108928	0.01462886
yjcZ	3.10495246	3.18290088	27.6701933	0.00109606	0.01467516
xynB	-1.578366	6.87770968	27.2814493	0.0011064	0.01476882
glgC	0.81674405	4.74651939	25.0439715	0.00112895	0.01496636
secG	-0.8905682	7.0972923	24.9955405	0.00113567	0.01496636
cwlT	1.30547885	4.00868302	24.8917168	0.00115024	0.01496636
B4U62_RS22	0.9268376	4.41748695	24.8847256	0.00115123	0.01496636
ytlD	2.95170972	2.17621972	25.2030618	0.00115333	0.01496636
fhuB	-0.814293	7.71932337	24.8531375	0.00115571	0.01496636
purM	-2.1391069	7.19217299	29.9226162	0.00115583	0.01496636
B4U62_RS11	-3.0076347	1.75974812	24.8493225	0.00115625	0.01496636
spoIIE	1.57333084	5.11033662	25.4184669	0.00115702	0.01496636
panB	-0.6156735	7.33570052	24.8330116	0.00115858	0.01496636
spoIIID	3.76114743	3.42572414	29.2862317	0.00118228	0.01518385
ytvI	2.22397376	2.6643286	24.6688548	0.00118231	0.01518385
srfAC	1.06468294	9.86240839	24.5828609	0.00119498	0.01530202
yxlA	0.8574577	4.0333841	24.4737463	0.00121131	0.01542682
ureA	1.22890888	3.60566434	24.4513387	0.0012147	0.01542682
ybfJ	-0.7061621	6.57872943	24.4437167	0.00121586	0.01542682
sirA	-1.0203192	3.969762	24.4247503	0.00121874	0.01542682
ytlI	1.55323021	2.00631654	24.39107	0.00122387	0.01544745
ythA	1.21970281	2.64865167	24.2872724	0.00123988	0.01560472
purF	-1.9249745	8.7664305	29.0770184	0.00125079	0.01569723
bdbB	1.02462682	8.76656852	24.1729464	0.00125781	0.01574046
frxB	-0.9879879	8.30601119	24.1276888	0.001265	0.01574448
yugF	1.06863038	2.5803865	24.0450817	0.00127825	0.01574448
walL	1.39762866	8.7376517	26.00351	0.00128046	0.01574448
gntK	-1.0581221	5.21444788	23.9982745	0.00128584	0.01574448
B4U62_RS07	2.00225659	0.15073706	23.9802455	0.00128878	0.01574448
ytkA	-0.648991	7.41895421	23.9735295	0.00128987	0.01574448

Table S5. Genes differentially expressed between ΔmnmE and WT in transcriptome analyses

motS	-1.1475868	2.96506479	23.9732418	0.00128992	0.01574448
dtpT	1.08553097	5.06346975	23.9709405	0.0012903	0.01574448
yodS	3.53501298	0.73857357	23.9001417	0.00130193	0.01578351
murG	0.66278787	6.24740234	23.89692	0.00130247	0.01578351
yfmF	-0.7651143	6.8578572	23.8861744	0.00130424	0.01578351
bioI	-0.5972898	8.16541594	23.80359	0.00131802	0.01582231
artP	0.68808671	8.09793286	23.802982	0.00131812	0.01582231
spmB	1.76418716	1.87478187	23.7779916	0.00132232	0.01582231
yqeH	0.62110771	6.91022069	23.7734076	0.00132309	0.01582231
srfAD	1.03634324	7.54799536	23.7426857	0.00132829	0.01582231
ytxK	-0.585497	6.09611605	23.7173421	0.00133259	0.01582231
hmoA	-0.8534505	6.94082078	23.6842795	0.00133824	0.01584659
pyrR	-1.0483997	9.32303747	23.6126285	0.00135057	0.015933
bsrG	-1.7277058	10.1230923	26.5364382	0.00135277	0.015933
B4U62_RS21	1.7591798	0.90550866	23.4994943	0.00137033	0.01609681
B4U62_RS14	1.61252099	2.28909289	23.3258144	0.00140138	0.01641779
yneT	-0.6964372	5.61720304	23.2862367	0.00140858	0.01645836
gerPD	1.93676662	3.48472372	23.2120129	0.00142221	0.01657364
cotR	1.65636158	3.40637445	23.1197057	0.00143939	0.01667343
endB	-0.6428197	8.81842477	23.1121084	0.00144082	0.01667343
B4U62_RS02	1.53995045	3.0047816	23.0868151	0.00144558	0.01667343
eesA	-0.6418362	8.61811042	23.0850219	0.00144591	0.01667343
ybdG	0.87954651	4.93871976	23.0287351	0.00145658	0.01670675
alsT	0.75925553	5.32479084	22.9796727	0.00146596	0.01670775
mmgA	3.22547828	0.79115201	22.9697913	0.00146785	0.01670775
mhqA	-0.7616701	4.32503517	22.9052919	0.00148032	0.01674986
conD	1.32166838	4.34778464	22.9020927	0.00148094	0.01674986
sspA	0.71724791	5.09322276	22.8722003	0.00148676	0.01674986
asiMA	0.720546	6.58878128	22.8212598	0.00149676	0.01681685
yybK	1.5419587	6.32226994	24.3795282	0.00150049	0.01681685
arsC	1.37803554	2.13414097	22.7690308	0.00150709	0.01681685
srfAB1	1.02191165	9.34281823	22.7645482	0.00150798	0.01681685
citT	-0.7460936	5.40016722	22.7198808	0.0015169	0.01687356
yjbA	0.94036321	4.44411156	22.6959397	0.0015217	0.01688438
thiT	-1.5618468	4.62205143	23.0290163	0.00153011	0.01693498
fur	-0.6147698	7.01825012	22.5901787	0.00154316	0.01703666
yloC	-0.6190071	9.03291685	22.5510707	0.00155119	0.01708252
moaB	-1.1361169	2.55497362	22.489732	0.0015639	0.01717947
yckB	-0.843846	5.66805062	22.3877721	0.00158531	0.01729322
B4U62_RS19	0.99860734	5.22141468	22.374061	0.00158821	0.01729322
spoIISB	-0.7803173	4.898433	22.3471605	0.00159394	0.01729322
sigE	1.37479685	3.11208078	22.3290295	0.00159781	0.01729322
menF	-0.6106289	7.46318115	22.2786715	0.00160862	0.01734793
yrdA	0.64663788	6.85701914	22.2688678	0.00161074	0.01734793
helP	0.94205391	5.82340735	22.2274867	0.00161971	0.01740201

Table S5. Genes differentially expressed between ΔmnmE and WT in transcriptome analyses

nirD	0.81146717	9.96928244	22.0874558	0.00165054	0.01760703
spoVAEA	1.03290483	2.98771749	22.0737115	0.00165361	0.01760703
bglC	-3.1839734	6.14527503	29.7329743	0.00165361	0.01760703
ndoA	-0.6196425	8.61267848	22.0684525	0.00165478	0.01760703
spoVID	1.45038046	6.08201534	23.1138575	0.00166317	0.01765364
tkmA	-0.588979	7.77278568	21.9790171	0.00167492	0.01773567
rbsK	-0.8417373	4.953879	21.8910143	0.00169505	0.0179057
pksS	-0.7294267	7.07096395	21.8570565	0.00170289	0.01791556
tagA	-0.73825	8.86748004	21.8507618	0.00170435	0.01791556
rapE	-1.0287716	7.42862488	21.8134946	0.00171302	0.01791556
bsrH	-1.3409867	7.34611048	23.0373636	0.00171611	0.01791556
fldN	-0.668444	8.8670724	21.7994149	0.00171631	0.01791556
B4U62_RS17	2.50779146	2.23300642	21.7620672	0.00172508	0.01796451
B4U62_RS18	0.60774353	6.4054513	21.7271956	0.00173332	0.01800773
yfiS	1.30526732	3.18227692	21.5497865	0.00177601	0.01833441
yutI	-1.0460267	6.59342644	21.5446861	0.00177725	0.01833441
yvqJ	0.67170687	8.31999054	21.4742678	0.00179458	0.01846867
comS	1.10240116	6.23532772	21.4512178	0.00180029	0.01846867
yrzF	-0.7623736	6.81786787	21.4336618	0.00180466	0.01846867
hpf	0.68520741	10.1713086	21.4187987	0.00180837	0.01846867
katA	-1.5179294	11.5121711	22.8177224	0.00181439	0.01846867
spoIIQ	2.6647852	4.95457234	25.431618	0.00181542	0.01846867
braB	0.7662868	5.53555477	21.3595939	0.00182325	0.01850553
ypuA	0.72353969	5.67508422	21.3375327	0.00182883	0.01851951
yodQ	2.27680084	2.43141475	21.3186999	0.00183361	0.01852534
conC	1.20938233	3.76070102	21.2406497	0.0018536	0.01868441
pdaB	3.47347562	1.71054148	21.9080058	0.00185795	0.01868553
lrgA	5.15410099	6.72879357	29.4025263	0.00187121	0.01870596
yheD	2.36417512	4.44635055	24.2019298	0.0018721	0.01870596
ybcF	2.53897276	3.93097912	23.6887116	0.00187697	0.01870596
B4U62_RS10	1.58495338	2.60231127	21.0857833	0.00189407	0.01883381
yybJ	1.55173946	6.94106175	23.4000061	0.00190121	0.01886225
qdoI	-1.0511904	3.36709516	21.0334464	0.001908	0.01888705
prkA	2.62300563	4.54987162	24.8457957	0.00191551	0.01891888
spsE	2.02260894	4.90650928	23.4614215	0.00192759	0.01895868
ydeR	1.15708837	2.70038645	20.9585888	0.00192815	0.01895868
rsfA	1.78178232	3.11783672	20.9288093	0.00193624	0.01897862
ywrD	1.60939348	1.79380772	20.913434	0.00194043	0.01897862
B4U62_RS22	1.49333557	2.40449562	20.8754487	0.00195084	0.01899499
ytcA	2.9579189	4.03113499	24.6644301	0.0019534	0.01899499
mgtE	-0.7893618	7.30488622	20.765055	0.00198149	0.01915109
spsL	1.86105124	2.98938309	20.7488253	0.00198605	0.01915109
xylA	-1.3492323	9.7331205	21.8630488	0.00199327	0.01915109
phrE	-1.1515465	7.43412123	21.135393	0.00199817	0.01915109
B4U62_RS22	-3.7504128	-0.0374752	20.6998364	0.00199989	0.01915109

Table S5. Genes differentially expressed between ΔmnmE and WT in transcriptome analyses

yqgW	-0.7549912	7.84197544	20.6485389	0.00201451	0.01924924
B4U62_RS13	-0.767502	3.9822311	20.6295295	0.00201996	0.01925955
mrgA	-1.0679271	6.79911821	20.5472965	0.00204376	0.01940247
rsuA	0.83754835	4.27796123	20.4442308	0.00207408	0.01963882
tmrB	-0.7906178	4.46440606	20.3839273	0.00209209	0.01973376
glnH	3.95491775	2.80190732	23.7289331	0.00209874	0.01975416
ytlC	2.9448514	2.1971887	20.9756012	0.00211523	0.01982467
dhbF	-0.6831597	13.3457071	20.249003	0.0021331	0.01991359
purR	0.60288425	8.27176126	20.2468594	0.00213376	0.01991359
ptsH	-0.787463	8.10022876	20.2219457	0.00214145	0.01992178
ylbB	0.84672195	4.0272091	20.214717	0.00214368	0.01992178
xynP	-1.7049734	6.81745978	23.070996	0.00215196	0.01995662
ppsB	-0.6031844	10.5237295	20.1183977	0.00217377	0.02011647
cwlQ	-0.7064656	7.36416713	20.074504	0.00218765	0.02020252
ylnD	1.34354752	7.96977174	21.6452945	0.00220202	0.02026584
fhuC	-0.6104131	8.10716033	20.0241783	0.00220371	0.02026584
yoeA	0.75854831	8.95066783	19.9796962	0.00221803	0.02032672
nadD	0.59634091	6.07810283	19.9749582	0.00221956	0.02032672
putC	1.25112846	7.07387121	20.8151513	0.00224326	0.02045875
srfAA	0.94168159	10.6405045	19.8781788	0.00225114	0.02048818
yrzH	-1.1289872	5.83210854	19.7788235	0.00228416	0.02067295
xpt	-1.713939	5.7350475	22.00715	0.00228685	0.02067295
pheA	0.64676154	6.73334478	19.7614788	0.00228998	0.02067295
B4U62_RS02	0.96092685	5.51568332	19.7499887	0.00229385	0.02067295
purL	-1.74268	9.04270519	23.2131269	0.00229491	0.02067295
xre	-0.7272644	5.0378321	19.6613966	0.00232397	0.02089199
yczM	1.9917591	3.96995137	20.6234876	0.00235085	0.0210906
B4U62_RS11	-1.965055	0.32344423	19.5650007	0.0023573	0.02110552
dgcK	-0.8201376	5.63732017	19.5300967	0.00236952	0.02117189
cotF	3.73941191	3.6444126	23.5050935	0.00238545	0.02127102
yckD	1.08405509	2.32975084	19.4174245	0.00240951	0.02144222
yczE	-0.6165435	5.42822254	19.3919672	0.00241866	0.02148028
sepF	-0.5883497	7.63573888	19.3558137	0.00243174	0.02154998
sxzZ	-0.6625427	7.09875954	19.3373757	0.00243844	0.02154998
yodT	3.16266214	1.92343598	20.0137422	0.00245031	0.02154998
ybcH	1.86113368	2.79390758	19.299981	0.0024521	0.02154998
B4U62_RS02	1.06230016	4.46807319	19.2794763	0.00245964	0.02154998
B4U62_RS05	-1.5914979	3.26940454	19.2654246	0.00246482	0.02154998
tcaB	1.36518442	4.37695877	19.251022	0.00247014	0.02154998
guaC	0.88861451	7.40187144	19.1739578	0.00249887	0.02154998
natR	-0.6335012	5.22989574	19.1634935	0.0025028	0.02154998
B4U62_RS21	0.70409684	5.93020166	19.1521526	0.00250707	0.02154998
yhcN	1.18860403	4.19540065	19.146066	0.00250937	0.02154998
wprA	0.79364691	9.66761849	19.1450564	0.00250975	0.02154998
racX	-0.6721512	7.86378089	19.131801	0.00251476	0.02154998

Table S5. Genes differentially expressed between ΔmnmE and WT in transcriptome analyses

metE	-1.9253415	10.4988893	22.4005644	0.0025206	0.02154998
B4U62_RS22'	-2.5000264	2.05807983	19.1075442	0.00252737	0.02154998
cse15	2.50626426	2.99903257	20.1043165	0.00253158	0.02154998
yyaH	-1.1575131	2.29518204	19.0814991	0.00253389	0.02154998
B4U62_RS18:	1.18000637	4.38350014	19.0764569	0.00253582	0.02154998
pgl	-0.6581321	7.82196244	19.0680684	0.00253903	0.02154998
prsW	-0.5958353	8.25202909	19.0455132	0.00254769	0.0215819
yvkC	0.68397723	4.83104131	18.9874071	0.00257018	0.02166293
cstA	-0.7421364	5.68465285	18.9725339	0.00257597	0.02166293
B4U62_RS22:	0.62042781	7.09510772	18.9587272	0.00258136	0.02166293
yxaM	0.58762056	9.86494706	18.9574894	0.00258185	0.02166293
hupN	-3.3741963	0.43516759	18.9326076	0.00259161	0.02168365
B4U62_RS12·	1.00753267	2.94436211	18.9261127	0.00259416	0.02168365
yitH	-0.5870939	6.59501034	18.8927851	0.00260732	0.02172934
acuA	-0.6415451	7.45739841	18.8873048	0.00260949	0.02172934
pdaC	-1.0599499	6.96173031	19.0660218	0.0026235	0.02180474
ywrK	2.14487724	5.53478777	22.0398974	0.0026308	0.02182425
dacF	1.98406139	2.77135359	18.7564511	0.00266204	0.02202507
queD	0.63853222	4.7267382	18.7368983	0.00267001	0.02202507
opuBA	0.84288489	6.23795863	18.7039997	0.00268348	0.02206395
B4U62_RS12(	-0.6420531	5.80948281	18.7009335	0.00268474	0.02206395
ablA	1.96415357	2.85987669	18.5526861	0.00274655	0.02247155
psmA	1.40326756	1.69336305	18.5453908	0.00274964	0.02247155
ytlA	2.79834097	2.50565186	19.4639697	0.00276264	0.02249435
yubD	1.00635648	3.90359307	18.4907913	0.00277289	0.02253612
acnA	1.17532807	10.8121481	18.8245231	0.00278145	0.02254473
skfE	0.76631609	8.27585793	18.4644744	0.00278418	0.02254473
ssrA	-0.6187158	15.1138519	18.4307872	0.00279873	0.02259711
B4U62_RS11:	-2.2766816	1.05864905	18.4257422	0.00280091	0.02259711
ribBA	-1.8159671	9.3971614	21.5016961	0.00280604	0.02259711
yflR	-0.6979819	5.90059621	18.382776	0.00281962	0.02262585
yobO	-0.8222383	8.16427576	18.370434	0.00282502	0.02262585
ytwF	-0.7184757	4.685209	18.3513208	0.00283341	0.02265188
B4U62_RS23	-1.9486038	2.57362554	18.3385743	0.00283903	0.02265564
ffs	-1.1841028	10.657197	18.6677139	0.00284928	0.02269631
yxaH	-0.9844821	3.47466662	18.279075	0.00286542	0.0227694
ytcQ	-0.8929028	3.75708384	18.1747109	0.00291246	0.02307444
ywaD	0.80667172	5.95756891	18.1348018	0.00293071	0.02313577
psmB	1.42513016	1.77425478	18.1056466	0.00294413	0.0231785
ribH	-1.677528	8.4858353	21.1006869	0.00294664	0.0231785
ptsJ	1.5604991	1.35964099	18.0809714	0.00295555	0.02320709
ydcH	-0.6673259	4.64161418	18.0569854	0.0029667	0.02325322
ywjG	0.79235147	5.11620948	18.039545	0.00297484	0.02327563
oxdD	2.04951456	2.31030134	17.9632874	0.00301078	0.0234227
helD	0.61877833	5.64050798	17.9602279	0.00301223	0.0234227

Table S5. Genes differentially expressed between ΔmnmE and WT in transcriptome analyses

spoIIIAB	1.76465843	1.36523934	17.9189006	0.00303195	0.02342828
spmG	2.44934091	4.08644835	20.4784541	0.00305878	0.02352534
ybbK	-1.0040463	4.69677212	17.8603479	0.00306016	0.02352534
comFB	-0.6100472	4.40384443	17.8445366	0.00306784	0.02354325
spaC	2.54966383	4.34839514	20.7327549	0.00308385	0.02354803
yxlF	-0.8395714	4.77612271	17.7777352	0.00310054	0.02354803
glnQ	3.39031958	2.74410546	20.0680339	0.00311905	0.02361834
ycsA	-0.6755729	4.38496986	17.737348	0.00312052	0.02361834
cotZ	3.54976196	6.0088351	23.7413471	0.00314175	0.02369764
oxdC	1.61211442	4.04490376	17.9975127	0.00315659	0.02376883
B4U62_RS10	2.07904092	1.25599276	17.6473227	0.00316564	0.02379633
ytaF	0.7158504	7.08173145	17.6326043	0.0031731	0.02381174
spoIID	-1.1905647	5.87450673	17.9506256	0.00317962	0.02382013
usd	3.64122189	2.25816057	19.4535126	0.00319936	0.02392012
ykzV	2.21391498	1.63748883	17.57239	0.00320383	0.02392012
gutB	-1.4202117	2.00014573	17.5017142	0.00324038	0.02415209
spoVR	2.08749965	2.88107293	17.7214263	0.0032937	0.02442549
mraY	0.6538101	7.49428164	17.2695261	0.00336423	0.02490657
mrpC	0.71378858	4.23912074	17.1782518	0.00341454	0.02519437
yhbY	0.84475167	4.94204311	17.1550647	0.00342747	0.02520535
yfjT	-0.8681085	3.55824693	17.1113959	0.00345199	0.02534338
B4U62_RS12	0.71208087	4.03070067	17.096944	0.00346016	0.02536105
rsmE	0.58225794	6.53846254	17.0497692	0.00348698	0.02547287
yjcF	-0.6098875	7.77253732	17.0170257	0.00350575	0.02549355
pchC	-0.58211	13.1538373	17.0151367	0.00350684	0.02549355
yqcK	1.34898873	2.19248928	17.0120646	0.0035086	0.02549355
bsuRC	0.65585437	7.72598178	17.0045053	0.00351296	0.02549355
prtG	2.36264777	2.8600407	17.7481304	0.00353642	0.02553776
spsF	2.04231789	4.3684037	18.8787683	0.0035382	0.02553776
nasF	0.90117104	10.8944052	16.9105107	0.0035677	0.02567564
spmH	2.25747699	3.64987195	18.4729946	0.00358522	0.02567564
spoIIIAE	1.46700365	2.66216325	16.8794107	0.00358604	0.02567564
kefQ	0.65845643	4.20086096	16.8765927	0.00358771	0.02567564
spsG	2.070506	4.4454316	18.9614986	0.00359051	0.02567564
gutP	-1.0727872	3.34647314	16.8474827	0.003605	0.0257247
spsD	2.09925619	4.37304269	18.8750304	0.00363333	0.0257247
hmpA	1.33815826	8.13946492	18.5345188	0.00363452	0.0257247
putB	1.38635944	5.79774774	17.7776986	0.0036357	0.0257247
cdd	-0.6406255	9.51164785	16.7903516	0.00363924	0.0257247
ylbD	2.02531147	2.08564828	16.7862727	0.0036417	0.0257247
ywcH	-0.7584066	4.94224651	16.7823173	0.00364409	0.0257247
B4U62_RS10	1.68748992	2.738458	16.7614047	0.00365675	0.02577275
spoIVA	2.16588989	6.06357851	20.0734779	0.00367452	0.02585666
pghB	-0.7423066	4.92777981	16.7084399	0.00368905	0.02591116
yheG	-0.865454	4.70868803	16.6790308	0.00370715	0.0259206

Table S5. Genes differentially expressed between ΔmnmE and WT in transcriptome analyses

sspN	2.83204541	1.27021702	16.6224537	0.00374673	0.02611446
B4U62_RS15(	-1.0497372	5.46990042	16.5901769	0.00376249	0.02618292
B4U62_RS21(	-0.6569591	5.60338467	16.5342989	0.00379784	0.02634561
yvbJ	-0.9836306	6.6021628	16.4533352	0.00385433	0.02661182
ilvE	-0.922658	6.03111763	16.3477626	0.00391888	0.02701516
gerPE	1.9556259	4.29688227	17.9675509	0.0039416	0.02712926
aroB	0.65699142	8.60557608	16.2889127	0.00395808	0.02720022
yvgT	-0.7495394	3.85033886	16.1057511	0.00408327	0.02772446
B4U62_RS22!	1.20408748	1.34072423	16.1036827	0.00408472	0.02772446
cotJB	3.69837296	2.7823704	18.5066268	0.00412053	0.02792452
phrK	-0.7788437	6.04682732	16.039407	0.00412985	0.02794469
gerE	1.06276236	4.92155676	15.9678024	0.00418087	0.02824655
yisZ	2.6540235	3.08062027	17.5278346	0.00420317	0.0283537
B4U62_RS22:	-1.5667214	0.68472618	15.9068154	0.00422496	0.0284388
ftsY	0.64164011	6.02984259	15.8320637	0.0042798	0.0286254
ykvU	1.56313857	2.85753882	15.8298971	0.0042814	0.0286254
yfnE	1.88791177	4.23223801	17.2542174	0.0042976	0.02868321
B4U62_RS23	2.62628163	2.97541202	17.3117709	0.00430783	0.02870802
acdA	0.91919161	3.99475909	15.767602	0.00432781	0.02875954
thpR	-0.5898116	5.33363618	15.7516842	0.00433977	0.02879017
yjzH	0.75380876	5.30664663	15.7019656	0.00437739	0.02890916
rsiW	-0.8359726	8.89831679	15.6654866	0.00440526	0.02904965
yonR	-0.8711166	3.69226258	15.6291872	0.00443321	0.02919029
splB	-1.1636151	4.64396632	15.5807041	0.0044709	0.02931258
gntP	-0.6197258	5.9931916	15.5796157	0.00447175	0.02931258
cotY	3.27547737	5.86475896	20.4185777	0.00447866	0.02931426
yoaD	-2.0437798	6.22849418	18.5030036	0.00449415	0.02937199
ytrB	0.68671201	5.17027967	15.5397665	0.00450303	0.02938646
B4U62_RS11	-2.4318701	0.96812106	15.5050666	0.0045305	0.02952197
yybL	1.27949316	6.6034044	16.5837858	0.00453836	0.02952953
glnM	2.47298913	1.22621123	15.4750797	0.00455441	0.0295902
yqgE	-1.1353051	5.63045106	15.6993868	0.00458834	0.02976675
yflP	-0.6519611	5.36699284	15.3962063	0.00461805	0.02991545
yunB	0.70892565	3.92102605	15.3715007	0.00463822	0.03000196
cotJA	3.33516906	3.20143589	17.6315955	0.00468214	0.03020592
yqfT	1.62294369	1.18157341	15.3159039	0.004684	0.03020592
speE	-0.9245681	5.40015746	15.308288	0.00469032	0.03020592
dnaN	0.59085848	7.03218774	15.2927106	0.00470327	0.03024512
B4U62_RS10:	1.72107966	2.80795872	15.213958	0.00476945	0.03056797
cotB	2.14139105	4.78417838	17.4490883	0.00477429	0.03056797
ytcB	3.13473665	3.64637193	17.8360543	0.00478842	0.03061392
rpoZ	-0.6990464	6.14455692	15.1602348	0.00481527	0.03065212
folN	-0.6588373	4.53914846	15.1503009	0.0048238	0.03066213
yutG	2.35054796	1.88450399	15.2335785	0.00488061	0.03085953
bioYB	-0.9919826	9.14296048	15.4717015	0.0048823	0.03085953

Table S5. Genes differentially expressed between ΔmnmE and WT in transcriptome analyses

cesA	1.53681074	6.68852364	17.1229045	0.00488288	0.03085953
spsB	2.09510154	4.77196031	17.2099237	0.00494064	0.03109075
bdbA	0.9225987	8.93319117	15.2262369	0.00497971	0.03121742
yotM	-3.1047207	-0.8265274	14.9700222	0.00498203	0.03121742
mdtP	-0.6188142	7.28077144	14.9293867	0.0050186	0.03133539
fabHB	0.93329726	2.51313215	14.9175484	0.00502931	0.03133539
rpsT	-0.8271616	6.58354086	14.8855187	0.00505845	0.03138376
xkdJ	-0.6343975	7.51537841	14.853931	0.00508739	0.03151895
spsI	1.72463347	3.95115327	15.5135984	0.00509959	0.03152023
cotE	3.29437841	3.62393844	17.6588711	0.00510233	0.03152023
yfnC	0.65700273	7.87102881	14.830427	0.00510907	0.03152023
cotP	2.63907103	1.49626763	14.9476761	0.00517433	0.03187822
ywfO	-0.8406878	7.88601564	14.7377163	0.0051957	0.03190371
dinL	0.64919308	5.44864934	14.7349141	0.00519835	0.03190371
ribE	-1.9141908	7.8304953	17.9127532	0.0052002	0.03190371
feuB	-0.6098638	10.447527	14.7204364	0.00521205	0.03192708
B4U62_RS22	-0.7766537	4.35300282	14.7097987	0.00522215	0.03192708
ypjB	0.5832233	5.6573773	14.7060124	0.00522575	0.03192708
bceB	0.65619074	5.37630735	14.6728606	0.00525741	0.032076
B4U62_RS05	2.96973944	4.63212017	18.300858	0.00529252	0.03224554
yrdP	0.97446272	2.3610584	14.6154978	0.00531276	0.03232415
ftsX	0.59298238	8.10765014	14.5805088	0.00534688	0.03248688
bdbD	0.58311654	9.2621638	14.5702252	0.00535696	0.0325033
rocG	0.86349441	3.70848416	14.5241189	0.00540245	0.03268929
ltdD	0.64229797	4.04376205	14.5036555	0.0054228	0.0327674
ptsG	-3.2916445	8.32241357	18.7670933	0.00543789	0.03281359
B4U62_RS10	1.95125763	0.85895054	14.4474397	0.0054792	0.0329725
murI	-0.5899134	5.02700429	14.4265638	0.00550033	0.03305451
yrzI	0.80159595	4.03947458	14.3819669	0.00554582	0.03323719
spoIIP	0.88045346	3.45265958	14.3345348	0.00559472	0.03339398
sspB	1.32206829	3.72928045	14.2951071	0.00563579	0.03357796
B4U62_RS11	-1.0320465	3.17595738	14.2771825	0.00565458	0.03361462
thrD	0.59406989	7.9034056	14.2546109	0.00567836	0.03369733
conB	1.18537904	5.51940076	14.6675528	0.00569714	0.03369733
slpS	2.33016924	1.8879966	14.4114777	0.0056991	0.03369733
spmA	1.40900364	2.22717958	14.2128816	0.00572266	0.0337913
ytrF	0.62267631	5.39560566	14.1835327	0.00575407	0.03393132
B4U62_RS11	-1.9336123	0.16846622	14.1459534	0.00579462	0.03412472
yocR	0.61111396	6.68775122	14.1263232	0.00581594	0.03420455
ycsD	-0.9103361	7.09274379	14.2395936	0.00584712	0.03424567
yfiV	-0.8473669	3.10942644	14.0954155	0.00584971	0.03424567
phrC	-1.0207633	5.84026044	14.2334643	0.00585373	0.03424567
yrhH	1.06121173	2.88856812	14.091482	0.00585403	0.03424567
psdB	0.68987122	6.52387096	14.0202081	0.00593293	0.0344977
murB	0.72411296	6.19408316	14.0172168	0.00593627	0.0344977

Table S5. Genes differentially expressed between ΔmnmE and WT in transcriptome analyses

mmgD	1.91593308	2.23727494	14.0056297	0.00594923	0.03449997
yugS	0.85348317	3.69986795	13.9984433	0.00595729	0.03449997
B4U62_RS11	-2.2808044	0.67949407	13.9478285	0.00601443	0.03476845
purQ	-1.4906384	7.67011726	15.6904602	0.00619626	0.03562343
yhjA	-0.6323072	6.70303696	13.7512222	0.00624306	0.03574519
cwlH	2.17695701	2.11653981	13.9134112	0.00624831	0.03574519
thiC	-2.4950837	7.76298064	17.6258565	0.00626925	0.03581848
cidB	2.36913752	5.5091505	16.7822493	0.00628856	0.03588226
uxuA	1.62241154	1.48555612	13.6476761	0.00636788	0.03625146
B4U62_RS05	2.50384194	5.86290174	17.2193146	0.00636972	0.03625146
yodD	-0.8169664	2.9958617	13.5863862	0.00644325	0.03652946
cotJC	3.37359071	3.35871495	16.0306381	0.00644653	0.03652946
yfnF	2.34741075	3.89028287	15.387183	0.00649409	0.03658117
B4U62_RS09	-0.600958	4.35979453	13.5382639	0.00650321	0.03658575
B4U62_RS11	3.11804378	-1.2079109	13.4789702	0.00657807	0.03691336
comER	1.55959769	3.39013069	13.6324379	0.0065782	0.03691336
yjdF	-0.5930149	4.16745496	13.4616123	0.00660019	0.03697598
rfbB	1.53868554	4.33460137	14.1389963	0.00660615	0.03697598
sdpC	0.80425411	12.3733401	13.4239307	0.00664853	0.03716091
yhcQ	0.99858081	4.52727505	13.4180907	0.00665606	0.03716091
ybcI	1.16696777	2.45704409	13.3763251	0.00671025	0.03736869
mntD	0.79632424	4.6229789	13.3688351	0.00672002	0.03737588
tyrA	0.65525416	8.03898827	13.3199569	0.00678426	0.03759077
yjoA	-0.5805905	6.70023591	13.2874611	0.00682739	0.03778225
pyrAA	-3.0829668	6.01181456	16.929722	0.0068624	0.03787764
yppG	1.16476885	2.09952732	13.2547929	0.00687111	0.03787764
mrpE	0.61395874	4.6853584	13.2547167	0.00687121	0.03787764
ypqE	-0.7187943	4.82534258	13.2489037	0.00687903	0.03787764
B4U62_RS15	-0.8143152	9.91887028	13.226074	0.00690983	0.03799975
lcfA	-0.6232998	8.0529742	13.1637802	0.00699477	0.03829378
mrpB	0.60543611	4.59846808	13.1613813	0.00699807	0.03829378
yitA	2.45606395	3.73961146	14.9025734	0.00701391	0.03833283
yjzD	-1.1605626	4.85372958	13.3405212	0.00703026	0.0383746
fabD	0.59785182	6.71364459	13.1154476	0.00706159	0.03840538
yonK	-3.4778588	-0.5702643	13.1057162	0.00707514	0.03840538
gerD	1.85389615	0.92484785	13.1024044	0.00707975	0.03840538
fatR	0.60723879	5.1473724	13.1002875	0.00708271	0.03840538
yxlE	-0.9352838	3.26616511	13.096466	0.00708805	0.03840538
yhzC	-1.1014018	6.34822188	13.6329742	0.00708821	0.03840538
nucB	0.86344468	2.43912555	13.0821707	0.00710806	0.0384057
mrpF	0.6952835	4.09150292	13.0819639	0.00710835	0.0384057
mcpB	0.63294281	6.23465425	13.0622595	0.00713604	0.03847525
cotNH	-0.660172	4.60052861	13.0346796	0.00717504	0.03863355
czcD	1.24771389	1.83394246	13.0291152	0.00718295	0.03863355
yqbD	-1.3242442	1.43281457	12.9621532	0.00727889	0.03895918

Table S5. Genes differentially expressed between ΔmnmE and WT in transcriptome analyses

ydgA	3.3030396	0.75659574	13.236406	0.00728418	0.03895918
fhuG	-0.6037608	8.00336211	12.9470699	0.00730073	0.03895918
metI	-1.1184499	7.97899718	13.973673	0.00730866	0.03895918
yrbE	-0.7380798	6.99631317	12.9377626	0.00731424	0.03895918
albF	0.70890337	12.0304823	12.9291095	0.00732684	0.03897913
asiMB	0.7963067	4.41116048	12.8803074	0.00739838	0.03922843
yukE	-0.8212372	7.14810718	12.8760837	0.00740461	0.03922843
B4U62_RS22	-2.4771012	-0.7292881	12.8728941	0.00740932	0.03922843
spsC	1.98517985	4.50704382	14.5272923	0.00745319	0.03927255
cotSA	2.43665877	2.84412974	13.9522156	0.00745331	0.03927255
cotQ	2.41041671	1.58423243	13.0317555	0.00746276	0.03927536
sdhC	-0.6073651	8.18635566	12.828763	0.00747489	0.03929225
ylaK	1.3186898	3.55136197	12.8184305	0.00749035	0.03932658
yxkC	1.0718103	6.37329087	13.2960274	0.00750656	0.03936477
ykvO	-0.9022782	2.33242146	12.7993447	0.00751901	0.03938318
cotM	1.88897094	2.48031136	12.9220419	0.00755661	0.0394905
B4U62_RS10	-2.4705523	-0.3370354	12.7713126	0.00756136	0.0394905
rex	-0.6888736	8.72952373	12.7629738	0.00757402	0.0394905
yczN	1.68242752	4.39503099	13.856047	0.00757971	0.0394905
ycbK	0.87022125	2.93387526	12.7386417	0.0076111	0.0395831
lipC	0.62269955	5.19230269	12.6772987	0.0077056	0.03993616
gerQ	3.23292987	4.22976103	15.790195	0.00774019	0.04001825
mmgC	2.6305688	1.90861038	13.113992	0.00788703	0.0406819
yxaC	0.76150434	3.43717094	12.5287646	0.00794064	0.04086433
sigY	-0.8520623	4.09988363	12.5285766	0.00794095	0.04086433
B4U62_RS09	2.21344376	1.16949764	12.5145978	0.00796353	0.04092113
nsrR	-0.9052059	4.72231916	12.5102544	0.00797056	0.04092113
yhgE	0.58417099	6.77943026	12.474025	0.00802954	0.04117593
xis	0.76677407	4.45503874	12.4189673	0.00812023	0.04155684
glgD	0.8514144	5.25433153	12.4101534	0.00813486	0.04155684
dacB	1.5018897	2.62177961	12.395959	0.00815851	0.04155684
thrZ	-0.6877442	6.16363729	12.3950468	0.00816003	0.04155684
adhA	-0.6967145	4.74954421	12.3936319	0.00816239	0.04155684
yhaJ	-0.6285654	4.97649687	12.3891629	0.00816986	0.04155684
spoIIIAA	1.72102315	2.53930321	12.377827	0.00818883	0.0415779
ypbG	0.76768913	3.60538934	12.3754169	0.00819288	0.0415779
bglA	0.87133381	4.19021712	12.3667098	0.0082075	0.04160418
vbfB	0.62429078	4.33901218	12.3609501	0.00821719	0.04160543
abfB	-0.837982	3.36909394	12.287533	0.008342	0.04211902
B4U62_RS22	-1.4786189	2.49195074	12.2390324	0.00842576	0.04246623
ytzH	1.39653848	1.4083457	12.2182468	0.00846199	0.04260006
ycgJ	2.39410804	7.29955152	15.359444	0.00850942	0.04276767
cgeA	2.75519087	5.73081197	15.3030335	0.0085781	0.04297315
B4U62_RS22	-1.6393836	1.64290975	12.14597	0.00858949	0.04297315
ytcP	-0.9673766	2.23605536	12.1430815	0.00859463	0.04297315

Table S5. Genes differentially expressed between ΔmnmE and WT in transcriptome analyses

B4U62_RS07	-0.6778586	4.66013221	12.1315738	0.00861517	0.04299663
ylmB	-1.2679365	4.55533545	12.4597419	0.00861885	0.04299663
ydgB	2.78259388	1.48888539	12.5766388	0.00863959	0.04303176
csk22	2.74644133	2.47505308	13.2680148	0.00864543	0.04303176
opuBB	0.79640969	4.76544488	12.0702002	0.00872576	0.04325451
mneS	-0.6663343	5.93556413	12.0629765	0.0087389	0.04325451
yhbI	0.84146185	3.34342809	12.060913	0.00874265	0.04325451
albE	0.68800983	12.3161962	12.0604946	0.00874341	0.04325451
pbpX	0.79813569	4.65127442	12.0523425	0.00875828	0.04325451
ykvP	1.29655361	3.48316026	12.0514242	0.00875995	0.04325451
pbuX	-1.505513	6.56708947	13.5570401	0.00876874	0.04325451
glnP	1.77820722	1.34438939	12.0225127	0.00881293	0.0433575
gltC	0.60094698	6.16046643	11.9959834	0.0088619	0.04351915
ugpC	-0.8459471	5.22775057	11.9563679	0.00893567	0.04378379
ydjN	0.60918617	5.90410475	11.9146954	0.00901411	0.04402122
cotS	2.37409148	2.79112464	12.847512	0.00908857	0.04423774
ymxH	1.09118658	2.06552706	11.8478604	0.00914173	0.04439837
cydA	0.68385348	5.8667156	11.8246949	0.0091865	0.04456665
scrA	-0.7203829	3.89977038	11.8114499	0.00921222	0.04464227
cotI	2.92668532	3.53434342	13.7170719	0.00925111	0.04478146
cotX	2.70834969	7.34357896	14.6940133	0.0093697	0.04509721
ytzI	-0.8001553	4.88917896	11.7273041	0.00937776	0.04509721
bdbC	0.68888284	9.24452216	11.7044485	0.00942337	0.04524322
yneN	0.88942571	5.56512084	11.7018053	0.00942867	0.04524322
yhcB	0.72371945	4.08139344	11.6339501	0.00956584	0.04575195
mtnA	-1.5724258	7.98238284	13.4779584	0.00962616	0.04584132
B4U62_RS22	-1.8321688	0.47224018	11.5904197	0.00965517	0.04592985
ydbB	-0.6027711	4.22693154	11.5602554	0.00971769	0.04617739
ytcC	3.13227571	3.64563	13.8113271	0.00976746	0.04636385
exuT	1.34090631	2.58559052	11.51191	0.00981897	0.04650812
bcaP	-0.6010003	7.03917359	11.4975238	0.00984936	0.04660197
yfiH	-0.995202	1.75383108	11.4600896	0.00992901	0.04691004
trmB	-0.5914396	6.38226096	11.4332205	0.00998667	0.04704952
yodH	1.7087814	2.77435474	11.5903474	0.00999773	0.04705129
xlyB	-0.8048755	6.45681168	11.4193016	0.01001671	0.04709032
mntC	0.83185729	5.25739078	11.4051994	0.01004727	0.04713334
ablB	1.77423398	2.04232045	11.3497105	0.01016863	0.04751442
yezD	1.18900271	0.52646051	11.3483438	0.01017165	0.04751442
B4U62_RS11	-1.5861273	1.53247996	11.3360044	0.0101989	0.04759125
prfA	-0.6268256	7.35820488	11.287081	0.01030785	0.04791504
arfM	1.51601052	3.4205803	11.5048222	0.01031473	0.04791504
levD	-0.8438103	2.19432529	11.2675711	0.01035171	0.04791504
dpaB	1.95014481	3.58210918	12.1988326	0.01041995	0.04816355
yxlC	-0.7262833	3.31622985	11.2305851	0.01043551	0.04818491
rpsO	-0.6340322	9.52832465	11.2038354	0.01049665	0.04841647

Table S5. Genes differentially expressed between ΔmnmE and WT in transcriptome analyses

skfA	1.31155016	6.7614302	12.4436696	0.01055557	0.04863732
rpsR	-0.680537	6.91884213	11.1600463	0.01059771	0.04878048
ykwD	0.63914333	3.85802168	11.1184765	0.0106948	0.0491036
B4U62_RS11:	-1.7695505	1.43750511	11.0339762	0.01089563	0.0497876

Table S5. Genes differentially expressed between ΔmnmE and WT in transcriptome analyses

E. Significantly differentially expressed genes (contrast of interest *ΔmnmE* -WT) in MSGA, 24H timepoint:

gene_name	logFC	logCPM	F	PValue	FDR
lctE	-5.8820916	13.1118514	2687.36962	6.43E-12	2.83E-08
lctP	-5.5191397	13.4376808	2049.74638	2.02E-11	4.45E-08
nirC	-5.4563479	9.2833005	1240.39644	1.69E-10	2.48E-07
cggR	-4.558043	10.8134277	1122.20971	2.57E-10	2.50E-07
yybF	-4.738259	9.71364156	1096.20999	2.84E-10	2.50E-07
gap	-3.4318331	12.5623075	1006.45253	4.07E-10	2.99E-07
yhgD	-4.0026072	7.96252684	899.497543	6.53E-10	4.11E-07
cydD	-3.4036655	11.2359345	866.230063	7.65E-10	4.21E-07
ywfA	4.27265231	8.52888071	816.040054	9.84E-10	4.81E-07
yhgE	-3.9560873	9.7348736	730.153014	1.57E-09	6.91E-07
cydB	-3.9498047	10.4768116	710.446835	1.76E-09	7.05E-07
pgk	-2.7970316	11.3543915	651.767629	2.53E-09	9.28E-07
cydC	-2.8313297	10.6340896	580.307483	4.11E-09	1.38E-06
mnmE	-9.6679135	6.8986209	571.632983	4.38E-09	1.38E-06
guaC	-2.7082447	7.23511819	558.080995	4.84E-09	1.42E-06
gpmI	-2.6966275	11.5836902	528.605577	6.08E-09	1.67E-06
pyrG	-3.0234957	7.61707057	507.973032	7.18E-09	1.86E-06
yfkAB	-2.7783385	6.76609996	494.474342	8.03E-09	1.86E-06
tpiA	-2.6609193	10.5987102	487.023985	8.56E-09	1.86E-06
cydA	-4.3246504	10.5114381	486.872565	8.57E-09	1.86E-06
eno	-2.4858167	11.4962757	483.142208	8.85E-09	1.86E-06
tasA	4.79378089	6.00595554	473.678995	9.61E-09	1.87E-06
fruA	-2.4801666	7.45135706	471.98517	9.76E-09	1.87E-06
cadA	-4.0354085	8.47292228	446.491849	1.23E-08	2.26E-06
bacB	2.19065414	8.15019794	432.646634	1.40E-08	2.40E-06
menF	-2.3319914	7.77875967	431.616913	1.42E-08	2.40E-06
menD	-2.3528049	7.72883183	420.236956	1.58E-08	2.52E-06
sipW	4.99222524	3.79550895	419.286737	1.60E-08	2.52E-06
nrdI	-2.1604668	8.7158722	409.787881	1.76E-08	2.67E-06
yerA	-2.6121753	7.83272492	398.6252	1.97E-08	2.90E-06
epsI	3.17490107	4.2856941	392.805333	2.10E-08	2.97E-06
yttB	-2.968798	5.06051122	388.293628	2.20E-08	2.97E-06
motA	-2.5533393	7.9463994	384.880718	2.28E-08	2.97E-06
bpr	2.57510318	10.2034819	384.527984	2.29E-08	2.97E-06
B4U62_RS102	3.03126432	5.92238775	375.077649	2.54E-08	3.20E-06
yrrM	-2.7136385	5.84228084	372.590297	2.61E-08	3.20E-06
bacC	2.03639483	7.88558642	365.48388	2.83E-08	3.24E-06
bacA	2.39985097	7.82024574	364.881471	2.85E-08	3.24E-06
B4U62_RS117	2.62869334	7.02660056	364.32522	2.87E-08	3.24E-06
pfkB	-3.2213418	6.15603373	359.954549	3.02E-08	3.32E-06
epsN	3.64375311	4.95427436	348.899623	3.43E-08	3.62E-06
yvnB	3.09769813	8.56601054	346.910729	3.52E-08	3.62E-06

Table S5. Genes differentially expressed between ΔmnmE and WT in transcriptome analyses

fruR	-3.2580718	6.37528069	346.54297	3.53E-08	3.62E-06
yrrO	-1.8918319	7.49497761	343.887021	3.65E-08	3.65E-06
abrB	-2.2674418	6.99906716	340.987314	3.78E-08	3.70E-06
skfH	1.85265474	6.90989991	336.05299	4.01E-08	3.84E-06
yxbB	1.98862656	11.2357721	329.570176	4.35E-08	4.04E-06
czcD	-3.3992208	4.36720331	328.65645	4.40E-08	4.04E-06
kimA	-2.281549	8.2272588	327.066309	4.49E-08	4.04E-06
pchE	4.80856542	11.5615752	323.648343	4.69E-08	4.13E-06
yfmG	2.64354921	10.4970641	309.932345	5.61E-08	4.85E-06
ydfK	-3.3704569	4.61174109	307.726139	5.78E-08	4.90E-06
B4U62_RS184	3.00681323	4.80107104	302.181722	6.23E-08	5.18E-06
sprA	2.44319001	8.23167786	295.154706	6.87E-08	5.60E-06
narG	-3.0360334	9.87260326	292.322636	7.15E-08	5.73E-06
yvqJ	2.1059622	8.23512303	289.566948	7.44E-08	5.85E-06
braB	-2.5093459	5.59387021	283.54292	8.11E-08	6.17E-06
nfi	2.20342362	8.65628672	283.482409	8.12E-08	6.17E-06
ndhF	2.66262984	8.51340363	279.872481	8.56E-08	6.34E-06
ykwB	2.10402945	7.64553691	279.231847	8.64E-08	6.34E-06
metC	2.73584287	9.13702186	267.67941	1.03E-07	7.43E-06
epsG	3.02624955	4.12197766	262.040894	1.12E-07	7.97E-06
yfkN	1.98510087	9.3288777	261.097548	1.14E-07	7.97E-06
epsM	3.7430027	4.21548015	258.094476	1.20E-07	8.23E-06
ydgH	-2.2881732	6.95451084	251.205262	1.34E-07	9.06E-06
ydgG	-3.2590011	4.13743322	247.122912	1.43E-07	9.47E-06
udk	-1.643364	7.10742619	246.684158	1.44E-07	9.47E-06
yxbA	1.99212394	9.85146603	244.663011	1.49E-07	9.59E-06
serS	-1.8324224	7.51318156	243.779492	1.51E-07	9.59E-06
B4U62_RS184	3.37380524	5.16796319	243.334848	1.52E-07	9.59E-06
yvpB	-1.9012623	6.48113566	242.04092	1.56E-07	9.66E-06
narI	-2.4549484	7.90945637	238.824285	1.65E-07	9.88E-06
asnH	2.32805419	11.3611767	238.690379	1.65E-07	9.88E-06
rlmG	-1.7755998	7.04650033	238.304614	1.66E-07	9.88E-06
narH	-2.7196556	8.90466529	235.147373	1.75E-07	1.03E-05
nrdE	-1.7756078	10.7569129	233.053236	1.82E-07	1.04E-05
ywqJ	2.10714096	9.17020666	232.373649	1.84E-07	1.04E-05
fnr	-2.2495684	7.36960686	232.109491	1.85E-07	1.04E-05
skfB	2.13508884	7.20112135	230.670384	1.90E-07	1.06E-05
srfAD	1.73294539	7.29801166	228.571528	1.97E-07	1.09E-05
epsL	3.60835157	4.08629827	227.399854	2.01E-07	1.09E-05
yrrN	-2.5395503	6.22882048	225.938574	2.07E-07	1.11E-05
skfG	1.76237524	6.33568302	223.316748	2.17E-07	1.11E-05
mscC	-2.3289167	6.3221257	222.580134	2.20E-07	1.11E-05
bkdR	-1.6774751	6.69737268	221.820362	2.23E-07	1.11E-05
wprA	1.73477145	8.93953688	221.797268	2.23E-07	1.11E-05
sigF	1.51195939	6.9752549	220.764556	2.27E-07	1.11E-05

Table S5. Genes differentially expressed between ΔmnmE and WT in transcriptome analyses

epsF	2.85091384	4.17415662	220.532469	2.28E-07	1.11E-05
narJ	-2.7029251	7.83307227	220.501516	2.28E-07	1.11E-05
vpr	2.48474966	8.67016453	220.452424	2.29E-07	1.11E-05
nrdF	-1.6345352	9.61187433	220.333997	2.29E-07	1.11E-05
psmB	-3.2433377	5.03248601	215.770107	2.50E-07	1.20E-05
pdaC	-1.8562353	7.73427628	210.057683	2.79E-07	1.31E-05
mneP	-2.1747957	5.43259096	210.005783	2.79E-07	1.31E-05
bacE	1.84062329	7.96812108	207.997646	2.90E-07	1.35E-05
bacF	1.75875905	7.64703878	207.458173	2.93E-07	1.35E-05
opuCB	1.74624488	8.42298598	205.710165	3.04E-07	1.38E-05
rttO	1.98719869	7.81763998	204.580041	3.10E-07	1.40E-05
epsH	3.06929157	3.71389037	203.19522	3.19E-07	1.40E-05
thrC	2.00424167	11.1516308	202.601505	3.23E-07	1.40E-05
bacD	1.90053141	8.74105211	202.528268	3.24E-07	1.40E-05
epsA	2.6710214	4.35267371	202.382899	3.24E-07	1.40E-05
pchR	4.76697937	9.65949828	201.592533	3.30E-07	1.41E-05
menH	-1.5544236	7.1418984	196.975704	3.62E-07	1.53E-05
wapA	1.34807664	12.2448346	196.335184	3.67E-07	1.53E-05
yxaM	2.59255235	10.2821519	196.305374	3.68E-07	1.53E-05
skfC	1.92361408	8.05572026	194.007262	3.86E-07	1.59E-05
yxbC	1.40480277	11.0221472	193.467468	3.90E-07	1.59E-05
trmFO	-1.6844516	6.29683661	189.68171	4.23E-07	1.71E-05
nprB	1.97322826	5.39805623	189.070803	4.28E-07	1.71E-05
opuCC	1.57258451	8.98730971	188.885682	4.30E-07	1.71E-05
yclJ	-1.5296065	6.14063806	188.119106	4.37E-07	1.72E-05
ybcC	2.40278763	9.72207298	185.478917	4.63E-07	1.81E-05
hag	-1.4920094	11.8758794	184.643183	4.72E-07	1.82E-05
yitM	2.23176504	7.25010648	182.724692	4.92E-07	1.88E-05
spoIIAB	1.43615027	6.27889403	182.497177	4.95E-07	1.88E-05
pyc	1.5484669	10.5745079	181.537836	5.06E-07	1.90E-05
mtnK	3.89932975	8.62846734	179.295251	5.32E-07	1.97E-05
B4U62_RS221	1.7086186	7.17689127	179.218192	5.33E-07	1.97E-05
ssbA	-1.3227487	9.95028648	174.822885	5.89E-07	2.16E-05
yhjC	1.58606867	6.88621239	174.218327	5.98E-07	2.18E-05
dacA	-2.2996699	7.64791178	173.306733	6.11E-07	2.20E-05
bcaP	1.92221811	8.8147797	172.575842	6.21E-07	2.22E-05
opuCA	1.56142945	9.47586753	170.539688	6.52E-07	2.32E-05
yhjH	4.0454657	9.51639819	169.360413	6.71E-07	2.36E-05
yfmT	-1.9908403	8.71266407	167.417165	7.03E-07	2.46E-05
panB	-1.3794284	7.62140847	166.260939	7.23E-07	2.51E-05
xylA	2.39546418	6.86244183	165.921868	7.29E-07	2.51E-05
frlO	1.69542676	11.3982571	165.142874	7.43E-07	2.54E-05
artP	1.48895591	9.72783987	163.441118	7.75E-07	2.62E-05
kinB	1.35824419	6.64111623	163.15967	7.80E-07	2.62E-05
B4U62_RS230	1.45141148	8.29947036	162.888448	7.85E-07	2.62E-05

Table S5. Genes differentially expressed between ΔmnmE and WT in transcriptome analyses

yrdP	-2.864838	4.62275722	162.608513	7.91E-07	2.62E-05
srfT	1.56318968	7.23675393	161.382394	8.15E-07	2.68E-05
ypzJ	1.75035018	5.02640345	158.094637	8.86E-07	2.89E-05
pgdS	-1.6916444	7.86310083	157.708619	8.95E-07	2.90E-05
hom	2.02765786	10.6570574	157.249277	9.06E-07	2.91E-05
srfAB2	1.48330049	10.2534938	156.669778	9.19E-07	2.93E-05
yrhP	-2.1818266	4.45580875	155.488377	9.48E-07	3.00E-05
ahpF	1.37913952	11.9665388	155.241066	9.54E-07	3.00E-05
artR	1.7014447	9.32284217	154.797458	9.65E-07	3.01E-05
yqgE	-2.1025488	4.98235193	154.606548	9.70E-07	3.01E-05
metI	2.42703064	8.3475032	154.235801	9.80E-07	3.02E-05
tyrZ	2.50137672	4.47303063	152.542106	1.02E-06	3.13E-05
thrB	2.02070356	10.7537527	152.258611	1.03E-06	3.14E-05
artQ	1.81315591	9.09951481	151.989571	1.04E-06	3.14E-05
pyrP	-1.4753292	7.2454821	151.157967	1.06E-06	3.17E-05
mtnA	3.41239605	8.61300519	151.027134	1.07E-06	3.17E-05
xerC	-1.3944267	8.6464817	150.818895	1.07E-06	3.17E-05
skfF	1.77795879	7.90991946	149.722874	1.10E-06	3.23E-05
ilvC	1.48570794	10.5941827	149.52933	1.11E-06	3.23E-05
ywqI	1.95406533	6.25618649	149.328081	1.12E-06	3.23E-05
ilvE	1.44439773	6.65406501	148.906345	1.13E-06	3.25E-05
ybcI	1.98682242	6.41053238	148.048671	1.16E-06	3.31E-05
B4U62_RS012	1.78392774	7.82757074	147.089538	1.19E-06	3.34E-05
epsO	3.50261966	4.49767782	147.044938	1.19E-06	3.34E-05
samT	2.65357838	9.03021879	146.949377	1.19E-06	3.34E-05
dagK	-1.3105516	6.96443993	146.663217	1.20E-06	3.35E-05
pksJ	1.3703305	10.4372362	145.63758	1.23E-06	3.40E-05
pyrR	-2.6070335	7.8375458	145.599621	1.24E-06	3.40E-05
B4U62_RS231	-1.9780616	5.21855969	145.270039	1.25E-06	3.41E-05
purA	-1.6254742	9.23035417	144.349489	1.28E-06	3.48E-05
srfAB1	1.34220039	9.13603543	144.086464	1.29E-06	3.48E-05
yrbE	-1.9821322	4.93307074	143.19628	1.32E-06	3.55E-05
swrA	-1.9404094	6.44085822	142.899487	1.33E-06	3.56E-05
tatCY	-1.2512779	9.9722059	141.715175	1.38E-06	3.65E-05
leuB	1.61264449	10.0808086	141.617349	1.38E-06	3.65E-05
ylnD	1.86700239	8.65604235	141.027746	1.41E-06	3.68E-05
opuBB	2.02887484	5.37456618	140.880308	1.41E-06	3.68E-05
motB	-2.1946744	7.91117971	140.599564	1.42E-06	3.69E-05
hrcA	-1.3877941	8.33926979	140.229352	1.44E-06	3.70E-05
B4U62_RS061	2.71003745	5.11435898	139.694021	1.46E-06	3.74E-05
ycgJ	2.57425733	3.98668012	138.787447	1.50E-06	3.80E-05
ilvB	1.74221357	10.9812916	138.778696	1.50E-06	3.80E-05
B4U62_RS134	-1.4647565	6.84520767	138.513438	1.51E-06	3.80E-05
srfAC	1.46375653	9.57429828	137.548095	1.55E-06	3.89E-05
pdeH	-1.3202059	6.71464044	137.346003	1.56E-06	3.89E-05

Table S5. Genes differentially expressed between ΔmnmE and WT in transcriptome analyses

yjlC	-1.334509	8.9301408	137.04689	1.58E-06	3.90E-05
leuA	1.75573522	10.7919644	136.759911	1.59E-06	3.90E-05
arfM	-1.8241735	4.63402272	136.66265	1.60E-06	3.90E-05
mltG	-1.2283415	6.91066658	136.433812	1.61E-06	3.91E-05
B4U62_RS214:	2.11800406	9.98932693	135.715253	1.64E-06	3.97E-05
mtnD	2.0142137	7.40677263	134.781492	1.69E-06	4.05E-05
clpQ	-1.412031	8.09524847	134.720184	1.69E-06	4.05E-05
mtnX	2.14936749	8.35010405	134.113067	1.72E-06	4.10E-05
pxpG	2.32799686	6.07532013	133.597632	1.75E-06	4.14E-05
yfmI	1.9477874	6.19569167	132.268631	1.82E-06	4.27E-05
leuC	1.1673208	10.3128962	132.219019	1.82E-06	4.27E-05
mcpB	-1.5866693	7.39979153	131.853828	1.84E-06	4.29E-05
mtnW	2.15803715	8.15614334	131.642057	1.85E-06	4.29E-05
kinE	1.20337829	8.45064464	131.533727	1.86E-06	4.29E-05
pxpA	2.2101158	5.36893025	131.339507	1.87E-06	4.29E-05
gerPE	1.99733619	2.93425923	130.960222	1.89E-06	4.32E-05
ydhE	-1.1699539	6.81872814	130.76126	1.90E-06	4.32E-05
mcpC	-1.4071355	8.27519245	130.096253	1.94E-06	4.38E-05
hemE	-1.7863828	9.23385902	129.937547	1.95E-06	4.38E-05
pbpA	-1.2019535	8.55067412	129.831511	1.96E-06	4.38E-05
mtnB	2.02619631	7.82524288	129.70108	1.97E-06	4.38E-05
murJ	-1.1556175	7.88082296	129.125004	2.00E-06	4.39E-05
frlB	1.64183756	12.0025796	128.660717	2.03E-06	4.39E-05
frlM	1.79005649	9.84732214	128.582864	2.04E-06	4.39E-05
rpsJ	-1.1704102	9.37780286	128.5073	2.04E-06	4.39E-05
tlpC	-1.6658798	4.97347153	128.492623	2.04E-06	4.39E-05
rsmJ	-1.6194459	5.46897838	128.447576	2.05E-06	4.39E-05
lytG	-1.6300994	5.78858102	128.243751	2.06E-06	4.39E-05
B4U62_RS210:	1.72692339	6.68843899	128.161734	2.06E-06	4.39E-05
skfE	1.34401499	7.4124396	128.141881	2.07E-06	4.39E-05
B4U62_RS210	1.6371955	5.8380085	127.731755	2.09E-06	4.43E-05
xylB	1.96021734	5.85940476	127.515614	2.11E-06	4.44E-05
frlD	1.70918066	9.74731114	127.247245	2.12E-06	4.46E-05
frlN	1.76335307	10.1764772	125.570856	2.24E-06	4.62E-05
srfAA	1.27660012	10.1798994	125.347328	2.26E-06	4.62E-05
yxjG	1.63161127	9.69574294	125.335093	2.26E-06	4.62E-05
opuCD	1.45295175	8.73301197	125.266342	2.26E-06	4.62E-05
yclK	-1.4118707	7.53447778	125.248933	2.26E-06	4.62E-05
gamA	-1.5195464	4.45452159	125.162529	2.27E-06	4.62E-05
yoeC	-2.059898	5.09411458	125.076839	2.28E-06	4.62E-05
thrD	1.83979199	8.63677852	124.768347	2.30E-06	4.64E-05
bmr3	1.60606421	6.07103423	124.626408	2.31E-06	4.64E-05
dhbF	3.1031271	11.7514492	124.456751	2.32E-06	4.65E-05
cidB	-2.0435074	6.45683999	124.025815	2.35E-06	4.69E-05
comS	1.49311511	5.87341724	123.147727	2.42E-06	4.80E-05

Table S5. Genes differentially expressed between ΔmnmE and WT in transcriptome analyses

rplJ	-1.20274	10.1692503	122.928807	2.44E-06	4.80E-05
yvnA	1.42664147	7.36404335	122.876121	2.44E-06	4.80E-05
treR	-1.4120941	5.65660543	122.494575	2.47E-06	4.84E-05
cysH	1.58398455	9.41789755	122.228416	2.49E-06	4.86E-05
ktrA	-1.570844	4.97888047	121.891933	2.52E-06	4.89E-05
ackA	-1.631699	7.37950694	121.699891	2.54E-06	4.90E-05
cwlQ	-1.4298887	8.16667491	121.202329	2.58E-06	4.96E-05
B4U62_RS210	1.49014445	6.28288648	120.972334	2.60E-06	4.96E-05
psmA	-3.1708467	4.87459165	120.96973	2.60E-06	4.96E-05
tatAY	-1.3926335	8.99200701	120.761791	2.62E-06	4.97E-05
gltP	1.43847511	5.99156648	120.03211	2.68E-06	5.05E-05
isp	3.62799547	8.50972048	120.012892	2.68E-06	5.05E-05
pelC	-1.9133011	4.30660019	119.202688	2.76E-06	5.17E-05
tkmA	-1.2335799	7.48361385	118.848683	2.79E-06	5.21E-05
hemH	-1.5994742	9.71242345	118.460267	2.83E-06	5.25E-05
yjnA	2.43164325	5.44889961	118.072659	2.86E-06	5.30E-05
dppE	1.7509093	11.0561657	117.850335	2.89E-06	5.30E-05
dgcK	-1.3769918	5.72758901	117.83246	2.89E-06	5.30E-05
ysmB	-1.2710887	6.92190423	117.711652	2.90E-06	5.30E-05
ybcF	2.17616743	7.79015315	117.323481	2.94E-06	5.35E-05
gntK	-1.4654884	5.51673305	117.038948	2.97E-06	5.38E-05
yhjN	1.63426479	6.10674443	116.698318	3.00E-06	5.41E-05
iseA	-2.9565631	10.1849223	116.510397	3.02E-06	5.41E-05
yocS	1.63771311	7.94394986	116.508747	3.02E-06	5.41E-05
lytF	-1.4442004	8.32199106	116.108022	3.06E-06	5.46E-05
yqzC	-1.3033491	7.49298609	115.631376	3.11E-06	5.51E-05
essA	1.49559138	7.11296315	115.629319	3.11E-06	5.51E-05
pfkA	-1.2804062	8.4388487	114.7173	3.21E-06	5.66E-05
mtnU	2.06270376	6.8892362	114.620933	3.22E-06	5.66E-05
yhbJ	-1.2935794	5.84198105	114.319665	3.26E-06	5.69E-05
tapA	4.68880258	3.13701327	113.967684	3.30E-06	5.74E-05
yutJ	1.59508585	8.97077343	112.634482	3.46E-06	5.99E-05
ppuG	1.14885614	6.27480031	111.835438	3.55E-06	6.14E-05
B4U62_RS229	-2.6983256	3.18072823	110.20409	3.77E-06	6.48E-05
yfmS	-1.7495748	8.25441783	109.66381	3.84E-06	6.59E-05
ybcH	2.03262128	6.85232705	109.446269	3.87E-06	6.61E-05
B4U62_RS101	1.16570901	5.99618204	108.971874	3.94E-06	6.67E-05
opuBA	2.11146572	6.78385177	108.938269	3.94E-06	6.67E-05
silP	-1.6804001	8.7872509	108.878643	3.95E-06	6.67E-05
bznD	1.15560286	8.13751324	108.092093	4.07E-06	6.84E-05
yhjG	4.39660142	11.0244354	112.767219	4.15E-06	6.95E-05
B4U62_RS029	1.27386411	6.60084936	107.274028	4.19E-06	7.00E-05
ahpC	1.25411691	10.6927504	106.699433	4.28E-06	7.12E-05
bcrC	1.32258951	7.01903787	106.497013	4.32E-06	7.12E-05
fabHB	-2.1076394	5.53423415	106.389308	4.33E-06	7.12E-05

Table S5. Genes differentially expressed between ΔmnmE and WT in transcriptome analyses

yjcL	1.23912565	6.34538274	106.344041	4.34E-06	7.12E-05
mcpA	-1.7344921	8.95095753	106.305647	4.35E-06	7.12E-05
B4U62_RS094	-1.3771322	5.87462486	106.07393	4.38E-06	7.15E-05
cssR	-1.2945929	5.88107901	105.716317	4.44E-06	7.22E-05
bsnA	1.51893814	5.87523787	105.640041	4.46E-06	7.22E-05
dppC	1.86569027	10.3194926	105.147308	4.54E-06	7.32E-05
yfjR	1.24727191	5.60922525	105.000817	4.56E-06	7.33E-05
B4U62_RS109	-2.2239767	3.47095704	104.916352	4.58E-06	7.33E-05
dppA	1.54082484	10.4142084	104.79057	4.60E-06	7.34E-05
yhcR	1.18184095	8.0831556	104.446213	4.66E-06	7.41E-05
feuC	1.87345354	8.65975677	104.109264	4.72E-06	7.48E-05
kinA	1.12162054	7.59143209	103.171245	4.89E-06	7.72E-05
feuB	1.74088306	9.37842307	103.012844	4.92E-06	7.74E-05
narK	-1.7360205	6.56229679	102.773507	4.97E-06	7.77E-05
mmuP	2.53788446	6.57134043	102.728161	4.98E-06	7.77E-05
cysP	1.81981046	9.3163912	102.463493	5.03E-06	7.77E-05
ctaA	-2.0562145	9.79959542	102.45127	5.03E-06	7.77E-05
spoIIAA	1.35867085	5.45656342	102.449919	5.03E-06	7.77E-05
cssS	-1.1947745	6.84341114	101.596288	5.20E-06	8.01E-05
queD	-1.2893507	5.4729583	101.188166	5.28E-06	8.11E-05
argC	1.30581963	9.88890089	100.809976	5.36E-06	8.14E-05
ywqH	1.91839537	6.04337777	100.804832	5.36E-06	8.14E-05
dppD	1.79839507	10.3909492	100.75142	5.37E-06	8.14E-05
epsE	2.17192631	3.62813692	100.736343	5.38E-06	8.14E-05
ldcB	-1.9756261	4.52307817	100.385809	5.45E-06	8.22E-05
sufB	1.05804458	11.0251219	100.100921	5.51E-06	8.27E-05
gudB	-1.2202762	9.75645095	100.077692	5.52E-06	8.27E-05
murE	-1.1489846	7.49347872	99.8538809	5.57E-06	8.31E-05
ybbC	1.46725091	5.53276482	99.7747841	5.58E-06	8.31E-05
yhbI	-1.8265767	4.93497902	99.1693359	5.72E-06	8.46E-05
metN	1.44377421	7.04357859	99.1523109	5.72E-06	8.46E-05
yvfG	-1.7545548	3.99807288	98.6214021	5.85E-06	8.61E-05
mtnE	1.7642563	6.98720008	98.1386886	5.96E-06	8.75E-05
yvaQ	-1.1446771	6.01576727	97.493495	6.12E-06	8.95E-05
sat	2.21065006	8.86633323	96.7839295	6.29E-06	9.15E-05
rnhB	-1.4397201	6.49739294	96.7318396	6.31E-06	9.15E-05
cypX	2.13209218	12.3638757	96.7141888	6.31E-06	9.15E-05
pxpI	2.3319575	5.23175602	96.590092	6.34E-06	9.15E-05
fliD	-1.5824453	8.20017194	96.3842607	6.40E-06	9.15E-05
opuBD	1.67897795	4.93526908	96.3738499	6.40E-06	9.15E-05
yeeF	1.31485204	7.32373038	96.3712014	6.40E-06	9.15E-05
epsB	2.64716647	4.42607038	96.0594752	6.48E-06	9.24E-05
panD	-1.0792257	6.31564389	95.3830699	6.67E-06	9.47E-05
eesA	1.89942272	7.74705861	95.228155	6.71E-06	9.50E-05
yojN	-1.1038323	5.96918499	94.9029653	6.80E-06	9.60E-05

Table S5. Genes differentially expressed between ΔmnmE and WT in transcriptome analyses

yobE	1.11375069	6.21936847	94.6072049	6.88E-06	9.66E-05
glcT	-1.2789921	6.59333885	94.606523	6.88E-06	9.66E-05
bdhA	-1.2042892	11.3191863	94.3185934	6.97E-06	9.73E-05
ydhB	-1.643556	4.71487126	94.2681507	6.98E-06	9.73E-05
ctaB	-1.3288486	9.66288743	94.1298921	7.02E-06	9.76E-05
B4U62_RS210(	1.6666391	6.27287733	93.9556002	7.07E-06	9.79E-05
yczC	1.87883085	3.66732003	93.8949217	7.09E-06	9.79E-05
panC	-1.1160833	7.45554375	93.7970357	7.12E-06	9.79E-05
B4U62_RS103(	1.6273695	7.03155612	93.7462583	7.13E-06	9.79E-05
dppB	1.77389248	10.7765555	93.5184937	7.20E-06	9.85E-05
yocC	-1.9095161	3.76429198	93.2314815	7.29E-06	9.94E-05
ldcA	1.65651952	8.68587862	92.5964603	7.49E-06	0.00010181
yojO	-1.0847901	6.615492	92.4919665	7.52E-06	0.00010195
conE	2.19071917	5.94747959	91.9929762	7.68E-06	0.00010381
yqcF	1.05930504	7.1869777	91.8432428	7.73E-06	0.00010407
mazG	-1.0112522	7.04562677	91.7911404	7.75E-06	0.00010407
rluD	-1.152193	6.2141963	91.6018608	7.81E-06	0.0001046
yhjB	1.86696332	9.14505636	91.2507786	7.93E-06	0.00010586
mnmG	-1.0138521	8.20096957	91.0578658	8.00E-06	0.00010621
pyrB	-1.9560982	5.4622405	91.0348708	8.00E-06	0.00010621
ydaB	-1.0414499	6.38521025	90.5836856	8.16E-06	0.00010797
eepC	1.65859366	8.58809195	90.4060257	8.22E-06	0.00010832
ykfD	1.73175073	8.98811537	90.3613549	8.24E-06	0.00010832
ypiB	1.21630899	8.29141603	90.2697531	8.27E-06	0.00010832
ylxM	-1.0129464	9.02173747	90.2315334	8.29E-06	0.00010832
maeL	-0.9936737	6.68947807	89.2940749	8.63E-06	0.00011185
trmL	-1.2821362	6.06607478	89.0935832	8.71E-06	0.00011185
trpA	1.21327776	9.45427515	89.0233183	8.74E-06	0.00011185
yvbW	1.4320643	5.40240369	89.0069321	8.74E-06	0.00011185
yxkH	-2.2541227	4.73595138	88.9849847	8.75E-06	0.00011185
yneA	-1.2534637	5.4235482	88.9758082	8.75E-06	0.00011185
besA	1.75683784	7.47616561	88.9730143	8.76E-06	0.00011185
fliS	-1.4717307	6.92764893	88.9593379	8.76E-06	0.00011185
ktrB	-1.2354643	5.64085135	88.8608186	8.80E-06	0.00011202
yxjH	1.68966414	8.33888401	88.7267046	8.85E-06	0.00011235
ybfO	1.789469	8.37848531	88.2678361	9.03E-06	0.00011432
B4U62_RS087!	-1.272468	7.69730508	88.1874897	9.06E-06	0.0001144
steT	-1.361267	5.06237137	87.9887755	9.14E-06	0.00011508
rlmKB	-1.0164693	6.80491608	87.7849143	9.23E-06	0.00011573
skiZ	1.07195094	8.61709412	87.734638	9.25E-06	0.00011573
trpE	1.45686998	9.83935501	87.6673311	9.28E-06	0.00011575
gltC	1.1372405	7.39835042	87.5527125	9.32E-06	0.00011601
yoaB	2.94734524	5.57383526	87.4237875	9.38E-06	0.00011635
cgeD	-1.5022689	4.70894766	87.3551552	9.41E-06	0.00011638
kapB	1.54000582	4.97233365	87.1881555	9.48E-06	0.00011693

Table S5. Genes differentially expressed between ΔmnmE and WT in transcriptome analyses

mbtH	2.85856663	5.82860009	86.9974008	9.56E-06	0.0001176
ytvB	1.64451225	5.65982237	86.7824229	9.65E-06	0.00011841
yjoA	-1.1661181	5.81375128	86.6639629	9.70E-06	0.00011862
B4U62_RS210	1.63985297	6.30375371	86.6193388	9.72E-06	0.00011862
gltB	1.31314499	12.2996521	86.0908759	9.96E-06	0.00012115
rapI	1.48731603	5.56463063	85.9777721	1.00E-05	0.00012133
srtA	1.15027301	5.9853923	85.9375408	1.00E-05	0.00012133
cah	1.96845887	5.02268418	85.8652051	1.01E-05	0.00012139
queC	-1.1876875	5.76402734	85.7596099	1.01E-05	0.00012164
tagC	-1.5725036	6.1425577	85.5176293	1.02E-05	0.00012265
B4U62_RS161	-1.012144	6.72672796	85.3890696	1.03E-05	0.00012304
fldP	3.39109794	6.07297487	85.2485849	1.03E-05	0.0001235
dhbA	2.68082751	7.76328569	85.1193916	1.04E-05	0.0001236
tcyP	1.53837816	5.47385768	85.1121772	1.04E-05	0.0001236
ndh	-1.2476757	10.4318457	84.7500412	1.06E-05	0.00012533
ybxG	0.98247152	7.36668031	84.150251	1.09E-05	0.00012818
yxiJ	1.65407059	5.00406032	84.1453552	1.09E-05	0.00012818
yoeA	0.98790437	8.18510903	83.7046034	1.11E-05	0.00013048
ythQ	1.28945873	8.06880691	83.5587268	1.12E-05	0.00013102
dinL	-1.1362131	6.10194187	83.3872991	1.13E-05	0.00013125
rapF	1.37969844	8.39277505	83.3625948	1.13E-05	0.00013125
leuD	1.09272599	9.00855146	83.3496785	1.13E-05	0.00013125
miaB	-0.9828763	8.49768155	82.9129391	1.15E-05	0.00013341
murI	-1.232942	5.04730713	82.8885093	1.15E-05	0.00013341
yvkC	1.57703351	5.74672008	82.8174908	1.16E-05	0.00013351
dhbC	2.71811891	8.60211532	82.6476397	1.17E-05	0.00013422
ykuO	3.27362402	7.89531579	82.4986286	1.18E-05	0.00013482
conB	2.25092092	5.17353151	82.3628166	1.18E-05	0.00013533
B4U62_RS190	-1.2686634	6.71624077	82.2127358	1.19E-05	0.00013594
hemQ	-1.5652769	10.2673666	82.1426016	1.20E-05	0.00013604
mreBH	-1.4975955	4.77988883	82.00649	1.20E-05	0.00013644
ugpC	1.35190643	8.89526633	81.9719464	1.20E-05	0.00013644
sdaAB	-1.1962184	5.56521675	81.57363	1.23E-05	0.00013868
ilvN	1.61361414	9.04119925	81.4042721	1.24E-05	0.00013945
dgrA	-1.5021381	4.48055529	80.9523667	1.26E-05	0.00014213
rplL	-1.1982386	9.8012596	80.5135802	1.29E-05	0.00014453
queF	-1.1351382	6.02588206	80.4215062	1.30E-05	0.00014453
yxiI	1.4080623	5.68393686	80.4054998	1.30E-05	0.00014453
refZ	1.5497969	4.639842	80.393456	1.30E-05	0.00014453
yjhB	1.96158516	4.39105133	80.2228506	1.31E-05	0.00014518
gntR	-1.6404758	3.86157373	80.1827689	1.31E-05	0.00014518
queE	-1.4508357	5.67017797	80.0599272	1.32E-05	0.00014518
folE2	0.97229956	8.8624705	80.0384696	1.32E-05	0.00014518
ilvA	1.06705016	9.39885192	80.0236356	1.32E-05	0.00014518
cysK	1.54625223	9.10188451	79.9886316	1.32E-05	0.00014518

Table S5. Genes differentially expressed between ΔmnmE and WT in transcriptome analyses

flhP	-1.2440397	7.41795592	79.866661	1.33E-05	0.00014568
dhbB	2.85154935	8.30614031	79.6032141	1.35E-05	0.00014719
metP	1.50869503	7.16061355	79.2208099	1.38E-05	0.00014959
dnaE	-0.9483276	9.33894303	78.9755399	1.39E-05	0.00015102
yloC	-0.8995563	9.22121672	78.7570739	1.41E-05	0.00015187
yxiC	1.69915137	5.18998705	78.7090897	1.41E-05	0.00015187
flaG	-1.7342761	5.43018258	78.6790254	1.41E-05	0.00015187
xis	1.96100043	3.86383953	78.6616171	1.41E-05	0.00015187
ywcE	2.22830763	4.43810488	78.5741643	1.42E-05	0.00015216
katA	1.77749264	8.27596492	78.4853517	1.43E-05	0.00015218
resA	-1.6214528	7.09627362	78.4460059	1.43E-05	0.00015218
yxbD	1.82376564	6.66907727	78.4103506	1.43E-05	0.00015218
pdhD	-1.0406804	10.066325	78.3383095	1.44E-05	0.00015218
B4U62_RS139(	2.13476209	8.67372294	78.3259833	1.44E-05	0.00015218
yvyE	-1.5558496	5.21818423	78.1540958	1.45E-05	0.00015305
rncS	-1.2994935	7.60100575	78.1138873	1.45E-05	0.00015305
iscSA	-1.0624605	7.98040735	78.0468171	1.46E-05	0.00015319
ykfB	1.65289138	8.84492215	77.8653968	1.47E-05	0.00015418
B4U62_RS190:	-1.1140699	6.48783065	77.8208267	1.47E-05	0.00015418
recU	-0.9463651	7.10967973	77.5288745	1.50E-05	0.00015607
ylmA	1.20665604	6.52768444	77.4194921	1.50E-05	0.00015655
ykvZ	-1.3540753	4.53102207	76.7602905	1.55E-05	0.00016143
yoaC	2.49349329	6.38096117	76.3619784	1.59E-05	0.00016431
ycgA	0.9562034	7.13609411	76.065688	1.61E-05	0.0001664
liaM	1.51980567	8.34347394	75.8068949	1.63E-05	0.00016821
mblK	1.06243513	7.65773255	75.7444451	1.64E-05	0.00016835
yxaL	-2.0014753	9.10666346	75.6262971	1.65E-05	0.00016897
hemY	-1.2487102	9.70728915	75.5018842	1.66E-05	0.00016926
yknY	1.02433914	8.32221103	75.4653102	1.66E-05	0.00016926
nicK	2.12994185	6.00631321	75.418791	1.66E-05	0.00016926
appC	1.23243269	9.41089158	75.3884396	1.67E-05	0.00016926
ltaSC	-1.0193654	9.38405378	75.3667018	1.67E-05	0.00016926
yhdX	1.54382155	6.76175932	75.1743668	1.68E-05	0.00017047
gerPC	1.5104149	2.81743751	75.1346905	1.69E-05	0.00017047
yoaD	2.42970178	5.83996469	75.0925795	1.69E-05	0.00017047
sdpI	1.20065804	10.3592978	74.9663338	1.70E-05	0.00017119
feuA	1.63517503	9.84201051	74.8130403	1.72E-05	0.00017215
tdh	-1.5770173	5.0326387	74.7139552	1.72E-05	0.00017256
murF	-0.9985888	7.12267796	74.6780631	1.73E-05	0.00017256
yocM	1.4225415	5.74186826	74.0914893	1.78E-05	0.00017748
yjiB	0.99375419	6.02386649	73.9506839	1.79E-05	0.00017838
liaE	1.86215323	8.35674226	73.9064605	1.80E-05	0.00017839
ylbN	-1.0951841	8.72473652	73.7660256	1.81E-05	0.0001793
cidA	-2.4812013	4.78315222	73.6914393	1.82E-05	0.00017959
yuiB	1.40103629	11.0366647	73.5083014	1.84E-05	0.00018067

Table S5. Genes differentially expressed between ΔmnmE and WT in transcriptome analyses

gerPD	1.74038137	2.34933134	73.4911705	1.84E-05	0.00018067
lspA	-1.4147001	4.66284224	73.4393335	1.84E-05	0.00018076
hemAT	-1.1889965	7.70004704	73.131621	1.87E-05	0.00018329
ysdB	1.24305642	7.99547855	72.5784987	1.93E-05	0.0001883
yozB	-1.7233533	8.60018472	72.4844032	1.94E-05	0.00018882
hfq	1.17929973	7.51990748	72.0683662	1.98E-05	0.00019261
B4U62_RS192	2.17416752	7.44598758	71.9659607	1.99E-05	0.00019324
B4U62_RS226	1.33393591	5.60493798	71.8292473	2.01E-05	0.00019423
guaD	1.95509475	4.14898134	71.7260391	2.02E-05	0.00019452
sirC	1.47881019	7.88852726	71.7190476	2.02E-05	0.00019452
conD	2.44327123	3.98487004	71.6298065	2.03E-05	0.00019503
ald	-1.0316511	9.38987051	71.5428839	2.04E-05	0.00019551
conC	2.43096362	3.4169747	71.2804155	2.07E-05	0.00019785
pckA	1.71676345	6.67880502	71.1120279	2.08E-05	0.00019922
dhbE	2.85646944	8.75395387	71.0355336	2.09E-05	0.00019961
yfmJ	0.97486461	7.25837709	70.9483107	2.10E-05	0.00020012
sufS	0.94888111	10.4358	70.8861994	2.11E-05	0.00020036
phrF	1.2421155	5.42421353	70.7169053	2.13E-05	0.00020177
yciB	0.92365714	8.2951786	70.3098644	2.18E-05	0.00020556
cheV	-1.1035241	6.99390295	70.2952454	2.18E-05	0.00020556
appB	1.18743131	9.33774729	70.0899968	2.20E-05	0.00020743
yvjA	-1.8172157	4.41438656	69.8790492	2.23E-05	0.00020932
gltD	1.05419207	10.5925125	69.8467589	2.23E-05	0.00020932
scpA	-1.4090023	4.67349347	69.800907	2.24E-05	0.0002094
nrnA	0.93667628	5.80179238	69.6944605	2.25E-05	0.00021018
B4U62_RS028	2.29072273	3.97837386	69.4533408	2.28E-05	0.00021225
B4U62_RS227	2.07804338	4.23446566	69.4388945	2.28E-05	0.00021225
esaA	1.25198253	9.06181237	69.3798705	2.29E-05	0.0002125
mleN	-1.6868986	4.89547322	69.3415821	2.30E-05	0.0002125
murP	1.15344491	5.82116074	69.281245	2.30E-05	0.00021276
B4U62_RS210	1.32905167	5.41276924	69.2024989	2.31E-05	0.00021324
trpC	1.45839047	8.8265056	69.1381403	2.32E-05	0.00021355
gltT	0.92865978	10.0445328	69.0038896	2.34E-05	0.0002147
ydjE	-1.0366445	7.43453209	68.809927	2.36E-05	0.00021657
yokF	-1.2834425	4.62103512	68.2362434	2.44E-05	0.00022313
cwlT	2.69078421	4.11591041	68.2003417	2.45E-05	0.00022313
yxiE	-2.2728705	8.19014749	68.0496178	2.47E-05	0.00022456
flhO	-1.4031876	7.56561772	67.8337495	2.50E-05	0.00022683
yozQ	1.615255	3.52583823	67.6893262	2.52E-05	0.00022821
yitP	2.79559281	3.9460705	67.4854007	2.55E-05	0.00023038
ychF	-1.1697127	6.73024462	67.3808134	2.56E-05	0.00023127
pksF	1.48174656	7.04816938	67.3117667	2.57E-05	0.0002317
ffh	-0.8362843	10.5597084	67.246986	2.58E-05	0.00023208
remA	-0.8567606	8.22439008	67.1849351	2.59E-05	0.00023233
adhA	-1.2541884	4.14518643	67.1269923	2.60E-05	0.00023233

Table S5. Genes differentially expressed between ΔmnmE and WT in transcriptome analyses

scoC	1.20892952	8.9924147	67.1205738	2.60E-05	0.00023233
yhdH	-0.9522664	5.78415089	67.0563242	2.61E-05	0.00023246
yoaH	-1.7572255	5.16549211	67.0161968	2.62E-05	0.00023246
rapH	0.82937072	8.93613932	67.0039353	2.62E-05	0.00023246
pksI	1.47389758	6.30760911	66.8802036	2.64E-05	0.00023363
B4U62_RS077:	-1.8879143	4.43433572	66.6783217	2.67E-05	0.00023586
mtlA	-2.5265376	4.48037834	66.62273	2.67E-05	0.00023614
ptsG	-1.0081967	9.73880824	66.5532323	2.69E-05	0.0002366
yvcI	-1.0485456	6.15513717	66.0880134	2.76E-05	0.00024219
epsD	1.80356591	4.522365	66.0772436	2.76E-05	0.00024219
hemA	-0.8403147	9.35611457	65.9390778	2.78E-05	0.00024364
fliF	-1.1787921	9.95780203	65.8671322	2.79E-05	0.00024417
nagZ	1.30410536	6.06273143	65.7118705	2.82E-05	0.00024588
pksL	0.91127	10.0930286	65.5757025	2.84E-05	0.00024734
rpsD	-1.0363762	9.82960536	65.5172505	2.85E-05	0.00024769
yhxA	-0.9457745	7.27264073	65.4152757	2.87E-05	0.0002485
acuA	-1.0715905	5.86661186	65.3936468	2.87E-05	0.0002485
luxS	1.19578425	7.37212854	65.3480644	2.88E-05	0.00024867
pksH	1.4359136	6.24635929	65.2700815	2.89E-05	0.00024931
yplQ	-1.1078368	7.98481255	65.0295172	2.93E-05	0.00025233
pksG	1.44448975	6.65061342	64.9965986	2.94E-05	0.00025233
gntP	-1.1278931	5.9572047	64.9245104	2.95E-05	0.0002529
conQ	2.05701742	6.77530819	64.8764378	2.96E-05	0.00025312
cccB	-2.2387323	2.38014187	64.7229935	2.99E-05	0.00025492
metE	2.08985383	10.758711	64.6118709	3.01E-05	0.00025609
yaaC	2.05808811	4.14509755	64.5672667	3.01E-05	0.00025627
B4U62_RS224:	-1.2265909	6.22613835	64.5063982	3.02E-05	0.00025669
thyB	-0.7980712	7.19256605	64.2354095	3.07E-05	0.00026032
mtlF	-2.4260266	3.78304779	64.1731434	3.08E-05	0.00026078
sacC	-1.7399953	3.58354467	63.9460885	3.13E-05	0.00026371
ywaE	1.96102424	6.04509196	63.9198977	3.13E-05	0.00026371
hmpA	1.56761208	5.19520306	63.7984917	3.15E-05	0.00026511
opuBC	1.85058684	5.49686179	63.7131892	3.17E-05	0.00026595
yoqO	1.37901586	5.72677036	63.6747219	3.18E-05	0.00026605
ywhH	1.13528787	4.90570989	63.0660246	3.29E-05	0.00027493
ywdF	-1.0675744	5.74673236	63.0142467	3.30E-05	0.00027493
dltE	0.92810643	8.69171629	63.0036567	3.31E-05	0.00027493
dgcW	-1.1666126	6.19090291	62.9993118	3.31E-05	0.00027493
yxbF	1.16019111	4.79204235	62.8567024	3.34E-05	0.00027678
pyk	-0.8629293	10.5644795	62.7478435	3.36E-05	0.00027808
pepF	0.86531229	9.14173087	62.6627161	3.38E-05	0.00027841
sufU	0.82134096	8.77686446	62.6083967	3.39E-05	0.00027841
rplK	-0.8283613	11.3018359	62.5972005	3.39E-05	0.00027841
mraZ	-0.9673037	7.73099948	62.5770258	3.39E-05	0.00027841
bslA	1.1019532	7.19424332	62.5441999	3.40E-05	0.00027841

Table S5. Genes differentially expressed between ΔmnmE and WT in transcriptome analyses

yccK	1.00464861	6.13451909	62.542589	3.40E-05	0.00027841
fhuC	1.14258896	6.9989882	62.3505727	3.44E-05	0.00028114
caiJ	1.18362346	7.84482217	62.1063688	3.49E-05	0.00028482
B4U62_RS101	1.93511111	3.39558818	62.0026848	3.51E-05	0.00028593
cdaA	-1.1421021	8.00203059	61.9815722	3.52E-05	0.00028593
glgP	1.11183229	4.28755179	61.688967	3.58E-05	0.00029007
fliE	-1.4189375	7.07496777	61.686188	3.58E-05	0.00029007
ykvA	-1.0764886	7.3446551	61.5957463	3.60E-05	0.00029115
yfjF	1.41128303	5.5249117	61.5019346	3.62E-05	0.00029196
ytlR	-0.8805999	6.74668253	61.4909474	3.63E-05	0.00029196
tyrA	0.99610124	8.45485475	61.3789807	3.65E-05	0.00029297
yesJ	-1.1619665	5.00111273	61.3750772	3.65E-05	0.00029297
B4U62_RS093	1.10726448	6.65375243	61.3078613	3.67E-05	0.00029365
cymR	-1.3149356	6.1706338	61.1830317	3.69E-05	0.00029504
sdhA	-0.8584005	9.74135343	61.1727373	3.70E-05	0.00029504
ybaR	-1.0184746	6.83797817	61.0273386	3.73E-05	0.00029716
acnA	1.04121911	11.2108414	60.9182456	3.76E-05	0.00029863
rpsF	-1.4426351	8.79216226	60.7511532	3.79E-05	0.00030075
aprE	2.51909148	8.19884512	60.7305745	3.80E-05	0.00030075
yrzF	-0.9287109	6.40237029	60.713073	3.80E-05	0.00030075
nasF	0.93567627	9.65463003	60.6885061	3.81E-05	0.00030075
cdaR	-0.8913746	8.69152033	60.5287448	3.85E-05	0.00030268
rex	-1.3292106	8.5695936	60.5147833	3.85E-05	0.00030268
speB	-1.5210077	5.06670409	60.4722221	3.86E-05	0.00030268
argG	1.12803208	11.0327742	60.4543473	3.87E-05	0.00030268
glgD	1.20531732	4.17275669	60.4424467	3.87E-05	0.00030268
tsf	-1.1050812	9.22255952	60.3284063	3.90E-05	0.0003043
ecfA	-0.8741835	6.95344065	60.1981389	3.93E-05	0.00030625
mntR	-1.1397487	7.50255002	60.018896	3.97E-05	0.00030916
sufD	0.80044925	10.3793536	59.9771277	3.98E-05	0.00030943
xylR	0.81504653	6.32829768	59.9235433	4.00E-05	0.00030966
phyC	1.57100664	2.98114339	59.9090321	4.00E-05	0.00030966
B4U62_RS045	1.15198182	6.36795424	59.8572593	4.01E-05	0.00031013
mgtE	-0.958403	6.64468256	59.626589	4.07E-05	0.00031412
rplV	-1.1549551	9.7318296	59.5504105	4.09E-05	0.00031508
B4U62_RS227	0.993368	5.77087829	59.4695346	4.11E-05	0.00031533
ycdA	1.57035811	7.88896614	59.4617303	4.11E-05	0.00031533
rhaE	-1.45648	3.26614229	59.4549302	4.12E-05	0.00031533
yjzC	-3.0965713	1.38947978	59.0935804	4.21E-05	0.00032209
queG	-1.0718806	4.53026327	58.9675741	4.25E-05	0.00032387
pchC	1.85580055	11.5830571	58.9523766	4.25E-05	0.00032387
resB	-1.4383902	8.60160405	58.8178371	4.29E-05	0.00032583
yumC	0.90727995	9.02219486	58.803951	4.29E-05	0.00032583
ribBA	1.83121154	8.5542527	58.7048966	4.32E-05	0.00032734
levF	-1.5214893	4.00472707	58.5239854	4.37E-05	0.0003303

Table S5. Genes differentially expressed between ΔmnmE and WT in transcriptome analyses

maf	-0.9716968	6.27292416	58.4908853	4.38E-05	0.0003303
conG	2.20319206	5.69140801	58.4840183	4.38E-05	0.0003303
pbuX	1.67373408	6.6286096	58.4396952	4.39E-05	0.00033067
yqcG	0.88300274	8.26826206	58.3642801	4.41E-05	0.00033172
sspG	2.28171228	1.33644394	58.328082	4.42E-05	0.00033192
yfjQ	-0.9595172	5.82852207	58.2568014	4.44E-05	0.00033235
cysC	2.0490029	8.29993031	58.25509	4.44E-05	0.00033235
sirB	1.61192377	8.71017192	58.2062474	4.46E-05	0.00033284
yraJ	0.99592258	5.94491891	58.0831554	4.49E-05	0.00033438
B4U62_RS210	1.38533857	4.59480946	58.0625227	4.50E-05	0.00033438
tagG	-0.8959763	6.5225157	58.0561081	4.50E-05	0.00033438
epsC	1.38578412	5.04166267	58.0028633	4.52E-05	0.00033497
yhcJ	1.03160123	7.00329099	57.8983402	4.55E-05	0.00033668
splA	-1.3982621	3.0070565	57.7823962	4.58E-05	0.00033865
argJ	1.0833894	10.0361844	57.3721999	4.71E-05	0.00034723
pheT	0.77520688	8.29348934	57.3132091	4.72E-05	0.00034799
ltaS1	-0.8759332	8.4067612	57.2323625	4.75E-05	0.00034925
rplA	-0.8083631	11.1712605	57.1615272	4.77E-05	0.00035029
ptkA	-1.057372	7.02409293	57.0938983	4.79E-05	0.00035126
B4U62_RS028	2.35315705	3.21102777	57.0017897	4.82E-05	0.00035281
epr	-1.5278054	7.38349742	56.8835739	4.86E-05	0.00035446
fldN	3.0276821	6.03249896	56.8800737	4.86E-05	0.00035446
ywrJ	1.42452815	2.37706177	56.6845865	4.92E-05	0.00035847
yocA	-0.8008588	7.68754319	56.4872147	4.99E-05	0.00036258
lytD	-1.2889408	7.56907435	56.4590581	5.00E-05	0.00036266
rpsS	-0.9525457	9.78182918	56.4175076	5.01E-05	0.00036306
pdhC	-0.9166691	10.4358229	56.3450892	5.04E-05	0.00036421
hmoA	1.08845695	6.13803832	56.2322381	5.07E-05	0.00036635
ydjP	1.16428339	7.65626437	56.0805677	5.12E-05	0.00036946
dnaB	-0.9588665	6.4620272	56.0054778	5.15E-05	0.00037071
trpD	1.36510301	8.93373572	55.97976	5.16E-05	0.00037074
rplC	-0.8183632	11.0214202	55.8543994	5.20E-05	0.00037325
rpsR	-1.2797889	7.58490442	55.7995496	5.22E-05	0.00037373
addB	-0.7962359	8.50908698	55.7866364	5.23E-05	0.00037373
yknW	1.12161523	9.29938997	55.6937938	5.26E-05	0.00037545
ytkA	-1.977814	6.07150943	55.5554893	5.31E-05	0.00037834
yybM	1.2007563	7.83431219	55.408771	5.36E-05	0.00038148
yeaA	1.06776021	7.42159755	55.360091	5.38E-05	0.00038211
med	-0.7387734	7.10694566	55.2248039	5.43E-05	0.000385
rpmF	-1.0435841	7.0236947	55.1761772	5.45E-05	0.00038564
B4U62_RS117	1.20419075	4.6405981	55.0187841	5.50E-05	0.00038914
rlmH	-0.9360635	5.40841304	54.9758011	5.52E-05	0.00038914
B4U62_RS185	-1.4433394	7.95396831	54.9718378	5.52E-05	0.00038914
ktrD	-1.1504692	5.53829287	54.745168	5.61E-05	0.00039455
wapI	1.37047052	7.80637318	54.4829478	5.71E-05	0.00040103

Table S5. Genes differentially expressed between ΔmnmE and WT in transcriptome analyses

pyrH	-1.0703068	7.81906708	54.3337106	5.77E-05	0.0004045
mutT	-1.8732356	2.06772512	54.1403588	5.84E-05	0.00040877
ltaS	-0.9179602	8.7299874	54.1345161	5.85E-05	0.00040877
motP	-1.4646061	3.36478446	54.0703907	5.87E-05	0.00040993
amiE	1.26858566	5.64124017	53.9824727	5.91E-05	0.00041176
yisI	1.56110615	3.67375784	53.9508838	5.92E-05	0.00041201
rplB	-0.7170176	11.5017818	53.9264331	5.93E-05	0.00041205
fhuG	0.98474725	6.70313809	53.8356014	5.97E-05	0.00041399
B4U62_RS027	2.02464994	4.51417791	53.7783087	5.99E-05	0.00041498
pxpB	2.18827236	5.23807507	53.7515968	6.00E-05	0.0004151
radC	-0.9549719	6.47192067	53.7106549	6.02E-05	0.00041562
ccdA	1.1769156	5.39067323	53.6002027	6.07E-05	0.00041798
B4U62_RS104	1.23569136	4.16814558	53.5839434	6.07E-05	0.00041798
xseA	-1.0439394	6.41165098	53.4539386	6.13E-05	0.00042111
rsmG	-0.7715549	7.5793823	53.4190381	6.14E-05	0.00042138
rbsR	-1.343457	4.74255394	53.3999836	6.15E-05	0.00042138
metQ	1.20897835	7.7444257	53.1222415	6.27E-05	0.00042877
acpK	1.44189462	5.47189043	53.1062272	6.28E-05	0.00042877
B4U62_RS027	2.01451446	5.34023191	53.0652464	6.30E-05	0.00042933
helP	1.84171361	5.40832112	52.9955779	6.33E-05	0.00043077
speA	-0.9392129	5.33261657	52.8815957	6.38E-05	0.00043346
B4U62_RS025	-1.4764049	8.70514718	52.862674	6.39E-05	0.00043346
ypzA	1.01102673	5.35221579	52.6639103	6.48E-05	0.00043888
yfmQ	-2.2059078	7.78372451	52.6069078	6.50E-05	0.00043997
mtlD	-2.1034847	5.09941025	52.5119421	6.55E-05	0.00044225
hslU	-0.9260794	9.09019865	52.4095373	6.59E-05	0.00044478
yqbG	-2.3221745	1.97664119	52.3704531	6.61E-05	0.00044526
dinG	-0.7550934	8.50453137	52.3511921	6.62E-05	0.00044526
tlpB	-1.107763	5.08524029	52.3115893	6.64E-05	0.00044583
ywpJ	0.76790429	6.32794617	52.2799247	6.65E-05	0.00044615
yodF	1.27079658	8.29960688	52.2471455	6.67E-05	0.00044651
cdd	-1.0086981	8.61647622	52.0406227	6.77E-05	0.00045242
dtd	0.74354775	7.22298783	51.9653615	6.80E-05	0.00045417
rpsC	-0.7131352	10.7909906	51.8588423	6.86E-05	0.00045694
sboA	-1.7381113	10.9472743	51.8135367	6.88E-05	0.00045743
grpE	-0.8093975	8.1731077	51.8013537	6.88E-05	0.00045743
phoA	0.96787709	5.454632	51.7135582	6.93E-05	0.00045962
xkdA	1.34259201	5.52600677	51.6529537	6.96E-05	0.00046093
spo0M	0.96190239	8.63423779	51.5410124	7.01E-05	0.00046394
ywrD	2.51438186	1.94329488	51.4981743	7.04E-05	0.00046467
glgB	1.47882887	4.02905595	51.3935584	7.09E-05	0.00046748
pfeT	1.22894052	6.14601278	51.3370277	7.12E-05	0.00046869
ytcJ	0.96732579	7.88286171	51.3136714	7.13E-05	0.00046877
ycsN	0.76630903	9.23003327	51.2451429	7.17E-05	0.00047039
yqiG	-0.794889	6.25618194	51.2047133	7.19E-05	0.00047107

Table S5. Genes differentially expressed between ΔmnmE and WT in transcriptome analyses

B4U62_RS034	1.13846534	3.80581871	51.1167216	7.23E-05	0.00047273
rplW	-0.7574264	9.79806584	51.1147869	7.23E-05	0.00047273
levG	-1.4355087	4.23931662	50.8296483	7.38E-05	0.00048189
rpiB	-0.7768636	7.4466503	50.7911992	7.40E-05	0.00048252
dgk	-1.0530421	5.99145711	50.7259428	7.44E-05	0.00048351
yfmB	1.16554562	6.69860973	50.722483	7.44E-05	0.00048351
pbuO	-1.3279622	4.45019671	50.6228304	7.50E-05	0.00048631
feuV	0.83603155	9.15596797	50.4381605	7.60E-05	0.00049219
skiX	0.92790664	8.58582918	50.1983007	7.73E-05	0.00050019
flgM	-1.0387154	7.30823255	50.1599371	7.75E-05	0.00050051
ydeQ	1.14394754	4.82779475	50.1494755	7.76E-05	0.00050051
gabR	1.17886387	4.2953765	50.0154546	7.84E-05	0.00050388
exlX	1.08841743	4.74844758	50.0054535	7.84E-05	0.00050388
ywaD	1.71986425	5.30050698	49.9988465	7.85E-05	0.00050388
bsrA	-1.1287244	8.91516021	49.9244894	7.89E-05	0.00050591
fliT	-1.5011715	6.81972217	49.8923993	7.91E-05	0.00050638
pta	-1.0632498	7.68394072	49.720005	8.01E-05	0.00051213
deoD	-0.952525	5.66197778	49.6956744	8.02E-05	0.00051231
spoVG	1.23820781	9.28534388	49.6186936	8.07E-05	0.0005145
ybfA	-1.212222	4.54082265	49.4667038	8.16E-05	0.00051959
ylqF	-0.9825816	5.68898427	49.4037519	8.20E-05	0.000521
hisC	1.21854685	10.1841135	49.3864461	8.21E-05	0.000521
yugI	-1.0155164	7.0919658	49.372158	8.22E-05	0.000521
flgN	-0.8405063	7.94578169	49.1954766	8.33E-05	0.00052668
citZ	1.10960026	11.0840775	49.1883529	8.33E-05	0.00052668
yvrC	-0.947126	6.83154743	48.974048	8.47E-05	0.00053443
rpsQ	-0.7705311	9.8632818	48.8109077	8.57E-05	0.00054025
cwlS	0.85491696	8.3366892	48.7042601	8.64E-05	0.00054246
yddM	1.2184167	5.09691671	48.7017396	8.64E-05	0.00054246
yhfW	1.60719632	3.19727893	48.6999317	8.64E-05	0.00054246
pxpR	1.6448807	4.43011535	48.6599817	8.67E-05	0.00054332
flgC	-1.2644773	8.23707946	48.467152	8.80E-05	0.00055052
slrA	0.89909217	6.49157293	48.3393835	8.88E-05	0.00055509
sodC	1.12585567	4.53460053	48.2534371	8.94E-05	0.00055793
B4U62_RS231	-2.0042486	1.93844922	48.2041702	8.98E-05	0.00055923
B4U62_RS034	1.44112895	2.41115831	48.1556781	9.01E-05	0.00055976
ycnB	1.06328065	7.0567644	48.1547825	9.01E-05	0.00055976
lytB	-1.1169587	8.61253685	48.0688426	9.07E-05	0.00056179
ywnH	-0.9504524	5.63483106	48.0596262	9.08E-05	0.00056179
fliG	-1.3269313	8.58526214	48.0517076	9.08E-05	0.00056179
B4U62_RS225	-1.8181346	1.55495222	47.9179883	9.17E-05	0.00056677
B4U62_RS066	1.21929775	4.06502967	47.8888417	9.19E-05	0.00056724
ytiB	0.81509485	7.25223529	47.8609376	9.21E-05	0.00056765
lrpC	-1.1324813	4.53213798	47.7775042	9.27E-05	0.00057049
clsA	-0.8267104	6.6283172	47.6099983	9.39E-05	0.00057707

Table S5. Genes differentially expressed between ΔmnmE and WT in transcriptome analyses

liaI	1.19435445	3.19711406	47.5473754	9.44E-05	0.00057905
mraY	-0.7520081	7.25766052	47.4637302	9.50E-05	0.00058199
B4U62_RS105	1.41525421	2.28789073	47.3948304	9.55E-05	0.00058428
yczN	1.71594779	3.02248486	47.2904163	9.63E-05	0.00058819
moaC	-0.832147	6.04525042	47.1315068	9.75E-05	0.00059424
ywqN	1.56380105	3.78191046	47.1225781	9.75E-05	0.00059424
ftsE	-0.9991128	7.3676943	47.0970457	9.77E-05	0.0005946
fabF	-0.9766037	8.78887191	47.0442754	9.81E-05	0.00059622
B4U62_RS098	1.13904665	10.2784524	47.0080466	9.84E-05	0.00059707
sboX	-1.6007563	10.7707432	46.951701	9.88E-05	0.00059887
yqeC	1.49530057	2.46775402	46.9329563	9.90E-05	0.00059892
pstA	-1.7997571	2.16541329	46.9056685	9.92E-05	0.00059937
levE	-1.8304184	3.55779012	46.7127038	0.0001007	0.00060715
fapR	-1.2219458	7.55075741	46.6957188	0.00010083	0.00060715
rsiX	0.81207488	8.2257593	46.6878303	0.00010089	0.00060715
ycnK	-1.4643544	10.3348589	46.6297787	0.00010135	0.00060909
cgeE	-1.1472193	5.6891433	46.5145818	0.00010227	0.00061377
ywdK	-1.4103389	7.08680644	46.4606041	0.00010271	0.00061492
yoyG	-1.1400373	3.18177402	46.4562335	0.00010274	0.00061492
dnaA	-0.8949074	8.31474051	46.2694713	0.00010426	0.00062317
bioA	0.92180812	9.9153021	46.1612223	0.00010516	0.00062766
B4U62_RS204	-1.5430795	4.25705475	46.0726983	0.0001059	0.00063122
yvbX	-0.777446	6.91794802	45.9852562	0.00010663	0.00063428
rapA	1.53496826	11.7274145	45.9773354	0.0001067	0.00063428
ykcB	-1.4951206	3.0703195	45.7954816	0.00010825	0.00064264
ykcC	-1.8401309	1.90646941	45.6829445	0.00010922	0.00064755
ycbP	0.79140387	7.88262342	45.5414413	0.00011046	0.00065325
bioF	0.90082244	9.92934734	45.531014	0.00011056	0.00065325
gmk	-0.7292633	8.68020639	45.5227314	0.00011063	0.00065325
ypuF	-1.4478771	1.96846932	45.4247729	0.0001115	0.00065699
rpsI	-0.7352423	10.2670334	45.417185	0.00011157	0.00065699
dltD	0.77460926	9.33359993	45.4011192	0.00011171	0.00065699
ribH	1.58658699	7.69157421	45.3358548	0.0001123	0.00065956
yonS	-1.335381	5.48881278	45.3158436	0.00011248	0.00065974
yjcK	0.97706352	5.24739293	45.2580136	0.000113	0.00066193
era	-0.8365832	9.68717237	45.2217256	0.00011333	0.0006624
ytwI	-0.9694009	4.15548229	45.2160282	0.00011338	0.0006624
niaP	-0.8578561	8.04627917	45.110654	0.00011435	0.00066716
oxdC	3.04360836	6.8295927	49.328455	0.00011502	0.00067017
rpsL	-0.6559568	10.8663715	44.9692523	0.00011566	0.00067303
nprE	1.61640605	7.01121895	44.8875657	0.00011643	0.00067659
glgA	1.04746491	4.17727368	44.8531504	0.00011675	0.00067759
ypjC	-0.8691097	6.3840509	44.6844526	0.00011836	0.00068602
fliI	-1.0223104	9.48439438	44.6318625	0.00011887	0.00068805
ywoF	-1.1072459	7.40059859	44.5467323	0.00011969	0.00069192

Table S5. Genes differentially expressed between ΔmnmE and WT in transcriptome analyses

yqhL	-1.5005911	5.78939691	44.4772489	0.00012037	0.00069494
yxkC	-1.3714086	7.73484041	44.3873781	0.00012126	0.0006983
yqjN	1.03965494	7.45003874	44.3860593	0.00012127	0.0006983
yhaO	-0.7630405	6.15141605	44.3411709	0.00012172	0.00069995
ydbT	0.84251445	8.90747732	44.3061124	0.00012207	0.00070025
ythP	1.07816589	7.13693419	44.3040731	0.00012209	0.00070025
tagV	-0.8425868	7.03150407	44.2220304	0.00012291	0.00070265
yfjA	1.18407431	3.38953456	44.2190292	0.00012294	0.00070265
yopD	0.72710925	6.47476698	44.2147027	0.00012298	0.00070265
yycB	0.85839817	9.84750377	44.1292006	0.00012385	0.00070668
ywjB	0.96204238	7.07468673	43.9526326	0.00012566	0.00071608
fdhA	0.99236849	7.3278836	43.8342923	0.00012689	0.00072217
minJ	-0.7442645	7.99927288	43.7310024	0.00012798	0.00072742
yfzA	1.414265	4.87260678	43.618782	0.00012918	0.00073327
bslB	1.01006704	9.53230827	43.5734353	0.00012966	0.00073509
B4U62_RS045	1.13392803	5.74421846	43.5438822	0.00012998	0.00073594
galM	0.67488778	7.13436682	43.4361193	0.00013115	0.00074142
rplN	-0.7414753	9.8592858	43.4221539	0.0001313	0.00074142
ywtG	1.19968785	9.54752564	43.4084677	0.00013145	0.00074142
flgB	-1.3214876	8.1426991	43.3914858	0.00013164	0.00074152
yhcB	-0.8929273	5.5004932	43.3328798	0.00013228	0.00074398
yyaJ	1.23435397	5.04205089	43.3210223	0.00013241	0.00074398
dapB	-1.0794471	5.88578117	43.1911866	0.00013386	0.00075059
ypdA	-0.7822153	7.42055915	43.1845667	0.00013393	0.00075059
ytxK	-0.7908875	6.14055658	43.1600909	0.00013421	0.00075118
yxiM	1.22798861	5.62025434	42.9681708	0.00013638	0.00076239
nfeDA	1.26997859	10.7246512	42.8463289	0.00013779	0.00076926
sdpC	0.95166429	11.7838647	42.7895882	0.00013845	0.00077197
atpI	-1.3303085	8.81868651	42.6532482	0.00014005	0.00077992
yxeN	2.0481938	4.89116375	42.6191806	0.00014045	0.00078118
yqgC	0.80049556	7.86888422	42.4565011	0.0001424	0.00079102
B4U62_RS230	4.11925277	1.5429217	42.353731	0.00014365	0.0007968
nudF	-0.9028534	5.92353752	42.3411226	0.0001438	0.0007968
yisQ	-1.700003	2.59327867	42.3166511	0.0001441	0.00079746
lnrJ	-0.9475978	5.4221769	42.2290893	0.00014518	0.00080242
efpI	-0.9952445	3.95231188	42.1909286	0.00014566	0.00080381
yfjD	1.01143013	6.09047938	42.179347	0.0001458	0.00080381
yjbA	0.98282789	4.70986268	42.1246707	0.00014648	0.00080656
licR	0.74003709	7.06760629	41.9900884	0.00014818	0.00081488
cotB	0.94657806	3.69924053	41.8327926	0.00015019	0.00082492
sdhB	-0.700087	9.22436132	41.785109	0.00015081	0.00082728
yqgM	1.06759133	4.53716838	41.7545089	0.00015121	0.00082843
tsaC	-1.0297096	5.48486543	41.6102691	0.0001531	0.00083775
ggt	1.43128729	6.30932909	41.4940257	0.00015464	0.00084516
yxiD	1.41864042	6.55786912	41.4456103	0.00015529	0.00084765

Table S5. Genes differentially expressed between ΔmnmE and WT in transcriptome analyses

bacG	0.72999965	8.33131822	41.4035634	0.00015586	0.00084969
spoIID	-1.0949348	4.80599074	41.3760526	0.00015623	0.00085067
serA	1.03289966	10.1341637	41.2992129	0.00015728	0.0008553
purL	1.37307425	9.86359593	41.2804701	0.00015753	0.00085564
ftsW	0.72276597	7.89382225	41.2202069	0.00015836	0.00085908
phrH	0.70481766	6.5481345	41.2012109	0.00015862	0.00085944
yabP	-0.8043028	6.66969444	41.1063376	0.00015994	0.00086551
cysI	1.28316864	9.144585	41.0609715	0.00016057	0.00086788
rsbU	0.77372658	7.90019519	41.0151868	0.00016122	0.00086965
sigI	-0.7710913	6.04942691	40.9977015	0.00016146	0.00086965
yfhC	1.12993685	7.33821042	40.9954887	0.00016149	0.00086965
yabM	-0.8374948	5.23602858	40.9259804	0.00016248	0.00087387
ureC	1.99491335	8.5563113	40.9123264	0.00016267	0.00087387
dnaC	-0.914293	6.89426147	40.8936668	0.00016294	0.00087423
pbpB	-0.8000222	7.09907842	40.8557974	0.00016348	0.00087607
cmk	-1.3423398	6.56414911	40.8106218	0.00016413	0.00087848
aroA	0.90682412	8.45805711	40.7525514	0.00016497	0.00088189
lutR	-0.718647	6.25618791	40.5243051	0.00016832	0.00089871
ywiC	-1.0720556	4.14363836	40.4223502	0.00016984	0.00090574
yolD	-1.4239412	2.27742842	40.3205147	0.00017138	0.00091284
rnpB	-1.2877063	10.7902041	40.2530624	0.00017241	0.00091721
B4U62_RS139	1.71306624	8.72235504	40.237391	0.00017265	0.00091738
pbuE	-1.4906362	3.58937639	40.1279475	0.00017433	0.00092523
fhuD	1.11803537	8.79795734	40.0850381	0.000175	0.00092766
B4U62_RS227	1.34326914	5.27695196	40.0511526	0.00017553	0.00092934
ptpZ	-1.075466	4.67896302	39.9704006	0.0001768	0.00093494
chrB	1.22255204	4.29327545	39.9168923	0.00017765	0.00093763
pdhA	-1.013565	8.79356064	39.9114146	0.00017773	0.00093763
ywiB	-0.9874551	5.94682623	39.8973953	0.00017796	0.00093768
yvrN	0.82212121	8.05557592	39.8344204	0.00017896	0.00094185
spoIISB	-0.9431408	5.01866246	39.7878805	0.00017971	0.00094466
ycnJ	-1.1803368	11.4178521	39.7574733	0.0001802	0.00094611
sndC	1.13187982	10.1596995	39.6900162	0.0001813	0.00095072
ywaC	-1.2145515	5.17575111	39.6613344	0.00018176	0.00095184
xynB	1.23389435	4.7354906	39.6414776	0.00018209	0.00095184
nin	-0.9556307	4.43642621	39.6268528	0.00018233	0.00095184
floA	1.25563929	9.88374617	39.6241509	0.00018237	0.00095184
yqaS	-1.4664295	2.74528492	39.5577281	0.00018347	0.00095641
yubF	-1.3958821	4.88699558	39.461702	0.00018506	0.00096359
prli42	-1.1222515	3.76975065	39.3196079	0.00018745	0.00097489
yxeK	1.77839859	4.51026314	39.2568511	0.00018852	0.0009793
pstBB	-1.7372196	2.16713812	39.2407306	0.0001888	0.00097957
purF	1.4474753	9.95647549	39.2238243	0.00018909	0.00097992
bfmBAA	-0.7459808	6.23684914	39.1445729	0.00019045	0.00098584
ogt	0.93230456	7.84757835	39.1214674	0.00019085	0.00098675

Table S5. Genes differentially expressed between ΔmnmE and WT in transcriptome analyses

dat	0.68537579	9.49669695	39.0170011	0.00019268	0.00099501
pbpF	-0.8211484	6.09284503	38.9835507	0.00019327	0.00099688
racE	-0.9648156	7.57226422	38.9543633	0.00019378	0.00099837
cysS	-0.6654746	7.73048297	38.8823836	0.00019506	0.00100378
yoxD	-0.8965291	5.03322745	38.8578029	0.0001955	0.00100487
gabP	0.82230426	8.78723331	38.777415	0.00019694	0.00101111
ribD	1.74074824	8.38992486	38.7522673	0.0001974	0.00101226
walK	-0.667026	7.82849957	38.6018765	0.00020014	0.00102458
ywlG	-0.6928409	8.1617905	38.5951728	0.00020026	0.00102458
ywzB	-1.1824856	7.11356199	38.5773781	0.00020059	0.00102507
pxpC	1.86062417	5.21205221	38.5076094	0.00020189	0.00103048
B4U62_RS123	0.84931183	8.41410158	38.4842027	0.00020232	0.00103151
fabH	-0.9288542	7.77516326	38.458267	0.00020281	0.00103278
B4U62_RS176	2.27109927	0.72454987	38.4410082	0.00020313	0.00103323
ptsH	-0.925171	8.7738988	38.4065463	0.00020378	0.00103362
lytE	0.71350864	8.79514884	38.3999499	0.0002039	0.00103362
ykoJ	1.06577775	4.17956564	38.3995131	0.00020391	0.00103362
ytbD	-1.3715655	1.76642459	38.3210166	0.00020539	0.00103935
yyzF	-0.9570329	3.78173108	38.2858062	0.00020606	0.00103935
ykoX	-0.9163442	4.90490829	38.2799452	0.00020618	0.00103935
amhX	0.75682438	9.13610096	38.2788445	0.0002062	0.00103935
acuC	-0.8239478	6.95982601	38.2776294	0.00020622	0.00103935
rlmCD	-0.9511466	5.81119754	38.1062628	0.00020952	0.00105368
dck	-0.7576843	6.10848334	38.1054684	0.00020954	0.00105368
yqxK	-0.6535651	6.99624174	37.9647288	0.0002123	0.00106636
nupN	0.75962628	9.18422636	37.8906101	0.00021378	0.00107254
ftsL	-0.7207812	7.07666405	37.8729792	0.00021413	0.00107263
cysE	-1.0032251	5.8015992	37.8652654	0.00021428	0.00107263
mutY	-0.8279924	5.69831859	37.7226493	0.00021716	0.00108581
chrA	1.2264119	3.92541431	37.6977338	0.00021767	0.00108694
ywzC	-1.3322266	3.44090979	37.6874241	0.00021788	0.00108694
yphE	0.87892105	6.97345602	37.5692366	0.00022031	0.00109783
bshB1	-1.0040908	5.66374751	37.5002831	0.00022175	0.00110373
ntdR	0.76442806	7.89614533	37.429541	0.00022323	0.00110982
mecA	0.75368701	9.92158406	37.4180324	0.00022348	0.00110982
rplX	-0.8245933	9.4896007	37.346032	0.000225	0.00111613
hemZ	-1.083764	4.89841567	37.2492188	0.00022707	0.00112514
rapZ	-0.6372313	8.06342539	37.2161737	0.00022778	0.00112627
yusT	0.89904855	4.93952842	37.2080235	0.00022796	0.00112627
yutE	-0.7180828	5.99420161	37.2031034	0.00022807	0.00112627
rpsB	-0.6761942	10.0137889	37.1729762	0.00022872	0.00112822
B4U62_RS063	0.82730353	10.7027756	37.1310246	0.00022963	0.00113146
ycgK	0.985758	4.04300218	37.0401932	0.00023162	0.00113882
cysJ	1.1088898	8.42548699	37.039292	0.00023164	0.00113882
gamP	-0.9663265	5.6812546	36.9747139	0.00023307	0.00114457

Table S5. Genes differentially expressed between ΔmnmE and WT in transcriptome analyses

ydhC	-1.0190993	4.50358819	36.9387785	0.00023387	0.00114722
bmrD	0.64176581	7.02508742	36.8699692	0.00023541	0.0011535
ydfF	0.79268019	5.69779837	36.8171173	0.00023661	0.00115806
lpdV	-0.8281445	6.28969001	36.7551761	0.00023801	0.00116365
yabE	-0.917573	8.47563244	36.7122013	0.000239	0.00116625
argH	0.88101192	11.2765826	36.7087378	0.00023908	0.00116625
yvrO	0.74699554	7.13519572	36.5399091	0.00024299	0.00118401
fliH	-1.167215	8.35885245	36.5151216	0.00024357	0.00118553
B4U62_RS161	-0.7803145	7.34160634	36.4642259	0.00024476	0.00118992
degU	0.93146656	8.30954716	36.4539235	0.00024501	0.00118992
ydeD	1.45172511	3.2626673	36.436308	0.00024542	0.00119063
ygaD	-0.8981405	6.64455625	36.3711663	0.00024697	0.00119682
lcfA	-0.8184845	8.15834121	36.3403094	0.00024771	0.00119884
dnaX	-0.6351271	7.85843704	36.3309707	0.00024793	0.00119884
tcdA	-0.6968208	6.72180818	36.2956787	0.00024878	0.00120163
ribE	1.90420411	6.94937821	36.2840498	0.00024906	0.00120166
ydcK	-0.8096233	6.0194365	36.2723171	0.00024935	0.00120171
yvbH	0.90066904	6.51701616	36.1877491	0.0002514	0.00121028
ydbM	-1.2718282	7.84322312	36.1608484	0.00025206	0.00121095
B4U62_RS189	1.27750138	4.67507242	36.159565	0.00025209	0.00121095
sndB	1.77676619	5.35583199	36.1468166	0.0002524	0.00121113
B4U62_RS197	1.66975512	3.74151284	36.1093739	0.00025332	0.00121326
yfmK	0.97400857	4.62331414	36.0973653	0.00025362	0.00121326
snaB	2.0145927	3.45278322	36.084794	0.00025393	0.00121326
ylmC	0.97151744	5.19391527	36.0840131	0.00025394	0.00121326
galE	-0.7373759	7.61378065	36.0223604	0.00025547	0.00121924
ykpC	-1.6842221	2.11522197	35.9891526	0.0002563	0.00122187
B4U62_RS116	-1.1143984	5.12068896	35.9426372	0.00025747	0.0012261
menB	-0.6450711	8.47404428	35.8910747	0.00025877	0.00122978
yoyD	1.16271269	5.82444619	35.8869679	0.00025887	0.00122978
pnbA	0.61526901	7.50084763	35.8787777	0.00025908	0.00122978
yopT	-0.9275495	4.69988663	35.8493436	0.00025982	0.001232
nirD	0.83822136	8.47617984	35.8241601	0.00026047	0.00123287
rplM	-1.1566812	10.1714467	35.8200961	0.00026057	0.00123287
yceH	0.85924048	8.20269647	35.7511703	0.00026233	0.00123989
lmrA	0.71541541	5.83983166	35.6982155	0.0002637	0.00124501
yybJ	1.53205008	6.56125942	35.6822266	0.00026411	0.00124564
yonR	-1.6312922	3.92235666	35.631567	0.00026543	0.00125051
phrA	1.54304103	8.12857428	35.5183789	0.00026841	0.00126317
rlmB	-0.8183937	6.7241958	35.4980624	0.00026894	0.00126435
B4U62_RS045	1.0298751	5.75212172	35.4696685	0.0002697	0.00126655
ydaF	1.05346159	5.32442103	35.4492667	0.00027024	0.00126682
pdhB	-1.0547546	8.54526535	35.445913	0.00027033	0.00126682
yflR	-0.7911494	5.75585228	35.3436803	0.00027308	0.00127832
B4U62_RS144	0.92744666	3.90781454	35.3135975	0.00027389	0.00128078

Table S5. Genes differentially expressed between ΔmnmE and WT in transcriptome analyses

rpmC	-0.6756724	9.56859762	35.2538808	0.00027552	0.00128701
B4U62_RS177!	0.66782761	8.40080157	35.2141692	0.0002766	0.00129002
gntZ	-0.753309	6.70692201	35.2090293	0.00027675	0.00129002
rsmH	-0.6282643	8.72578221	35.1876477	0.00027733	0.00129139
ydjJ	1.44859296	7.09036968	35.0501779	0.00028115	0.0013074
speE	-1.1285036	5.62623788	35.0388363	0.00028147	0.0013074
pyrD	0.68286219	7.9249359	35.0319916	0.00028166	0.0013074
fumC	0.63265591	9.47605837	35.0124998	0.00028221	0.00130857
dacC	0.63614879	7.30594515	34.9934002	0.00028275	0.00130968
sigO	2.2428587	2.4174155	34.9597761	0.0002837	0.00131271
ytrD	0.85881115	5.19413585	34.9096968	0.00028512	0.0013178
rsiO	2.89033276	2.01169522	34.9000575	0.0002854	0.0013178
yqiS	-0.7156753	6.5458918	34.8277317	0.00028747	0.00132598
B4U62_RS115!	-1.0402828	5.6256335	34.8111023	0.00028795	0.0013268
trpF	1.41445543	8.8902806	34.7771276	0.00028893	0.00132923
pps	0.83181052	5.27644352	34.7720462	0.00028908	0.00132923
nucB	1.6434211	2.16826748	34.6890759	0.0002915	0.00133895
ylqH	-1.2404655	4.05011531	34.6782102	0.00029182	0.00133901
appF	0.75247558	9.42180693	34.585553	0.00029455	0.00135015
comZ	-0.7846526	5.68002603	34.5566845	0.00029541	0.00135268
ywjA	1.05168676	9.793625	34.5261997	0.00029632	0.00135544
yxaB	0.7446022	6.55438015	34.4451272	0.00029876	0.00136516
yckB	-1.1431884	4.60537027	34.4228477	0.00029943	0.00136682
hypO	-0.7355136	5.31245515	34.3750946	0.00030088	0.00137202
yetK	1.02720325	6.90876317	34.2690297	0.00030413	0.00138542
pcrB	-1.003415	4.92395426	34.2262864	0.00030546	0.00138884
ydeK	-0.9035678	4.38484112	34.2203895	0.00030564	0.00138884
sigG	1.2296433	2.6561441	34.2142639	0.00030583	0.00138884
znuC	-0.7589062	6.54389	34.1839678	0.00030677	0.00139169
yfkL	0.81873847	5.80941334	34.1385425	0.00030819	0.0013967
mrnC	-0.7184397	5.90936092	34.114491	0.00030895	0.00139869
ilvD	0.72135497	9.47494658	34.073431	0.00031025	0.00140311
dhaS	0.70121859	11.0035483	34.0571119	0.00031076	0.00140401
mnmA	-0.8662775	8.02582391	34.0374257	0.00031139	0.00140539
nupP	0.89851677	7.86428446	33.9417981	0.00031445	0.00141774
yxeO	1.96363552	4.46595019	33.8653325	0.00031692	0.00142743
bglH	-1.8644218	6.55235503	33.8458717	0.00031755	0.00142882
B4U62_RS105	1.59033663	2.56849293	33.7917835	0.00031932	0.00143435
B4U62_RS095	1.8982449	3.23260826	33.7883106	0.00031943	0.00143435
rmgR	0.91448608	4.35903218	33.7699879	0.00032003	0.00143559
B4U62_RS101!	-1.2307652	3.88318021	33.6035369	0.00032557	0.00145892
yfiU	1.4768045	2.65142992	33.577545	0.00032644	0.00146135
aroX	0.94016162	9.21931621	33.5261748	0.00032818	0.00146764
levD	-1.6968875	2.81499896	33.5012007	0.00032903	0.00146887
trmB	-1.1045277	5.98084087	33.4900578	0.00032941	0.00146887

Table S5. Genes differentially expressed between ΔmnmE and WT in transcriptome analyses

appD	0.79544042	9.33434518	33.4886251	0.00032945	0.00146887
spxO	-0.690468	6.41864861	33.46943	0.00033011	0.0014703
ywnJ	1.41594459	2.31221927	33.4555479	0.00033058	0.00147093
yceG	0.8228317	9.06189538	33.2251503	0.00033859	0.00150383
trpB	1.23852322	8.87090568	33.2231134	0.00033866	0.00150383
yxjL	0.92904205	5.03533825	33.1771757	0.00034028	0.00150952
racX	0.77502859	7.8479755	33.1590134	0.00034093	0.00151086
lipA	-0.5981541	8.39257899	33.1272537	0.00034206	0.00151435
polYB	-1.5848262	2.17733294	33.0972714	0.00034313	0.00151757
xseB	-1.1955277	3.35774467	33.0852296	0.00034357	0.00151796
rsbQ	-0.6211376	6.58713101	32.9945951	0.00034684	0.00153088
hslR	-1.0593775	6.203701	32.9817474	0.00034731	0.00153141
nucA	-1.1088059	4.34211755	32.9509779	0.00034843	0.00153482
ycbU	0.70747829	5.4030576	32.8994274	0.00035032	0.0015416
ispA	-0.7404644	5.8678057	32.7830121	0.00035463	0.00155903
ftsZ	0.67755951	10.415074	32.6954058	0.00035792	0.00157192
mrfA	-0.7010723	7.20760687	32.6642305	0.0003591	0.00157553
tagH	-0.6196285	7.13520538	32.5617691	0.00036301	0.0015911
glcR	0.67100914	6.07658352	32.5006121	0.00036537	0.00159985
lipC	1.38785993	3.79578743	32.4773434	0.00036627	0.00160221
aroQ	-0.6010347	7.55037794	32.4133034	0.00036877	0.00161154
ykuC	-0.6329976	6.5333607	32.38287	0.00036997	0.00161388
ndk	-1.1535037	6.74590203	32.3810455	0.00037004	0.00161388
ywmB	-0.9289996	8.31862783	32.3644586	0.00037069	0.00161513
bioY	1.01927617	6.51373013	32.3529338	0.00037115	0.00161528
rpsN	-0.5916233	9.83866283	32.3392995	0.00037169	0.00161528
pbpD	-0.6471096	8.89574639	32.3357275	0.00037183	0.00161528
rplP	-0.6081551	10.4646418	32.2788087	0.00037409	0.0016235
yxlF	-0.7075519	5.60092602	32.1710036	0.00037842	0.00164068
htpX	-0.7473536	7.06792078	32.1054499	0.00038108	0.0016506
yugF	1.45831765	3.32927046	32.0900902	0.00038171	0.0016517
ywqG	0.79490426	5.74423919	32.0378869	0.00038385	0.00165934
B4U62_RS226	0.60099105	7.67931747	31.9715727	0.00038659	0.00166955
pbpE	0.81101135	8.26954607	31.8810122	0.00039038	0.00168407
rpe	0.60416813	7.00544184	31.8728751	0.00039072	0.00168407
corA	0.72164671	7.84444844	31.8610886	0.00039122	0.00168456
bglP	-1.90985	7.63184026	31.7674166	0.00039519	0.00169951
xerD	-0.6214976	7.05867095	31.7610844	0.00039546	0.00169951
nupQ	1.02978543	7.70549658	31.7484916	0.000396	0.00170017
purQ	1.1650958	8.12549384	31.6324577	0.000401	0.0017183
minD	0.60958794	8.54322792	31.4922206	0.00040716	0.00174151
yjdF	-0.9959478	4.19667727	31.4909993	0.00040721	0.00174151
ypiF	0.8145694	4.49198409	31.4502772	0.00040902	0.00174755
ywdJ	-0.6366979	6.3147383	31.4036085	0.0004111	0.00175476
yybK	1.47061275	6.00475545	31.3792537	0.0004122	0.00175772

Table S5. Genes differentially expressed between ΔmnmE and WT in transcriptome analyses

cmpA	2.08384498	0.6511123	31.3491823	0.00041355	0.00176179
arsF	-0.8706558	4.70229901	31.1346317	0.00042338	0.00180192
nupO	0.73114396	7.90811462	31.04616	0.00042752	0.00181777
xepA	1.31105127	6.3160174	31.0061703	0.0004294	0.00182403
brnQ	0.84453455	5.1765672	30.9714312	0.00043105	0.00182886
ycgL	1.20672374	2.96955804	30.9646934	0.00043137	0.00182886
yycC	0.73877154	7.44960347	30.8968143	0.00043462	0.00184084
srfP	0.58163245	10.9962206	30.8416534	0.00043727	0.00185032
B4U62_RS155	-0.9757676	4.4557442	30.8007126	0.00043926	0.00185539
yxjM	0.95170273	6.2385102	30.7996223	0.00043931	0.00185539
essC	0.9341141	9.06763296	30.7621264	0.00044114	0.00186133
B4U62_RS229	-0.711742	6.18800555	30.7335441	0.00044254	0.00186545
ftsA	0.6545445	10.1597927	30.7206115	0.00044318	0.00186634
B4U62_RS068	-0.7144872	6.82461732	30.5409019	0.00045213	0.00190221
yrzB	-0.7540387	6.74916339	30.2986272	0.00046455	0.0019526
B4U62_RS138	0.83178327	7.50601287	30.2890721	0.00046505	0.00195284
B4U62_RS114	-0.9834342	4.15103405	30.2474714	0.00046722	0.00196011
ypjD	-1.0894308	2.7091222	30.1990925	0.00046977	0.00196893
ytrC	0.71285463	5.81966675	30.1475708	0.00047251	0.0019785
yvdD	-1.1022409	4.60736041	30.059407	0.00047723	0.00199638
yrhG	1.72370139	4.39801526	30.0269188	0.00047898	0.00200182
apt	-0.6211846	6.16108942	29.9693797	0.00048211	0.00201298
ruvX	-0.6416104	6.8877548	29.9149039	0.0004851	0.00202353
ywiE	0.82900409	9.61753559	29.8933346	0.00048628	0.00202657
B4U62_RS230	0.93278669	7.385101	29.8453543	0.00048894	0.00203457
yyzM	-1.2907922	2.01168916	29.8378561	0.00048936	0.00203457
bcd	-0.6255249	6.92883574	29.8336543	0.00048959	0.00203457
deoR	-0.7342986	6.2404535	29.7766516	0.00049277	0.00204529
xynA	-0.8516577	6.73961086	29.7660386	0.00049337	0.00204529
xynP	1.36256011	4.30544884	29.7625924	0.00049356	0.00204529
murR	0.76016037	5.58822211	29.6739692	0.00049857	0.00206411
B4U62_RS096	-0.7553592	5.04660362	29.5233991	0.00050723	0.00209799
yraI	0.7542203	5.64323301	29.4978421	0.00050872	0.0021009
yddF	2.15210962	3.04296918	29.4942832	0.00050893	0.0021009
murD	-0.712091	7.69467196	29.481645	0.00050967	0.0021009
B4U62_RS137	0.86008658	8.69807072	29.4691582	0.0005104	0.0021009
liaL	1.19307213	8.75729594	29.464039	0.0005107	0.0021009
uglF	-0.648002	6.05161625	29.4623103	0.0005108	0.0021009
yhcA	-0.945473	6.71388229	29.4421418	0.00051198	0.00210381
pyrC	-1.3631226	6.16040136	29.4057958	0.00051413	0.00211064
yxeM	1.72634341	4.63981417	29.3848672	0.00051536	0.00211376
rae1	-0.6305936	6.22548106	29.3704512	0.00051622	0.0021153
yxxB	0.78165254	5.76913289	29.3094708	0.00051986	0.00212823
ysnB	0.78834524	8.0181183	29.2758217	0.00052188	0.00213449
slrR	2.572223	1.9157188	29.2620782	0.00052271	0.00213449

Table S5. Genes differentially expressed between ΔmnmE and WT in transcriptome analyses

icaC	0.8734625	3.66093782	29.2480329	0.00052355	0.00213449
ecfAB	-0.7584519	6.90738263	29.245957	0.00052368	0.00213449
xkdW	1.1616438	5.59461485	29.2437927	0.00052381	0.00213449
coiA	-0.7820071	4.69102385	29.0908428	0.00053316	0.00216954
ispH	-0.5907433	6.42806462	29.0870457	0.0005334	0.00216954
sipV	-0.6080772	6.10659488	29.0277591	0.00053708	0.00218251
B4U62_RS225	-1.4095466	4.08093402	28.9956897	0.00053909	0.00218864
ycsA	-0.8861854	3.50059793	28.9190152	0.00054392	0.00220622
ydcH	-0.7667707	4.67195018	28.8663579	0.00054727	0.00221776
ylqB	-1.1898159	8.31737059	28.8248664	0.00054992	0.00222508
ypqE	-0.9043015	4.76362179	28.8223861	0.00055008	0.00222508
yjbK	-0.6718193	5.64759782	28.8129131	0.00055069	0.0022255
mleA	-0.6557259	6.05618728	28.6728827	0.00055979	0.00225845
maeN	1.03079043	5.24985402	28.6717257	0.00055987	0.00225845
yxjI	1.16103359	6.13386032	28.6439259	0.0005617	0.00226352
yhfT	0.92338055	7.82892057	28.6370209	0.00056215	0.00226352
B4U62_RS092	1.21165262	1.7722295	28.627141	0.00056281	0.00226408
sigX	0.68452099	7.25490434	28.5957105	0.00056489	0.00226917
akrN	0.78435251	9.22475137	28.5924727	0.0005651	0.00226917
yfhB	0.6498288	7.67384622	28.4930612	0.00057176	0.00229107
liaK	1.51961668	7.99160089	28.4876986	0.00057212	0.00229107
yjdG	0.82161647	5.54561747	28.469966	0.00057332	0.00229378
B4U62_RS028	1.68260144	2.43156367	28.3272806	0.00058307	0.00233068
B4U62_RS114	-3.051731	-0.213388	28.2952775	0.00058528	0.00233654
ylbP	-0.8438859	4.81546154	28.2907939	0.00058559	0.00233654
yezD	2.58323838	2.5611632	28.2210733	0.00059046	0.00235382
yxjN	1.02647858	5.50880508	28.2128449	0.00059104	0.00235399
lnrK	-0.8089509	4.65828453	28.1123211	0.00059815	0.00237801
ylaH	0.64716278	7.30562644	28.1043339	0.00059872	0.00237813
ganR	-0.7631821	4.68180303	28.0645806	0.00060156	0.00238728
B4U62_RS058	-0.9217508	2.96068532	28.0555952	0.00060221	0.00238769
accD	-0.8664412	8.10736188	28.0367386	0.00060356	0.00239091
ygxA	0.80798049	8.03439225	28.005417	0.00060583	0.00239772
brxA	-0.7184712	6.15925395	27.971693	0.00060827	0.00240525
yuiA	1.405362	10.3246141	27.9160947	0.00061234	0.00241697
B4U62_RS111	-1.9917597	0.58612197	27.8982605	0.00061365	0.00241913
glcD	1.39036393	5.87681051	27.8937203	0.00061398	0.00241913
fxsA	-0.6896036	7.01223931	27.8307944	0.00061863	0.00243528
mobA	-0.8944125	4.28255948	27.8032483	0.00062069	0.00244118
acpP	-0.8423878	7.25170779	27.7906653	0.00062162	0.00244133
lytA	-1.2674344	5.4637328	27.7878893	0.00062183	0.00244133
hemW	-0.739657	5.91011686	27.7692911	0.00062323	0.00244462
skfA	1.34088307	5.76903161	27.7132799	0.00062744	0.00245897
B4U62_RS194	1.41667784	2.1655225	27.6968693	0.00062868	0.00246165
glcF	1.40787171	5.76266991	27.6574911	0.00063168	0.00247117

Table S5. Genes differentially expressed between ΔmnmE and WT in transcriptome analyses

B4U62_RS103	-0.7651068	5.1322799	27.6039638	0.00063578	0.0024843
yrrS	-0.6521006	7.17721534	27.5987343	0.00063618	0.0024843
nfeDB	1.6127288	8.4155642	27.5916209	0.00063673	0.0024843
yktD	0.71600739	4.85824747	27.5651963	0.00063876	0.00249005
acuB	-0.8465555	6.13252822	27.5510576	0.00063986	0.00249211
bioD	0.7720247	8.91958083	27.4984295	0.00064395	0.00250517
opcR	0.96740349	5.2932237	27.4933174	0.00064435	0.00250517
yotL	-1.2289351	3.83110574	27.4618307	0.00064682	0.00251255
B4U62_RS028	2.08171579	2.35889328	27.3469951	0.00065591	0.00254412
ywdI	-1.0268123	3.9719522	27.3128405	0.00065865	0.0025495
ppiB	-0.8113425	7.11205563	27.3031289	0.00065943	0.00255028
yjbB	0.61330487	5.49120991	27.2956453	0.00066003	0.00255037
splB	-1.0052263	4.39267284	27.2607989	0.00066284	0.00255563
yflB	0.87003004	4.83718277	27.2572292	0.00066313	0.00255563
ycgS	1.00864387	4.19836609	27.243299	0.00066426	0.00255774
floT	1.49147551	9.37888095	27.2244506	0.00066579	0.00256088
spoVD	-1.2829844	3.4262749	27.2189843	0.00066624	0.00256088
yybL	1.53036969	6.32009589	27.1531859	0.00067162	0.00257933
ybzH	0.91161047	5.00096581	27.1391138	0.00067278	0.00258153
xpt	1.37280019	4.86261183	27.1241652	0.00067401	0.00258401
B4U62_RS232	-1.0550183	3.71692962	27.0843814	0.00067731	0.00259387
mneS	-0.7411134	6.44766474	27.0789258	0.00067776	0.00259387
mdtK	-0.6499237	5.46186799	27.0661106	0.00067883	0.0025957
rsmE	-0.699902	6.30258258	27.0473974	0.00068039	0.00259942
tagD	-1.1588196	6.41952328	27.0082294	0.00068368	0.0026083
cesA	-1.0439744	6.54273521	26.9946478	0.00068482	0.00260954
fmnP	0.95853073	7.03687625	26.9127004	0.00069177	0.00263373
moaB	-1.2133949	2.49528189	26.8533928	0.00069685	0.0026508
gerPF	1.874922	1.15391809	26.8122958	0.0007004	0.002662
yugH	-0.7181927	7.84356897	26.787108	0.00070259	0.00266801
ade	0.77004948	6.91330847	26.7708362	0.000704	0.00267108
ssuD	-1.8155959	1.87704125	26.740756	0.00070663	0.00267875
bshA	-0.7034937	6.08561445	26.6228735	0.00071705	0.00271312
qoxA	-0.8468097	11.4159769	26.6124083	0.00071798	0.00271312
bsrE	-1.0991731	7.10974978	26.610438	0.00071816	0.00271312
yomG	-1.1039931	3.82729767	26.4571649	0.00073202	0.00276311
rplD	-0.5927384	10.8743384	26.4111365	0.00073625	0.00277668
ppaC	0.58277856	8.61892178	26.1479645	0.000761	0.00286269
yyaE	-0.8000164	5.29813136	26.1073568	0.00076491	0.002872
B4U62_RS189	1.08237318	2.25302016	26.1051229	0.00076513	0.002872
B4U62_RS187	1.13874329	5.53019053	26.1019726	0.00076543	0.002872
walR	-0.5842231	7.38838018	26.0948882	0.00076612	0.00287212
sdaAA	-0.7472838	7.23682137	25.9467724	0.00078062	0.00292153
addA	-0.6010819	9.00859183	25.9186598	0.00078341	0.00292949
rpmGB	0.70442688	8.98667757	25.9089985	0.00078438	0.0029306

Table S5. Genes differentially expressed between ΔmnmE and WT in transcriptome analyses

glgC	0.94105568	3.58014535	25.8660234	0.00078867	0.00294416
hemL	0.68657091	9.51165267	25.8541474	0.00078987	0.00294611
lytC	-0.7288266	8.00518213	25.8348142	0.00079181	0.00294828
B4U62_RS102	1.47651692	2.41387369	25.8277674	0.00079252	0.00294828
gmuG	0.72158919	4.41500541	25.8234932	0.00079296	0.00294828
yusD	0.58784242	6.16705021	25.8218243	0.00079312	0.00294828
yhfS	0.95244368	7.25640202	25.7585071	0.00079955	0.00296967
yxxE	1.12918422	5.40882513	25.6799477	0.00080762	0.00299711
ykkB	0.85709827	4.63135158	25.5507559	0.00082111	0.00304366
B4U62_RS177	1.87535374	0.43786841	25.5402748	0.00082221	0.00304366
buk	-0.6619562	6.48959577	25.5400601	0.00082224	0.00304366
rocG	-0.9310058	3.77287851	25.5211566	0.00082424	0.0030485
sigY	-0.8162291	4.9275117	25.456344	0.00083114	0.00307122
B4U62_RS228	-1.493284	2.11432126	25.4504222	0.00083177	0.00307122
yrzA	-1.8565033	1.49437075	25.4220971	0.00083482	0.00307987
coaD	-1.3052869	3.40299133	25.2947994	0.00084866	0.00312607
xkdX	1.19261495	4.65487592	25.2939111	0.00084876	0.00312607
ywoG	1.1856154	5.93468537	25.2237301	0.00085651	0.003152
yckA	-0.9627389	4.05562288	25.2088588	0.00085817	0.00315545
tagE	-0.7689962	8.10597973	25.1805891	0.00086132	0.00316441
yuiD	0.64448722	7.20845656	25.1241934	0.00086766	0.00318505
prfA	-0.7999946	7.70482724	25.0818634	0.00087246	0.00319796
fhuB	0.76012392	6.35036745	25.0744431	0.0008733	0.00319796
yvaM	0.83895119	4.40483803	25.0739663	0.00087336	0.00319796
dacB	-1.1458894	1.81874187	24.9975784	0.00088211	0.00322731
fliJ	-0.9245462	7.02350031	24.9707859	0.0008852	0.00323104
tenI	-1.0629101	2.06277999	24.9698273	0.00088531	0.00323104
bdbD	-0.6696311	8.57884907	24.9697134	0.00088533	0.00323104
plsX	-0.958213	8.31430718	24.9349661	0.00088936	0.00324307
rocA	-0.7034156	7.57536892	24.8853286	0.00089516	0.00326153
fatR	-0.6645779	5.52762273	24.7249167	0.00091424	0.00332827
yuiC	0.67100644	6.70550392	24.7099007	0.00091605	0.00333211
sinI	0.70082968	5.84628016	24.6340053	0.00092527	0.00336011
yxiB	1.26302316	4.34680688	24.577028	0.00093227	0.00338274
ytlI	1.65511775	3.32888946	24.5039305	0.00094134	0.00341286
opuAC	-0.7060792	8.6287506	24.4932657	0.00094268	0.00341366
B4U62_RS103	1.81103933	7.7555477	24.5069433	0.00094311	0.00341366
ypmP	0.61486316	8.78671108	24.4527084	0.00094777	0.00342768
ywrE	0.80977204	5.94737101	24.4220842	0.00095164	0.00343885
yisT	0.7449568	6.83628418	24.3826075	0.00095665	0.00345413
rsbV	0.78199636	10.5737506	24.3642561	0.00095899	0.0034581
sdpB	1.21685925	9.98218976	24.3616987	0.00095932	0.0034581
mrpA	-0.6315731	7.277615	24.3550438	0.00096017	0.00345834
mdh	0.7108825	10.3414015	24.3373811	0.00096243	0.00346366
cutI	-0.9836129	10.2367284	24.3290748	0.0009635	0.00346467

Table S5. Genes differentially expressed between ΔmnmE and WT in transcriptome analyses

rbsK	-0.9654452	4.54289885	24.3133611	0.00096552	0.00346911
ylbG	-0.8717867	5.3022832	24.2886989	0.00096871	0.00347772
yugG	-0.7297104	7.03348715	24.2776144	0.00097014	0.00348003
yvbG	1.02539485	5.79415995	24.2592525	0.00097253	0.00348575
alaS	-0.8460904	7.32657136	24.1950121	0.00098093	0.00351299
rsuA	-0.7450616	4.29541763	24.1285593	0.00098971	0.003537
pucG	1.20356876	5.06750085	24.1177906	0.00099114	0.00353807
yetN	-1.0336444	3.66444897	24.0870422	0.00099525	0.00354984
purN	1.06285289	7.85801945	24.0733503	0.00099708	0.00355351
pstBA	-1.2078183	2.14337584	24.0224641	0.00100393	0.00357285
lip	1.13161201	4.88001146	24.0210015	0.00100413	0.00357285
rnpA	-0.6738748	5.98942401	23.9351356	0.00101583	0.00361048
ywmD	0.8802639	3.47770906	23.8806955	0.00102334	0.00362947
ung	-0.9128169	5.3245159	23.8589003	0.00102636	0.00363726
argB	0.83953756	9.00478667	23.8528194	0.00102721	0.00363733
mgsA	-0.9517968	5.17778245	23.8357204	0.00102959	0.00364284
pelA	-1.2249771	6.2538333	23.7956671	0.0010352	0.00365975
yngB	-0.9060477	4.57049935	23.7513201	0.00104145	0.00367595
msmX	-0.9877159	6.03893761	23.6971218	0.00104916	0.00370019
B4U62_RS222	-1.0216797	5.68693487	23.6450281	0.00105663	0.00372357
secE	0.75708621	8.8885434	23.6309023	0.00105867	0.00372762
metA	0.61893789	7.31209786	23.6253302	0.00105947	0.00372762
yutD	-0.9438805	4.71135868	23.5657063	0.00106814	0.0037551
ydeE	0.78426482	3.81257773	23.5517563	0.00107018	0.00375927
ypeP	0.69476758	5.52371916	23.5213464	0.00107464	0.00377194
B4U62_RS043	1.14529924	4.16075906	23.5087184	0.0010765	0.00377546
purM	1.07513172	8.33824525	23.4391673	0.00108682	0.00380861
ylxF	-0.9587431	7.72016731	23.412774	0.00109076	0.00381941
glpP	-0.6730361	5.61273736	23.3884421	0.00109442	0.00382783
ureB	1.43330284	5.22380115	23.3851847	0.00109491	0.00382783
yybH	-1.0367185	3.73311105	23.3634025	0.00109819	0.00383007
yoaE	1.00351671	7.77186973	23.3574713	0.00109909	0.00383007
ureA	1.6411615	4.52350648	23.3521605	0.0010999	0.00383007
B4U62_RS144	-0.8977991	8.02115209	23.3379484	0.00110205	0.00383454
yczE	-0.9978827	4.95363187	23.3066174	0.00110682	0.0038481
glmS	-0.7076804	10.8060458	23.142967	0.00113215	0.00393305
yrrB	-0.7853965	5.28766079	23.0466905	0.00114738	0.00398284
B4U62_RS114	-1.102036	4.3009885	23.0301164	0.00115003	0.00398889
eda	-0.9828095	2.8471409	23.0197255	0.0011517	0.00399152
ywhK	0.87726559	2.91650815	22.835673	0.00118168	0.00408578
prmA	-0.6366943	6.85543145	22.7928629	0.00118879	0.00410711
sxzY	1.08372227	7.1752562	22.787316	0.00118971	0.00410711
qdoI	0.63097601	5.59907877	22.7272945	0.00119978	0.00413863
B4U62_RS102	0.92711352	2.54722201	22.7139296	0.00120204	0.00414317
rsbRC	-0.7158362	4.8486443	22.6490162	0.00121307	0.00417793

Table S5. Genes differentially expressed between ΔmnmE and WT in transcriptome analyses

ydgE	0.61223231	5.5835057	22.5744563	0.0012259	0.00421553
B4U62_RS134	0.86011614	3.48718683	22.5615249	0.00122814	0.00421596
yjdB	0.88454484	8.41473456	22.5588234	0.00122861	0.00421596
lysA	0.69914555	7.90863389	22.5303091	0.00123358	0.00422872
ydcG	-0.6524439	4.66168558	22.4133592	0.0012542	0.00429273
mdtR	-0.8313235	4.08228712	22.377932	0.00126053	0.00431105
B4U62_RS105	1.54504922	2.31257632	22.3516494	0.00126525	0.00432384
B4U62_RS231	-1.5306278	3.73879683	22.3172849	0.00127146	0.00433833
yolA	-1.1773094	7.6737713	22.2594156	0.001282	0.00437039
ycxD	0.75268994	3.9301395	22.2548207	0.00128284	0.00437039
yodE	-0.9150561	3.43778636	22.2036492	0.00129226	0.00439567
dltC	0.65605329	7.50753131	22.1978322	0.00129333	0.00439594
mstX	1.04749668	4.8469907	22.1654398	0.00129935	0.00441052
trmK	-1.0892764	4.78546227	22.1599449	0.00130037	0.00441052
dfrA	-0.5877717	6.44134404	22.1585506	0.00130063	0.00441052
yobO	0.59133898	6.28738614	22.0238769	0.00132603	0.00448974
gltR	0.77685958	4.2142201	21.9909413	0.00133233	0.00450762
opuAA	-0.7208318	9.16139831	21.9812153	0.0013342	0.00451048
yvaG	-0.6401316	5.12738906	21.9513765	0.00133995	0.00452515
yrzL	-0.6617556	6.13127188	21.9355393	0.00134302	0.00452987
spoIISA	-0.6105492	6.45127939	21.8773035	0.00135437	0.00456466
B4U62_RS056	-1.1920225	3.97767937	21.7612325	0.00137735	0.00463502
yitK	0.60321488	6.62039138	21.6728103	0.00139518	0.00468862
yrbC	-0.876544	2.72782366	21.6716839	0.00139541	0.00468862
yufK	-0.9734419	5.46502493	21.6579363	0.00139821	0.00469445
yxkI	-0.7139903	6.66165974	21.5890386	0.00141234	0.00473828
B4U62_RS221	-1.4038052	6.04018089	21.5747657	0.00141529	0.00474456
frr	-0.8742211	7.11870977	21.5306116	0.00142446	0.00476805
kbl	-1.1019121	3.47052833	21.5019333	0.00143046	0.00478448
yqaT	-1.0014365	2.50222271	21.4914754	0.00143265	0.00478819
bsuRC	0.67120407	7.44890967	21.4163678	0.00144854	0.00483394
icd	0.58783081	10.6792466	21.3876812	0.00145466	0.00484749
clpE	-0.9911347	6.10839922	21.3851787	0.0014552	0.00484749
yjlA	0.67554595	5.26410234	21.2478657	0.00148497	0.00493683
rlbA	-0.8906815	4.89354844	21.2045366	0.00149452	0.00496484
bioB	0.67716519	8.91754505	21.1907121	0.00149759	0.00497127
gspA	0.84902372	11.6364934	21.1465058	0.00150744	0.0049937
scrA	-0.9869552	3.39426225	21.139746	0.00150895	0.0049937
yvrA	-0.6297615	5.06056941	21.1394297	0.00150902	0.0049937
yxlE	-0.8269678	4.20981357	21.1351019	0.00150999	0.0049937
yoaU	0.98774811	2.49648305	21.1349937	0.00151001	0.0049937
tdk	-0.6897713	4.60956145	21.0849354	0.00152129	0.00502721
degR	-1.2551263	2.42168066	21.0467394	0.00152996	0.00505208
ydeN	0.67788609	5.30090348	21.0375435	0.00153206	0.00505415
sdpR	1.00026894	8.4708883	21.0209901	0.00153584	0.00505634

Table S5. Genes differentially expressed between ΔmnmE and WT in transcriptome analyses

yqbF	-1.459187	1.84718835	21.0075077	0.00153893	0.00506273
rpsG	-0.5961846	10.9523082	20.949505	0.00155231	0.00509914
amyE	-0.5868536	6.44202499	20.8622562	0.00157272	0.00516231
rocR	0.66594692	5.71094008	20.7840362	0.00159129	0.00521551
maeM	-0.7056215	6.03747084	20.7730965	0.00159391	0.00522021
ylxY	-0.7131096	5.37829553	20.7653296	0.00159578	0.00522243
fur	0.81262258	5.79587078	20.7484214	0.00159984	0.00523185
B4U62_RS034:	1.00477137	3.88923693	20.7411289	0.0016016	0.00523371
estB	-1.0386226	3.61883494	20.703573	0.00161069	0.00525517
yuaI	1.53841453	6.94742994	20.6978598	0.00161207	0.00525517
yycA	0.68267452	6.69080028	20.694299	0.00161294	0.00525517
plsC	-0.5873811	6.70452401	20.6782008	0.00161686	0.00526406
B4U62_RS231(	-1.6542169	2.6002006	20.5731019	0.00164276	0.00534442
ykkA	0.83889009	5.26141765	20.5600412	0.00164601	0.00535105
ypfB	-1.208778	3.08500413	20.5522484	0.00164795	0.00535342
ydzA	-1.3775514	5.63598792	20.5047046	0.00165988	0.00538615
purD	0.93282374	8.77220042	20.4985701	0.00166143	0.00538615
ykuS	0.72235057	4.89763064	20.4975177	0.0016617	0.00538615
bioYB	1.07392329	8.28946349	20.4549167	0.00167249	0.00541715
kdgT	-0.8135328	3.61814095	20.4482822	0.00167418	0.00541864
ftsX	-0.646481	7.81791359	20.3117971	0.0017094	0.0055245
yobU	-1.7754066	0.39032772	20.2161724	0.00173462	0.00560191
yvgT	-0.8908599	3.31230764	20.1966226	0.00173983	0.00561052
allC	1.21156975	5.16481061	20.1696398	0.00174706	0.0056297
ybzG	-0.7021883	8.79532996	20.1600157	0.00174965	0.00563391
B4U62_RS216·	0.75027213	4.21392134	20.1538065	0.00175132	0.00563518
ykaA	-0.6814696	7.66804203	20.1465483	0.00175328	0.00563735
cwlC	0.93215231	2.90949296	20.1366648	0.00175594	0.00564182
yncC	-0.8912826	3.15640153	20.0997211	0.00176596	0.005667
msmF	1.00093489	5.16778436	20.0842544	0.00177018	0.00567513
yceF	0.74922781	10.0405801	20.0786339	0.00177171	0.00567592
yjgA	-0.6270571	4.62832152	19.9772049	0.00179969	0.00575591
yqjX	-1.6298226	0.7066457	19.9725432	0.00180099	0.00575591
yueI	-0.6217343	5.47047765	19.9692738	0.00180191	0.00575591
cotNH	-0.6733285	4.31649534	19.9502404	0.00180723	0.00576594
ydbS	0.62546446	7.64915199	19.9486728	0.00180767	0.00576594
sdpA	1.50004921	8.45858515	19.9407128	0.0018099	0.00576888
ylaJ	0.81869306	2.59333657	19.9087098	0.0018189	0.00578921
yofA	0.71329854	5.11830874	19.7785162	0.00185612	0.00590098
ydeF	0.74587231	4.95842602	19.7765063	0.0018567	0.00590098
frxB	1.39385019	6.26463418	19.7455389	0.00186569	0.00592101
fliK	-0.7228769	8.84600744	19.7359104	0.0018685	0.00592565
yxxD	1.31260982	5.31991873	19.6715635	0.00188739	0.00598127
yxeQ	1.22594061	4.91825604	19.6636694	0.00188973	0.00598437
yvrD	0.73800634	5.00653171	19.6485925	0.0018942	0.0059899

Table S5. Genes differentially expressed between ΔmnmE and WT in transcriptome analyses

yybN	0.86491516	10.37518	19.5680246	0.0019183	0.00605742
niaR	0.65038925	9.2908297	19.5349971	0.00192829	0.00607598
B4U62_RS115	-1.3217598	1.28554269	19.5349127	0.00192831	0.00607598
fabD	-0.6667923	7.78194832	19.5228307	0.00193198	0.00608319
pstS	-1.5274635	2.49907743	19.4898244	0.00194206	0.00611054
yueH	-0.6486202	4.93694787	19.4423788	0.00195665	0.00614767
uxaB	-1.0742223	1.64311588	19.4371042	0.00195828	0.0061484
B4U62_RS208	1.1216199	4.82988618	19.4308818	0.0019602	0.00615007
chrS	1.08462159	3.65147758	19.3992542	0.00197003	0.00617477
ilvK	0.58129639	9.40717292	19.3965142	0.00197088	0.00617477
atpB	-0.8055083	10.1035379	19.3135577	0.00199696	0.00624314
ycnC	0.6802172	6.29316994	19.2745978	0.00200935	0.00627743
yqgY	-0.7641528	4.48320079	19.2548488	0.00201567	0.00628428
yvdB	0.72064951	4.31575137	19.2543672	0.00201582	0.00628428
gerKA	1.00044607	2.03639695	19.2221826	0.00202617	0.00631208
yvrB	-0.645965	5.79124862	19.2115992	0.00202959	0.00631826
recG	-0.6394318	7.97966819	19.2056044	0.00203153	0.00631983
B4U62_RS038	1.14264849	1.67139795	19.1927853	0.00203568	0.00632383
yqfT	1.32183406	1.14376969	19.1511736	0.00204924	0.00635698
B4U62_RS225	-0.9288238	4.73379043	19.1147274	0.00206121	0.00638961
purH	0.9514764	9.33091725	19.0803521	0.00207258	0.00641925
yvqK	-0.7855674	3.86895856	19.0706278	0.00207581	0.0064213
rdgB	0.58547836	7.66496078	19.0327084	0.00208846	0.00645591
ykyA	-0.9520215	3.41135761	18.9616956	0.00211242	0.0065208
purS	1.0538057	7.10598536	18.9491456	0.00211668	0.00652941
argD	0.75470182	10.0003231	18.9400464	0.00211979	0.0065344
yfmC	0.65727933	7.82367941	18.9077114	0.00213085	0.00656392
yfkF	-0.6674264	5.43233935	18.9008585	0.00213321	0.00656658
efeU	0.79395004	6.46782877	18.8801075	0.00214036	0.00658399
rsgI	-0.736909	5.74047986	18.874859	0.00214217	0.00658497
B4U62_RS169	0.62977432	6.12070885	18.8691416	0.00214415	0.00658646
sucD	-0.6107973	8.57259684	18.7333871	0.00219177	0.00670935
xkdV	0.88321025	7.40625214	18.6853379	0.00220894	0.00675469
yusN	1.23787271	0.86887248	18.6833622	0.00220965	0.00675469
B4U62_RS225	-0.8109756	3.51251428	18.6478876	0.00222244	0.00678907
spaC	1.15730152	2.29190475	18.5843282	0.00224558	0.00683606
yxkF	-0.6597944	4.31744879	18.560185	0.00225445	0.00685832
coxB	-0.772514	8.6616705	18.486674	0.00228172	0.00693649
comGD	1.31583847	1.63766992	18.4337313	0.00230161	0.00698733
dapF	0.58060052	5.94645364	18.4267165	0.00230427	0.00699056
B4U62_RS055	1.59573458	2.39929354	18.3634004	0.00232838	0.00705399
yvcD	-0.6582657	6.63376503	18.3347129	0.0023394	0.00708252
spoIVCA	0.77157368	4.84143173	18.316793	0.00234632	0.0070986
rsmD	-0.9850304	4.31426394	18.3096998	0.00234907	0.00710203
tsaE	-0.5807135	4.77824745	18.2695961	0.00236467	0.0071443

Table S5. Genes differentially expressed between ΔmnmE and WT in transcriptome analyses

yxaH	0.63574629	5.50279683	18.2490291	0.00237273	0.00716371
ynzC	-0.7482531	5.8102392	18.1986619	0.00239259	0.00721873
pyrE	0.615561	7.38885443	18.1854508	0.00239783	0.0072296
B4U62_RS096:	-1.2993301	2.42774454	18.1781391	0.00240074	0.00723342
B4U62_RS072:	-1.1968283	5.93662888	18.1381968	0.0024167	0.00727157
yppF	-0.674799	5.03835694	18.084479	0.00243838	0.00731681
ndoA	-0.6368946	8.58067801	18.0691563	0.00244461	0.0073305
rhgP	-1.2521032	0.75910426	18.0536667	0.00245093	0.00734444
ydcL	1.03800887	5.92350133	18.0382536	0.00245723	0.00735833
uvrX	-1.2807912	2.68173632	18.0056827	0.00247062	0.0073934
yybS	-0.5860607	7.67535016	17.9797854	0.00248133	0.00742041
yvaD	0.82967071	2.81875409	17.963702	0.00248801	0.00743534
yxlD	-0.8673901	4.21768168	17.9374266	0.00249897	0.00745798
B4U62_RS173:	-1.6553981	0.16187664	17.9146024	0.00250854	0.00747643
spx	0.6069146	10.1852286	17.9145909	0.00250855	0.00747643
fabG	-0.6518958	7.75672239	17.881433	0.00252253	0.00750795
yrhF	-0.898191	2.3983523	17.8657702	0.00252917	0.00751755
yflA	0.96055023	10.994777	17.8371022	0.00254138	0.00753858
adhR	-0.7350357	2.63634197	17.7758181	0.00256773	0.0076116
B4U62_RS038	0.72738323	3.74206297	17.7623732	0.00257355	0.00761862
B4U62_RS225:	-1.2087489	1.1923481	17.7380284	0.00258414	0.00764483
albA	-0.9031896	12.5007331	17.7235843	0.00259045	0.00765834
licA	-1.5091791	3.36179458	17.6710403	0.00261356	0.00771966
yqbB	-1.1182291	1.54773586	17.6646853	0.00261637	0.00771966
sacT	1.06119597	3.57357214	17.6605562	0.0026182	0.00771966
malP	1.48592556	4.52453489	17.6508903	0.00262249	0.0077248
gerR	-1.7369458	1.29280109	17.6487218	0.00262345	0.0077248
B4U62_RS116	-0.7702601	4.4424516	17.5666722	0.00266022	0.0078174
kbfO	1.08659144	5.79542397	17.520524	0.00268119	0.00785818
pbpI	-0.6635327	4.17373292	17.473272	0.00270286	0.00791629
B4U62_RS157:	-0.8336917	9.49886328	17.4676704	0.00270545	0.00791859
dacF	1.27368824	1.14148643	17.44802	0.00271453	0.00793992
B4U62_RS103	2.63857913	-0.0673538	17.4275638	0.00272404	0.00796243
hisJ	0.60850331	7.17157191	17.4082538	0.00273304	0.00798346
B4U62_RS187:	0.77159871	7.40485062	17.33253	0.00276872	0.00805731
sigB	0.82843582	12.5679687	17.3322899	0.00276884	0.00805731
yhjA	-0.6297562	6.75962478	17.3230356	0.00277324	0.00805731
motS	-1.0402204	2.2148222	17.3225653	0.00277346	0.00805731
ywoH	0.93144721	4.84062555	17.3197803	0.00277479	0.00805731
mtlR	-0.6454044	5.92917602	17.2506665	0.00280795	0.00814823
ganS	-0.7407659	3.57185376	17.2204873	0.00282258	0.0081853
recR	-0.6541163	5.56666046	17.2038693	0.00283068	0.00820338
yhbB	-1.1289199	1.41518177	17.1966755	0.00283419	0.00820817
liaD	1.44933699	9.58293479	17.0374623	0.00291338	0.00841538
yrpDX	-0.6620608	4.37516949	17.0218117	0.00292131	0.00843276

Table S5. Genes differentially expressed between ΔmnmE and WT in transcriptome analyses

albB	-0.7881351	9.83500125	17.0051764	0.00292977	0.00845164
pgsC	1.48869391	0.58231676	16.949489	0.00295832	0.00852281
xkdU	0.93214073	6.44783877	16.908828	0.00297937	0.00856108
yqiI	1.09033218	1.79898071	16.8915832	0.00298836	0.00858131
txpA	-1.1292362	2.01154947	16.8850534	0.00299177	0.00858551
B4U62_RS111	-1.9876818	0.21695612	16.866893	0.00300129	0.00860561
spo0F	0.80032892	7.02479729	16.836038	0.00301754	0.00864255
lmr(B)	0.6395465	6.22441103	16.7443618	0.00306647	0.00877129
gapB	1.59041998	4.39889565	16.7205175	0.00307935	0.00880243
spo0E	0.98208137	7.63357712	16.7048518	0.00308786	0.00882102
rsiV	0.85185067	2.09334779	16.6752454	0.00310401	0.00886141
yjfB	-1.0893256	4.73393816	16.6590883	0.00311287	0.00888094
iscSB	-0.5986256	5.69406547	16.5114585	0.00319527	0.00909248
rbsD	-1.1746058	2.76682188	16.4920115	0.00320632	0.00911805
B4U62_RS051	-0.7601143	4.64787358	16.431715	0.0032409	0.0092045
B4U62_RS056	-0.9464519	4.51564352	16.3948331	0.00326228	0.00924804
yuzF	0.70869411	5.80121743	16.3944077	0.00326253	0.00924804
spoIVA	0.716858	3.71551801	16.2946572	0.00332124	0.00940841
liaH	0.65437225	3.81755254	16.2162599	0.00336831	0.00952337
ytcP	-1.2311755	0.88982789	16.2054562	0.00337486	0.00953577
B4U62_RS029	-0.8350454	5.03299228	16.0724182	0.00345683	0.00975432
thiC	-0.659765	4.84503702	16.0691899	0.00345885	0.00975432
ycbK	-0.8822231	3.80174839	16.0314934	0.00348255	0.009813
ypmA	-0.990165	3.38514247	15.9956947	0.00350523	0.00985357
bglS	-0.7591867	3.17543513	15.9790244	0.00351586	0.0098719
yoaG	0.84429867	4.66922124	15.9784356	0.00351623	0.0098719
catE	0.87069962	2.41199547	15.9643625	0.00352524	0.00989087
ebfC	-0.7300182	6.01998805	15.9352201	0.00354397	0.00993078
flgD	-0.7578842	7.27006865	15.8708111	0.00358582	0.01002891
yoaR	1.08383916	0.90332999	15.8618114	0.00359171	0.01003902
yqhO	-0.8079735	2.29822583	15.8271482	0.00361453	0.01009
yfhK	1.07561626	10.8554268	15.8173605	0.00362101	0.01009748
psdR	-0.6841671	5.04105733	15.8161719	0.0036218	0.01009748
yczM	1.42111925	1.88760502	15.7799676	0.00364589	0.01015821
pstC	-1.1228636	2.53942968	15.7109309	0.00369237	0.01026824
spoVT	0.70175368	6.90891236	15.5883103	0.00377676	0.01046987
birA	-0.7073487	5.29207053	15.5729653	0.00378749	0.01049301
opuAB	-0.6088221	8.87838574	15.5246137	0.00382154	0.01057405
xtmB	0.94353446	7.80059871	15.4084238	0.00390493	0.01079124
yxaF	-0.8264797	3.12408655	15.3626901	0.00393838	0.01085338
speD	-0.8311682	6.38229109	15.3617274	0.00393909	0.01085338
ydaD	-0.881978	7.17660891	15.3608371	0.00393974	0.01085338
holA	-0.7623071	5.04645859	15.2957893	0.00398794	0.01095875
glnM	1.68870444	0.9445347	15.264074	0.00401171	0.01101719
yfhI	-0.6588481	4.51198763	15.1923754	0.00406609	0.01115263

Table S5. Genes differentially expressed between ΔmnmE and WT in transcriptome analyses

ycxC	-0.7059104	3.32400612	15.1468212	0.00410111	0.01123471
yclI	-0.7934816	2.08710096	15.0768688	0.00415563	0.01136991
htpK	-0.7576855	5.5420175	15.0486314	0.00417789	0.01142372
yesK	-1.6317372	1.43824351	15.0409283	0.00418399	0.01142819
B4U62_RS225	-0.6696473	6.89075276	15.0168982	0.00420309	0.01146414
yhzC	0.77010622	7.54878127	15.0080691	0.00421013	0.01147625
B4U62_RS225	-0.8710449	7.60528446	14.9750572	0.00423659	0.01154124
pulA	-0.5918202	7.35421596	14.9693185	0.00424121	0.01154669
tenA	-0.9241263	2.05857303	14.9384051	0.00426622	0.01160042
yhfC	0.60970845	4.8436223	14.9090106	0.00429016	0.01165115
B4U62_RS218	-0.6140862	4.89426576	14.8943073	0.0043022	0.01167664
B4U62_RS109	-1.7247195	-0.4677565	14.8381503	0.00434857	0.0117686
ldeJ	1.17472354	1.50114299	14.8371074	0.00434944	0.0117686
spoIIGA	0.93193985	2.11817826	14.8237633	0.00436055	0.01179142
spoIISC	-0.674881	3.68790711	14.7852468	0.00439282	0.0118714
albC	-0.6425027	11.2417317	14.7307783	0.00443897	0.01198876
mmuM	0.71826939	5.21184893	14.7182092	0.0044497	0.01200428
yheA	-0.6932629	5.1874663	14.6770305	0.0044851	0.01208692
resC	-0.6298041	8.2912767	14.499167	0.00464204	0.01241597
ygaJ	0.71075085	4.03279244	14.4989348	0.00464225	0.01241597
yvzF	-0.7957739	3.27932195	14.4232121	0.00471113	0.01259256
yoaT	0.68667443	5.14300089	14.3586503	0.00477087	0.0127445
pghB	-0.6182627	4.38544971	14.349722	0.0047792	0.01275774
spoIIID	2.92433499	-1.2373127	14.3307604	0.00479696	0.0127832
rnmV	-0.6530219	4.23786334	14.3042358	0.00482195	0.01283424
msmG	0.94930772	5.14751113	14.2878349	0.00483747	0.01286475
yrzI	-0.5806783	3.94760961	14.285963	0.00483925	0.01286475
atpF	-0.7040932	8.91274136	14.272073	0.00485246	0.01289208
B4U62_RS117	-1.0273719	6.43696289	14.2640359	0.00486012	0.01289688
yhaQ	-0.7593866	4.93186306	14.253918	0.00486978	0.01290936
bdbB	1.12781935	6.60238379	14.2226712	0.00489979	0.0129631
glnH	1.15730076	1.67369475	14.1575471	0.00496306	0.01309902
glnQ	0.96464387	1.77994961	14.1377368	0.00498251	0.01314248
ssuA	-2.1066755	0.28729308	14.1274454	0.00499265	0.01316135
ykjA	1.12420316	1.29300039	14.104135	0.00501571	0.01321423
htpG	0.59629732	8.01929232	14.0827381	0.005037	0.01326238
ywsA	-1.128839	2.50218467	14.0210448	0.005099	0.01338564
blaOXA	0.82712516	3.47851141	13.9861227	0.00513452	0.01346283
kdgR	-0.623884	5.52046804	13.9712635	0.00514973	0.01348665
gerPB	0.89970169	1.25273804	13.9251441	0.00519728	0.01358772
ythB	0.85758993	2.93957674	13.924862	0.00519757	0.01358772
codY	-0.5904911	8.50091733	13.9081254	0.00521496	0.0136251
yesR	-0.8226841	1.70381829	13.8205331	0.00530718	0.0138414
B4U62_RS230	-0.7375302	5.40312768	13.8036833	0.00532515	0.01387184
gdnE	-1.7190897	2.15701653	13.7688296	0.00536256	0.01394455

Table S5. Genes differentially expressed between ΔmnmE and WT in transcriptome analyses

tatAC	-1.3916354	3.61449834	13.7037135	0.00543333	0.01411192
lrpA	-0.8767766	4.04923814	13.6793787	0.00546008	0.01417304
B4U62_RS209:	0.735241	7.5777743	13.6407227	0.0055029	0.01426124
rtp	0.62096745	3.24644052	13.6321065	0.0055125	0.01427547
alsR	0.64383374	5.14673448	13.6284247	0.00551661	0.01427772
comGB	0.79578562	2.80118479	13.6100082	0.00553722	0.01432265
yheG	-0.8452087	4.70300664	13.518017	0.00564162	0.01456703
fadN	-1.2102176	6.96560005	13.4460054	0.00572506	0.01476515
yoqW	0.59858409	5.57356328	13.3858594	0.00579592	0.01493042
lrgA	1.3986151	3.63188013	13.3312253	0.00586123	0.01508104
hdhQ	0.80911223	3.94750722	13.3044993	0.00589352	0.01515525
B4U62_RS029:	-0.6613247	3.93499348	13.282468	0.0059203	0.0151999
cotT	-1.233136	1.98704963	13.2801491	0.00592312	0.0151999
glnL	-0.6323022	4.00333308	13.2788693	0.00592468	0.0151999
rsbW	0.65315659	11.7640208	13.2548164	0.00595412	0.01526653
yxjF	-0.8999154	1.58790066	13.2344296	0.00597921	0.01532194
yjcD	-0.5960687	4.61047875	13.1927624	0.0060309	0.01542748
B4U62_RS141	-0.9624915	4.2084908	13.1329615	0.00610606	0.01561068
ywrK	0.75682548	2.46467018	13.1046204	0.00614209	0.01567858
sspJ	1.3710981	1.39296	13.0556565	0.00620496	0.01580846
malS	-0.5801978	5.27594368	13.0475041	0.0062155	0.01582617
ohrR	-0.8781849	2.3014524	13.0312189	0.00623663	0.01586775
flgG	-0.691244	8.06206084	13.0293729	0.00623903	0.01586775
sigE	0.74221977	2.10588651	13.0205507	0.00625052	0.01587863
dctB	-0.5867138	4.32523093	12.9215114	0.00638133	0.01617362
slrC	-0.8470299	3.71941399	12.8145767	0.0065264	0.01647495
yclH	-0.9424092	1.704786	12.8103556	0.00653221	0.01648017
ywnA	0.68938723	5.37062832	12.7683961	0.00659031	0.01660774
yoaP	0.80114929	3.40144656	12.7594	0.00660286	0.01662983
yycQ	0.66644091	3.18079304	12.7205712	0.00665733	0.01674216
acoA	3.03610969	2.35447164	13.1351016	0.00684618	0.01717393
ymzC	0.63870931	3.60947397	12.574535	0.00686726	0.017217
yjoB	0.66570532	6.61563549	12.5517344	0.00690077	0.01728135
ypzI	-1.6360504	-0.5182814	12.5478643	0.00690648	0.01728446
mntA	-0.8079092	3.74876489	12.5455725	0.00690986	0.01728446
xtmA	0.80602962	7.1201355	12.5379011	0.0069212	0.017303
ytaB	0.76379552	7.87125404	12.5130605	0.00695808	0.01738534
yxdJ	-0.6834368	3.59167242	12.4587924	0.0070395	0.0175788
ywlA	-0.5964404	4.27975005	12.4548895	0.0070454	0.01758357
spoVAA	1.90267789	-0.6972065	12.422511	0.0070946	0.01769387
xkdT	0.86984114	6.53281114	12.4205233	0.00709763	0.01769387
B4U62_RS227	-1.8911168	0.59573988	12.4132792	0.0071087	0.01770834
spdL	0.80028613	9.50659186	12.4114648	0.00711148	0.01770834
thiT	-0.9195946	2.24203351	12.3748425	0.0071678	0.01783851
yqbI	-1.486576	0.10465653	12.362964	0.00718619	0.01786409

Table S5. Genes differentially expressed between ΔmnmE and WT in transcriptome analyses

yvbK	0.65594351	3.34811709	12.3586033	0.00719295	0.01786885
yppD	1.12311998	1.64979215	12.3512819	0.00720433	0.01786885
yisN	1.38273796	0.63934919	12.3450751	0.00721399	0.01788274
rpoZ	-0.6263622	6.26183377	12.3403701	0.00722133	0.01789085
ydeL	-0.6021977	4.60133703	12.331568	0.00723507	0.01791483
yodH	0.92972894	1.82761464	12.2823593	0.00731252	0.01806599
ydeR	0.87019	2.41960327	12.2705183	0.00733131	0.01810226
yqfO	-0.7307696	6.51994606	12.2615205	0.00734563	0.0181173
ywcH	-0.7361058	3.94169751	12.241035	0.00737836	0.01816751
yomJ	-0.6297307	6.88512926	12.2179723	0.00741542	0.01824857
yezG	0.88066058	4.62731843	12.1935376	0.00745495	0.01832535
ctc	0.62767901	11.5761441	12.1878682	0.00746415	0.01833775
mntP	-0.6665738	3.22978631	12.1216038	0.00757282	0.01859436
yddS	-0.7118483	3.02675142	12.1171025	0.00758027	0.01859739
ltdD	0.72282142	4.53123896	12.1136783	0.00758595	0.01859739
gabD	0.61358271	8.60534558	12.1132123	0.00758672	0.01859739
yrvD	0.61323683	5.62569167	12.0862313	0.00763163	0.01869708
yozO	0.81637929	6.48140228	12.0094238	0.00776131	0.01895809
yhcQ	0.78437963	3.08134169	12.0058435	0.00776742	0.01895809
B4U62_RS231:	0.77184955	4.7106371	12.0057067	0.00776765	0.01895809
yxlH	-0.6164889	4.12465305	11.9866153	0.00780034	0.01902574
xhlA	0.67135362	6.06159151	11.971133	0.00782697	0.01908014
yuxJ	-0.9733959	6.95090262	11.9680672	0.00783226	0.01908247
tnrA	0.75491005	5.12094734	11.9535608	0.00785734	0.01913299
ythA	1.04857984	1.90276313	11.9063045	0.00793973	0.01930176
rpmH	-0.6109234	6.22132327	11.9062704	0.00793979	0.01930176
rapK	0.77546908	7.45540688	11.9032896	0.00794503	0.01930383
B4U62_RS108:	-3.3293177	-1.4370055	11.8980412	0.00795425	0.01930822
purC	0.76450087	7.63815967	11.8972731	0.0079556	0.01930822
yocR	0.58852254	6.53696643	11.80492	0.00812016	0.0196859
ywaF	1.37759588	0.59060681	11.7719745	0.00817988	0.01981978
csbC	0.88407234	11.8705814	11.7577493	0.00820583	0.01987174
yueE	0.73035272	7.4982725	11.7345606	0.00824837	0.01996376
rapE	0.77818784	7.80259835	11.6997848	0.00831266	0.02007526
yngA	-0.9024036	1.40304153	11.6748396	0.00835917	0.02015443
msmE	0.58988899	5.84189209	11.6625684	0.00838217	0.02019882
ywmC	1.17921428	1.54395292	11.5569109	0.00858345	0.02065872
yqaK	-1.9753132	-0.7333964	11.5527763	0.00859145	0.02065872
bsaA	1.10199939	3.60966403	11.5526099	0.00859177	0.02065872
B4U62_RS226:	1.52840842	1.17145559	11.4823071	0.00872921	0.02092019
xkdE	0.84459424	7.57641471	11.4404213	0.00881239	0.02107415
ydzF	0.6046074	3.66246306	11.4036639	0.0088862	0.02122761
yozG	0.65985972	3.97541134	11.371862	0.00895068	0.02133537
etfB	-1.010904	6.9131841	11.3419963	0.00901177	0.02145775
xth	-0.9589627	2.85203364	11.3233948	0.00905008	0.02152569

Table S5. Genes differentially expressed between ΔmnmE and WT in transcriptome analyses

ybdM	-0.5815561	4.62977838	11.2811025	0.00913793	0.02171121
yhfN	0.58109878	4.85963794	11.211111	0.00928566	0.02205032
yheD	0.80711287	2.90143449	11.1958817	0.0093182	0.02209186
rpmEB	0.74759852	8.63744343	11.1772821	0.00935813	0.02215659
ydeG	-0.7250827	2.17415372	11.1611037	0.00939304	0.02222145
scpB	-0.8896029	4.43796676	11.1113237	0.00950146	0.02245383
yvaV	0.8687955	5.76892712	11.0986536	0.00952931	0.02250756
hxlR	0.70766562	3.0792652	11.085785	0.00955769	0.0225625
B4U62_RS048	3.34356533	-1.4868191	11.0664456	0.00960055	0.0226394
ytzI	0.6229983	5.37630964	11.0562274	0.00962329	0.02268088
spoVK	-0.7242228	2.08901695	11.0055285	0.00973711	0.02292461
B4U62_RS120	-0.645518	2.96257152	10.9919327	0.00976791	0.02298486
yetH	0.81440001	2.68651949	10.9822204	0.00979	0.02302452
fliL	-0.5809629	6.73231347	10.9591482	0.0098427	0.02312378
yvkA	0.59510099	5.40117962	10.9515225	0.0098602	0.02315254
yvbU	0.60644163	4.13088693	10.8890132	0.0100051	0.02344279
pksE	0.63441879	7.41775271	10.8575624	0.010079	0.02359086
sunS	0.94899283	8.07843691	10.8283942	0.01014815	0.0237401
rsbX	0.65735401	12.0050706	10.7781181	0.01026873	0.02398397
licH	-1.3523643	5.75892968	10.7793413	0.01027658	0.02398959
gerPA	0.89520707	1.46852126	10.7380805	0.01036603	0.02418558
thiO	-0.5979316	2.63519254	10.703724	0.01045044	0.02431812
yqjB	0.87289475	1.70610632	10.6292587	0.01063635	0.02468553
B4U62_RS126	0.67070444	2.53190484	10.5736783	0.0107778	0.02497434
yxaJ	-0.8397307	4.2530338	10.4942797	0.01098395	0.02539858
yorG	-1.4852559	-0.1911625	10.4726269	0.01104102	0.02550297
yqiH	0.98247211	1.47630155	10.4705681	0.01104646	0.02550297
copA	0.58135274	6.26473739	10.4376942	0.01113386	0.02569129
yfmL	-0.6889168	4.21797221	10.4260683	0.01116498	0.02573634
yorD	-0.7998459	5.91624274	10.4063646	0.01121796	0.02582671
fosM	0.80545205	5.0652932	10.3933191	0.01125321	0.02587182
yoaS	0.85181844	3.80436377	10.2593487	0.01162332	0.02668095
B4U62_RS230	-1.1649492	1.54449636	10.1766706	0.01185931	0.02716601
glcU	1.21586153	0.74553507	10.1659379	0.01189038	0.02722303
yxeI	0.66380715	2.20038973	10.1267256	0.01200475	0.02745738
yjhA	0.97685384	1.43603128	10.1265717	0.0120052	0.02745738
yvgO	0.6450786	8.55156599	10.1206065	0.01202272	0.02746893
tcyL	1.59925613	0.19233203	10.0952769	0.01209747	0.0275898
B4U62_RS036	0.59599512	2.77363249	10.0941944	0.01210068	0.0275898
B4U62_RS229	0.67781119	3.77049082	10.0269246	0.0123021	0.02802003
ypeQ	0.77791197	3.72610266	9.97652797	0.01245573	0.02832601
B4U62_RS176	-1.3782699	8.16666889	10.3476353	0.01250434	0.0284204
yvaE	0.60356161	3.20014508	9.93491771	0.01258438	0.02852806
yfiF	0.6021542	2.63921558	9.93095	0.01259673	0.02852806
tcyK	1.54563306	0.7432507	9.93087017	0.01259698	0.02852806

Table S5. Genes differentially expressed between ΔmnmE and WT in transcriptome analyses

ephM	0.63186429	9.98890822	9.92047285	0.01262943	0.02856661
mdxF	0.90333187	3.26223633	9.91522797	0.01264583	0.02856661
ynaD	0.93346308	2.1375403	9.85866582	0.01282446	0.02891082
yckD	1.4360171	0.81260904	9.83236824	0.01290858	0.02905585
B4U62_RS221	-0.6401694	2.73130867	9.81799991	0.01295483	0.02913018
yokE	-0.7661722	3.45359393	9.72278052	0.01326661	0.02980082
B4U62_RS228	-1.1249943	1.44017741	9.70348626	0.01333092	0.02993001
desE	1.02468528	8.70802209	9.69899977	0.01334593	0.02994845
yjcM	-0.6385851	8.03349779	9.63903116	0.01354859	0.03038774
yceB	0.64348255	3.97697413	9.56162644	0.01381587	0.03092424
glpQA	0.80323861	2.43551958	9.54022007	0.01389094	0.03106071
acoL	1.85153294	4.28599441	9.89394403	0.0139103	0.03108821
B4U62_RS231	1.15743135	-0.0210464	9.52019842	0.01396162	0.03115549
efeM	0.70936623	6.98223374	9.50949269	0.0139996	0.03117706
ycbO	0.59730968	3.75665528	9.48907085	0.0140724	0.03129408
yncB	-0.9807273	1.99163098	9.47738345	0.01411427	0.03135318
ywbE	-0.5818781	2.83662181	9.42753206	0.01429464	0.03168992
B4U62_RS225	-0.9932875	2.47169589	9.40352997	0.0143825	0.03183664
yppC	0.91185769	1.01737655	9.30687406	0.01474322	0.03253701
B4U62_RS222	-1.4554715	0.27943331	9.27471777	0.01486572	0.03279093
rtpA	-0.9764565	1.86406578	9.27261923	0.01487376	0.03279224
ccpC	0.62009831	5.75288058	9.22845662	0.01504418	0.03313482
B4U62_RS227	1.10817644	1.70896953	9.22594228	0.01505396	0.03313978
B4U62_RS048	0.74705126	2.71971969	9.21958829	0.0150787	0.03317766
atpE	-0.6367633	8.47382626	9.20627213	0.01513072	0.03327549
pbpG	0.68162317	1.87003408	9.19822364	0.01516226	0.0333116
ydeP	-0.8044367	1.65324369	9.18905336	0.0151983	0.03337414
bsrH	-1.0300868	7.75662452	9.15140743	0.01534739	0.0336512
hupN	-1.2309301	-0.259871	9.14314292	0.01538037	0.03370672
ppeE	0.59178473	5.1025721	9.07979287	0.01563607	0.03417628
yqjF	0.77903775	5.40525739	9.06821551	0.01568337	0.03425149
B4U62_RS195	-0.994984	4.42424459	9.06205201	0.01570863	0.03427267
ssuB	-2.1070726	0.09746037	9.0500922	0.01575778	0.03432889
B4U62_RS110	-2.1334859	-1.2830013	9.0309035	0.01583704	0.03448451
yozV	-0.6979752	2.55426958	9.0285587	0.01584676	0.03448863
ywhL	0.71845869	2.53096137	9.00839242	0.01593066	0.0346541
gutB	-0.893428	1.68115099	8.96595058	0.01610905	0.03499031
yqcI	0.81095768	1.93302129	8.95429114	0.01615849	0.03506313
B4U62_RS207	0.59228613	4.30496238	8.91779285	0.01631449	0.03536679
yuzE	-0.5944326	2.44450351	8.85886951	0.01657032	0.03588424
tgl	-0.9335052	1.62501103	8.85664753	0.01658006	0.03588424
yhcU	-0.6296582	2.01806566	8.85534686	0.01658577	0.03588424
rsfA	0.74058348	1.81057049	8.85152391	0.01660256	0.03589944
yukE	0.69561373	6.80358992	8.85003749	0.0166091	0.03589944
ssuC	-1.3467533	0.93226729	8.80879571	0.01679167	0.03622297

Table S5. Genes differentially expressed between ΔmnmE and WT in transcriptome analyses

yydB	-0.7052073	6.50021937	8.80467418	0.01681005	0.03624488
B4U62_RS109(	-1.5320447	-0.8347761	8.78838705	0.01688294	0.03636644
ntdC	0.60262753	4.22902961	8.76218499	0.01700104	0.03660291
tlpA	-0.5978233	6.73590253	8.72128051	0.01718746	0.03696814
B4U62_RS108(	-1.4089578	-0.2375746	8.71092511	0.01723506	0.03705243
yjzD	-1.008909	4.96798882	8.64283016	0.01755219	0.03767903
B4U62_RS227:	-1.3515216	0.11718005	8.635006	0.01758909	0.03770313
yhxC	0.92635523	0.98645891	8.60623254	0.01772563	0.03797734
yebE	0.59836003	4.85122225	8.58544325	0.01782512	0.03817192
treC	-0.7475224	2.8132451	8.57196825	0.01788997	0.038255
edmS	1.87879596	-1.2336417	8.50713433	0.01820615	0.03883767
glpT	-1.3263501	1.11058645	8.50705589	0.01820654	0.03883767
xkdH	0.5959337	7.13892823	8.40149372	0.01873642	0.03977539
yrrI	0.66235007	1.91255787	8.31670077	0.01917605	0.04063036
kdgK	-0.6289404	4.68600574	8.29949699	0.01926682	0.04080304
yrdB	0.66234557	3.7601419	8.2966337	0.01928197	0.04081552
yxlC	-0.6876359	4.12406895	8.23387733	0.01961797	0.04146697
immA	0.79586818	5.3108891	8.22193307	0.01968275	0.041564
ykkC	-0.6327976	1.83257633	8.21634595	0.01971314	0.04160823
yceE	0.60434922	9.57784597	7.9336889	0.0213302	0.04461516
fadR	-0.9020651	7.44659021	7.91680724	0.02143193	0.04478542
cotR	0.6398559	2.1885526	7.86560356	0.02174422	0.0453338
B4U62_RS114:	-1.2239281	0.09319541	7.86534426	0.02174581	0.0453338
ytzH	1.66770614	0.14977065	7.85342052	0.02181936	0.0454656
B4U62_RS055:	-0.9431464	1.45404764	7.8466662	0.02186115	0.04553115
yteV	0.89730221	0.78702318	7.82863621	0.02197322	0.0457047
B4U62_RS097(	0.91366167	0.73777177	7.82825302	0.02197561	0.0457047
yycD	-1.3064102	1.64120185	7.81833395	0.02203758	0.04576875
capA	0.76987283	1.70515412	7.76700949	0.02236172	0.04633272
yhxD	-0.8167188	8.74805156	7.70921853	0.02273387	0.04688328
aseR	-0.7651502	2.16788369	7.69095327	0.0228531	0.04708507
licC	-1.2826511	5.756282	7.90638772	0.02298678	0.04729414
ylaC	-0.6944545	1.49061499	7.62637938	0.02328092	0.04783231
rplI	-0.6970629	5.93597815	7.57962965	0.02359692	0.04839481
B4U62_RS200(	0.7119701	5.56354683	7.57426467	0.02363353	0.04844378
B4U62_RS075(	-0.9751643	1.04525792	7.56924408	0.02366785	0.04849157
catD	0.80401752	1.22996825	7.46561659	0.02439036	0.04983282

Table S6 Output of ribosome profiling analysis generated using the HRIBO pipeline. The workbook is too large to be displayed as a PDF, please refer to the xlsx document.

Table S7. Genes with altered translation efficiency identified by ribosome profiling. The workbook is too large to be displayed as a PDF, please refer to the xlsx document.
